# Facile regiodivergent synthesis of spiro pyrrole-substituted pseudothiohydantoins and thiohydantoins via reaction of [*e*]-fused 1*H*-pyrrole-2,3-diones with thiourea

**DOI:** 10.3762/bjoc.15.280

**Published:** 2019-11-27

**Authors:** Aleksandr I Kobelev, Nikita A Tretyakov, Ekaterina E Stepanova, Maksim V Dmitriev, Michael Rubin, Andrey N Maslivets

**Affiliations:** 1 Department of Chemistry, Perm State University, ul. Bukireva 15, Perm 614990, Russian Federation; 2 Department of Chemistry, University of Kansas, 1567 Irving Hill Road, Lawrence, Kansas 66045, United States; 3 Department of Chemistry, North Caucasus Federal University, ul. Pushkina 1a, Stavropol 355009, Russian Federation

**Keywords:** diversity-oriented synthesis, hydantoin, nitrogen heterocycles, rearrangement, thiourea

## Abstract

A highly divergent synthesis of regioisomeric thiohydantoins and pseudothiohydantoins spiro-fused to a pharmacologically valuable pyrrole-2-one fragment involving the reaction of [*e*]-fused 1*H*-pyrrole-2,3-diones with thioureas was developed. The obtained spiro pseudothiohydantoin derivatives were found to undergo a pseudothiohydantoin–thiohydantoin rearrangement. The reactions were shown to proceed under catalyst-free conditions in good yields, and the products were isolated without applying preparative chromatography methods.

## Introduction

**Figure 1:**
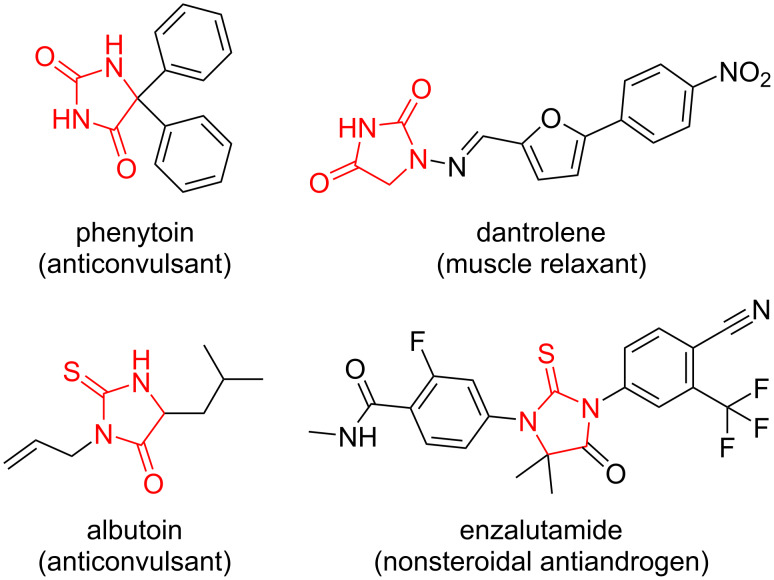
Hydantoin-based commercially available drugs.

Hydantoin (imidazolidine-2,4-dione) derivatives are omnipresent among biologically active compounds [1]. Many of them are commercially available drugs, for example, anticonvulsants phenytoin [2] and albutoin [3], the muscle relaxant dantrolene [4], or the nonsteroidal antiandrogen agent enzalutamide [5] (Figure 1).

Despite this fact, hydantoin derivatives belong to a special group of scaffolds in medicinal chemistry. Indeed, they are structurally related to rhodanine (2-thioxothiazolidin-4-one), and sometimes are classified as pan-assay interference compounds (PAINS), which are abhorrent in high-throughput screenings [6]. To clarify whether such compounds are privileged scaffolds or promiscuous binders, Mendgen and co-workers performed a systematic comparative study on rhodanines and related structures with respect to their medicinal chemistry properties [7]. As a result, it was shown that such structures could be suitable scaffolds for medicinal chemistry under proper conditions of biological evaluation, while their medicinal chemistry properties dramatically depend on the structure of the five-membered heterocyclic moiety and substituents in the C-5 position [7].

**Scheme 1:**
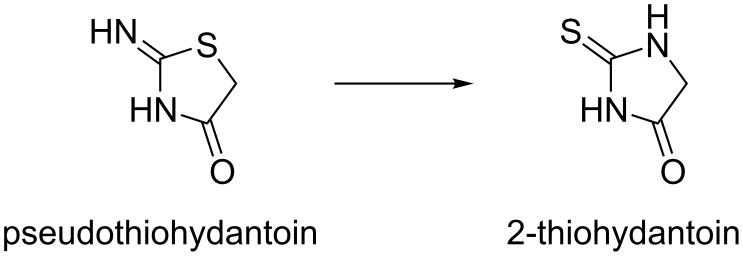
Pseudothiohydantoin–thiohydantoin rearrangement.

Some pseudothiohydantoins (2-iminothiazolidin-4-ones) are known to undergo a pseudothiohydantoin–thiohydantoin rearrangement (PTR, Scheme 1) [8–11], which is a special case of a quite poorly investigated iminothiolactone–thiolactam rearrangement [12–16]. This reaction offers attractive opportunities for the design of libraries of regioisomeric hydantoin-based compounds for drug discovery.

Spiro heterocycles are relatively new and insufficiently investigated structures in medicinal chemistry [17–18]. The introduction of spiro-fused cyclic moieties in small molecules increases the degree of their structural (shape) complexity, which may lead to the reduced binding promiscuity and improved clinical success [19]. Thus, recent development of 3D modelling methods facilitated investigations on the importance of 3D properties of small-molecular drug candidates [20–22] and inspired chemists to develop diversity-oriented methods for complex 3D molecules [23–24].

**Scheme 2:**
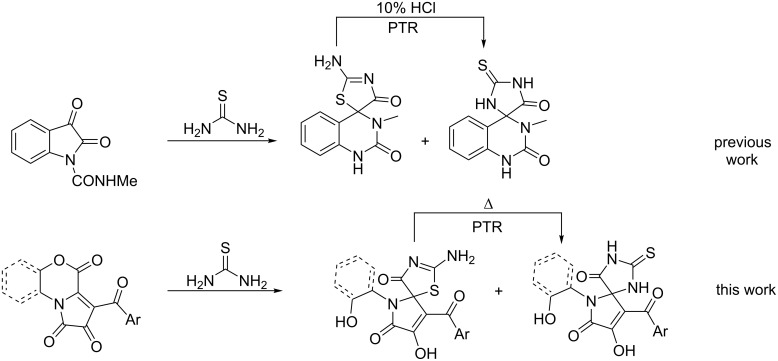
Syntheses of regioisomeric 5-spiro-substituted thiohydantoins and pseudothiohydantoins: previous [10] and this work.

Considering that to the best of our knowledge, only a sole report exists [10] that describes the synthesis of regioisomeric 5-spiro-substituted thiohydantoins and pseudothiohydantoins and their PTR (Scheme 2), we were encouraged to develop a divergent synthetic approach to access regioisomeric thiohydantoins and pseudothiohydantoins that are spiro-fused to a pharmacologically valuable pyrrole-2-one fragment and to investigate the scope and conditions of their PTR behavior (Scheme 2).

## Results and Discussion

**Figure 2:**
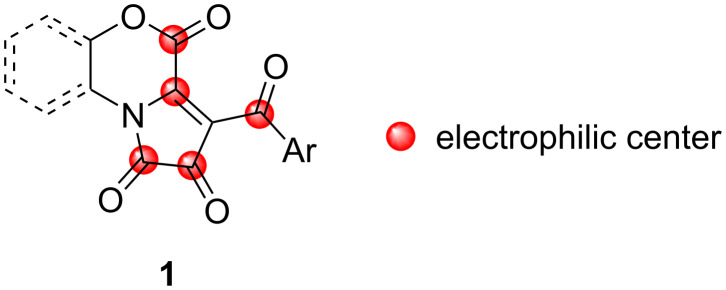
Electrophilic centers of FPDs **1**, starting materials of this study.

1*H*-Pyrrole-2,3-diones fused at [*e*]-side (FPDs) **1** can be viewed as a versatile polyelectrophilic synthetic platform (Figure 2), enabling facile preparation of libraries of heterocyclic molecules with an emphasis on skeletal diversity from a single set of reagents [25–27].

**Scheme 3:**
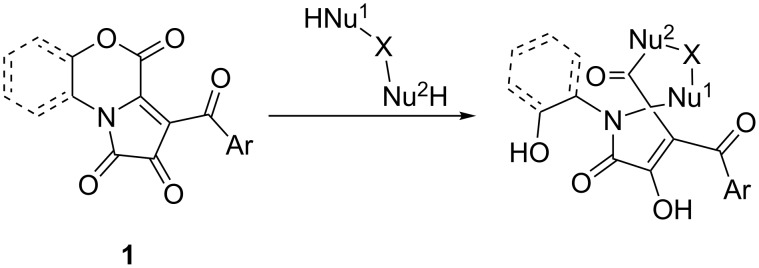
Cyclocondensation of FPDs **1** with 1,3-binucleophilic reagents, resulting in pyrrole-2-one-bearing spiro[4.4] heterocycles.

One of the most intriguing chemical properties of FPDs **1** is their ability to undergo a cyclocondensation with 1,3-binucleophilic reagents to form spiro[4.4] heterocycles bearing a pharmacophoric pyrrole-2-one moiety (Scheme 3) [28–29]. We employed this feature as a key step in the development of a straightforward and concise synthetic approach towards regioisomeric thiohydantoins and pseudothiohydantoins spiro-fused to a pyrrole-2-one fragment.

**Scheme 4:**
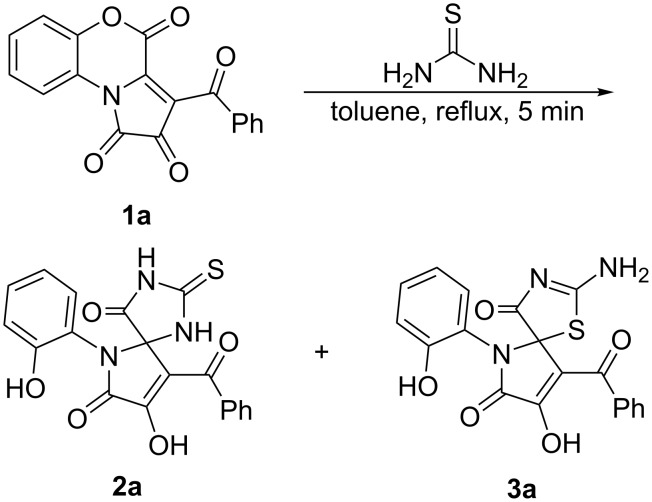
Reaction of FPD **1a** with thiourea.

To test the preparation of the target spiro-fused pseudothiohydantoins and thiohydantoins, we examined the reaction of FPD **1a** with thiourea by heating equimolar amounts of the reagents in toluene for 5 min (until the disappearance of the dark violet color of FPD **1a**). The reaction mixture was examined by UPLC–MS. Two major products with *m*/*z* = 396 ([M + H]^+^, ESI^+^) were observed in a ratio of ≈1:1, which corresponded to adducts of thiourea with FPD **1a**. The adducts were isolated, and their structures were elucidated as the desired spiro-fused thiohydantoin **2a** and pseudothiohydantoin **3a** (Scheme 4).

It should be pointed out that earlier, we have communicated initial data on this transformation [30–31]. However, taking into account more advanced structure elucidation methods employed in the present work (e.g., NMR, single crystal X-ray diffraction), we concluded that the structures of the products were identified incorrectly, and they are revised herein (for a detailed revision see Supporting Information File 1).

**Scheme 5:**
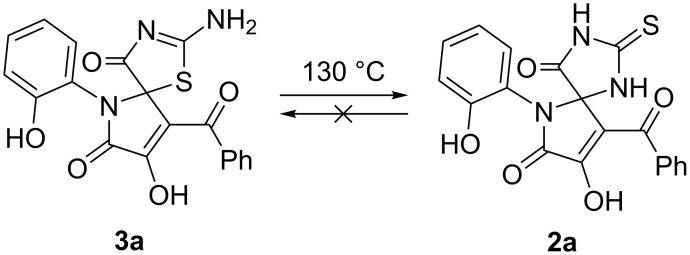
PTR of **3a** to **2a**.

Next, to find out whether compounds **2a** and **3a** were prone to undergo PTR, they were heated in open capillaries at 130 °C for 2 h, and the resulting mixtures were examined by UPLC–MS. As a result, we found that compound **2a** did not rearrange, and compound **3a** partially converted into its regioisomer **2a** due to irreversible PTR (Scheme 5).

Possibly, when carrying out the reaction of FPD **1a** with thiourea by refluxing the reaction mixture in toluene, part of the product **3a** was converted into **2a** because of PTR. We assumed that at lower temperatures, the main product of the reaction could be pseudothiohydantoin **3a**. To prove this assumption, we examined the reaction of FPD **1a** with thiourea at room temperature in various solvents (Table 1).

**Table 1:** Reaction of FPD **1a** with thiourea at room temperature in various solvents.^a^

Entry	Solvent	**2a**:**3a**^b^

1	acetone	30:70
2	ethyl acetate	5:95
3	1,4-dioxane	50:50
4	acetic acid	30:70
5	acetonitrile	50:50
6	butyl acetate	5:95
7	toluene	traces^c^

^a^Reaction mixtures were stirred until the disappearance of the dark violet color of FPD **1a** (about 2–4 h). ^b^According to UPLC–MS data. ^c^The reaction proceeded too slowly, and FPD **1a** underwent hydrolysis faster than reaction with thiourea (the reaction vessel was contaminated with water during samplings for analyses).

Obviously, the ratio of products **2a** and **3a** was dependent not only on the temperature of the reaction, but also on the polarity of the solvent. We found that optimal conditions for dominant formation of pseudothiohydantoin **3a** were applied by stirring the reaction mixture at room temperature in ethyl or butyl acetate (Table 1, entries 2 and 6).

For the development of a convenient procedure for thiohydantoin **2a** without isolation of its regioisomer **3a** being required, we carried out the reaction of FPD **1a** with thiourea in various solvents under reflux (Table 2).

**Table 2:** Reaction of FPD **1a** with thiourea at reflux in various solvents.^a^

Entry	Solvent	**2a**:**3a**^b^

1	acetone	30:70^c^
2	ethyl acetate	22:78^c^
3	1,4-dioxane	90:10^c^
4	1,4-dioxane	99:1^d^
5	acetic acid	30:70^c^
6	acetonitrile	25:75^c^
7	butyl acetate	95:5^c^
8	toluene	50:50^c^

^a^Reaction mixtures were refluxed until the disappearance of the dark violet color of FPD **1a** (about 5–15 min). ^b^According to UPLC–MS data. ^c^The reagents were mixed prior the heating. ^d^Thiourea was added to a boiling solution of FPD **1a**.

**Scheme 6:**
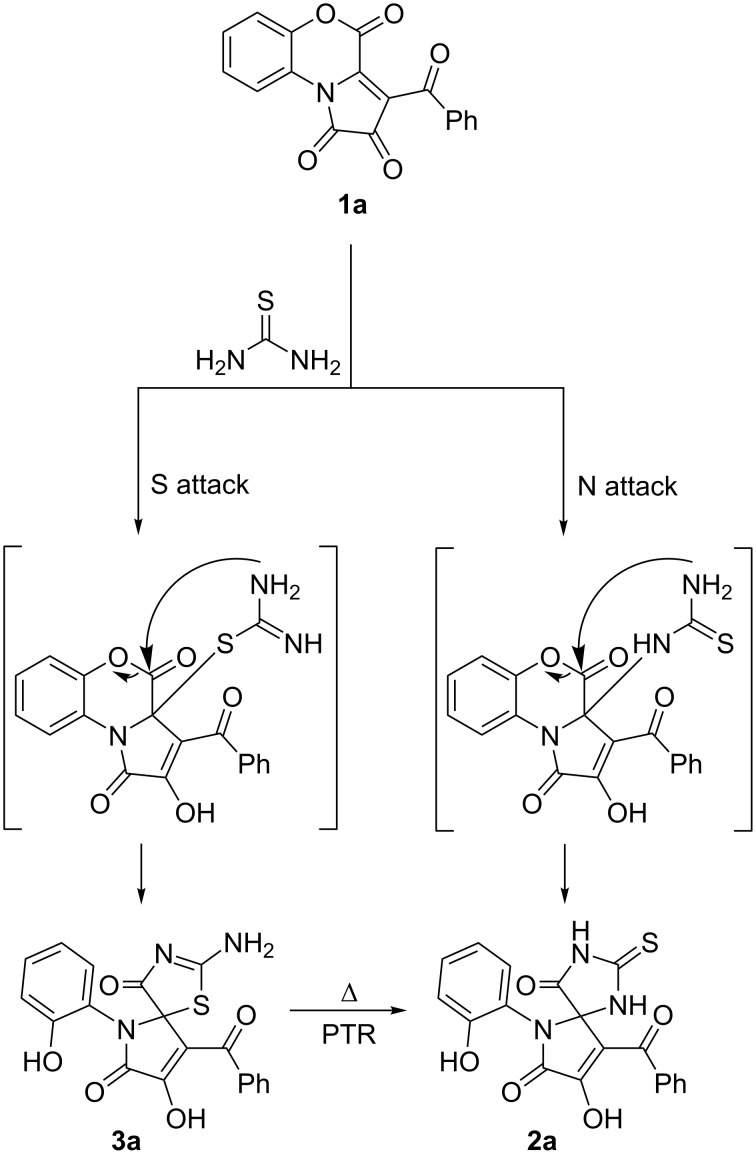
Pathways for the formation of compound **2a**.

As a result of this optimization, we established that thiohydantoin **2a** was formed as major product upon refluxing the reaction mixture in butyl acetate or 1,4-dioxane (Table 2, entries 3, 4, and 7). Interestingly, at different reagent ratios, the yield of compound **2a** was affected as well (Table 2, entries 3 and 4). When the reagents were mixed prior to heating (Table 2, entry 3), the yield of compound **2a** was lower than when the reagents were mixed in the boiling solvent (Table 2, entry 4). Probably, compound **2a** was formed not only as a result of the corresponding PTR, but as a result of a concurrent attack of the amino group of thiourea on the C-3a atom of FPD **1a** (Scheme 6).

Using the optimization data, we obtained a series of spiro-fused thiohydantoins **2a**–**m** and pseudothiohydantoins **3a**–**m** by utilizing various FPDs **1a**–**n** (Table 3). The aryl group-bearing FPDs **1a**–**m** reacted facilely to yield the desired compounds **2** and **3**, but ethoxycarbonyl group-bearing FPD **1n** reacted unselectively, giving a complex mixture of hardly identifiable products due to the occurrence of multiple side reactions involving the ethoxycarbonyl group.

**Table 3:** Series of spiro-fused thiohydantoins **2a**–**m** and pseudothiohydantoins **3a**–**m** from various FPDs **1a**–**n**.

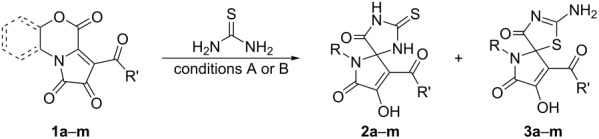				

Entry	R	R'	Yield of **2** (%)^a,b^	Yield of **3** (%)^a,c^

**a**	2-OH-C_6_H_4_	Ph	97 (CCDC 1952743)	79
**b**	5-Cl-2-OH-C_6_H_3_	Ph	78	87
**c**	2-OH-C_6_H_4_	4-OMe-C_6_H_4_	79	91
**d**	2-OH-C_6_H_4_	4-OEt-C_6_H_4_	90	79
**e**	2-OH-C_6_H_4_	4-Cl-C_6_H_4_	78	61 (CCDC 1952745)
**f**	2-OH-C_6_H_4_	4-Br-C_6_H_4_	87	61
**g**	2-OH-C_6_H_4_	4-Me-C_6_H_4_	89	89
**h**	5-NO_2_-2-OH-C_6_H_3_	Ph	95	58
**i**	2-OH-C_6_H_4_	4-NO_2_-C_6_H_4_	61	53
**j**	5-Br-2-OH-C_6_H_3_	Ph	65	59
**k**	2-OH-C_6_H_4_	4-F-C_6_H_4_	81	82
**l**	CH_2_CH_2_OH	4-Cl-C_6_H_4_	90 (CCDC 1952798)	97
**m**	CH_2_CH_2_OH	4-Me-C_6_H_4_	98	75
**n**	2-OH-C_6_H_4_	OEt	–	–

^a^Isolated yields. ^b^Conditions A: a mixture of FPD **1a**–**n** (1 mmol) and thiourea (1 mmol) was refluxed in 1,4-dioxane (5 mL) for 4 h. ^c^Conditions B: a mixture of FPD **1a**–**n** (1 mmol) and thiourea (1 mmol) was stirred in ethyl acetate (5 mL) for 12 h at room temperature.

**Scheme 7:**
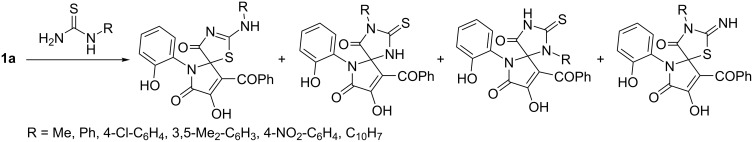
Reaction of FPD **1a** with monosubstituted thioureas.

Next, we examined the influence of substituents in the thiourea motif on its reaction with FPDs **1**. It was found that monosubstituted thioureas (*N*-methylthiourea, *N*-phenylthiourea, *N*-(4-chlorophenyl)thiourea, *N*-(3,5-dimethylphenyl)thiourea, *N*-(4-nitrophenyl)thiourea, and *N*-1-naphtylthiourea) reacted with FPD **1a** unselectively, forming a mixture of four inseparable adducts (the reaction mixtures were analyzed by UPLC–MS) (Scheme 7). Unfortunately, we did not succeed to find any conditions for a selective formation of either of them.

Unexpectedly, the implementation of *N*-acetylthiourea in the reaction with FPDs **1**, both under conditions A and B (see Table 3), afforded only one type of adducts, pseudothiohydantoins **4** (Table 4). Boiling compounds **4** in various solvents and heating them in open capillaries at 130 °C for 12 h did not provoke PTR, and compounds **4** remained unconverted. This phenomenon could be explained by the influence of the electron-withdrawing effect of the acetyl group, which reduced the nucleophilic properties of the acetyl-substituted nitrogen atom in *N*-acetylthiourea and decreased its reactivity, preventing the formation of regioisomeric compounds.

**Table 4:** ^b^Reaction of FPDs **1** with *N*-acetylthiourea.

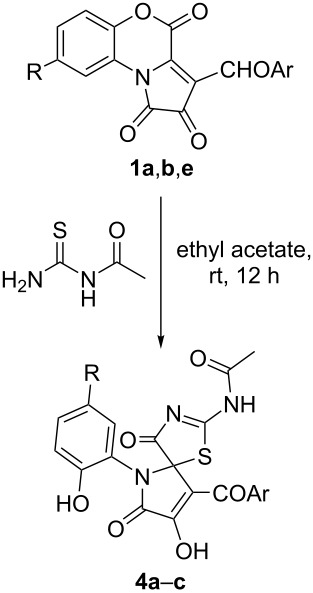			

Product	Ar	R	Yield of **4** (%)^a^

**4a**^b^	Ph	H	80
**4b**	4-Cl-C_6_H_4_	H	75
**4c**	Ph	Cl	79

^a^Isolated yields. ^b^CCDC 1952746.

**Scheme 8:**
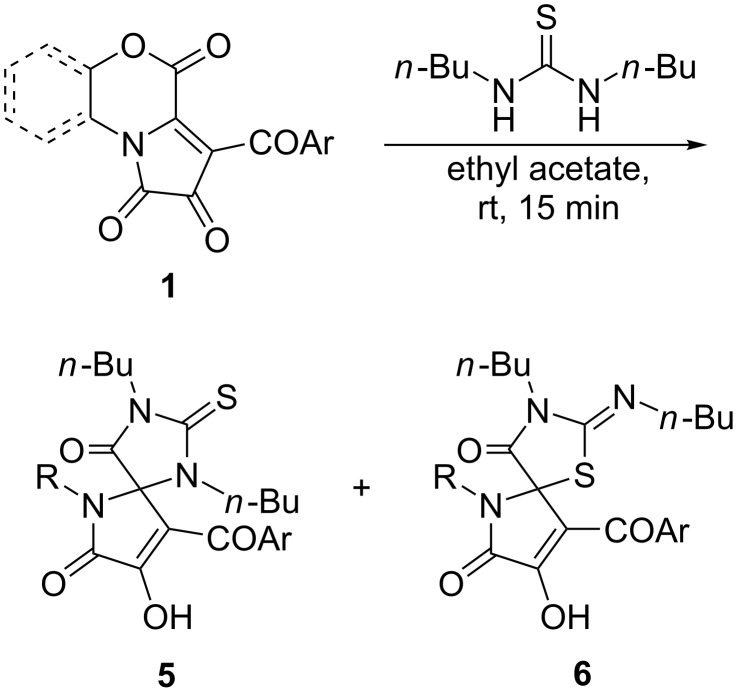
Reaction of FPDs **1** with 1,3-dibutylthiourea at room temperature.

1,3-Dibutylthiourea reacted smoothly at room temperature in ethyl acetate with FPDs **1** to form mixtures of products **5** and **6** (Scheme 8). Moreover, it was observed that the percentage of compounds **5** in reaction mixtures increased over time upon storage of the solutions. As such, **6** readily underwent PTR, affording the corresponding compounds **5**, but unfortunately, we did not succeed to isolate pseudothiohydantoins **6**.

The reaction of FPDs **1** with 1,3-dibutylthiourea in 1,4-dioxane resulted in exclusive formation of thiohydantoins **5** upon heating at reflux for 2–4 h (Table 5).

**Table 5:** Reaction of FPDs **1** with 1,3-dibutylthiourea at reflux.

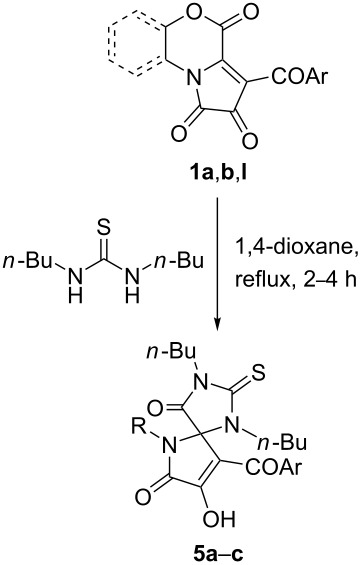			

Product	Ar	R	Yield of **5** (%)^a^

**5a**	Ph	2-OH-C_6_H_4_	75
**5b**^b^	Ph	5-Cl-2-OH-C_6_H_3_	76
**5c**	4-Cl-C_6_H_4_	CH_2_CH_2_OH	76

^a^Isolated yields. ^b^CCDC 1952744.

The facility of thiohydantoins **5** formation could be explained by the electron-donating effect of the butyl substituents, which increased the nucleophilicity of the butyl-substituted nitrogen atoms and promoted its attack on the electrophilic center C-3a of FPDs **1**.

The reaction of FPDs **1** with 1,3-diphenylthiourea proceeded in a similar manner. Thiohydantoins **7** were predominantly formed when the reaction was conducted at reflux in 1,4-dioxane, and pseudothiohydantoins **8** were formed as main products at room temperature (Table 6).

**Table 6:** Reaction of FPDs **1** with 1,3-diphenylthiourea.

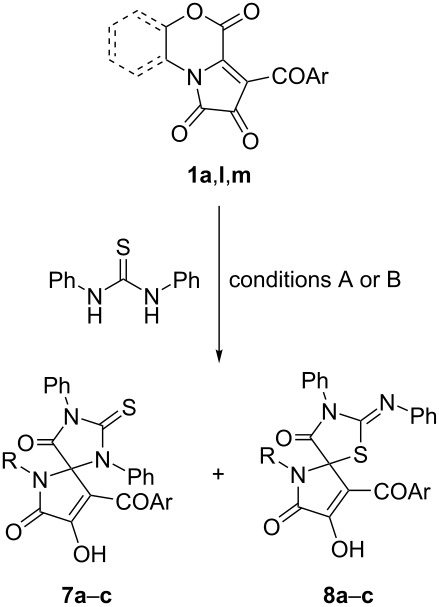				

Entry	Ar	R	Yield of **7** (%)^a,b^	Yield of **8** (%)^a,c^

**a**	Ph	2-OH-C_6_H_4_	71	78
**b**	4-Cl-C_6_H_4_	CH_2_CH_2_OH	63	82
**c**	4-Me-C_6_H_4_	CH_2_CH_2_OH	72	76

^a^Isolated yields. ^b^Conditions A: a mixture FPD **1** (1 mmol) and 1,3-diphenylthiourea (1 mmol) was refluxed in 1,4-dioxane (5 mL) for 8–12 h. ^c^Conditions B: a mixture FPD **1** (1 mmol) and 1,3-diphenylthiourea (1 mmol) was stirred in ethyl acetate (5 mL) for 12 h at room temperature.

Notably, the formation of thiohydantoins **7** required longer time in comparison with unsubstituted thiourea and 1,3-dibutylthiourea. Possible reasons for this are the influence of the steric hindrance induced by the phenyl substituents and weakening of the nucleophilic properties of the phenyl-substituted nitrogen atom, which prevented a nucleophilic attack of 1,3-diphenylthiourea on the electrophilic center C-3a of FPDs **1**.

**Scheme 9:**
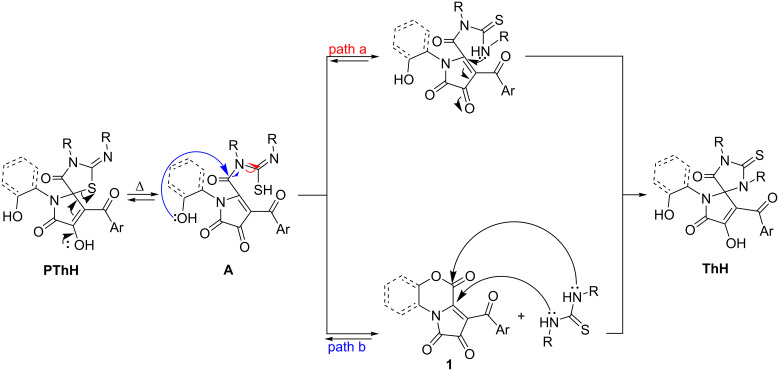
Plausible PTR pathways.

The observed PTRs could have proceed through two alternative pathways (Scheme 9), with the first stage being dissociation of the C_spiro_–S bond [32] in pseudothiohydantoins **PThH**, affording intermediate **A**. Then, **A** could have further undergone either an intramolecular cyclization by NH attack, forming thiohydantoin **ThH** (path A), or further dissociation to FPD **1** and thiourea. The latter would subsequently attack FPD **1** with both nucleophilic NH centers, forming thiohydantoin **ThH** (path B).

## Conclusion

In conclusion, we have developed a divergent approach to pharmaceutically interesting regiomeric thiohydantoins and pseudothiohydantoins spiro-fused to a pyrrole-2-one fragment via the reaction of [*e*]-fused 1*H*-pyrrole-2,3-diones with thioureas. The obtained pseudothiohydantoins were found to be prone to undergo a pseudothiohydantoin–thiohydantoin rearrangement. Therein, the substituents of the thiourea moiety were found to be crucial for tuning the conditions of this rearrangement. Notably, the reactions proceeded under catalyst-free conditions with good yields.

## Supporting Information

File 1 Experimental details, copies of ^1^H and ^13^C NMR spectra, X-ray crystallographic details, references to antimicrobial assay results, and a detailed revision of previously published structures.
