# Supplementary material for: Facile regiodivergent synthesis of spiro pyrrole-substituted pseudothiohydantoins and thiohydantoins via reaction of [e]-fused 1H-pyrrole-2,3-diones with thiourea
Source: Beilstein J Org Chem. 2019 Nov 27;15:2864–71. doi: 10.3762/bjoc.15.280 (PMC6902856; doi:10.3762/bjoc.15.280)
Supplement: File 1 — Experimental details, copies of 1H and 13C NMR spectra, X-ray crystallographic details, references to antimicrobial assay results, and a detailed revision of previously published structures. [file Beilstein_J_Org_Chem-15-2864-s001.pdf]

BEILSTEIN JOURNAL OF ORGANIC CHEMISTRY

Supporting Information

for

Facile regiodivergent synthesis of spiro pyrrole-substituted pseudothiohydantoins and thiohydantoins via reaction of [*e*]-fused 1*H*-pyrrole-2,3-diones with thiourea

Aleksandr I. Kobelev, Nikita A. Tretyakov, Ekaterina E. Stepanova, Maksim V. Dmitriev, Michael Rubin and Andrey N. Maslivets

Beilstein J. Org. Chem. **2019,** *15,* 2864–2871. doi:10.3762/bjoc.15.280

Experimental details, copies of ^{1}H and ^{13}C NMR spectra, X-ray crystallographic details, references to antimicrobial assay results, and a detailed revision of previously published structures

License and Terms: This is a supporting information file under the terms of the Creative Commons Attribution License (http://creativecommons.org/licenses/by/4.0). Please note that the reuse, redistribution and reproduction in particular requires that the authors and source are credited.
The license is subject to the *Beilstein Journal of Organic Chemistry* terms and conditions: (https://www.beilstein-journals.org/bjoc)

Table of contents

General……S3
Thiohydantoins **2a–m**……S4
Pseudothiohydantoins **3a–m**……S35
Pseudothiohydantoins **4a–c**……S65
Thiohydantoins **5a–c**……S73
Thiohydantoins **7a–c**……S81
Pseudothiohydantoins **8a–c**……S89
Crystal structure determination……S97
Biological evaluation of the reported products……S108
Comparison of structures of the compounds **2** and **3** reported in our previous works and in the present paper……S109
References……S114

Experimental

General

^{1}H, ^{13}C NMR spectra were acquired on a Bruker Avance-III HD 400 spectrometer in DMSO-d_6 using the solvent signal as an internal standard. IR spectra were recorded on a Perkin–Elmer Spectrum Two spectrometer from mulls in mineral oil. Melting points were measured on a Khimlabpribor PTP apparatus. X-ray crystallography was performed on an Xcalibur Ruby diffractometer. Elemental analyses were carried out on a Vario MICRO Cube analyzer. The reaction conditions were optimized using UPLC [Waters ACQUITY UPLC I-Class system; Acquity UPLC BEH C18 column, grain size of 1.7 μm; acetonitrile–water as eluents; flow rate of 0.6 mL/min; ACQUITY UPLC PDA eλ Detector (wavelength range of 230–780 nm); Xevo TQD mass detector; electrospray ionization; positive ion detection; ion source temperature of 150 °C; capillary voltage of 3500–4000 V; cone voltage of 20–70 V; vaporizer temperature of 150–300 °C]. Thin-layer chromatography (TLC) was performed on Merck silica gel 60 F_{254} plates using EtOAc/toluene, 1:5 v/v, toluene, EtOAc, MeOH/toluene, 1:3 v/v, MeOH/toluene, 1:5 v/v, MeOH/toluene, 1:10 v/v, MeOH as eluents. Starting FPDs **1a**–**n** were obtained according to reported procedures[1] from oxalyl chloride (purchased from commercial vendors) and heterocyclic enamines (obtained according to reported procedures[1] from commercially available reagents). Toluene and 1,4-dioxane were distilled over Na before the use. Ethyl acetate, butyl acetate and acetone were distilled over P_2O_5 before the use. Acetonitrile was dried over molecular sieves 4Å before the use. All other solvents and reagents were purchased from commercial vendors and were used as received.

Thiohydantoins 2a–m; General procedure

Thiourea (1.5 mmol, 115 mg) was added to a boiling solution of FPD **1a**–**m** (1.5 mmol) in anhydrous 1,4-dioxane (10 mL). The mixture was heated at reflux for 2–4 h, cooled to room temperature. The solvent was evaporated, and the resulting residue was triturated with toluene (15 mL). The formed solid was filtered off and recrystallized from methanol or toluene to afford the desired thiohydantoin **2a–m** (if the product still contains impurities after the recrystallization, it can be purified by an additional recrystallization from ethyl acetate (yields solvates), acetonitrile, methanol or toluene).

9-Benzoyl-8-hydroxy-6-(2-hydroxyphenyl)-2-thioxo-1,3,6-triazaspiro[4.4]non-8-ene-4,7-dione (2a)

Yield: 575 mg (97%); yellow solid; mp 165–167 °C (decomp.).

^{1}H NMR (400 MHz, DMSO-*d*$_6$): δ = 11.88 (s, 1 H), 10.10 (s, 1 H), 9.80 (br.s, 1 H), 7.77 (m, 2 H), 7.61 (m, 1 H), 7.52 (m, 2 H), 7.25 (m, 1 H), 6.98 (m, 2 H), 6.84 (m, 1 H).

^{13}C NMR (100 MHz, DMSO-*d*$_6$): δ = 187.7, 183.4, 171.7, 164.2, 154.8, 137.7, 132.4, 130.4, 129.5, 128.7 (2 C), 128.1, 127.9 (2 C), 119.9, 119.0, 116.6, 113.2, 80.6.

IR (mineral oil): 3442, 3290, 1764, 1727, 1671 cm^{-1}.

MS (ESI+): m/z calcd for $C_{19}H_{13}N_3O_5S+H^+$: 396.07 [M+H$^+$]; found: 395.97.

Anal. Calcd (%) for $C_{19}H_{13}N_3O_5S$: C 57.72; H 3.31; N 10.63. Found: C 57.99; H 3.47; N 10.41.

9-Benzoyl-6-(4-chloro-2-hydroxyphenyl)-8-hydroxy-2-thioxo-1,3,6-triazaspiro[4.4]non-8-ene-4,7-dione (2b)

Yield: 502 mg (78%); yellow solid; mp 183–185 °C (decomp.).

^{1}H NMR (400 MHz, DMSO-*d*$_6$): δ = 12.01 (s, 1 H), 10.25 (br.s, 1 H), 10.20 (s, 1 H), 7.77 (m, 2 H), 7.62 (m, 1 H), 7.52 (m, 2 H), 7.33 (m, 1 H), 7.01 (m, 2 H).

^{13}C NMR (100 MHz, DMSO-*d*$_6$): δ = 187.8, 183.5, 171.5, 164.1, 154.1, 137.5, 132.6, 130.4, 129.0, 128.8 (2 C), 128.0 (2 C), 127.3, 121.7, 120.9, 118.1, 113.6, 80.4.

IR (mineral oil): 3232, 1758, 1737, 1721, 1673 cm^{-1}.

MS (ESI+): m/z calcd for $C_{19}H_{12}ClN_3O_5S+H^+$: 430.03 [M+H$^+$]; found: 430.13.

Anal. Calcd (%) for $C_{19}H_{12}ClN_3O_5S$: C 53.09; H 2.81; N 9.78. Found: C 52.82; H 2.92; N 9.81.

8-Hydroxy-6-(2-hydroxyphenyl)-9-(4-methoxybenzoyl)-2-thioxo-1,3,6-triazaspiro[4.4]non-8-ene-4,7-dione (2c)

Yield: 504 mg (79%); yellow solid; mp 140–143 °C (decomp.).

^{1}H NMR (400 MHz, DMSO-d_6): δ = 11.88 (s, 1 H), 10.12 (br.s, 1 H), 9.81 (s, 1 H), 7.80 (m, 2 H), 7.25 (m, 1 H), 7.06 (m, 2 H), 6.97 (m, 2 H), 6.83 (m, 1 H), 3.86 (s, 3 H).

^{13}C NMR (100 MHz, DMSO-d_6): δ = 186.3, 183.4, 171.6, 164.1, 163.0, 154.8, 131.3 (2 C), 130.4, 130.0, 129.4, 119.8, 119.0, 116.6, 114.2, 113.3 (3 C), 80.6, 55.5.

IR (mineral oil): 3306, 3196, 1748, 1733, 1674 cm^{-1}.

MS (ESI+): m/z calcd for $C_{20}H_{15}N_3O_6S+H^+$: 426.08 [M+H$^+$]; found: 426.16.

Anal. Calcd (%) for $C_{20}H_{15}N_3O_6S$: C 56.47; H 3.55; N 9.88. Found: C 56.65; H 3.56; N 9.82.

9-(4-Ethoxybenzoyl)-8-hydroxy-6-(2-hydroxyphenyl)-2-thioxo-1,3,6-triazaspiro[4.4]non-8-ene-4,7-dione (2d)

Yield: 593 mg (90%); yellow solid; mp 163–165 °C (decomp.).

^{1}H NMR (400 MHz, DMSO-d_6): δ = 11.88 (s, 1 H), 10.12 (br.s, 1 H), 9.81 (s, 1 H), 7.79 (m, 2 H), 7.25 (m, 1 H), 7.04 (m, 2 H), 6.97 (m, 2 H), 6.83 (m, 1 H), 4.15 (q, J = 7.1 Hz, 2 H), 1.36 (t, J = 7.1 Hz, 3 H).

^{13}C NMR (100 MHz, DMSO-d_6): δ = 186.2, 183.5, 171.6, 164.1, 162.3, 154.8, 131.4 (2 C), 130.4, 129.8, 129.5, 119.8, 119.0, 116.6, 114.5, 114.3, 113.7 (2 C), 80.6, 63.5, 14.4.

IR (mineral oil): 3318, 3200, 1765, 1726, 1672 cm^{-1}.

MS (ESI+): m/z calcd for $C_{21}H_{17}N_3O_6S+H^+$: 440.09 [M+H$^+$]; found: 440.12.

Anal. Calcd (%) for $C_{21}H_{17}N_3O_6S$: C 57.40; H 3.90; N 9.56. Found: C 57.53; H 4.01; N 9.55.

9-(4-Chlorobenzoyl)-8-hydroxy-6-(2-hydroxyphenyl)-2-thioxo-1,3,6-triazaspiro[4.4]non-8-ene-4,7-dione (2e)

Yield: 502 mg (78%); yellow solid; mp 195–198 °C (decomp.).

^{1}H NMR (400 MHz, DMSO-d_6): δ = 11.89 (s, 1 H), 10.07 (s, 1 H), 9.80 (br.s, 1 H), 7.78 (m, 2 H), 7.59 (m, 2 H), 7.25 (m, 1 H), 6.97 (m, 2 H), 6.82 (m, 1 H).

^{13}C NMR (100 MHz, DMSO-d_6): δ = 186.4, 183.4, 171.7, 164.1, 154.8, 137.1, 136.5, 130.6 (2 C), 130.4, 129.4, 128.1 (2 C), 119.8, 119.0, 116.6 (2 C), 112.8, 80.5.

IR (mineral oil): 3316, 1769, 1707, 1675 cm^{-1}.

MS (ESI+): m/z calcd for $C_{19}H_{12}ClN_3O_5S+H^+$: 430.03 [M+H$^+$]; found: 430.05.

Anal. Calcd (%) for $C_{19}H_{12}ClN_3O_5S$: C 53.09; H 2.81; N 9.78. Found: C 53.35; H 2.99; N 9.79.

9-(4-Bromobenzoyl)-8-hydroxy-6-(2-hydroxyphenyl)-2-thioxo-1,3,6-triazaspiro[4.4]non-8-ene-4,7-dione (2f)
Yield: 619 mg (87%); yellow solid; mp 155–157 °C (decomp.).
^{1}H NMR (400 MHz, DMSO-d_6): δ = 11.87 (s, 1 H), 10.06 (s, 1 H), 9.79 (br.s, 1 H), 7.71 (m, 4 H), 7.25 (m, 1 H), 6.97 (m, 2 H), 6.82 (m, 1 H).
^{13}C NMR (100 MHz, DMSO-d_6): δ = 186.0, 183.2, 172.1, 164.7, 154.8, 137.2, 130.9 (2 C), 130.7 (2 C), 130.3, 129.5, 125.8, 120.1, 119.0, 116.6, 114.5, 111.8, 80.6.
IR (mineral oil): кох 3292, 3200, 1760, 1736, 1672 cm^{-1}.
MS (ESI+): m/z calcd for $C_{19}H_{12}BrN_3O_5S+H^+$: 473.98, 475.97 [M+H$^+$]; found: 474.06, 475.99.
Anal. Calcd (%) for $C_{19}H_{12}BrN_3O_5S$: C 48.12; H 2.55; N 8.86. Found: C 48.38; H 2.42; N 8.91.

8-Hydroxy-6-(2-hydroxyphenyl)-9-(4-methylbenzoyl)-2-thioxo-1,3,6-triazaspiro[4.4]non-8-ene-4,7-dione (2g)
Yield: 546 mg (89%); yellow solid; mp 198–201 °C (decomp.).
^{1}H NMR (400 MHz, DMSO-d_6): δ = 11.91 (s, 1 H), 10.14 (s, 1 H), 9.83 (br.s, 1 H), 7.69 (m, 2 H), 7.33 (m, 2 H), 7.25 (m, 2 H), 6.98 (m, 2 H), 6.83 (m, 1 H), 2.40 (s, 3 H).
^{13}C NMR (100 MHz, DMSO-d_6): δ = 187.5, 183.5, 171.6, 164.0, 154.8, 143.0, 134.9, 130.4, 129.4, 129.0 (2 C), 128.6 (2 C), 119.8, 119.0, 116.6, 115.6, 114.0, 80.5, 21.1.
IR (mineral oil): 3300, 3200, 1758, 1733, 1673 cm^{-1}.
MS (ESI+): m/z calcd for $C_{20}H_{15}N_3O_5S+H^+$: 410.08 [M+H$^+$]; found: 410.12.
Anal. Calcd (%) for $C_{20}H_{15}N_3O_5S$: C 58.67; H 3.69; N 10.26. Found: C 58.37; H 3.76; N 10.34.

9-Benzoyl-8-hydroxy-6-(2-hydroxy-5-nitrophenyl)-2-thioxo-1,3,6-triazaspiro[4.4]non-8-ene-4,7-dione (2h)
Yield: 627 mg (95%); yellow solid; mp 239–241 °C (decomp.).
^{1}H NMR (400 MHz, DMSO-d_6): δ = 12.05 (s, 1 H), 11.77 (s, 1 H), 10.32 (br.s, 1 H), 8.23 (m, 1 H), 7.96 (m, 1 H), 7.78 (m, 2 H), 7.62 (m, 1 H), 7.52 (m, 2 H), 7.17 (m, 1 H).
^{13}C NMR (100 MHz, DMSO-d_6): δ = 187.8, 183.6, 171.5, 164.2, 161.6, 154.4, 139.0, 137.5, 132.6, 128.8 (2 C), 128.0 (2 C), 126.8, 125.9, 120.1, 117.0, 113.7, 80.4.
IR (mineral oil): 3272, 3099, 1756, 1736, 1678 cm^{-1}.

MS (ESI+): m/z calcd for $C_{19}H_{12}N_4O_7S+H^+$: 441.05 [M+H$^+$]; found: 441.15.
Anal. Calcd (%) for $C_{19}H_{12}N_4O_7S$: C 51.82; H 2.75; N 12.72. Found: C 51.99; H 2.76; N 12.73.

8-Hydroxy-6-(2-hydroxyphenyl)-9-(4-nitrobenzoyl)-2-thioxo-1,3,6-triazaspiro[4.4]non-8-ene-4,7-dione (2i)
Yield: 403 mg (61%); yellow solid; mp 210–213 °C (decomp.).
^{1}H NMR (400 MHz, DMSO-d_6): δ = 11.78 (s, 1 H), 9.97 (s, 1 H), 9.71 (br.s, 1 H), 8.31 (m, 2 H), 7.94 (m, 2 H), 7.24 (m, 1 H), 6.98 (m, 2 H), 6.82 (m, 1 H).
^{13}C NMR (100 MHz, DMSO-d_6): δ = 184.5, 183.1, 172.3, 164.9, 154.7, 148.9, 144.2, 130.2, 129.8 (3 C), 129.5, 123.0 (2 C), 120.3, 119.0, 116.6, 110.5, 80.6.
IR (mineral oil): 3323, 3260, 1763, 1740, 1680 cm^{-1}.
MS (ESI+): m/z calcd for $C_{19}H_{12}N_4O_7S+H^+$: 441.05 [M+H$^+$]; found: 441.08.
Anal. Calcd (%) for $C_{19}H_{12}N_4O_7S$: C 51.82; H 2.75; N 12.72. Found: C 51.65; H 2.78; N 12.78.

9-Benzoyl-6-(5-bromo-2-hydroxyphenyl)-8-hydroxy-2-thioxo-1,3,6-triazaspiro[4.4]non-8-ene-4,7-dione (2j)
Yield: 462 mg (65%); yellow solid; mp 192–193 °C (decomp.).
^{1}H NMR (400 MHz, DMSO-d_6): δ = 11.97 (s, 1 H), 10.25 (br.s, 1 H), 10.16 (s, 1 H), 7.78 (m, 2 H), 7.50 (m, 4 H), 7.13 (m, 1 H), 6.95 (m, 1 H).
^{13}C NMR (100 MHz, DMSO-d_6): δ = 187.5, 183.4, 171.7, 164.4, 154.5, 137.7, 133.1, 132.3, 131.8, 128.8 (2 C), 127.9 (2 C), 125.7, 121.5, 118.6, 113.0, 108.8, 80.6.
IR (mineral oil): 3333, 3176, 1753, 1722, 1671 cm^{-1}.
MS (ESI+): m/z calcd for $C_{19}H_{12}BrN_3O_5S+H^+$: 473.98, 475.97 [M+H$^+$]; found: 474.04, 475.99.
Anal. Calcd (%) for $C_{19}H_{12}BrN_3O_5S$: C 48.12; H 2.55; N 8.86. Found: C 47.75; H 2.56; N 8.81.

9-(4-Fluorobenzoyl)-8-hydroxy-6-(2-hydroxyphenyl)-2-thioxo-1,3,6-triazaspiro[4.4]non-8-ene-4,7-dione (2k)
Yield: 502 mg (81%); yellow solid; mp 222–225 °C (decomp.).
^{1}H NMR (400 MHz, DMSO-d_6): δ = 11.88 (s, 1 H), 10.07 (s, 1 H), 9.81 (br.s, 1 H), 7.86 (m, 2 H), 7.34 (m, 2 H), 7.25 (m, 1 H), 6.97 (m, 2 H), 6.83 (m, 1 H).

^{13}C NMR (100 MHz, DMSO-d_6): δ = 186.1, 183.4, 171.8, 165.8, 164.2, 163.3, 154.8, 134.3, 131.7, 131.6, 130.4, 129.5, 119.9, 119.0, 116.6, 115.1, 114.9, 113.0, 80.6.
IR (mineral oil): 3179, 1752, 1716, 1669 cm^{-1}.
MS (ESI+): m/z calcd for $C_{19}H_{12}FN_3O_5S+H^+$: 414.06 [M+H$^+$]; found: 414.16.
Anal. Calcd (%) for $C_{19}H_{12}FN_3O_5S$: C 55.21; H 2.93; N 10.17. Found: C 55.46; H 3.17; N 10.28.

9-(4-Chlorobenzoyl)-8-hydroxy-6-(2-hydroxyethyl)-2-thioxo-1,3,6-triazaspiro[4.4]non-8-ene-4,7-dione (2l)
Yield: 516 mg (90%); yellow solid; mp 192–194 °C (decomp.).
^1H NMR (400 MHz, DMSO-d_6): δ = 12.25 (s, 1 H), 10.16 (s, 1 H), 7.71 (m, 2 H), 7.56 (m, 2 H), 3.53–3.40 (m, 2 H), 3.32 (m, 1 H), 3.09 (m, 1 H).
^{13}C NMR (100 MHz, DMSO-d_6): δ = 186.1, 183.7, 171.9, 164.9, 155.7, 137.2, 136.3, 130.5 (2 C), 128.1 (2 C), 112.6, 79.0, 57.9, 42.0.
IR (mineral oil): 3340, 1749, 1710, 1688 cm^{-1}.
MS (ESI+): m/z calcd for $C_{15}H_{12}ClN_3O_5S+H^+$: 382.03 [M+H$^+$]; found: 382.06.
Anal. Calcd (%) for $C_{15}H_{12}ClN_3O_5S$: C 47.19; H 3.17; N 11.01. Found: C 47.47; H 3.05; N 11.03.

8-Hydroxy-6-(2-hydroxyethyl)-9-(4-methylbenzoyl)-2-thioxo-1,3,6-triazaspiro[4.4]non-8-ene-4,7-dione (2m)
Yield: 531 mg (98%); yellow solid; mp 176–178 °C (decomp.).
^1H NMR (400 MHz, DMSO-d_6): δ = 12.26 (s, 1 H), 10.20 (s, 1 H), 7.62 (m, 2 H), 7.30 (m, 2 H), 3.54–3.40 (m, 2 H), 3.33 (m, 1 H), 3.08 (m, 1 H), 2.38 (s, 3 H).
^{13}C NMR (100 MHz, DMSO-d_6): δ = 187.2, 183.8, 171.8, 164.9, 153.9, 143.0, 134.8, 128.9 (2 C), 128.6 (2 C), 113.7, 79.1, 57.9, 42.1, 21.1.
IR (mineral oil): 3270, 1754, 1717, 1672 cm^{-1}.
MS (ESI+): m/z calcd for $C_{16}H_{15}N_3O_5S+H^+$: 362.08 [M+H$^+$]; found: 362.00.
Anal. Calcd (%) for $C_{16}H_{15}N_3O_5S$: C 53.18; H 4.18; N 11.63. Found: C 52.96; H 4.15; N 11.59.

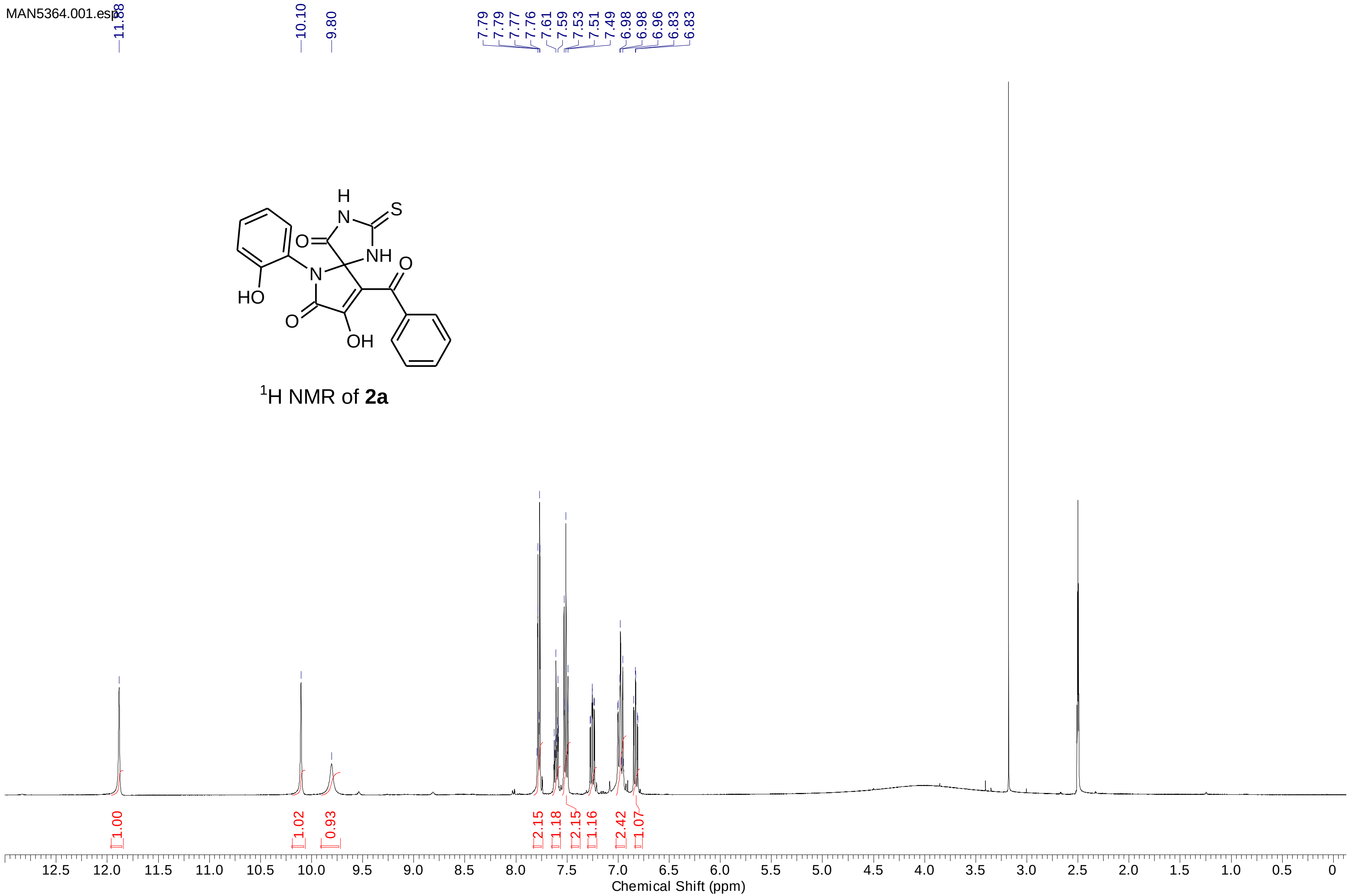
MAN5364.001.esp
11.88
10.10
9.80
7.79
7.79
7.77
7.76
7.61
7.59
7.53
7.51
7.49
6.98
6.98
6.96
6.83
6.83
1.00
1.02
0.93
2.15
1.18
2.15
1.16
2.42
1.07
Chemical Shift (ppm)

^{1}H NMR of **2a**

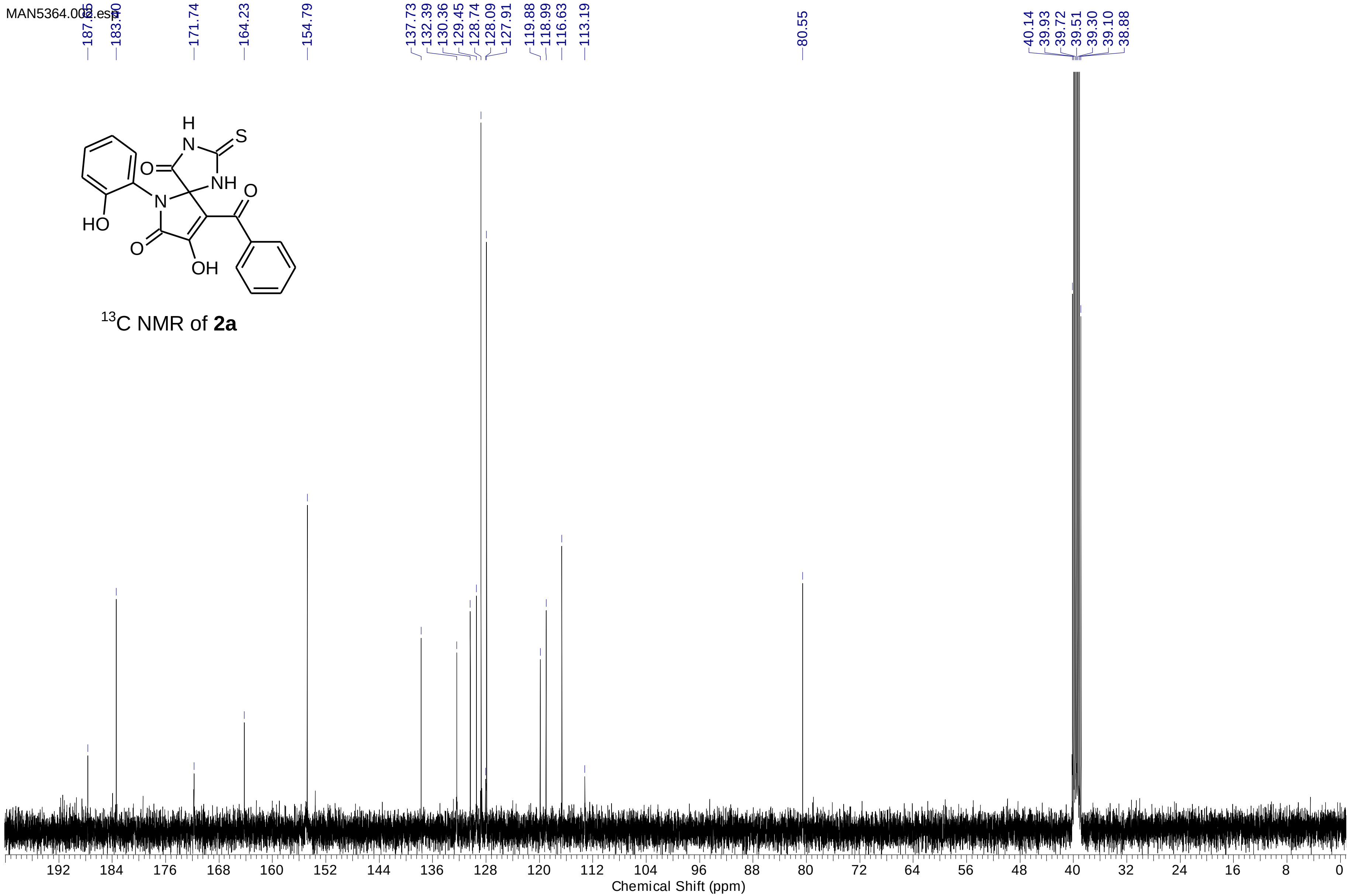
MAN5364.002.esp
187.65
183.40
171.74
164.23
154.79
137.73
132.39
130.36
129.45
128.74
128.09
127.91
119.88
118.99
116.63
113.19
80.55
40.14
39.93
39.72
39.51
39.30
39.10
38.88
H
N
S
O
NH
O
N
HO
O
OH
^{13}C NMR of **2a**
192
184
176
168
160
152
144
136
128
120
112
104
96
88
80
72
64
56
48
40
32
24
16
8
0
Chemical Shift (ppm)

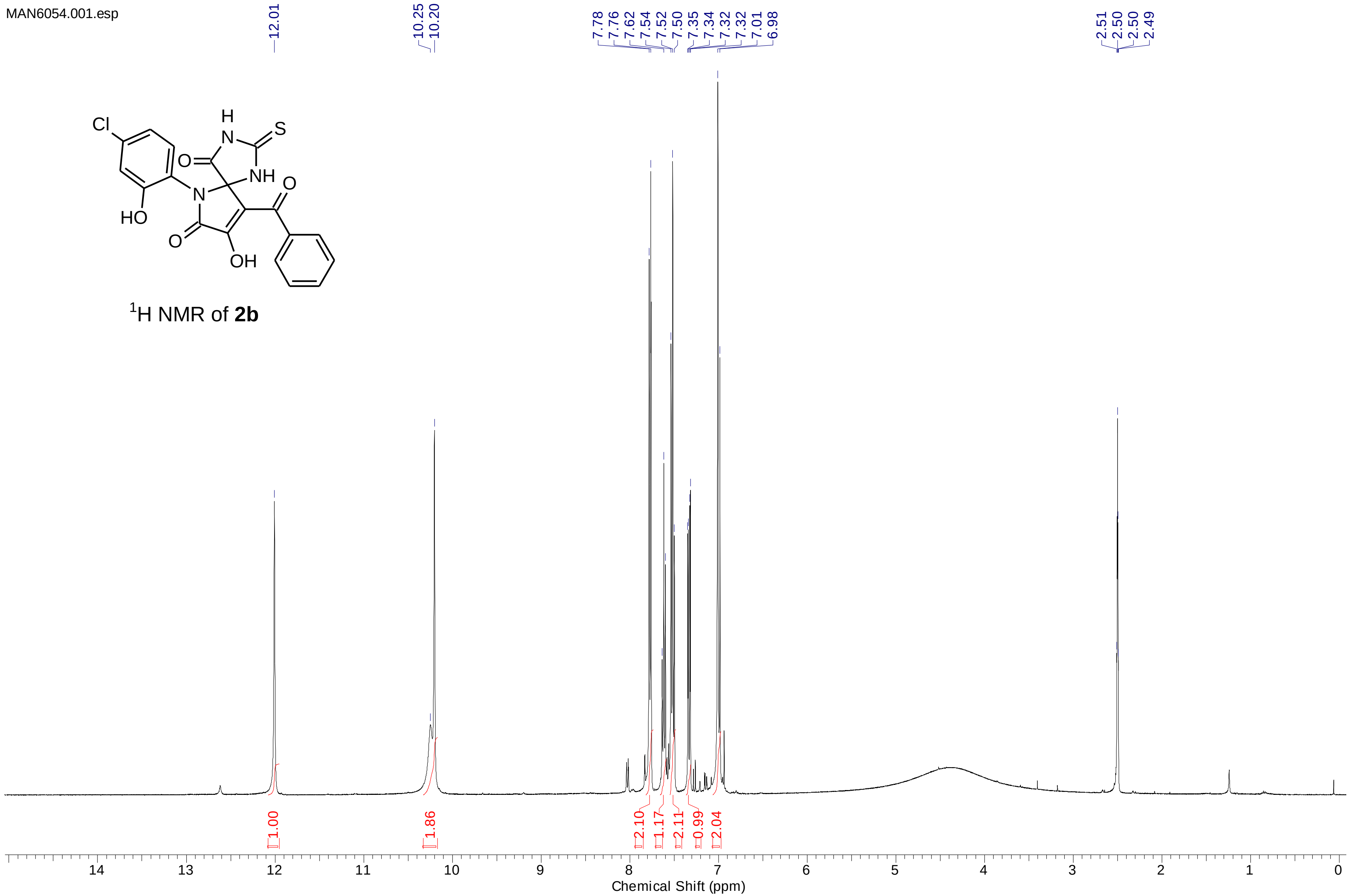

MAN6054.001.esp
12.01
10.25
10.20
7.78
7.76
7.62
7.54
7.52
7.50
7.35
7.34
7.32
7.32
7.01
6.98
2.51
2.50
2.50
2.49
1.00
1.86
2.10
1.17
2.11
0.99
2.04
Chemical Shift (ppm)

^{1}H NMR of **2b**

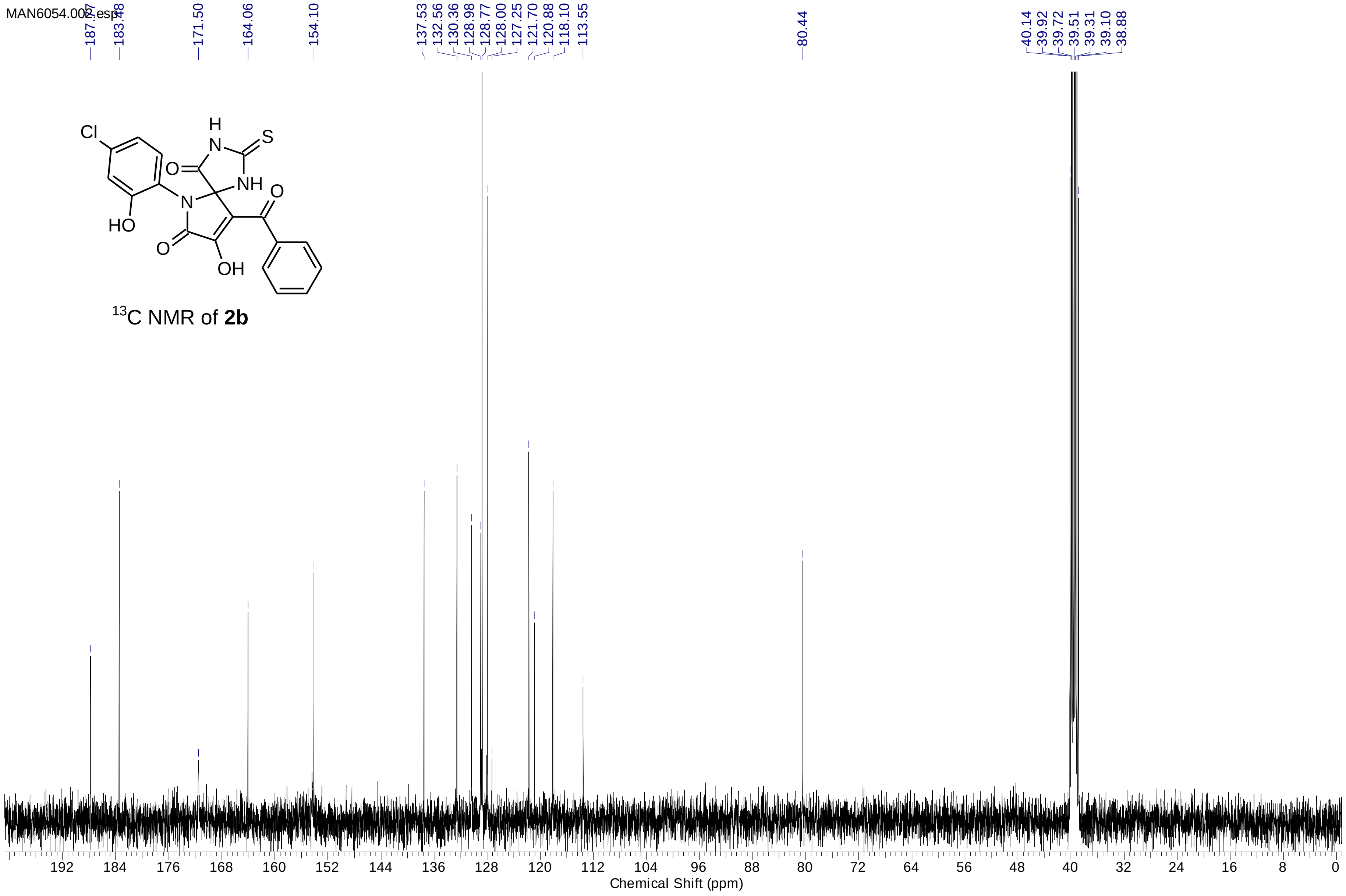
MAN6054.002.esp
187.77
183.48
171.50
164.06
154.10
137.53
132.56
130.36
128.98
128.77
128.00
127.25
121.70
120.88
118.10
113.55
80.44
40.14
39.92
39.72
39.51
39.31
39.10
38.88
Cl
H
N
S
O
NH
O
N
HO
O
OH
^{13}C NMR of **2b**
192
184
176
168
160
152
144
136
128
120
112
104
96
88
80
72
64
56
48
40
32
24
16
8
0
Chemical Shift (ppm)

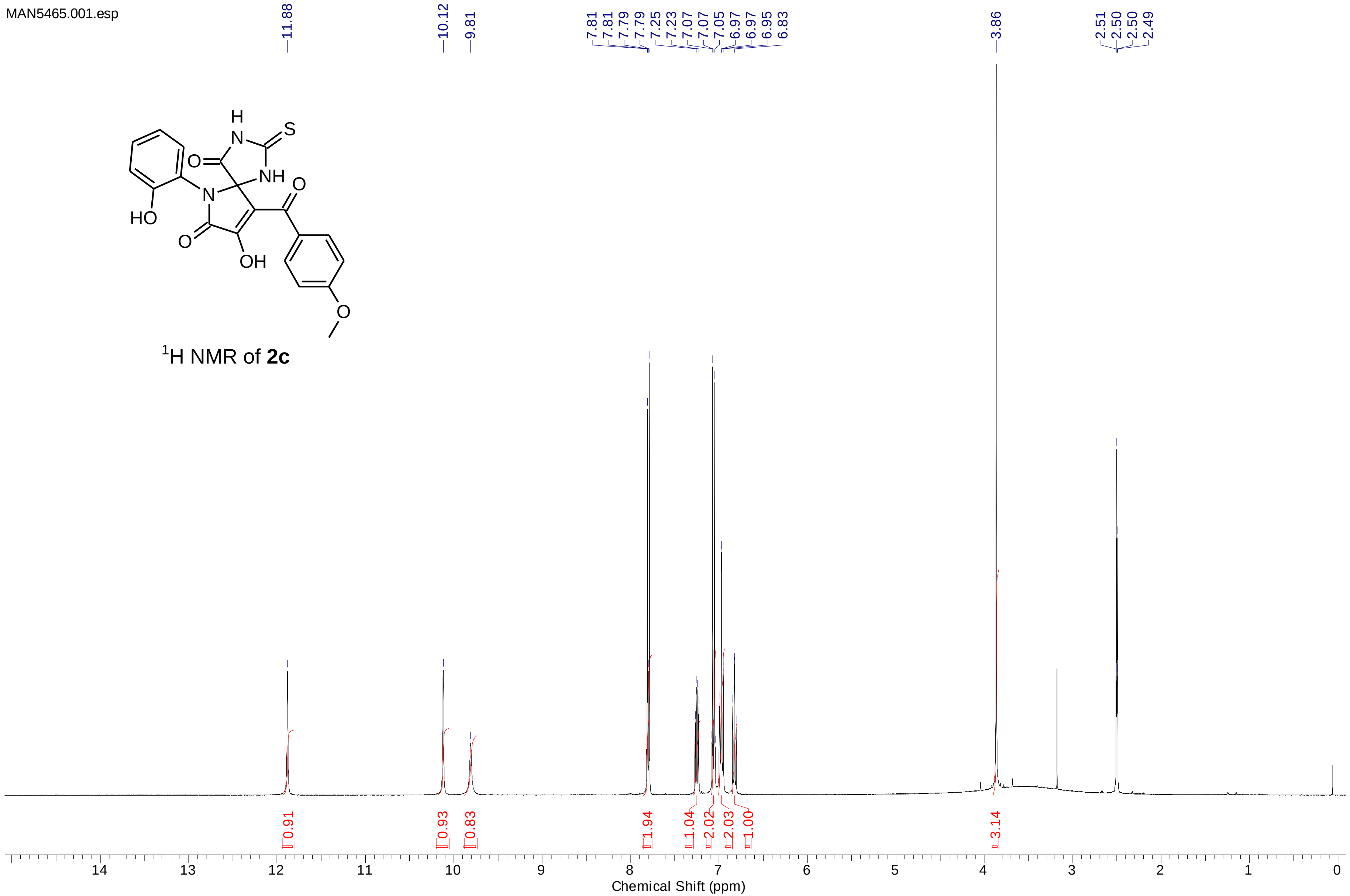
MAN5465.001.esp
11.88
10.12
9.81
7.81 7.81 7.79 7.79 7.25 7.23 7.07 7.07 7.05 6.97 6.97 6.95 6.83
3.86
2.51 2.50 2.50 2.49
H
N
S
O
NH
O
N
HO
O
OH
O
0.91
0.93
0.83
1.94
1.04
2.02
2.03
1.00
3.14
14 13 12 11 10 9 8 7 6 5 4 3 2 1 0
Chemical Shift (ppm)

^{1}H NMR of **2c**

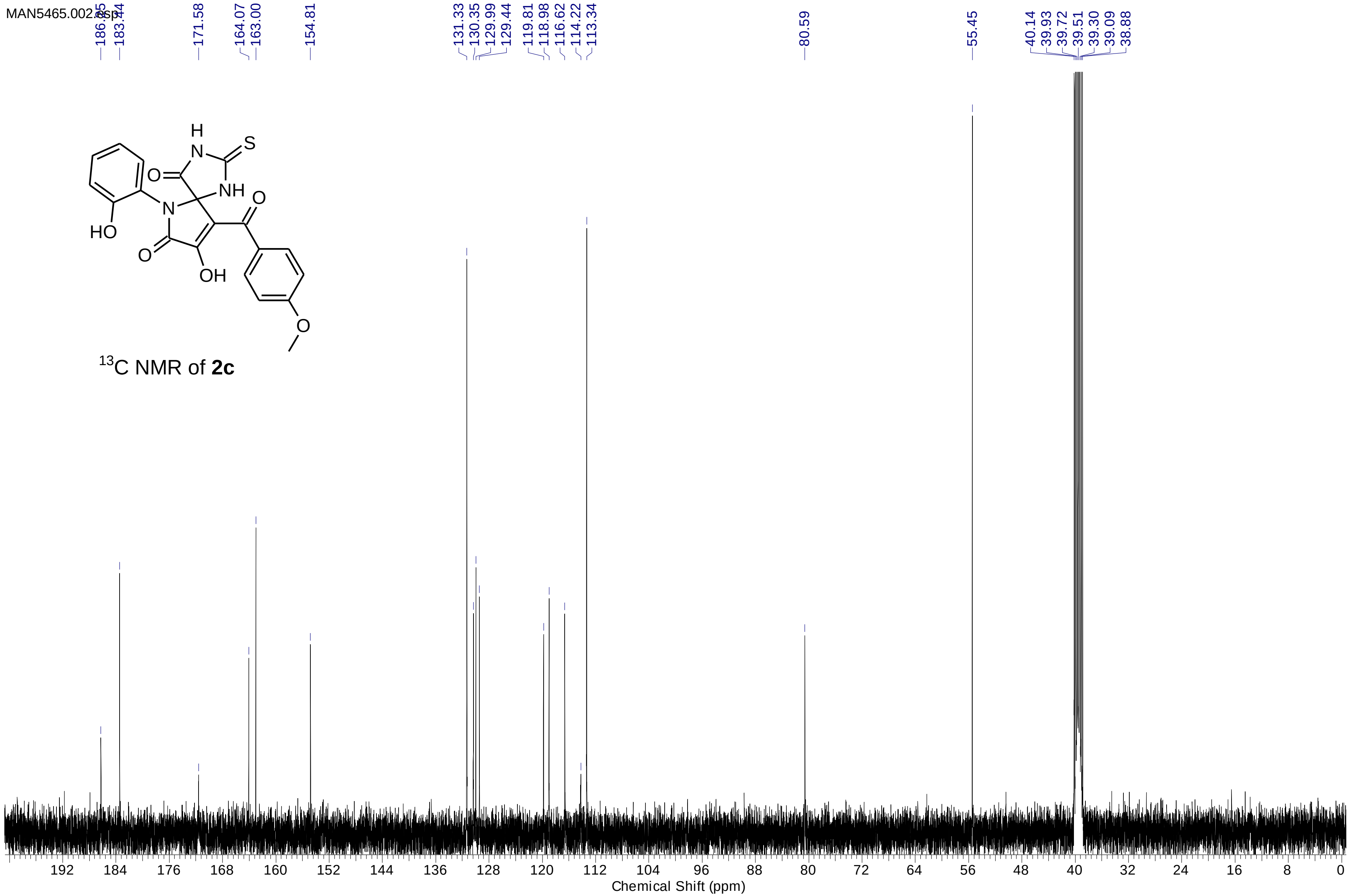
MAN5465.002.esp
186.55
183.74
171.58
164.07
163.00
154.81
131.33
130.35
129.99
129.44
119.81
118.98
116.62
114.22
113.34
80.59
55.45
40.14
39.93
39.72
39.51
39.30
39.09
38.88
Chemical Shift (ppm)

^{13}C NMR of **2c**

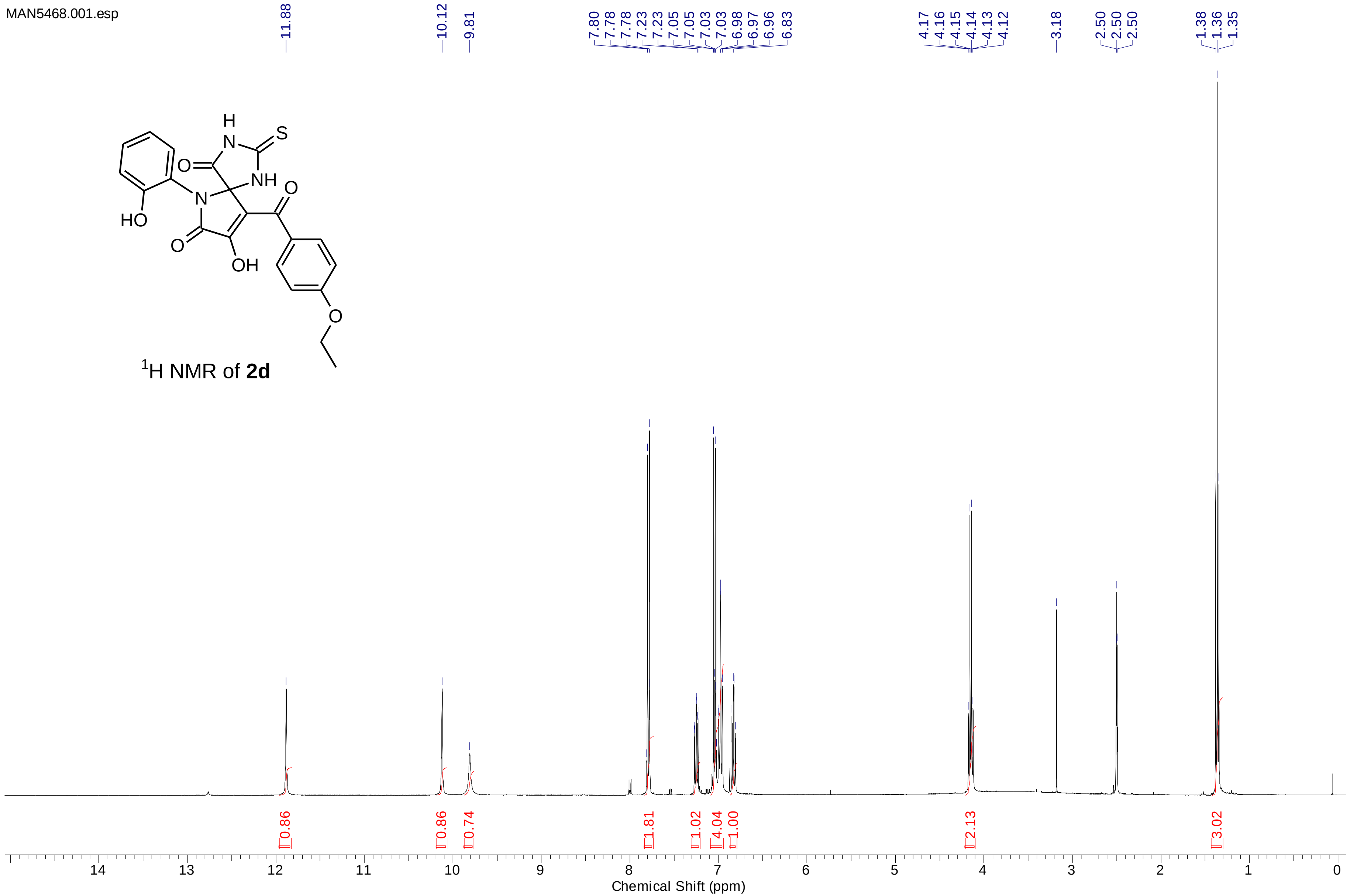
MAN5468.001.esp
11.88
10.12
9.81
7.80 7.78 7.78 7.23 7.23 7.05 7.05 7.03 7.03 6.98 6.97 6.96 6.83
4.17 4.16 4.15 4.14 4.13 4.12
3.18
2.50 2.50 2.50
1.38 1.36 1.35
0.86 0.86 0.74 1.81 1.02 4.04 1.00 2.13 3.02
Chemical Shift (ppm)

^{1}H NMR of **2d**

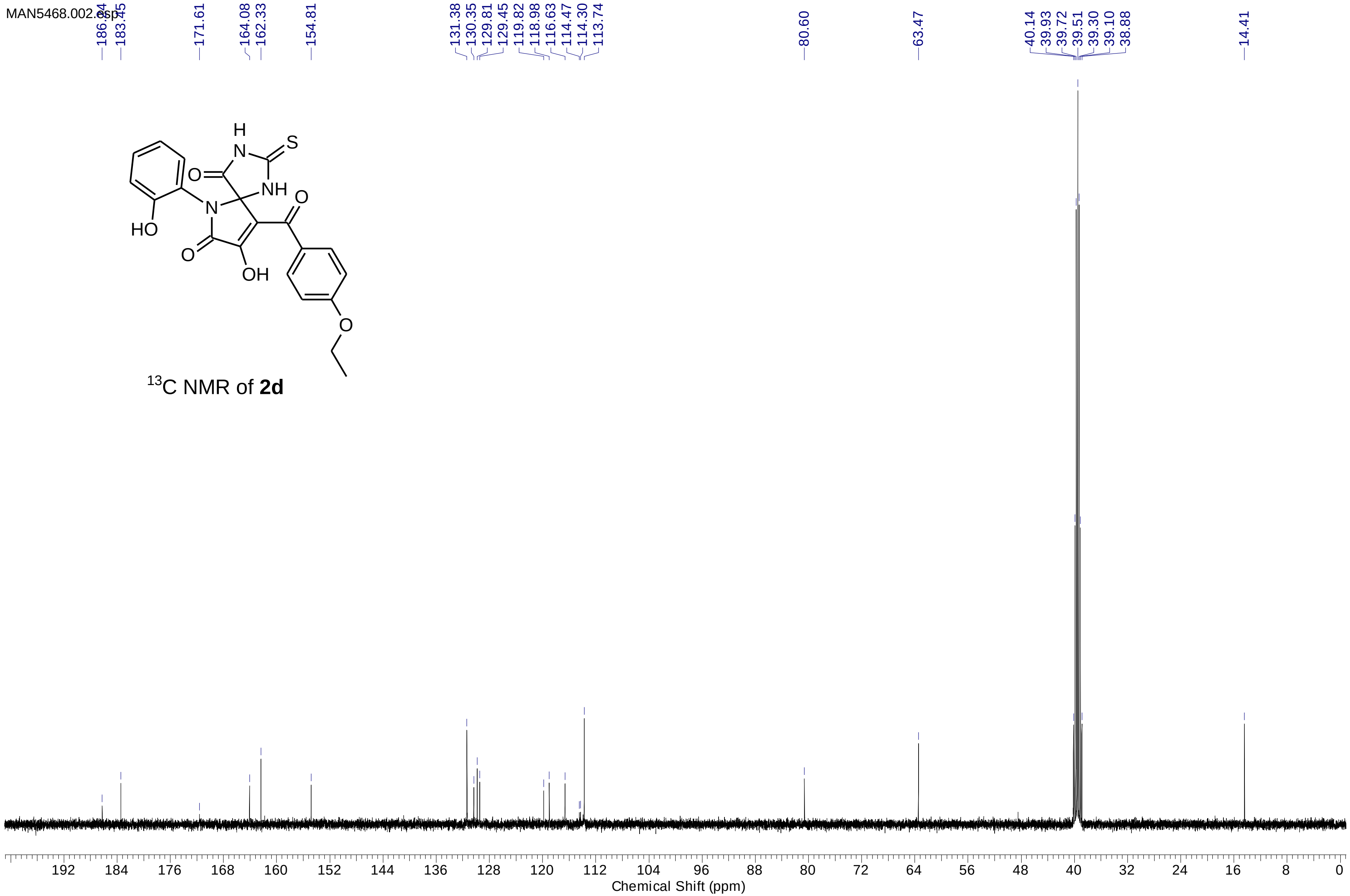
MAN5468.002.esp
186.24
183.45
171.61
164.08
162.33
154.81
131.38
130.35
129.81
129.45
119.82
118.98
116.63
114.47
114.30
113.74
80.60
63.47
40.14
39.93
39.72
39.51
39.30
39.10
38.88
14.41
Chemical Shift (ppm)

^{13}C NMR of **2d**

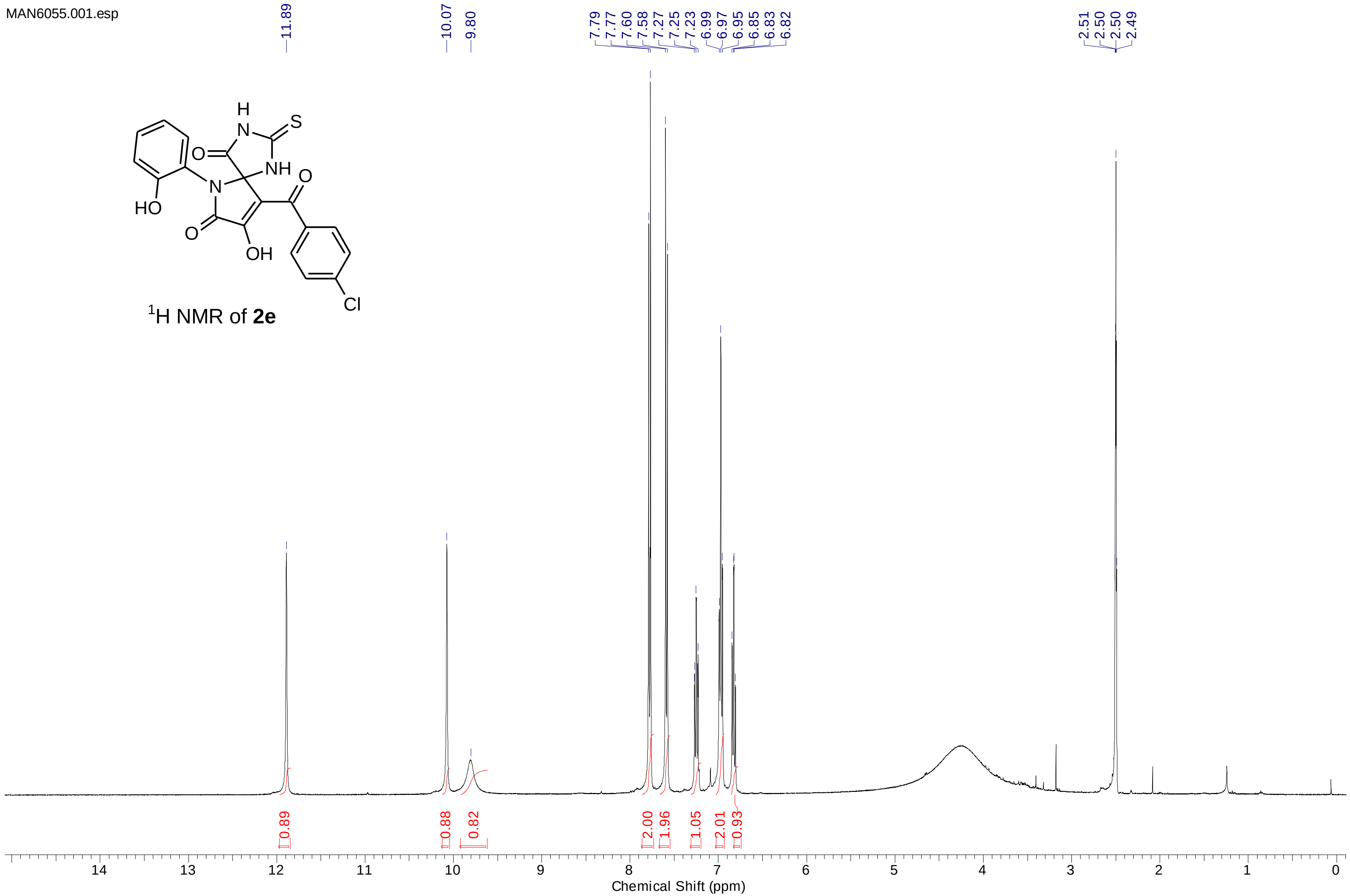
MAN6055.001.esp
11.89
10.07
9.80
7.79
7.77
7.60
7.58
7.27
7.25
7.23
6.99
6.97
6.95
6.85
6.83
6.82
2.51
2.50
2.50
2.49
0.89
0.88
0.82
2.00
1.96
1.05
2.01
0.93
14 13 12 11 10 9 8 7 6 5 4 3 2 1 0
Chemical Shift (ppm)
H
N
S
O
NH
O
N
HO
O
OH
Cl

¹H NMR of **2e**

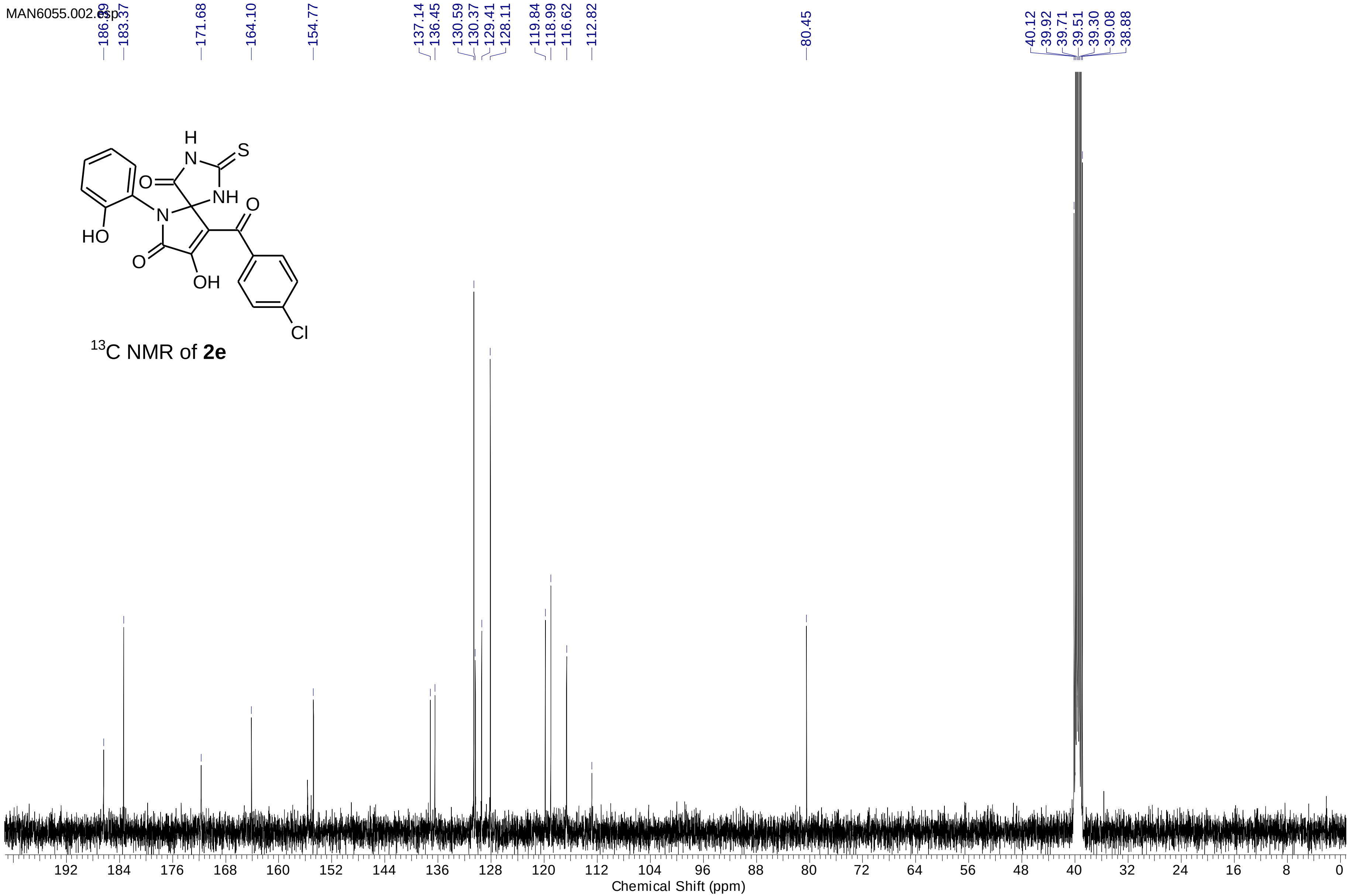
MAN6055.002.esp
183.37
171.68
164.10
154.77
137.14
136.45
130.59
130.37
129.41
128.11
119.84
118.99
116.62
112.82
80.45
40.12
39.92
39.71
39.51
39.30
39.08
38.88
H
N
S
O
NH
O
N
HO
O
OH
Cl
13C NMR of 2e
192
184
176
168
160
152
144
136
128
120
112
104
96
88
80
72
64
56
48
40
32
24
16
8
0
Chemical Shift (ppm)

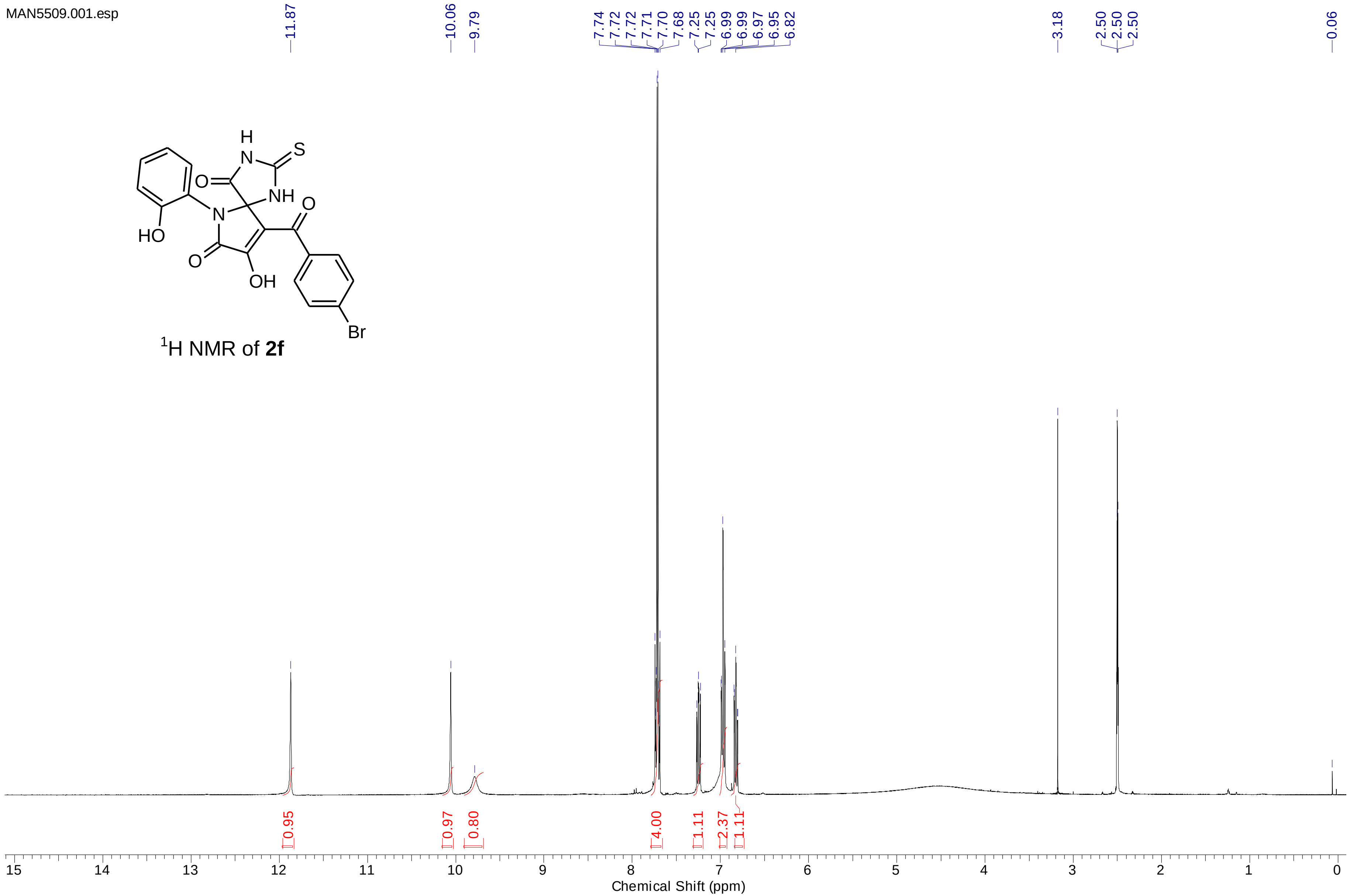
MAN5509.001.esp
11.87
10.06
9.79
7.74
7.72
7.72
7.71
7.70
7.68
7.25
7.25
6.99
6.99
6.97
6.95
6.82
3.18
2.50
2.50
2.50
0.06
0.95
0.97
0.80
4.00
1.11
2.37
1.11
Chemical Shift (ppm)
H
N
S
O
NH
O
N
HO
O
OH
Br

^{1}H NMR of **2f**

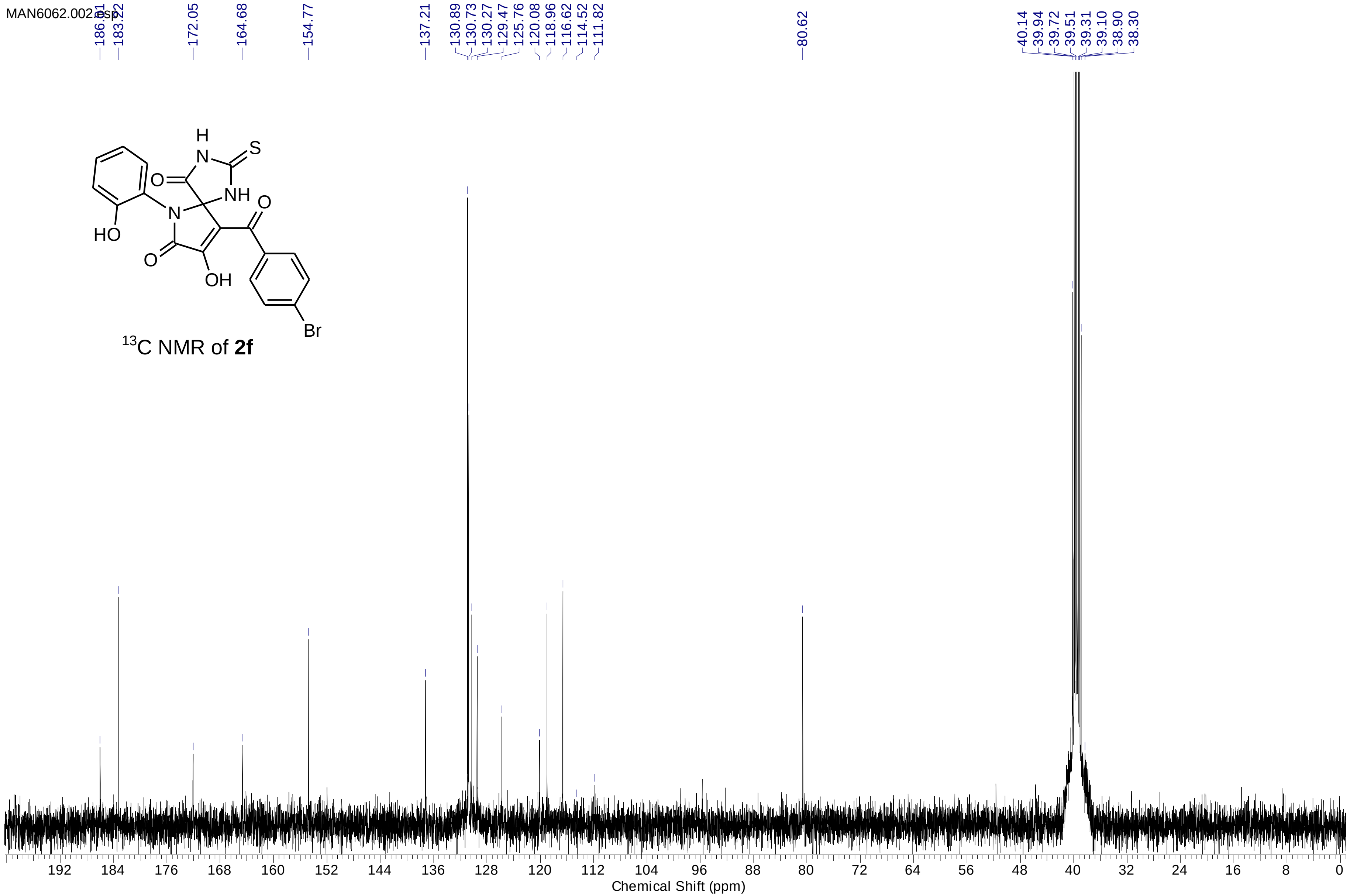
MAN6062.002.esp
186.91
183.22
172.05
164.68
154.77
137.21
130.89
130.73
130.27
129.47
125.76
120.08
118.96
116.62
114.52
111.82
80.62
40.14
39.94
39.72
39.51
39.31
39.10
38.90
38.30
H
N
S
O
NH
O
N
HO
O
OH
Br
Chemical Shift (ppm)

^{13}C NMR of **2f**

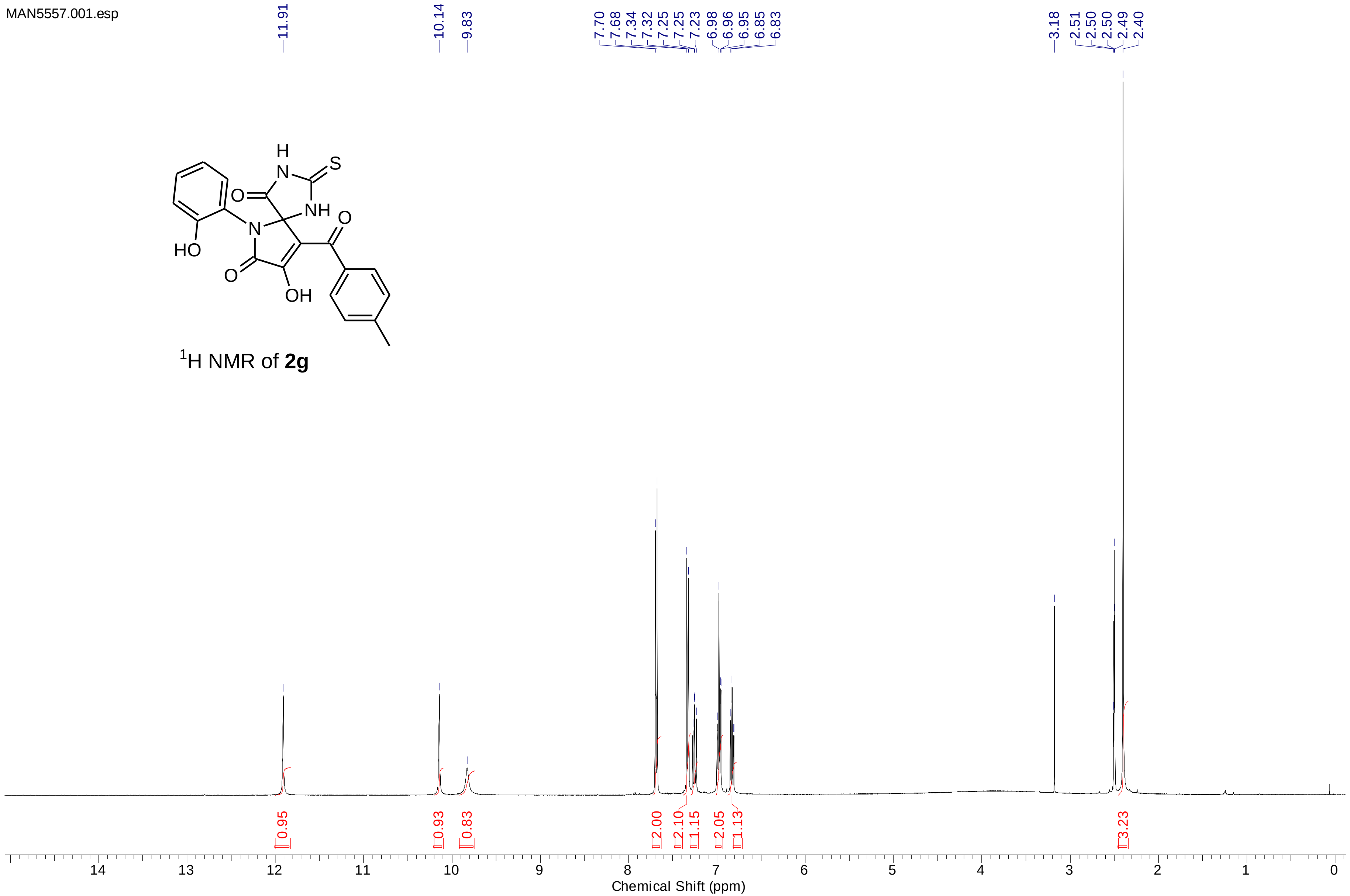
MAN5557.001.esp
11.91
10.14
9.83
7.70
7.68
7.34
7.32
7.25
7.25
7.23
6.98
6.96
6.95
6.85
6.83
3.18
2.51
2.50
2.50
2.49
2.40
H
N
S
O
NH
O
N
HO
O
OH
0.95
0.93
0.83
2.00
2.10
1.15
2.05
1.13
3.23
14
13
12
11
10
9
8
7
6
5
4
3
2
1
0
Chemical Shift (ppm)

^{1}H NMR of **2g**

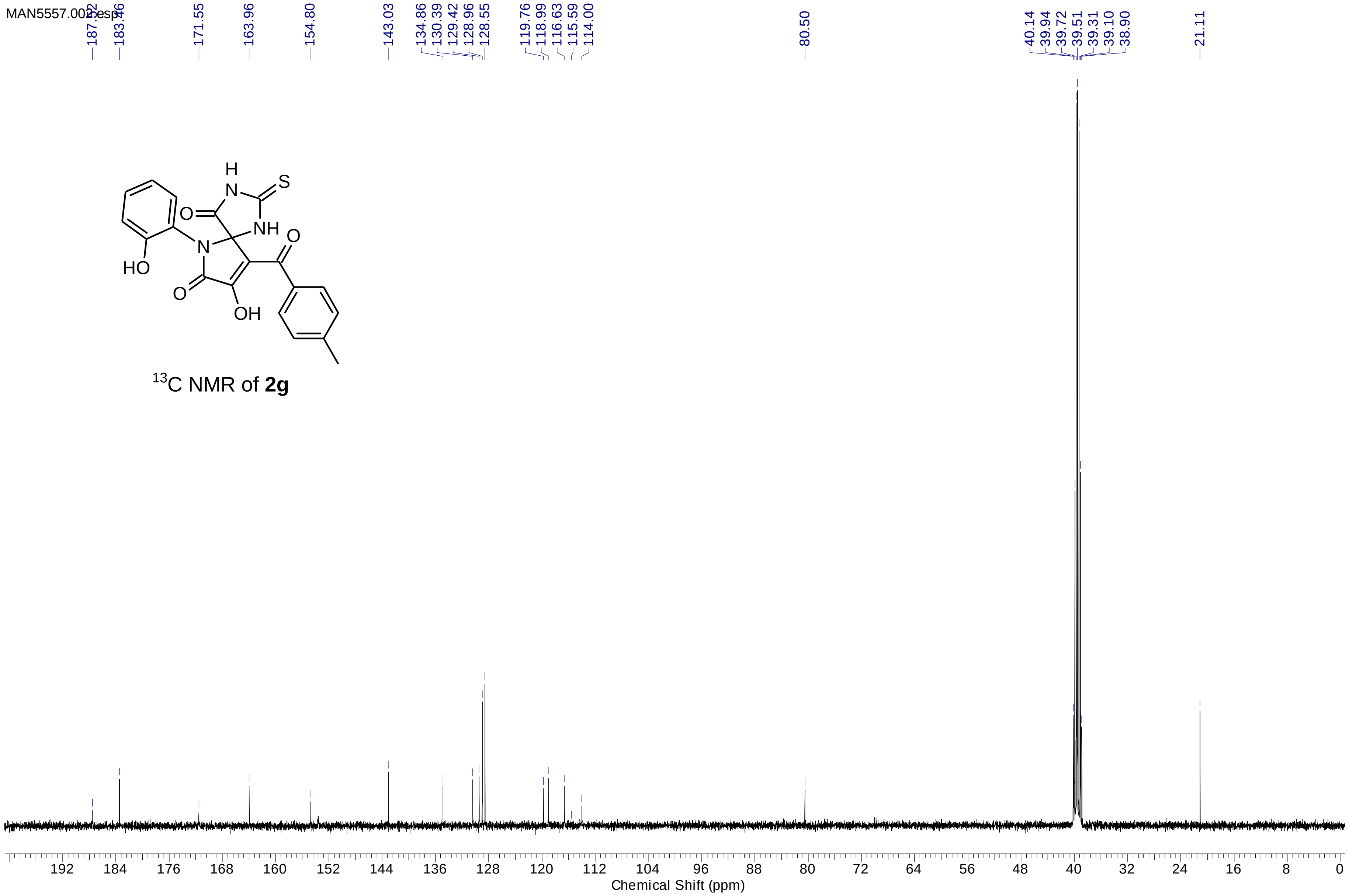
MAN5557.002.esp
187.52
183.46
171.55
163.96
154.80
143.03
134.86
130.39
129.42
128.96
128.55
119.76
118.99
116.63
115.59
114.00
80.50
40.14
39.94
39.72
39.51
39.31
39.10
38.90
21.11
H
N
S
O
NH
O
N
HO
O
OH
^{13}C NMR of **2g**
192 184 176 168 160 152 144 136 128 120 112 104 96 88 80 72 64 56 48 40 32 24 16 8 0
Chemical Shift (ppm)

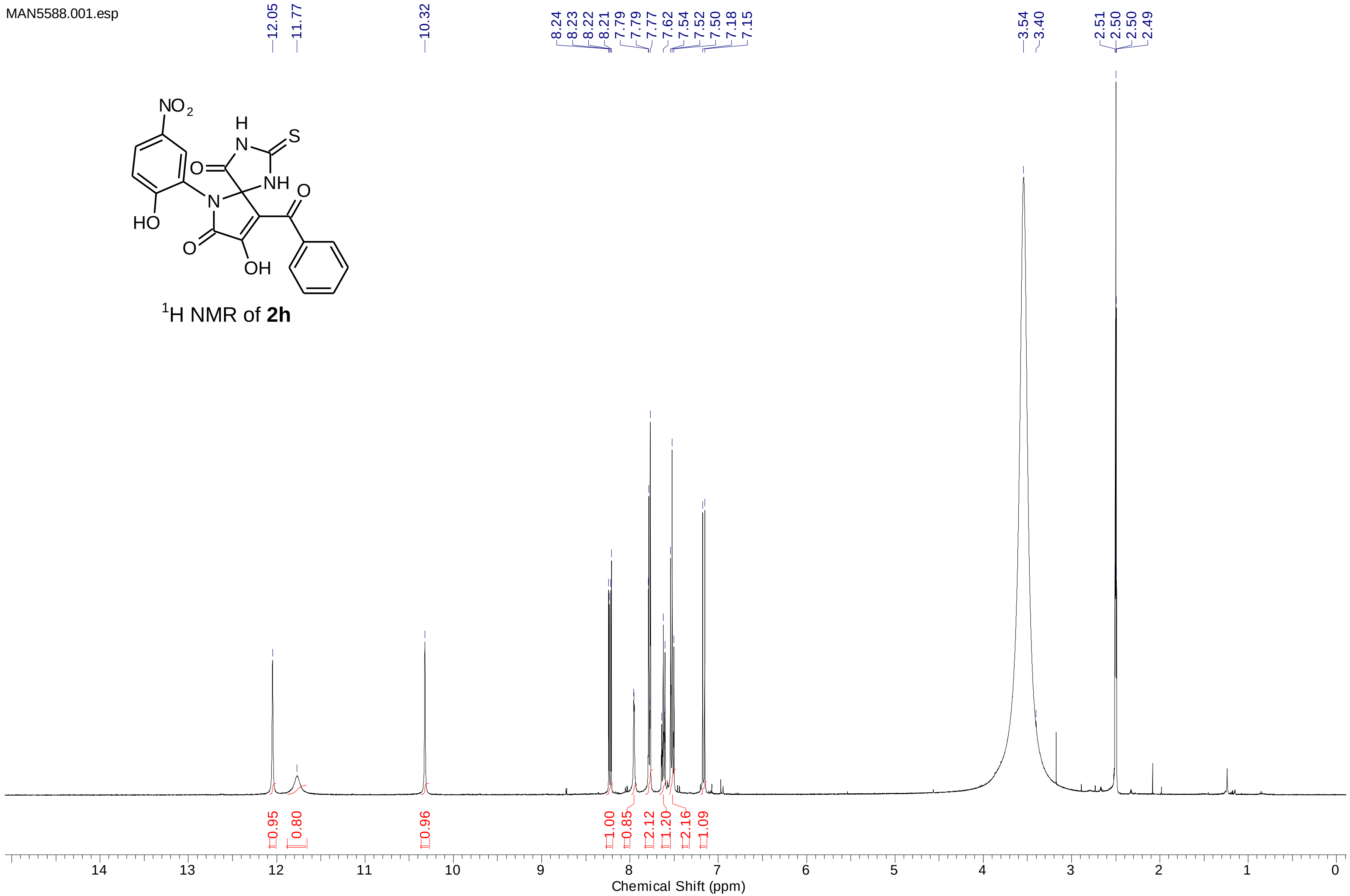
MAN5588.001.esp
12.05
11.77
10.32
8.24
8.23
8.22
8.21
7.79
7.79
7.77
7.62
7.54
7.52
7.50
7.18
7.15
3.54
3.40
2.51
2.50
2.50
2.49
NO2
H
N
S
O
NH
O
N
HO
O
OH
0.95
0.80
0.96
1.00
0.85
2.12
1.20
2.16
1.09
14
13
12
11
10
9
8
7
6
5
4
3
2
1
0
Chemical Shift (ppm)

^{1}H NMR of **2h**

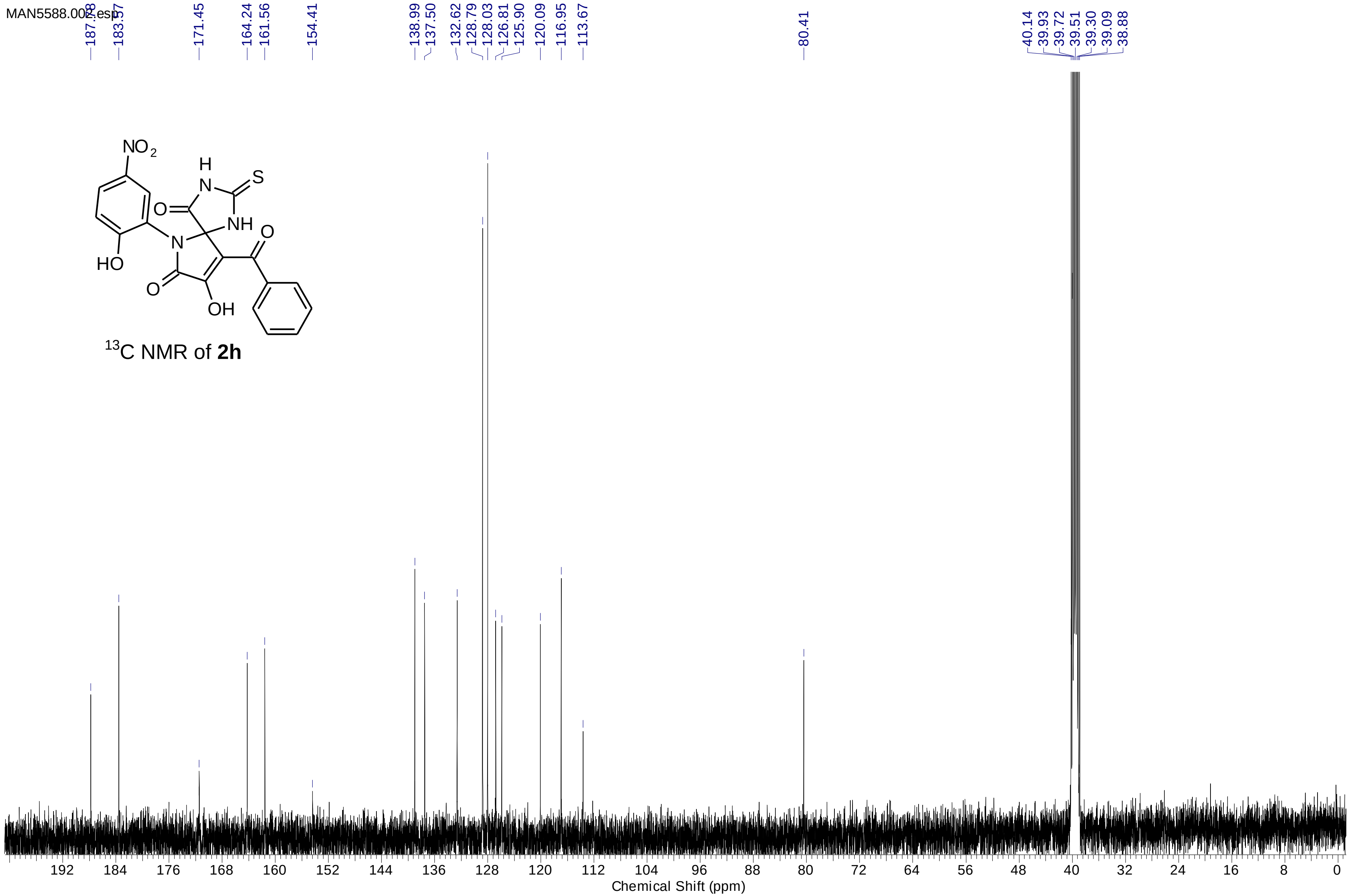
MAN5588.002.esp
183.97
171.45
164.24
161.56
154.41
138.99
137.50
132.62
128.79
128.03
126.81
125.90
120.09
116.95
113.67
80.41
40.14
39.93
39.72
39.51
39.30
39.09
38.88
NO_2
H
N
S
O
NH
O
N
HO
O
OH
192
184
176
168
160
152
144
136
128
120
112
104
96
88
80
72
64
56
48
40
32
24
16
8
0
Chemical Shift (ppm)

^{13}C NMR of **2h**

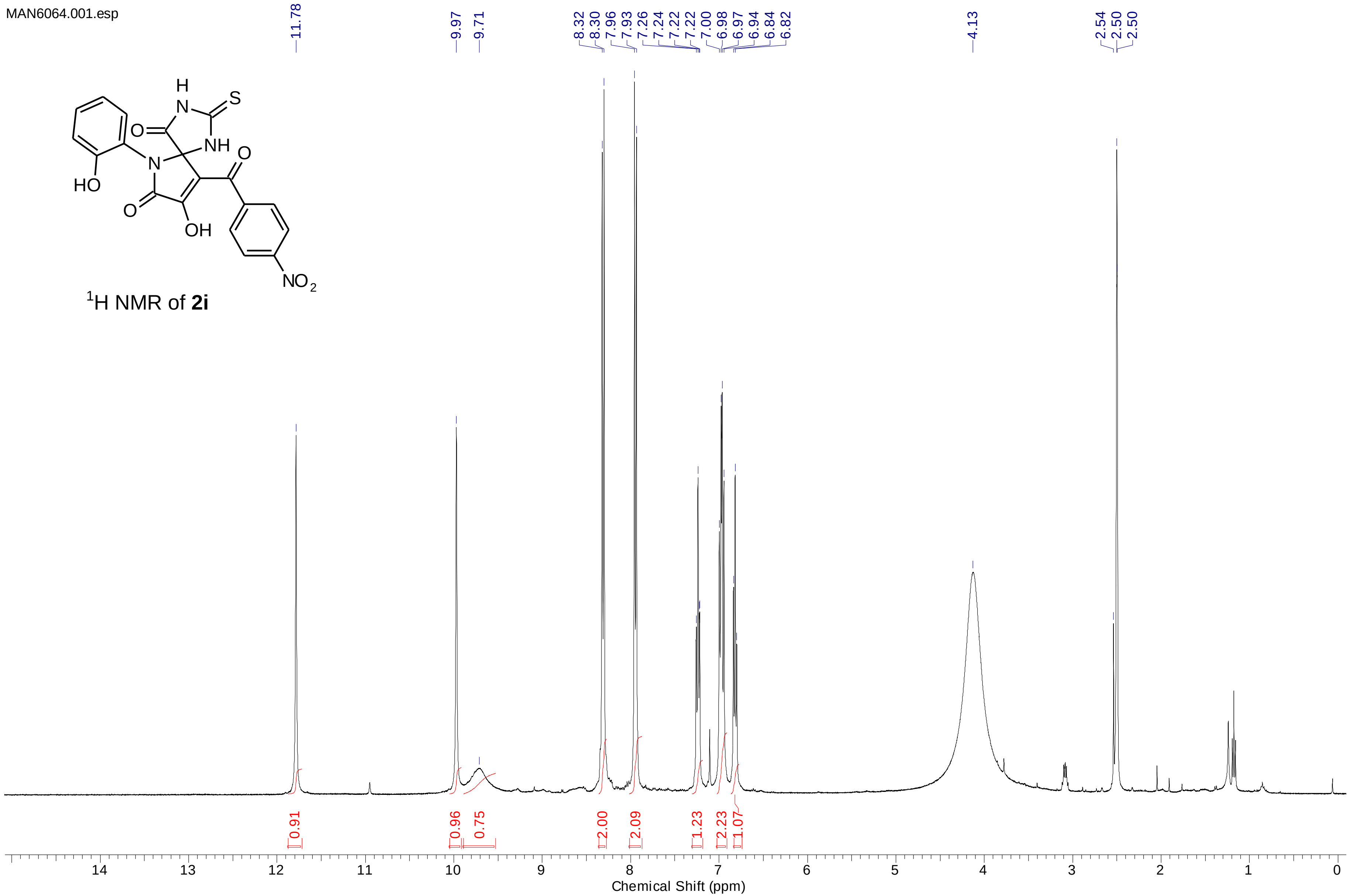

MAN6064.001.esp
11.78
9.97
9.71
8.32
8.30
7.96
7.93
7.26
7.24
7.22
7.22
7.00
6.98
6.97
6.94
6.84
6.82
4.13
2.54
2.50
2.50
0.91
0.96
0.75
2.00
2.09
1.23
2.23
1.07
Chemical Shift (ppm)

^{1}H NMR of **2i**

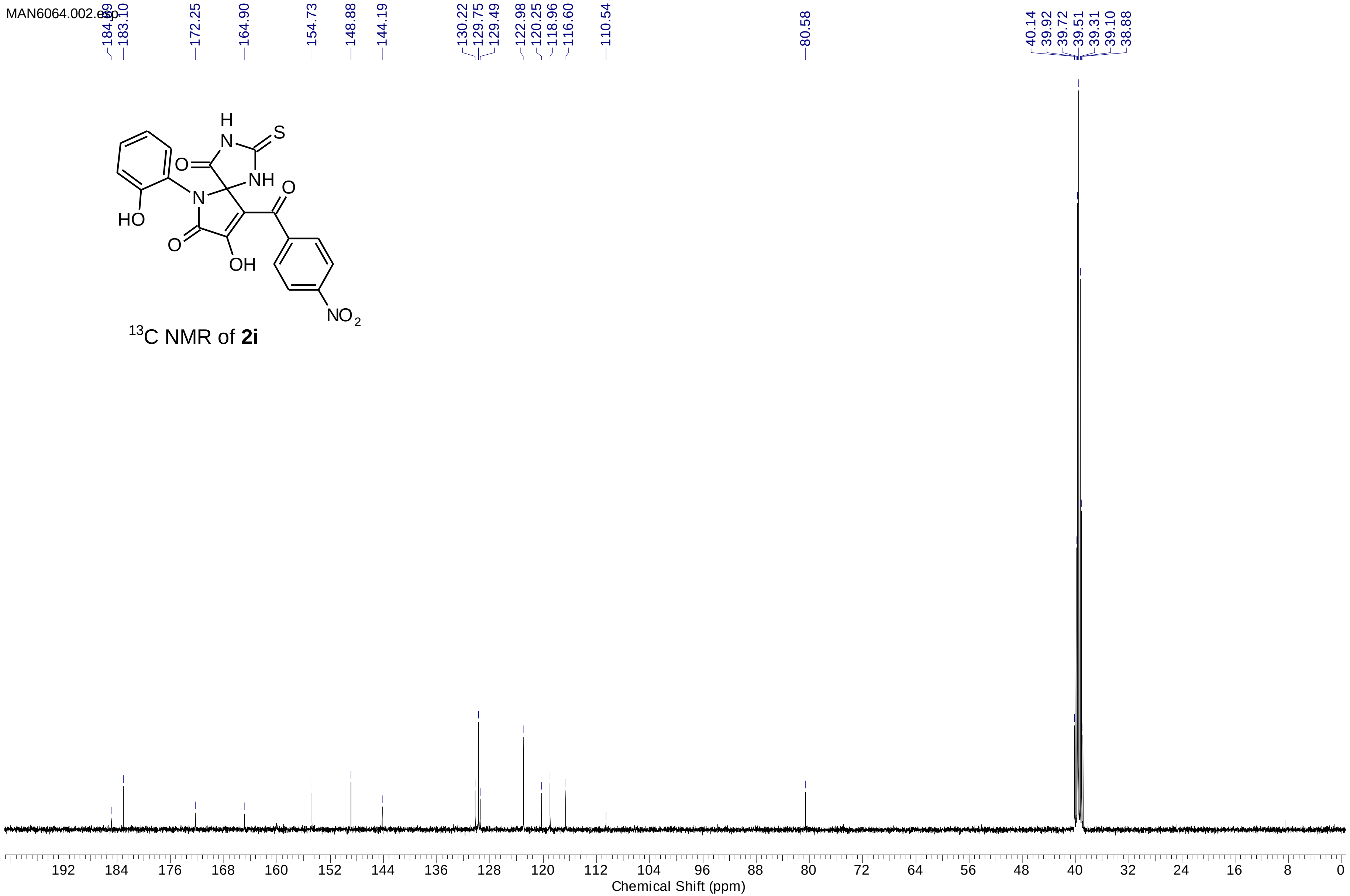
MAN6064.002.esp
184.89
183.10
172.25
164.90
154.73
148.88
144.19
130.22
129.75
129.49
122.98
120.25
118.96
116.60
110.54
80.58
40.14
39.92
39.72
39.51
39.31
39.10
38.88
H
N
S
O
NH
N
O
HO
O
OH
NO2
192 184 176 168 160 152 144 136 128 120 112 104 96 88 80 72 64 56 48 40 32 24 16 8 0
Chemical Shift (ppm)

^{13}C NMR of **2i**

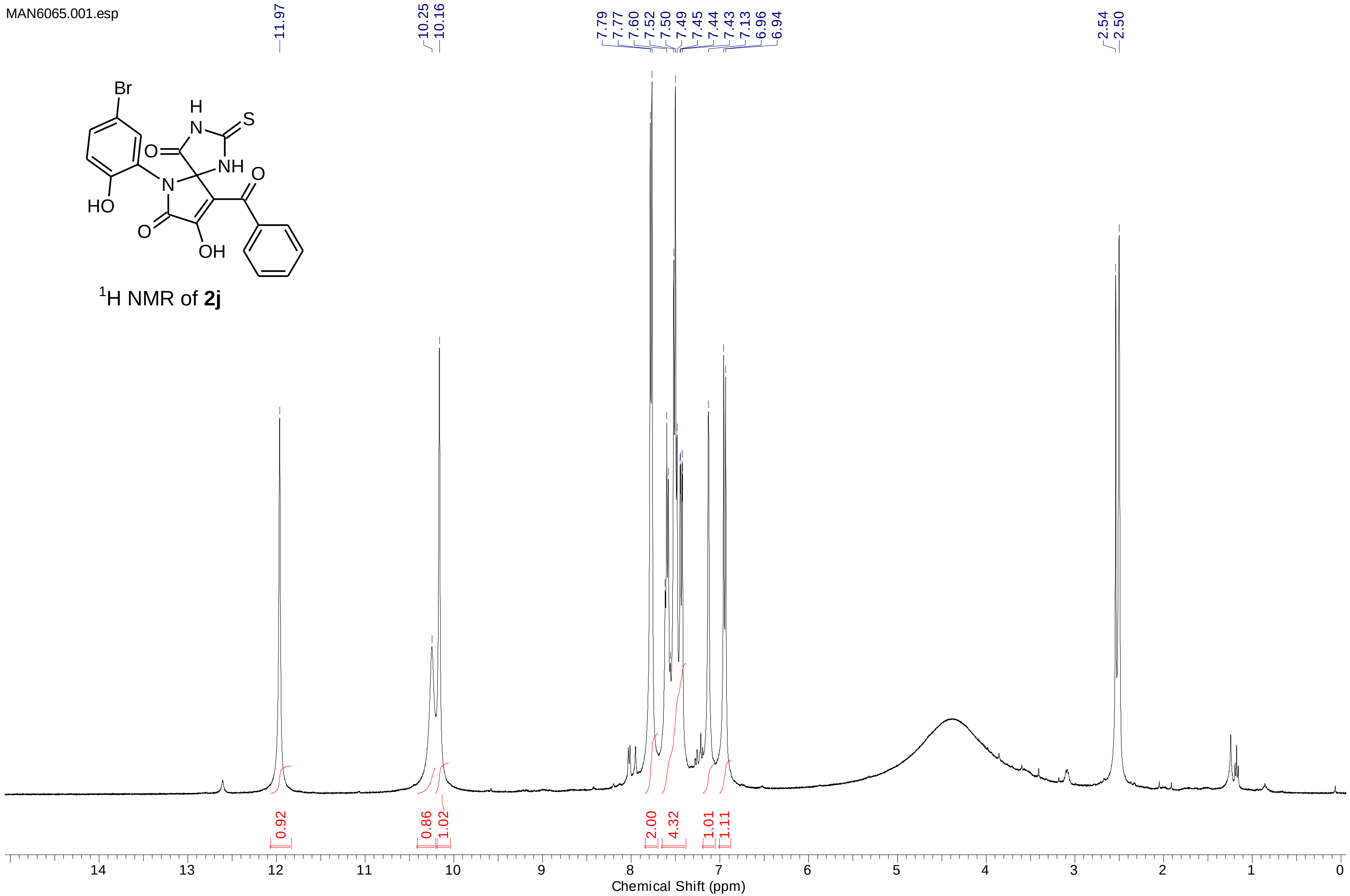
MAN6065.001.esp
11.97
10.25
10.16
7.79
7.77
7.60
7.52
7.50
7.49
7.45
7.44
7.43
7.13
6.96
6.94
2.54
2.50
Br
H
N
S
O
NH
O
N
HO
O
OH
¹H NMR of 2j
0.92
0.86
1.02
2.00
4.32
1.01
1.11
14
13
12
11
10
9
8
7
6
5
4
3
2
1
0
Chemical Shift (ppm)

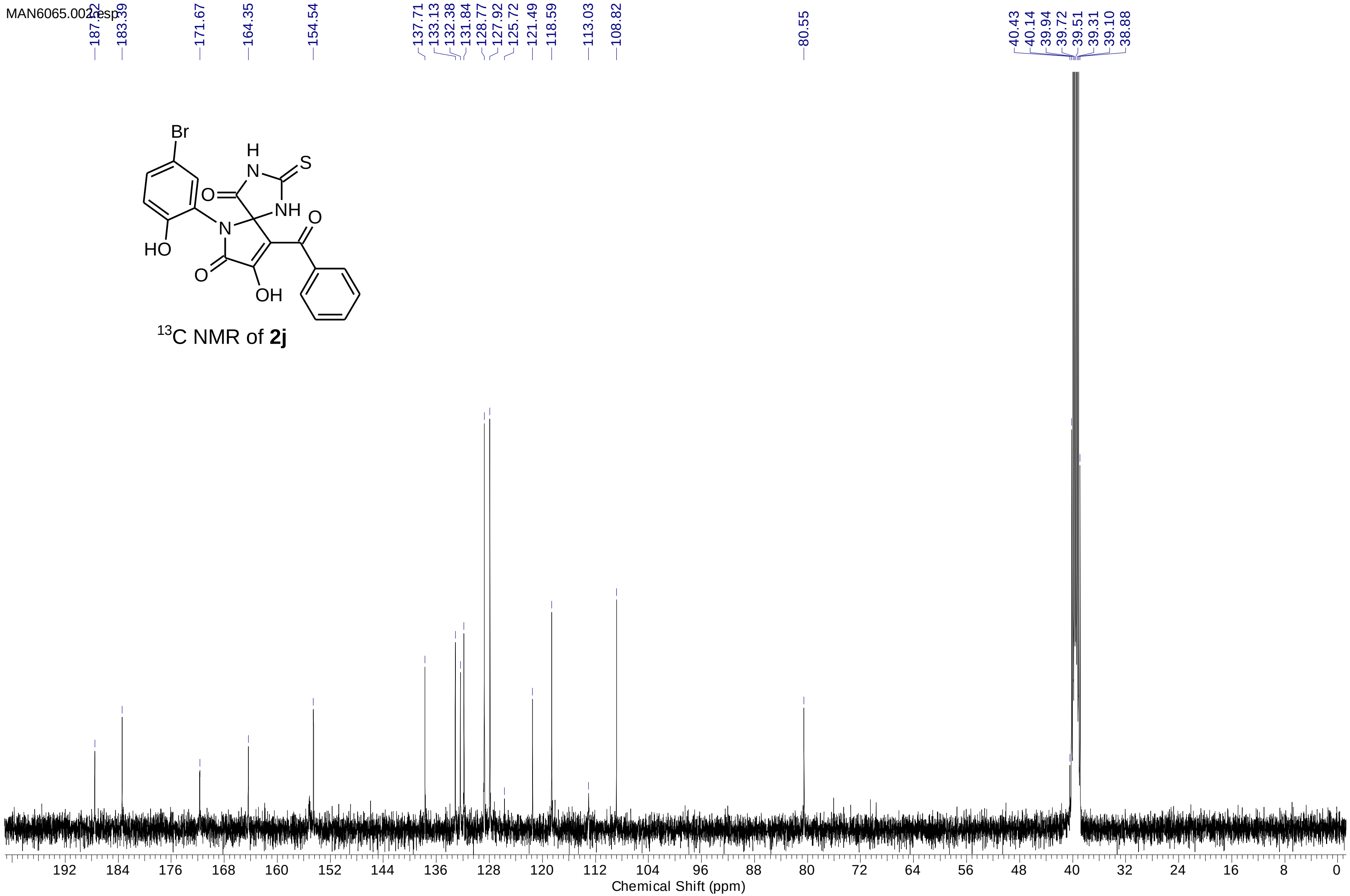
MAN6065.002.esp
187.22
183.39
171.67
164.35
154.54
137.71
133.13
132.38
131.84
128.77
127.92
125.72
121.49
118.59
113.03
108.82
80.55
40.43
40.14
39.94
39.72
39.51
39.31
39.10
38.88
Br
H
N
S
O
NH
O
N
HO
O
OH
Chemical Shift (ppm)

^{13}C NMR of **2j**

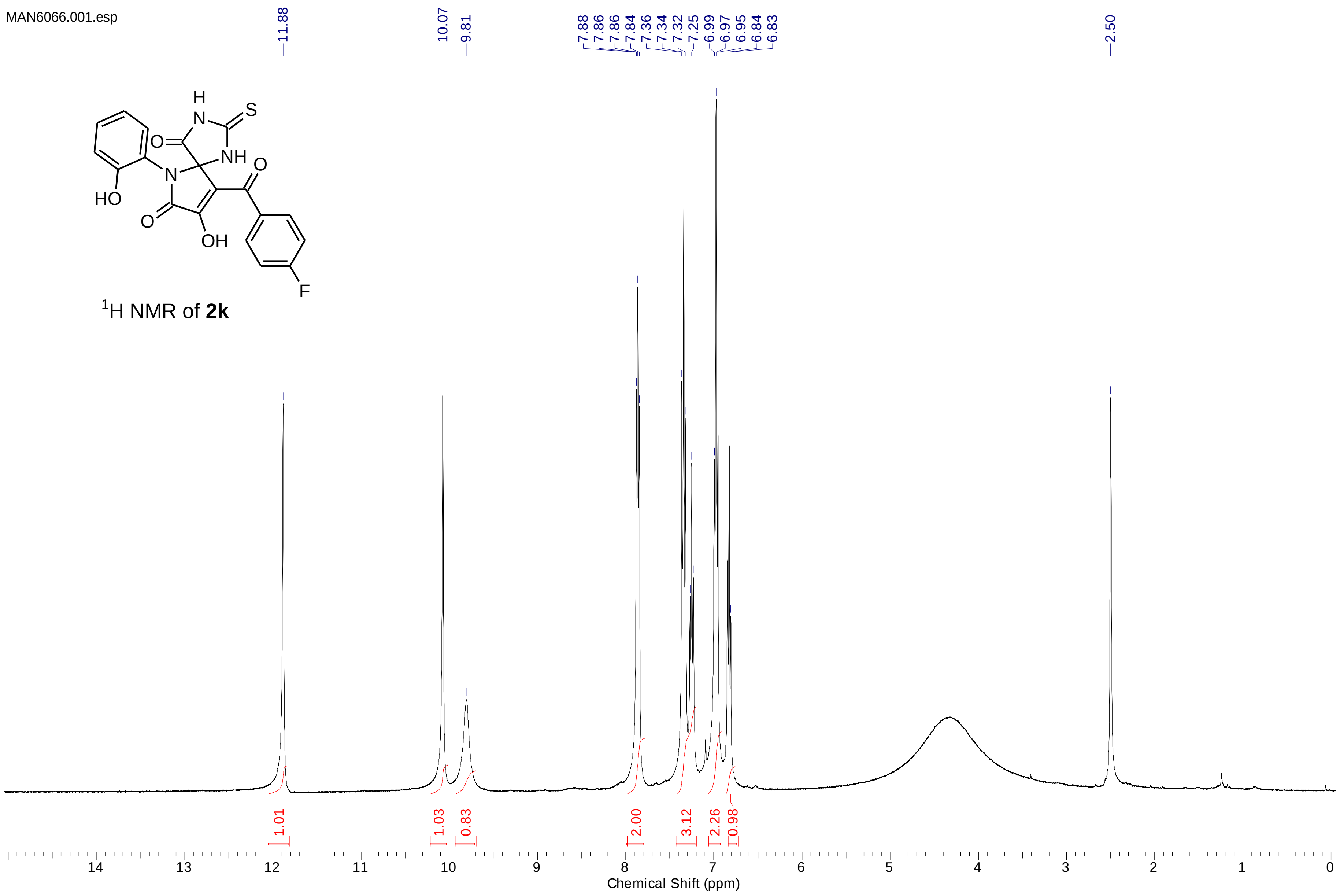
MAN6066.001.esp
11.88
10.07
9.81
7.88
7.86
7.86
7.84
7.36
7.34
7.32
7.25
6.99
6.97
6.95
6.84
6.83
2.50
H
N
S
O
NH
O
N
HO
O
OH
F
1H NMR of 2k
1.01
1.03
0.83
2.00
3.12
2.26
0.98
14
13
12
11
10
9
8
7
6
5
4
3
2
1
0
Chemical Shift (ppm)

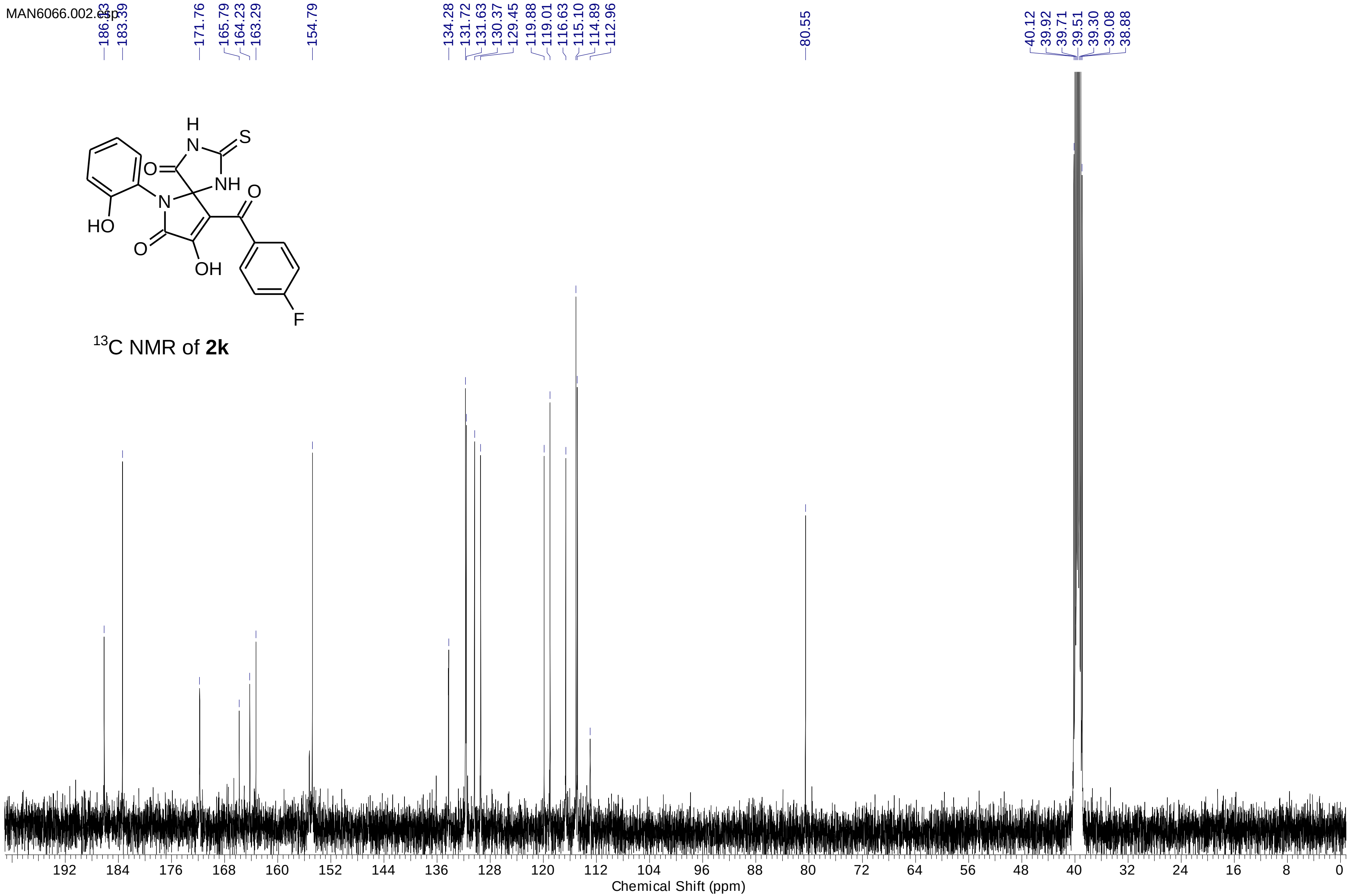
MAN6066.002.esp
186.33
183.39
171.76
165.79
164.23
163.29
154.79
134.28
131.72
131.63
130.37
129.45
119.88
119.01
116.63
115.10
114.89
112.96
80.55
40.12
39.92
39.71
39.51
39.30
39.08
38.88
H
N
S
O
NH
N
O
HO
O
OH
F
192 184 176 168 160 152 144 136 128 120 112 104 96 88 80 72 64 56 48 40 32 24 16 8 0
Chemical Shift (ppm)

^{13}C NMR of **2k**

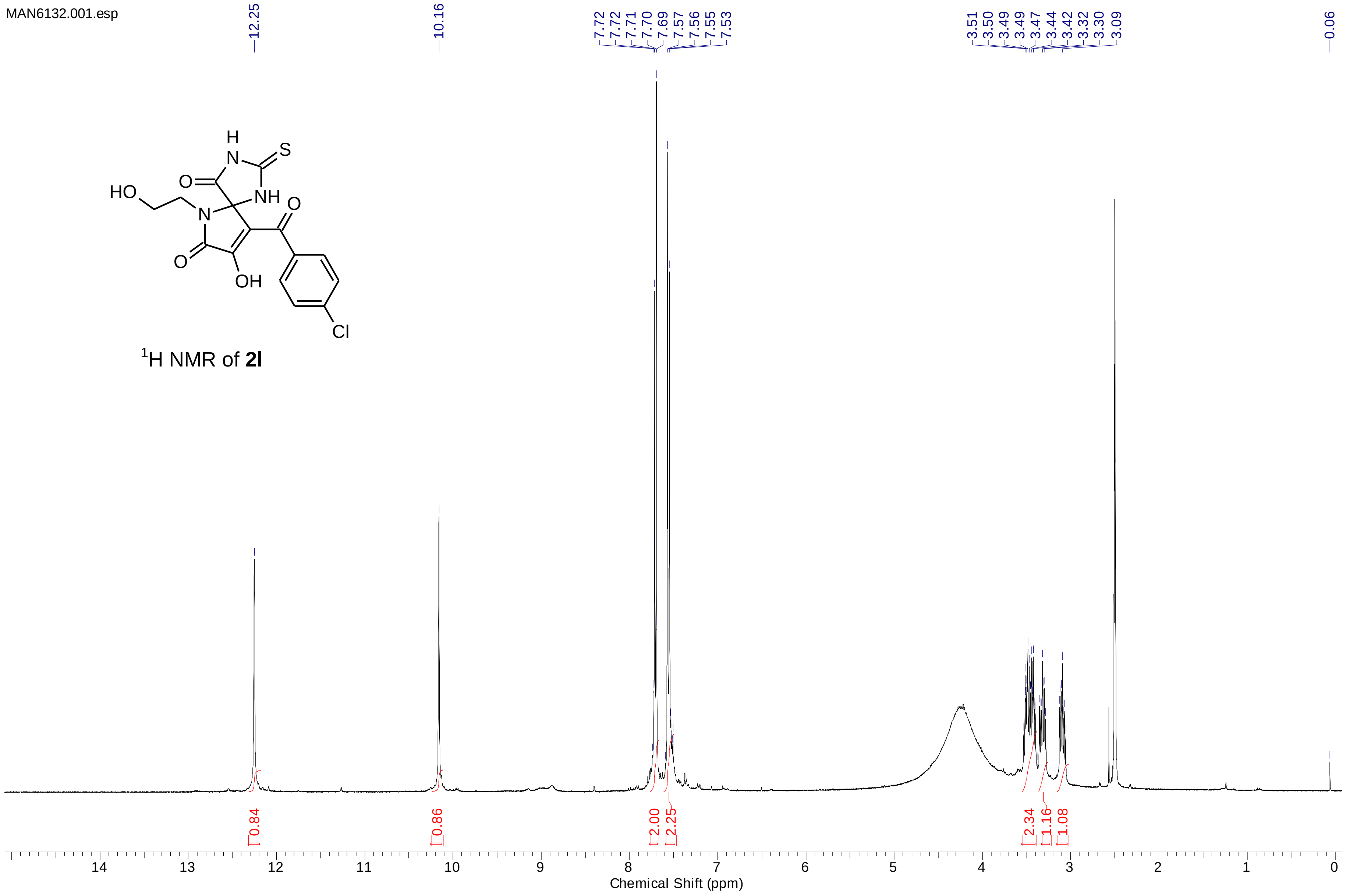
MAN6132.001.esp
12.25
10.16
7.72
7.72
7.71
7.70
7.69
7.57
7.56
7.55
7.53
3.51
3.50
3.49
3.49
3.47
3.44
3.42
3.32
3.30
3.09
0.06
H
N
S
O
HO
NH
O
N
O
OH
Cl
¹H NMR of 2l
0.84
0.86
2.00
2.25
2.34
1.16
1.08
14
13
12
11
10
9
8
7
6
5
4
3
2
1
0
Chemical Shift (ppm)

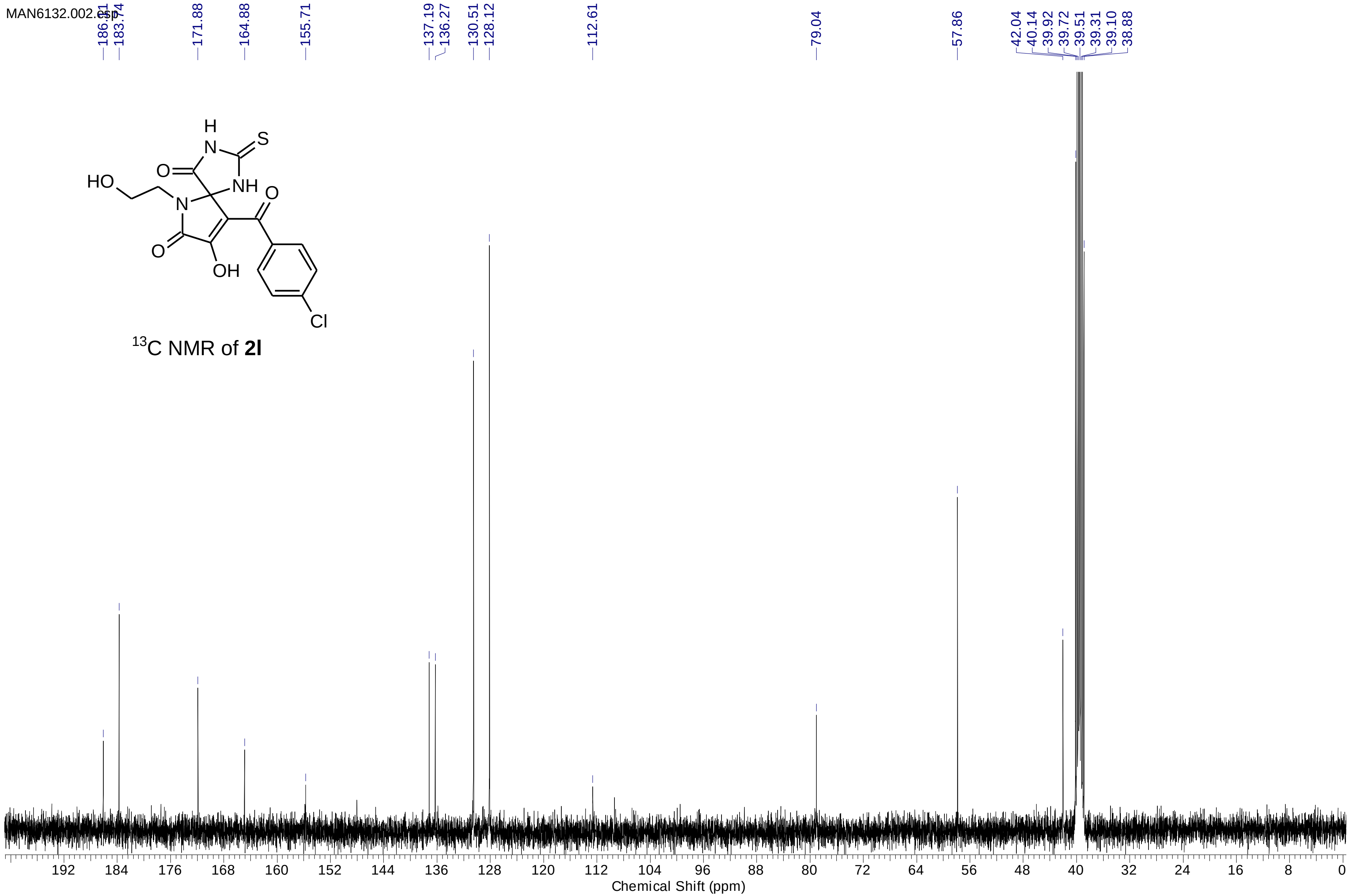
MAN6132.002.esp
186.11
183.74
171.88
164.88
155.71
137.19
136.27
130.51
128.12
112.61
79.04
57.86
42.04
40.14
39.92
39.72
39.51
39.31
39.10
38.88
H
N
S
O
HO
NH
O
N
O
OH
Cl
192
184
176
168
160
152
144
136
128
120
112
104
96
88
80
72
64
56
48
40
32
24
16
8
0
Chemical Shift (ppm)

^{13}C NMR of **2l**

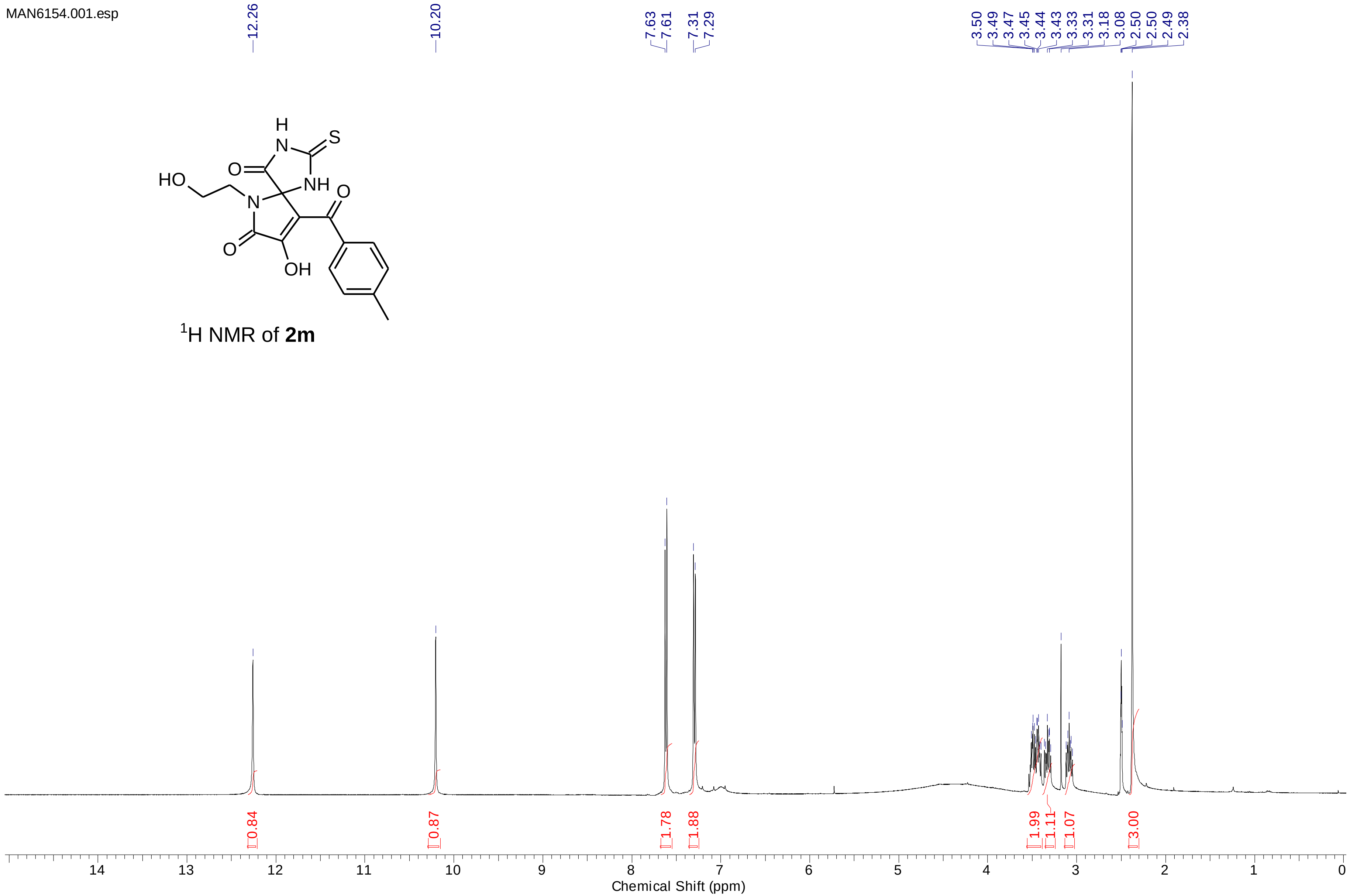
MAN6154.001.esp
12.26
10.20
7.63
7.61
7.31
7.29
3.50
3.49
3.47
3.45
3.44
3.43
3.33
3.31
3.18
3.08
2.50
2.50
2.49
2.38
HO
O
H
N
S
NH
O
N
O
OH
0.84
0.87
1.78
1.88
1.99
1.11
1.07
3.00
14
13
12
11
10
9
8
7
6
5
4
3
2
1
0
Chemical Shift (ppm)

^{1}H NMR of **2m**

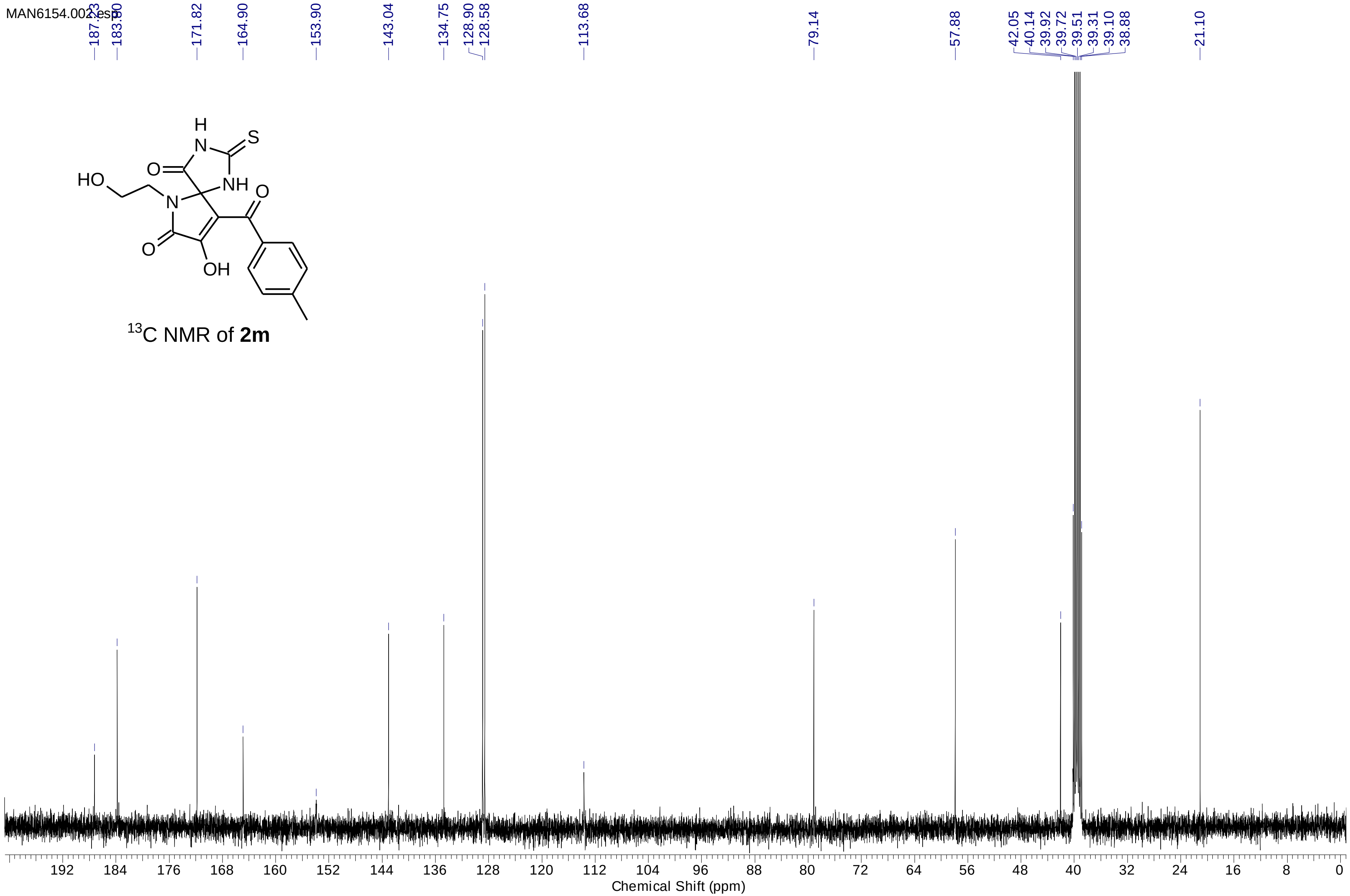
MAN6154.002.esp
187.23
183.80
171.82
164.90
153.90
143.04
134.75
128.90
128.58
113.68
79.14
57.88
42.05
40.14
39.92
39.72
39.51
39.31
39.10
38.88
21.10
H
N
S
O
NH
HO
N
O
O
OH
192
184
176
168
160
152
144
136
128
120
112
104
96
88
80
72
64
56
48
40
32
24
16
8
0
Chemical Shift (ppm)

^{13}C NMR of **2m**

Pseudothiohydantoins 3a–m; General procedure

Thiourea (1.5 mmol, 115 mg) was added to a suspension of FPD **1a**–**m** (1.5 mmol) in anhydrous ethyl acetate (10 mL). The mixture was stirred at room temperature for 12 h. The formed solid was filtered off and recrystallized from methanol to afford the desired pseudothiohydantoin **3a–m** (if the product still contains impurities after the recrystallization, it can be purified by stirring it in ethyl acetate (5 mL) for 12–24h, and the subsequent filtration of the solid gives the desired pseudothiohydantoin).

2-Amino-9-benzoyl-8-hydroxy-6-(2-hydroxyphenyl)-1-thia-3,6-diazaspiro[4.4]nona-2,8-diene-4,7-dione (3a)

Yield: 468 mg (79%); white solid; mp 199–200 °C (decomp.).

^{1}H NMR (400 MHz, DMSO-d_6): δ = 9.54 (s, 1 H), 9.07 (br.s, 1 H), 8.80 (s, 1 H), 7.75 (m, 2 H), 7.61 (m, 1 H), 7.50 (m, 2 H), 7.25 (m, 1 H), 6.94 (m, 2 H), 6.80 (m, 1 H).

^{13}C NMR (100 MHz, DMSO-d_6): δ = 187.8, 183.8, 179.6, 164.9, 154.7, 138.0, 132.4, 130.3, 129.2, 128.6 (2 C), 128.1 (2 C), 120.6, 119.1, 116.7 (2 C), 85.2.

IR (mineral oil): 3291, 1704, 1658 cm^{-1}.

MS (ESI+): m/z calcd for $C_{19}H_{13}N_3O_5S+H^+$: 396.07 [M+H$^+$]; found: 395.98.

Anal. Calcd (%) for $C_{19}H_{13}N_3O_5S$: C 57.72; H 3.31; N 10.63. Found: C 57.92; H 3.43; N 10.51.

2-Amino-9-benzoyl-6-(4-chloro-2-hydroxyphenyl)-8-hydroxy-1-thia-3,6-diazaspiro[4.4]nona-2,8-diene-4,7-dione (3b)

Yield: 560 mg (87%); white solid; mp 182–184 °C (decomp.).

^{1}H NMR (400 MHz, DMSO-d_6): δ = 9.93 (s, 1 H), 9.19 (br.s, 1 H), 8.90 (s, 1 H), 7.76 (m, 2 H), 7.61 (m, 1 H), 7.51 (m, 2 H), 7.33 (m, 1 H), 6.96 (m, 2 H).

^{13}C NMR (100 MHz, DMSO-d_6): δ = 187.8, 183.5, 179.5, 164.8, 154.1, 137.9, 132.5, 130.3, 128.6 (3 C), 128.1 (2 C), 121.8, 121.6, 118.3, 116.7, 85.0.

IR (mineral oil): 3283, 3092, 1714, 1639 cm^{-1}.

MS (ESI+): m/z calcd for $C_{19}H_{12}ClN_3O_5S+H^+$: 430.03 [M+H$^+$]; found: 430.09.

Anal. Calcd (%) for $C_{19}H_{12}ClN_3O_5S$: C 53.09; H 2.81; N 9.78. Found: C 52.99; H 2.81; N 9.89.

2-Amino-8-hydroxy-6-(2-hydroxyphenyl)-9-(4-methoxybenzoyl)-1-thia-3,6-diazaspiro[4.4]nona-2,8-diene-4,7-dione (3c)

Yield: 581 (91%); white solid; mp 191–193 °C (decomp.).

^{1}H NMR (400 MHz, DMSO-d_6): δ = 9.53 (s, 1 H), 9.04 (br.s, 1 H), 8.77 (s, 1 H), 7.78 (m, 2 H), 7.25 (m, 1 H), 7.04 (m, 2 H), 6.94 (m, 2 H), 6.80 (m, 1 H), 3.86 (s, 3 H).
^{13}C NMR (100 MHz, DMSO-d_6): δ = 186.4, 183.7, 179.5, 165.0, 163.0, 154.8, 131.2 (2 C), 130.5, 130.3, 129.2, 120.7, 119.1, 117.4, 116.7, 113.5 (2 C), 85.3, 55.5.
IR (mineral oil): 3318, 3207, 1698, 1668 cm^{-1}.
MS (ESI+): m/z calcd for $C_{20}H_{15}N_3O_6S+H^+$: 426.08 [M+H^+]; found: 426.14.
Anal. Calcd (%) for $C_{20}H_{15}N_3O_6S$: C 56.47; H 3.55; N 9.88. Found: C 56.21; H 3.65; N 9.91.

2-Amino-9-(4-ethoxybenzoyl)-8-hydroxy-6-(2-hydroxyphenyl)-1-thia-3,6-diazaspiro[4.4]nona-2,8-diene-4,7-dione (3d)
Yield: 520 mg (79%); white solid; mp 185–186 °C (decomp.).
^{1}H NMR (400 MHz, DMSO-d_6): δ = 9.51 (s, 1 H), 9.03 (br.s, 1 H), 8.77 (s, 1 H), 7.76 (m, 2 H), 7.25 (m, 1 H), 7.03 (m, 2 H), 6.94 (m, 2 H), 6.80 (m, 1 H), 4.15 (q, *J* = 7.1 Hz, 2 H), 1.36 (t, *J* = 7.1 Hz, 3 H).
^{13}C NMR (100 MHz, DMSO-d_6): δ = 186.5, 183.6, 179.4, 164.9, 162.3, 154.8, 131.2 (2 C), 130.3, 130.2, 129.2, 120.6, 119.1, 117.6, 116.7, 113.9 (2 C), 85.3, 63.5, 14.4.
IR (mineral oil): 3288, 3072, 1716, 1705, 1681, 1650 cm^{-1}.
MS (ESI+): m/z calcd for $C_{21}H_{17}N_3O_6S+H^+$: 440.09 [M+H^+]; found: 440.14.
Anal. Calcd (%) for $C_{21}H_{17}N_3O_6S$: C 57.40; H 3.90; N 9.56. Found: C 57.28; H 4.05; N 9.59.

2-Amino-9-(4-chlorobenzoyl)-8-hydroxy-6-(2-hydroxyphenyl)-1-thia-3,6-diazaspiro[4.4]nona-2,8-diene-4,7-dione (3e)
Yield: 393 (61%); white solid; mp 195–198 °C (decomp.).
^{1}H NMR (400 MHz, DMSO-d_6): δ = 9.53 (s, 1 H), 9.09 (br.s, 1 H), 8.83 (s, 1 H), 7.76 (m, 2 H), 7.58 (m, 2 H), 7.26 (m, 1 H), 6.94 (m, 2 H), 6.80 (m, 1 H).
^{13}C NMR (100 MHz, DMSO-d_6): δ = 186.6, 183.6, 179.6, 164.7, 154.7, 137.3, 136.7, 130.5 (2 C), 130.4, 129.1, 128.3 (2 C), 120.5, 119.2, 116.7, 116.4, 85.1.
IR (mineral oil): 3250, 3074, 1711, 1695, 1661 cm^{-1}.
MS (ESI+): m/z calcd for $C_{19}H_{12}ClN_3O_5S+H^+$: 430.03 [M+$H^+$]; found: 430.13.
Anal. Calcd (%) for $C_{19}H_{12}ClN_3O_5S$: C 53.09; H 2.81; N 9.78. Found: C 53.00; H 2.83; N 9.86.

2-Amino-9-(4-bromobenzoyl)-8-hydroxy-6-(2-hydroxyphenyl)-1-thia-3,6-diazaspiro[4.4]nona-2,8-diene-4,7-dione (3f)
Yield: 434 mg (61%); white solid; mp 188–190 °C (decomp.).
^{1}H NMR (400 MHz, DMSO-d_6): δ = 9.51 (s, 1 H), 9.10 (br.s, 1 H), 8.82 (s, 1 H), 7.70 (m, 4 H), 7.25 (m, 1 H), 6.94 (m, 2 H), 6.80 (m, 1 H).
^{13}C NMR (100 MHz, DMSO-d_6): δ = 186.7, 183.6, 179.5, 164.7, 154.7, 137.1, 131.2 (2 C), 130.8, 130.6 (2 C), 130.3, 129.1, 126.3, 120.5, 119.1, 116.7, 85.1.
IR (mineral oil): 3081, 1714, 1644 cm^{-1}.
MS (ESI+): m/z calcd for $C_{19}H_{12}BrN_3O_5S+H^+$: 473.98, 475.97 [M+H$^+$]; found: 474.04, 476.05.
Anal. Calcd (%) for $C_{19}H_{12}BrN_3O_5S$: C 48.12; H 2.55; N 8.86. Found: C 48.31; H 2.57; N 8.88.

2-Amino-8-hydroxy-6-(2-hydroxyphenyl)-9-(4-methylbenzoyl)-1-thia-3,6-diazaspiro[4.4]nona-2,8-diene-4,7-dione (3g)
Yield: 546 mg (89%); white solid; mp 192–194 °C (decomp.).
^{1}H NMR (400 MHz, DMSO-d_6): δ = 9.52 (s, 1 H), 9.05 (br.s, 1 H), 8.79 (s, 1 H), 7.68 (m, 2 H), 7.32 (m, 2 H), 7.26 (m, 1 H), 6.95 (m, 2 H), 6.80 (m, 1 H), 2.39 (s, 3 H).
^{13}C NMR (100 MHz, DMSO-d_6): δ = 187.6, 183.6, 179.5, 164.9, 154.8, 143.0, 135.3, 130.3, 129.2, 128.9 (2 C), 128.7 (2 C), 120.6, 119.1, 117.2, 116.7, 85.2, 21.1.
IR (mineral oil): 3280, 1709, 1695, 1650 cm^{-1}.
MS (ESI+): m/z calcd for $C_{20}H_{15}N_3O_5S+H^+$: 410.08 [M+H$^+$]; found: 410.14.
Anal. Calcd (%) for $C_{20}H_{15}N_3O_5S{\cdot}CH_3OH$: C 57.14; H 4.34; N 9.52. Found: C 57.24; H 4.52; N 9.42.

2-Amino-9-benzoyl-8-hydroxy-6-(2-hydroxy-5-nitrophenyl)-1-thia-3,6-diazaspiro[4.4]nona-2,8-diene-4,7-dione (3h)
Yield: 383 mg (58%); white solid; mp 215–218 °C (decomp.).
^{1}H NMR (400 MHz, DMSO-d_6): δ = 11.44 (br.s, 1 H), 9.24 (br.s, 1 H), 8.93 (s, 1 H), 8.22 (m, 1 H), 7.90 (m, 1 H), 7.77 (m, 2 H), 7.62 (m, 1 H), 7.52 (m, 2 H), 7.16 (m, 1 H).
^{13}C NMR (100 MHz, DMSO-d_6): δ = 187.8, 183.4, 179.3, 165.0, 161.6, 152.9, 139.2, 137.8, 132.6, 128.7 (2 C), 128.1 (2 C), 126.8, 125.7, 120.8, 117.3, 85.0.
IR (mineral oil): 3175, 1748, 1690, 1666 cm^{-1}.
MS (ESI+): m/z calcd for $C_{19}H_{12}N_4O_7S+H^+$: 441.05 [M+H$^+$]; found: 441.18.

Anal. Calcd (%) for $C_{19}H_{12}N_4O_7S$: C 51.82; H 2.75; N 12.72. Found: C 52.06; H 2.71; N 12.75.

2-Amino-8-hydroxy-6-(2-hydroxyphenyl)-9-(4-nitrobenzoyl)-1-thia-3,6-diazaspiro[4.4]nona-2,8-diene-4,7-dione (3i)

Yield: 350 mg (53%); white solid; mp 204–206 °C (decomp.).

^{1}H NMR (400 MHz, DMSO-d_6): δ = 9.57 (br.s, 1 H), 9.19 (br.s, 1 H), 8.93 (br.s, 1 H), 8.33 (m, 2 H), 7.92 (m, 2 H), 7.26 (m, 1 H), 6.96 (m, 2 H), 6.81 (m, 1 H).

^{13}C NMR (100 MHz, DMSO-d_6): δ = 186.1, 183.3, 179.5, 164.6, 154.7, 149.3, 143.6, 130.4, 129.7 (2 C), 129.1, 123.3 (2 C), 120.5, 119.1, 116.7, 115.6, 84.9.

IR (mineral oil): 3335, 3206, 1753, 1715, 1669 cm^{-1}.

MS (ESI+): m/z calcd for $C_{19}H_{12}N_4O_7S+H^+$: 441.05 [M+H$^+$]; found: 441.01.

Anal. Calcd (%) for $10C_{19}H_{12}N_4O_7S{\cdot}CH_3OH$: C 51.72; H 2.82; N 12.63. Found: C 51.99; H 2.75; N 12.60.

2-Amino-9-benzoyl-6-(5-bromo-2-hydroxyphenyl)-8-hydroxy-1-thia-3,6-diazaspiro[4.4]nona-2,8-diene-4,7-dione (3j)

Yield: 419 mg (59%); white solid; mp 197–198 °C (decomp.).

^{1}H NMR (400 MHz, DMSO-d_6): δ = 9.96 (s, 1 H), 9.18 (br.s, 1 H), 8.89 (s, 1 H), 7.75 (m, 2 H), 7.61 (m, 1 H), 7.50 (m, 2 H), 7.44 (m, 1 H), 7.07 (m, 1 H), 6.92 (m, 1 H).

^{13}C NMR (100 MHz, DMSO-d_6): δ = 187.8, 183.6, 179.5, 164.9, 154.6, 137.9, 133.2, 132.5, 131.5, 128.6 (2 C), 128.1 (2 C), 122.1, 118.8, 116.8, 109.0, 85.1.

IR (mineral oil): кох 3322, 3203, 1708, 1681, 1646 cm^{-1}.

MS (ESI+): m/z calcd for $C_{19}H_{12}BrN_3O_5S+H^+$: 473.98, 475.97 [M+H$^+$]; found: 474.08, 476.07.

Anal. Calcd (%) for $10C_{19}H_{12}BrN_3O_5S{\cdot}CH_3OH$: C 48.05; H 2.62; N 8.80. Found: C 47.89; H 2.68; N 8.83.

2-Amino-9-(4-fluorobenzoyl)-8-hydroxy-6-(2-hydroxyphenyl)-1-thia-3,6-diazaspiro[4.4]nona-2,8-diene-4,7-dione (3k)

Yield: 508 mg (82%); white solid; mp 188–190 °C (decomp.).

^{1}H NMR (400 MHz, DMSO-d_6): δ = 9.50 (s, 1 H), 9.05 (br.s, 1 H), 8.79 (s, 1 H), 7.84 (m, 2 H), 7.32 (m, 2 H), 7.24 (m, 1 H), 6.94 (m, 2 H), 6.80 (m, 1 H).

^{13}C NMR (100 MHz, DMSO-d_6): δ = 186.2, 183.8, 179.5, 165.8, 164.9, 163.3, 154.7, 134.7, 134.6, 131.6, 131.5, 130.3, 129.1, 120.7, 119.1, 116.7, 115.2, 115.0, 85.3.

IR (mineral oil): 3257, 1704, 1659 cm^{-1}.
MS (ESI+): m/z calcd for $C_{19}H_{12}FN_3O_5S+H^+$: 414.06 [M+H$^+$]; found: 414.14.
Anal. Calcd (%) for $C_{19}H_{12}FN_3O_5S$: C 55.21; H 2.93; N 10.17. Found: C 55.43; H 3.02; N 10.03.

2-Amino-9-(4-chlorobenzoyl)-8-hydroxy-6-(2-hydroxyethyl)-1-thia-3,6-diazaspiro[4.4]nona-2,8-diene-4,7-dione (3l)
Yield: 554 mg (97%); white solid; mp 196–198 °C (decomp.).
^{1}H NMR (400 MHz, DMSO-*d*$_6$): δ = 9.38 (br.s, 1 H), 9.12 (s, 1 H), 7.72 (m, 2 H), 7.52 (m, 2 H), 3.60–3.46 (m, 2 H), 3.33 (m, 1 H), 3.13 (m, 1 H).
^{13}C NMR (100 MHz, DMSO-*d*$_6$): δ = 186.1, 183.3, 179.3, 165.6, 154.5, 137.0, 136.8, 130.5 (2 C), 128.1 (2 C), 115.4, 84.2, 57.5, 42.8.
IR (mineral oil): 3155, 1761, 1712 cm^{-1}.
MS (ESI+): m/z calcd for $C_{15}H_{12}ClN_3O_5S+H^+$: 382.03 [M+H$^+$]; found: 382.08.
Anal. Calcd (%) for $C_{15}H_{12}ClN_3O_5S$: C 47.19; H 3.17; N 11.01. Found: C 47.35; H 3.22; N 11.12.

2-Amino-8-hydroxy-6-(2-hydroxyethyl)-9-(4-methylbenzoyl)-1-thia-3,6-diazaspiro[4.4]nona-2,8-diene-4,7-dione (3m)
Yield: 406 mg (75%); white solid; mp 196–198 °C (decomp.).
^{1}H NMR (400 MHz, DMSO-*d*$_6$): δ = 9.35 (br.s, 1 H), 9.14 (s, 1 H), 7.62 (m, 2 H), 7.28 (m, 2 H), 3.62–3.47 (m, 2 H), 3.34 (m, 1 H), 3.11 (m, 1 H), 2.37 (s, 3 H).
^{13}C NMR (100 MHz, DMSO-*d*$_6$): δ = 187.4, 183.1, 179.2, 165.5, 152.2, 142.9, 135.1, 128.9 (2 C), 128.6 (2 C), 116.7, 84.1, 57.5, 42.8, 21.1.
IR (mineral oil): 3157, 1763, 1706 cm^{-1}.
MS (ESI+): m/z calcd for $C_{16}H_{15}N_3O_5S+H^+$: 362.08 [M+H$^+$]; found: 362.11.
Anal. Calcd (%) for $C_{16}H_{15}N_3O_5S$: C 53.18; H 4.18; N 11.63. Found: C 53.06; H 4.21; N 11.69.

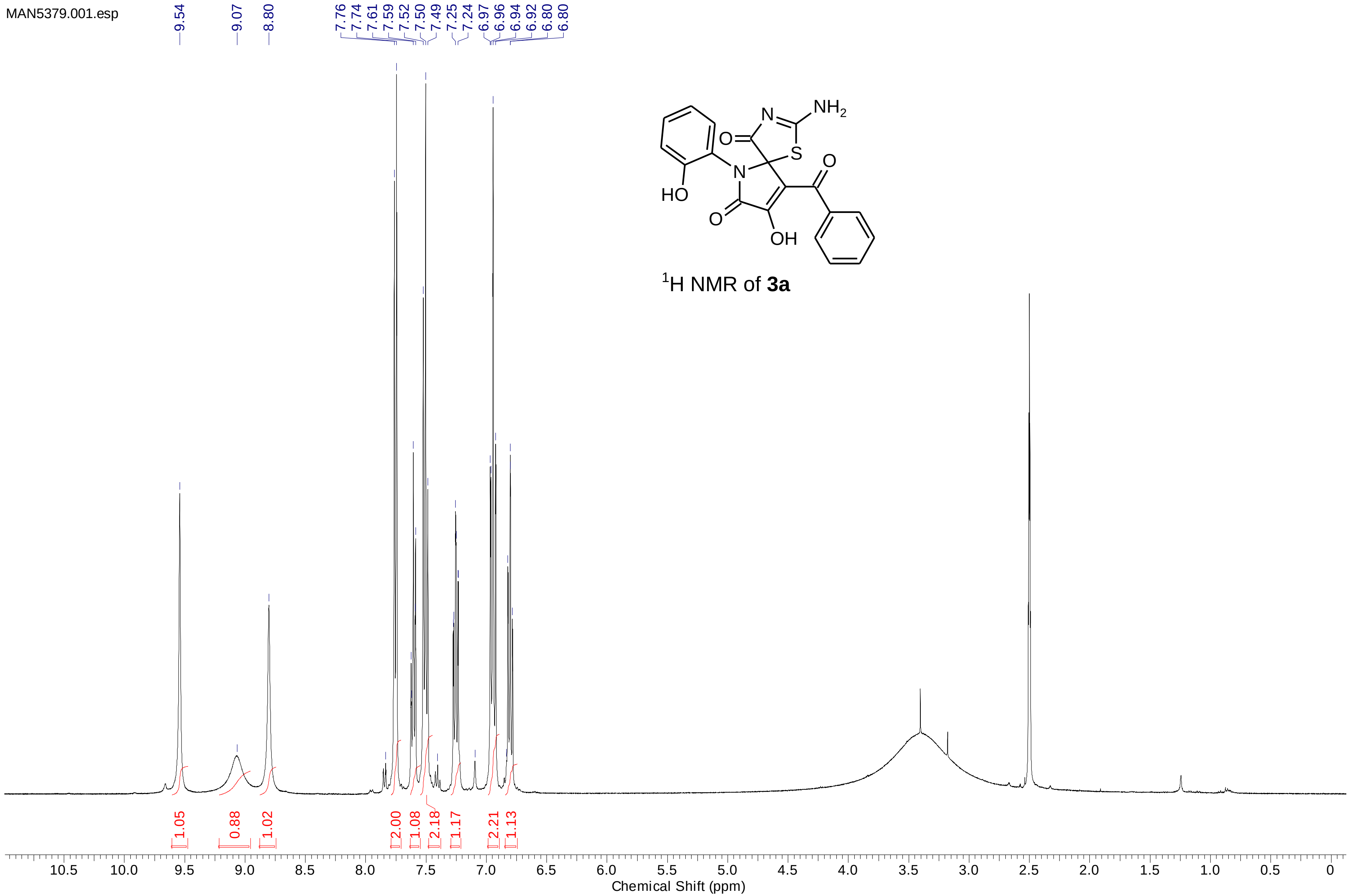
MAN5379.001.esp
9.54
9.07
8.80
7.76 7.74 7.61 7.59 7.52 7.50 7.49 7.25 7.24 6.97 6.96 6.94 6.92 6.80 6.80
NH2
N
O
S
O
N
HO
O
OH
1.05 0.88 1.02 2.00 1.08 2.18 1.17 2.21 1.13
10.5 10.0 9.5 9.0 8.5 8.0 7.5 7.0 6.5 6.0 5.5 5.0 4.5 4.0 3.5 3.0 2.5 2.0 1.5 1.0 0.5 0
Chemical Shift (ppm)

^{1}H NMR of **3a**

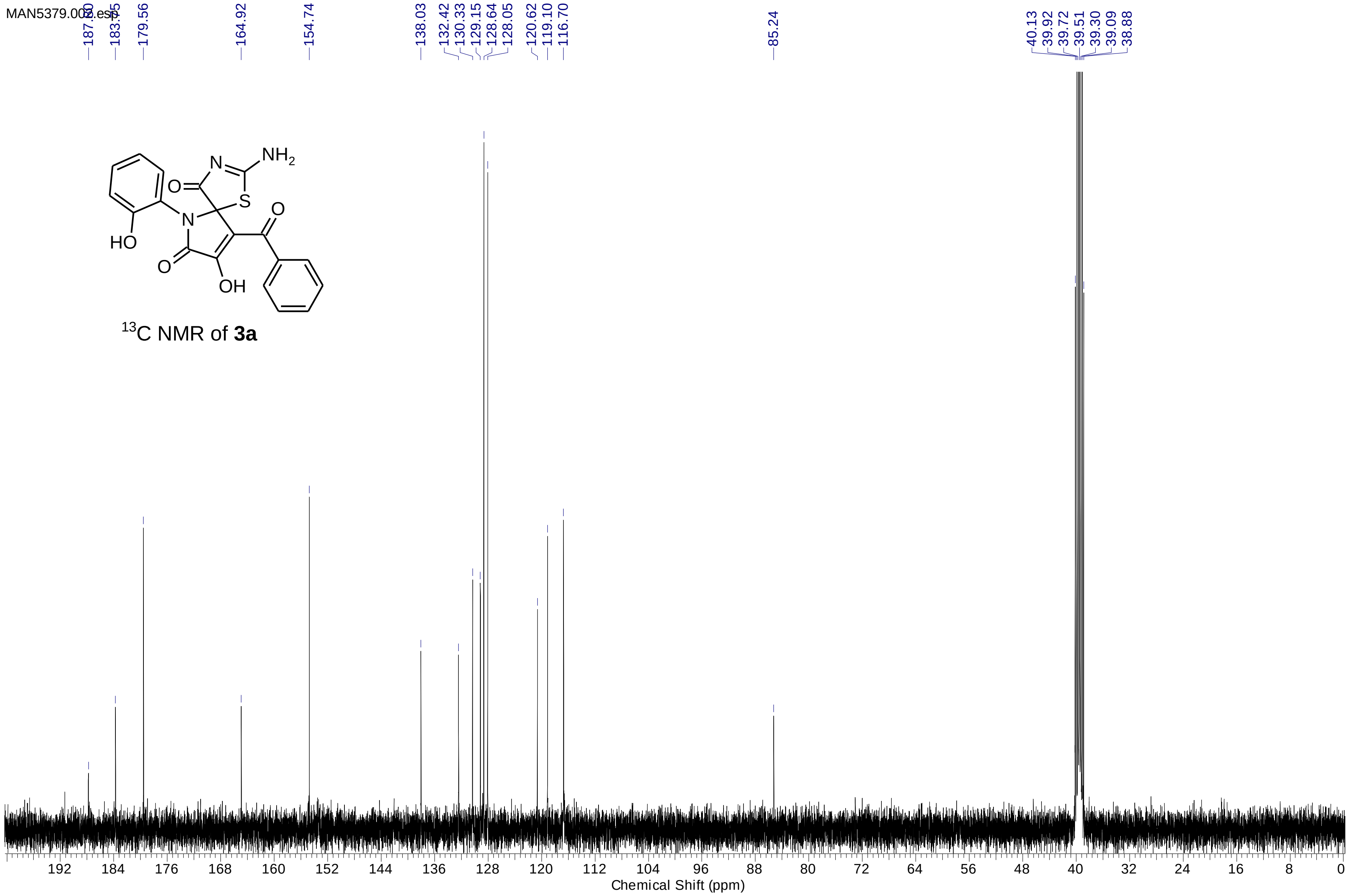
MAN5379.002.esp
187.80
183.75
179.56
164.92
154.74
138.03
132.42
130.33
129.15
128.64
128.05
120.62
119.10
116.70
85.24
40.13
39.92
39.72
39.51
39.30
39.09
38.88
N
NH2
O
S
N
O
HO
O
OH
192 184 176 168 160 152 144 136 128 120 112 104 96 88 80 72 64 56 48 40 32 24 16 8 0
Chemical Shift (ppm)

^{13}C NMR of **3a**

MAN5425.001.esp

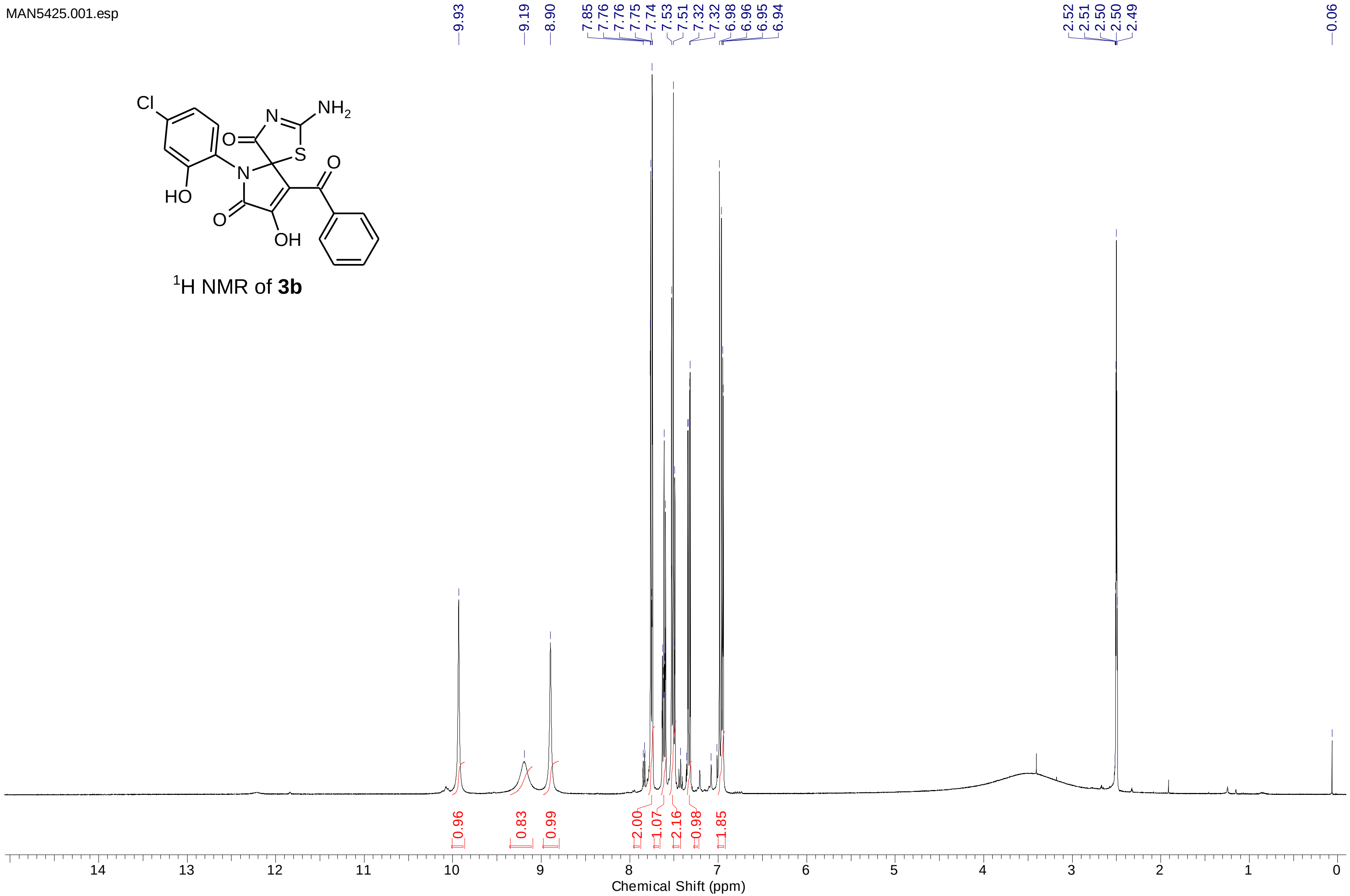

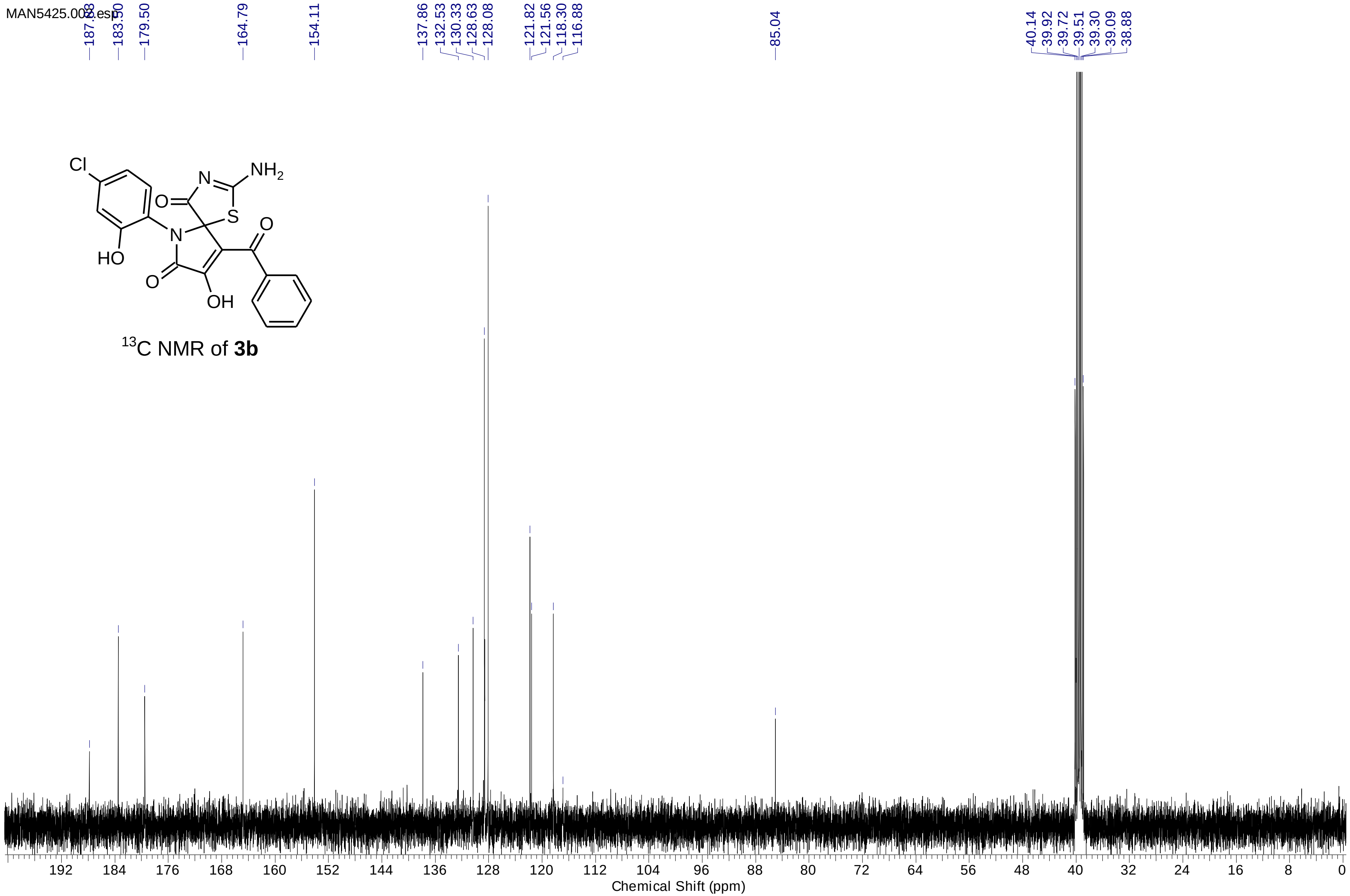
MAN5425.002.esp
187.03
183.50
179.50
164.79
154.11
137.86
132.53
130.33
128.63
128.08
121.82
121.56
118.30
116.88
85.04
40.14
39.92
39.72
39.51
39.30
39.09
38.88
Cl
N
NH2
O
S
O
N
HO
O
OH
Chemical Shift (ppm)

^{13}C NMR of **3b**

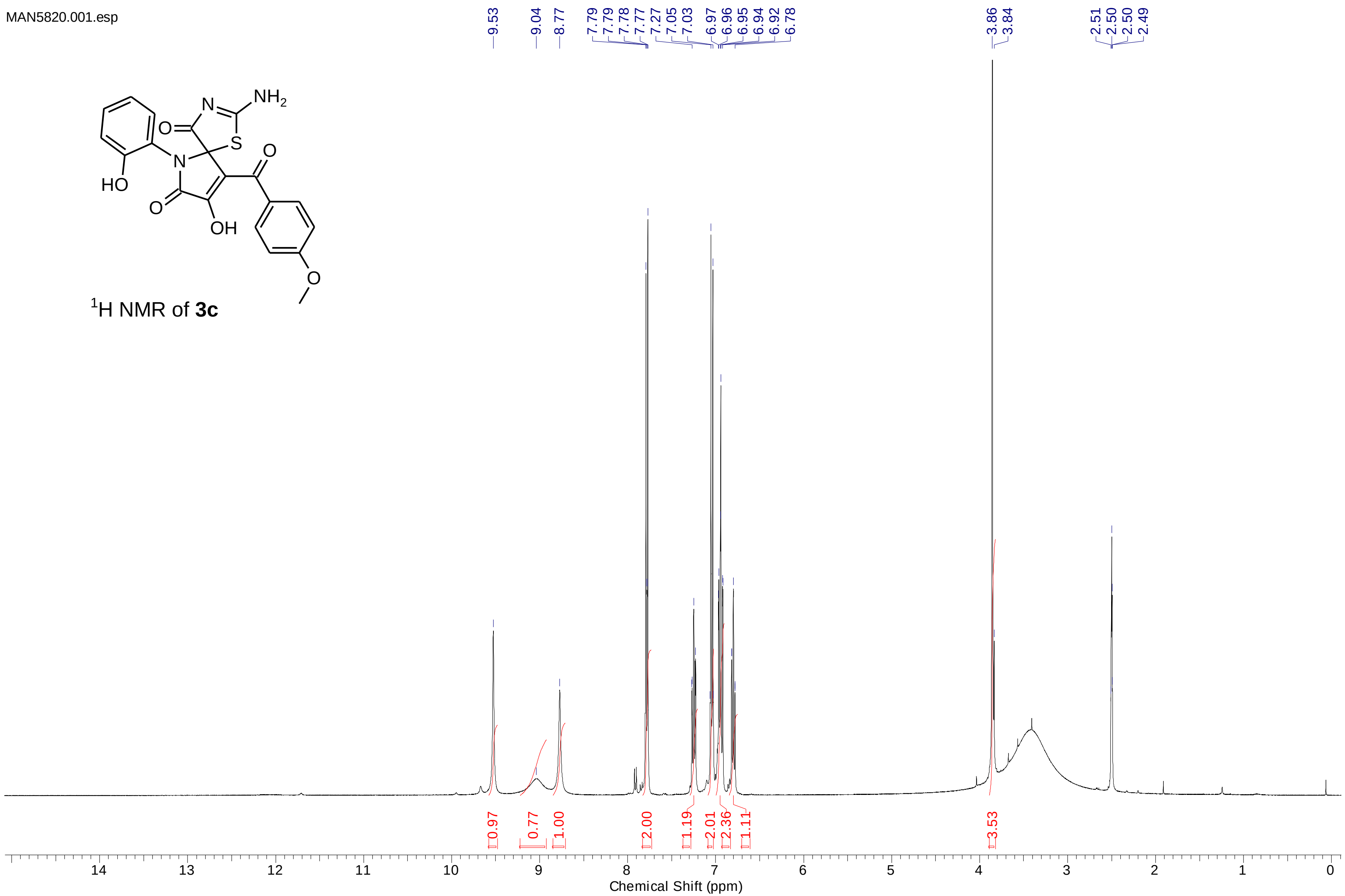
MAN5820.001.esp
9.53
9.04
8.77
7.79
7.79
7.78
7.77
7.27
7.05
7.03
6.97
6.96
6.95
6.94
6.92
6.78
3.86
3.84
2.51
2.50
2.50
2.49
NH2
N
O
S
O
N
HO
O
OH
O
0.97
0.77
1.00
2.00
1.19
2.01
2.36
1.11
3.53
14
13
12
11
10
9
8
7
6
5
4
3
2
1
0
Chemical Shift (ppm)

^{1}H NMR of **3c**

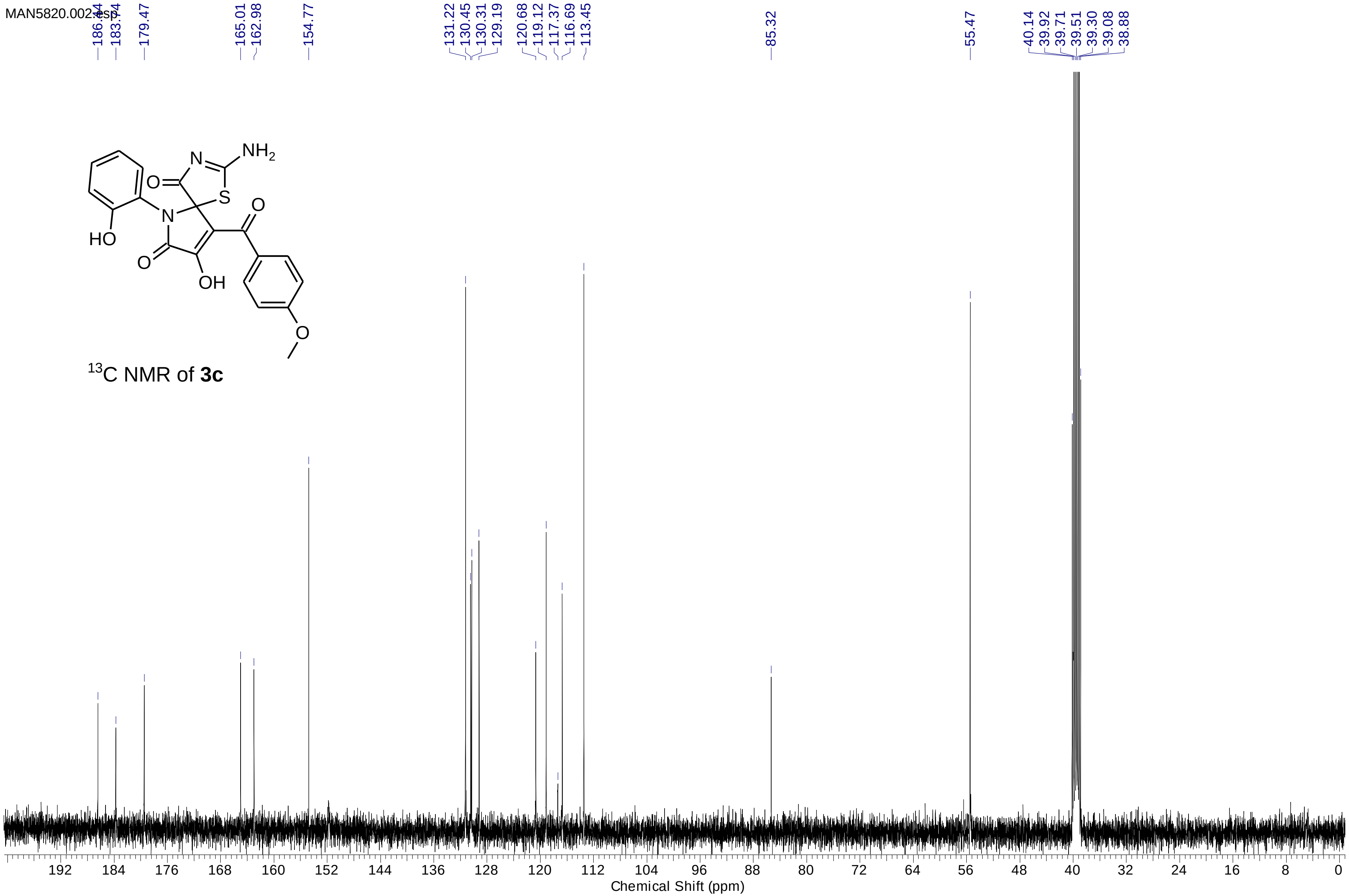
MAN5820.002.esp
186.44
183.74
179.47
165.01
162.98
154.77
131.22
130.45
130.31
129.19
120.68
119.12
117.37
116.69
113.45
85.32
55.47
40.14
39.92
39.71
39.51
39.30
39.08
38.88
NH2
N
O
S
O
N
HO
O
OH
O
192 184 176 168 160 152 144 136 128 120 112 104 96 88 80 72 64 56 48 40 32 24 16 8 0
Chemical Shift (ppm)

^{13}C NMR of **3c**

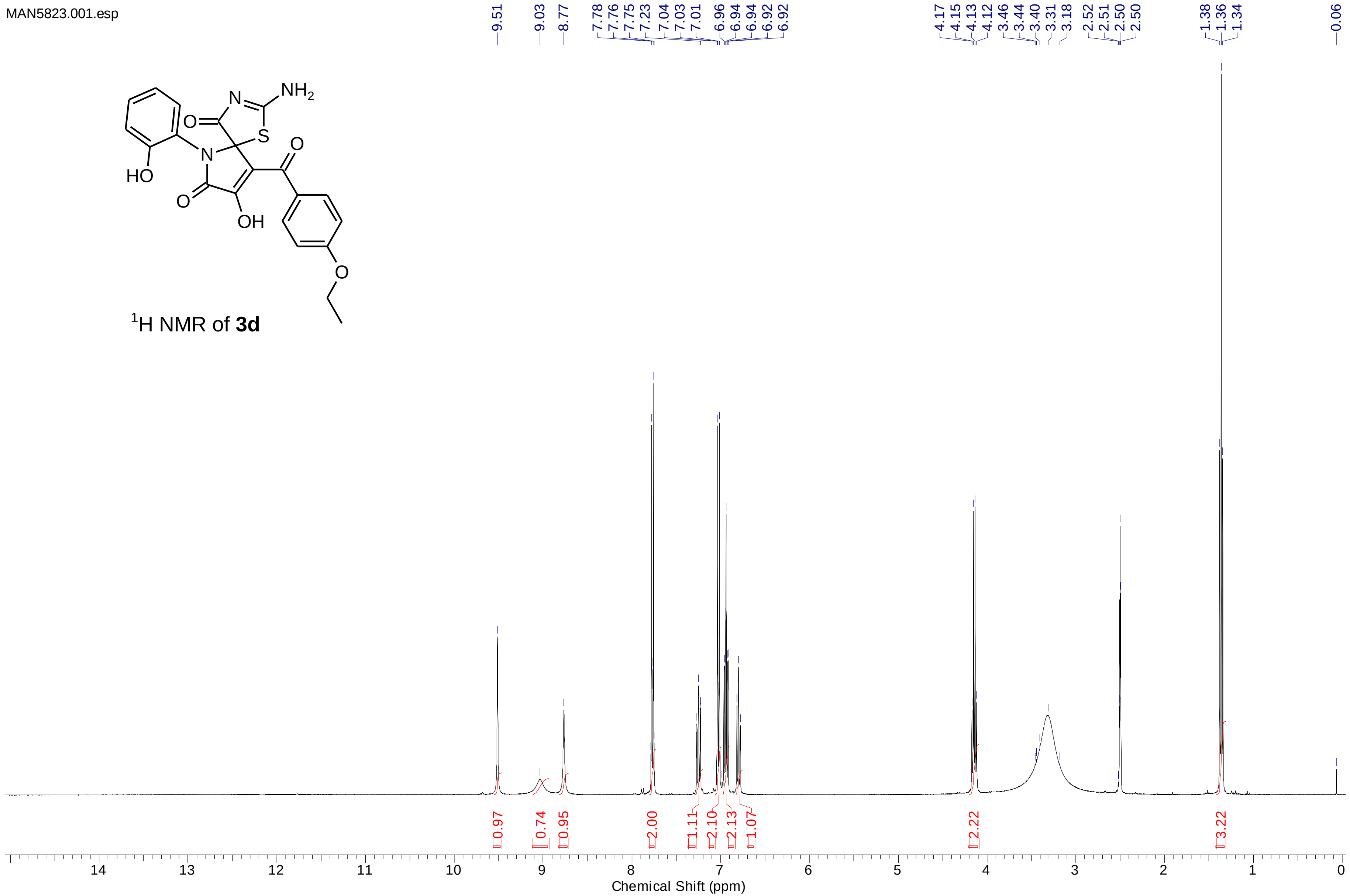
MAN5823.001.esp
9.51
9.03
8.77
7.78
7.76
7.75
7.23
7.04
7.03
7.01
6.96
6.94
6.94
6.92
6.92
4.17
4.15
4.13
4.12
3.46
3.44
3.40
3.31
3.18
2.52
2.51
2.50
2.50
1.38
1.36
1.34
0.06
NH2
N
O
S
O
N
HO
O
OH
O
1H NMR of 3d
0.97
0.74
0.95
2.00
1.11
2.10
2.13
1.07
2.22
3.22
14
13
12
11
10
9
8
7
6
5
4
3
2
1
0
Chemical Shift (ppm)

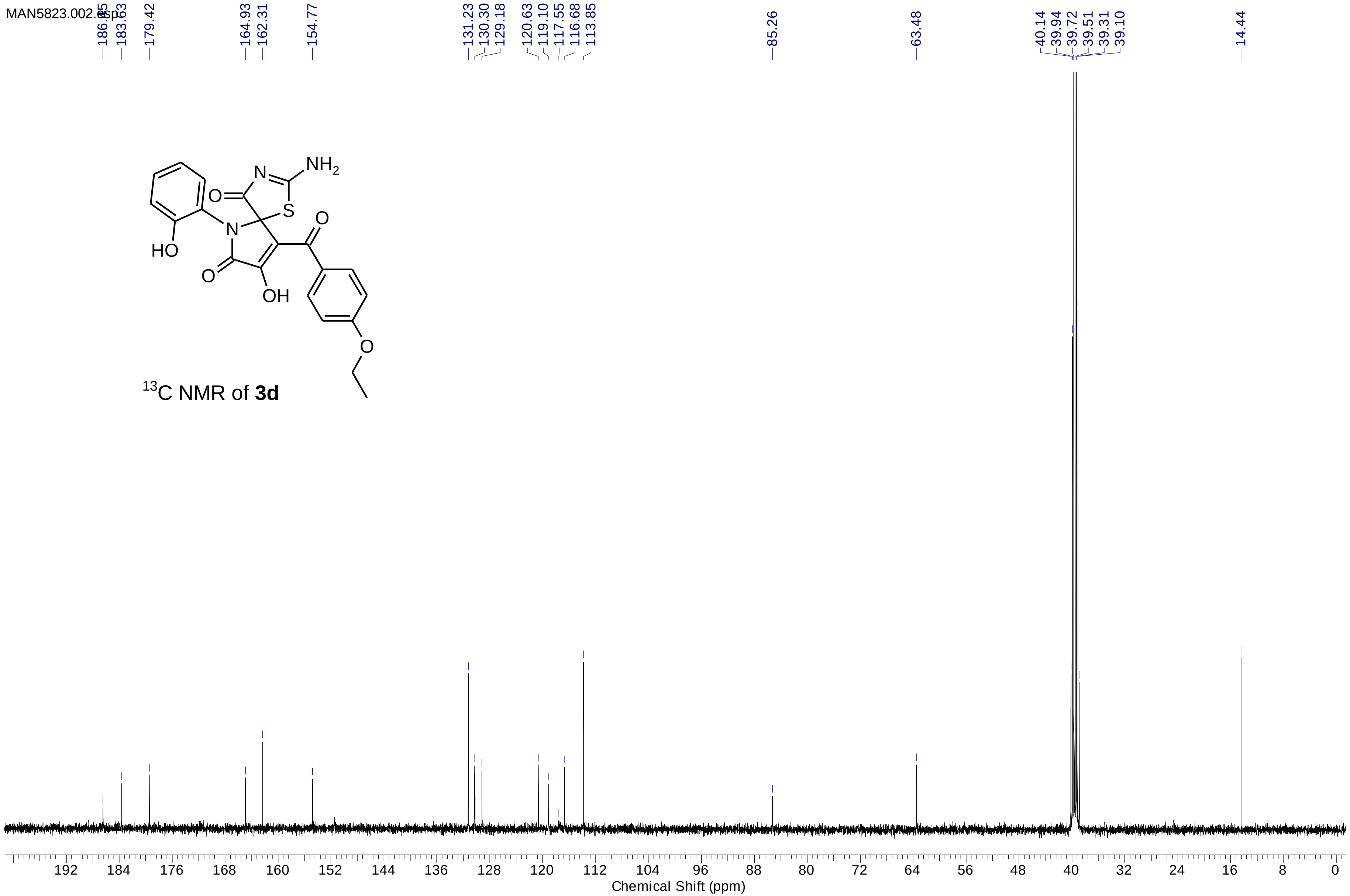
MAN5823.002.esp
186.85
183.63
179.42
164.93
162.31
154.77
131.23
130.30
129.18
120.63
119.10
117.55
116.68
113.85
85.26
63.48
40.14
39.94
39.72
39.51
39.31
39.10
14.44
NH2
N
O
S
N
HO
O
OH
O
O
Chemical Shift (ppm)

^{13}C NMR of **3d**

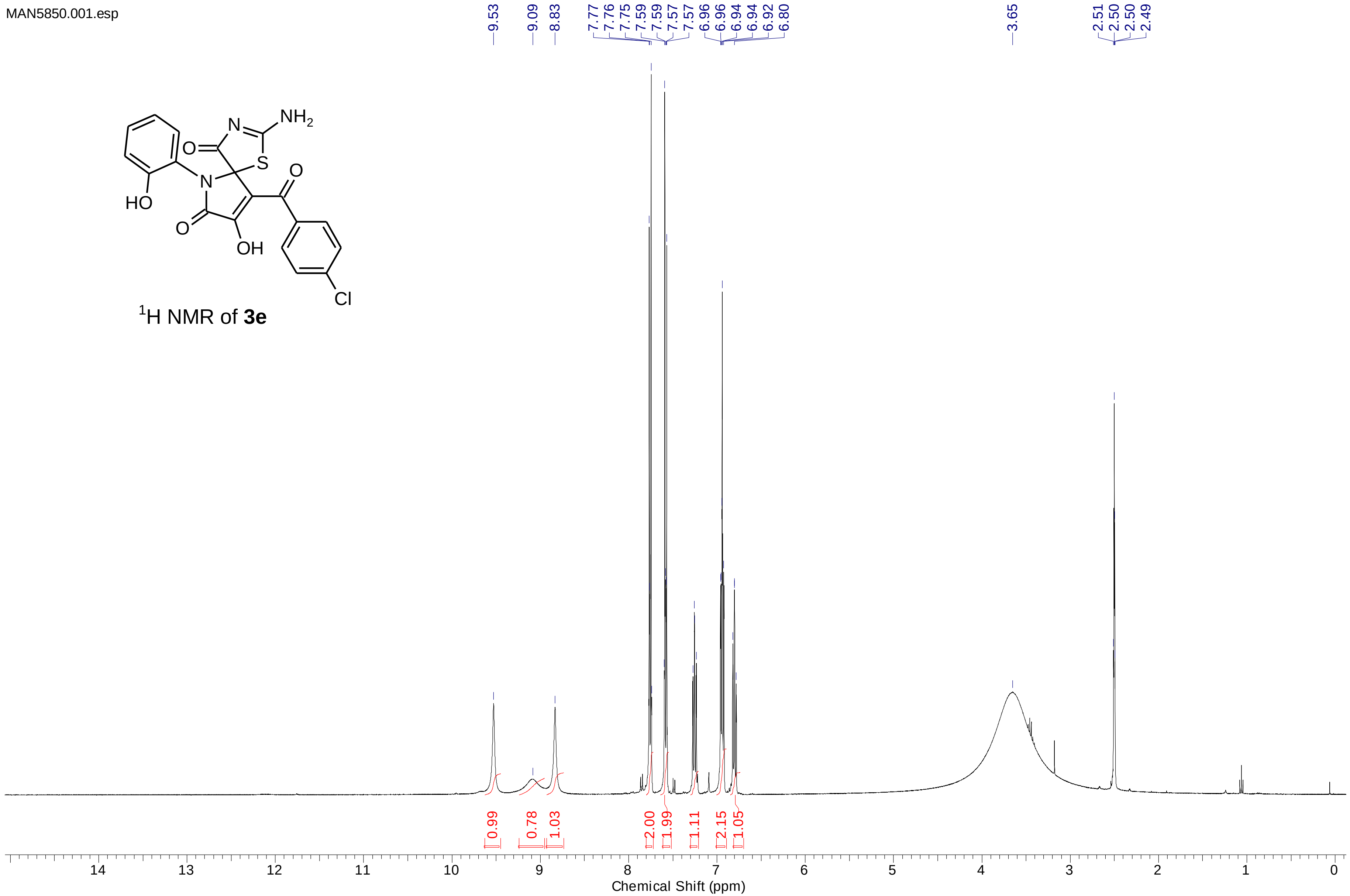
MAN5850.001.esp
9.53
9.09
8.83
7.77
7.76
7.75
7.59
7.59
7.57
7.57
6.96
6.96
6.94
6.94
6.92
6.80
3.65
2.51
2.50
2.50
2.49
0.99
0.78
1.03
2.00
1.99
1.11
2.15
1.05
14
13
12
11
10
9
8
7
6
5
4
3
2
1
0
Chemical Shift (ppm)

¹H NMR of **3e**

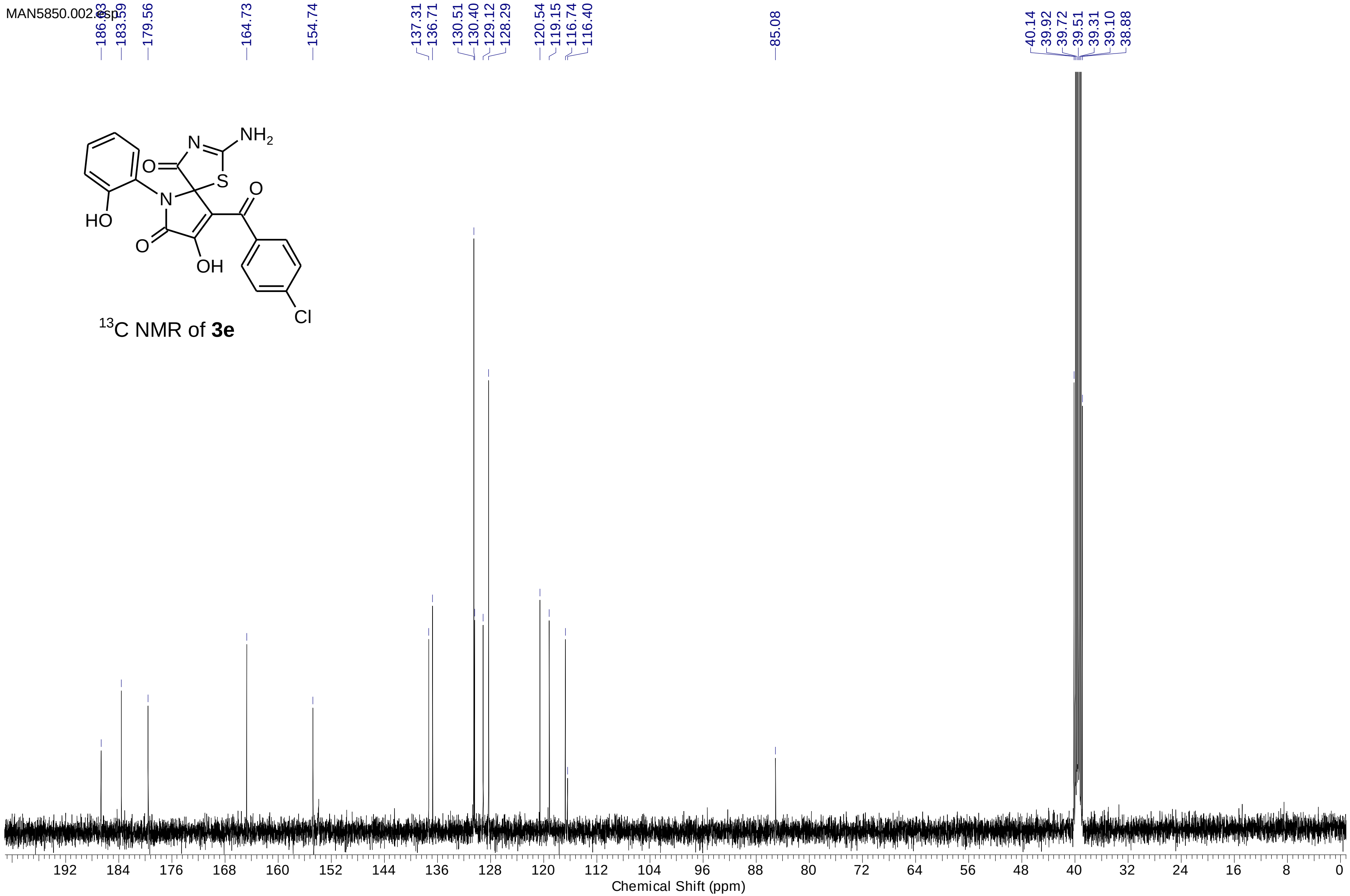
MAN5850.002.esp
186.63
183.59
179.56
164.73
154.74
137.31
136.71
130.51
130.40
129.12
128.29
120.54
119.15
116.74
116.40
85.08
40.14
39.92
39.72
39.51
39.31
39.10
38.88
NH2
N
O
S
N
HO
O
O
OH
Cl
192
184
176
168
160
152
144
136
128
120
112
104
96
88
80
72
64
56
48
40
32
24
16
8
0
Chemical Shift (ppm)

^{13}C NMR of **3e**

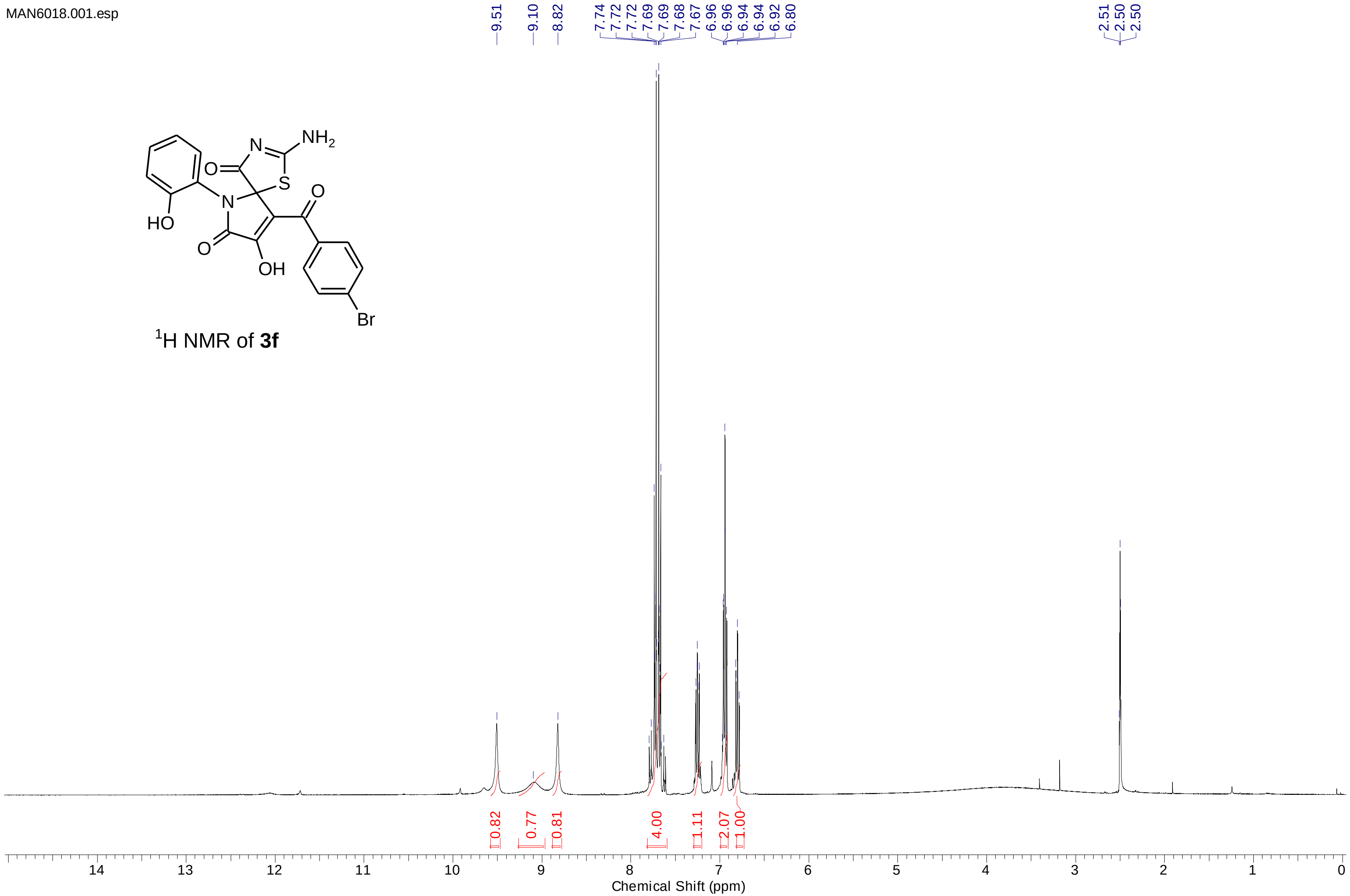
MAN6018.001.esp
9.51
9.10
8.82
7.74 7.72 7.72 7.69 7.69 7.68 7.67 6.96 6.96 6.94 6.94 6.92 6.80
2.51 2.50 2.50
NH2
N
O
S
N
HO
O
OH
Br
0.82
0.77
0.81
4.00
1.11
2.07
1.00
14 13 12 11 10 9 8 7 6 5 4 3 2 1 0
Chemical Shift (ppm)

^{1}H NMR of **3f**

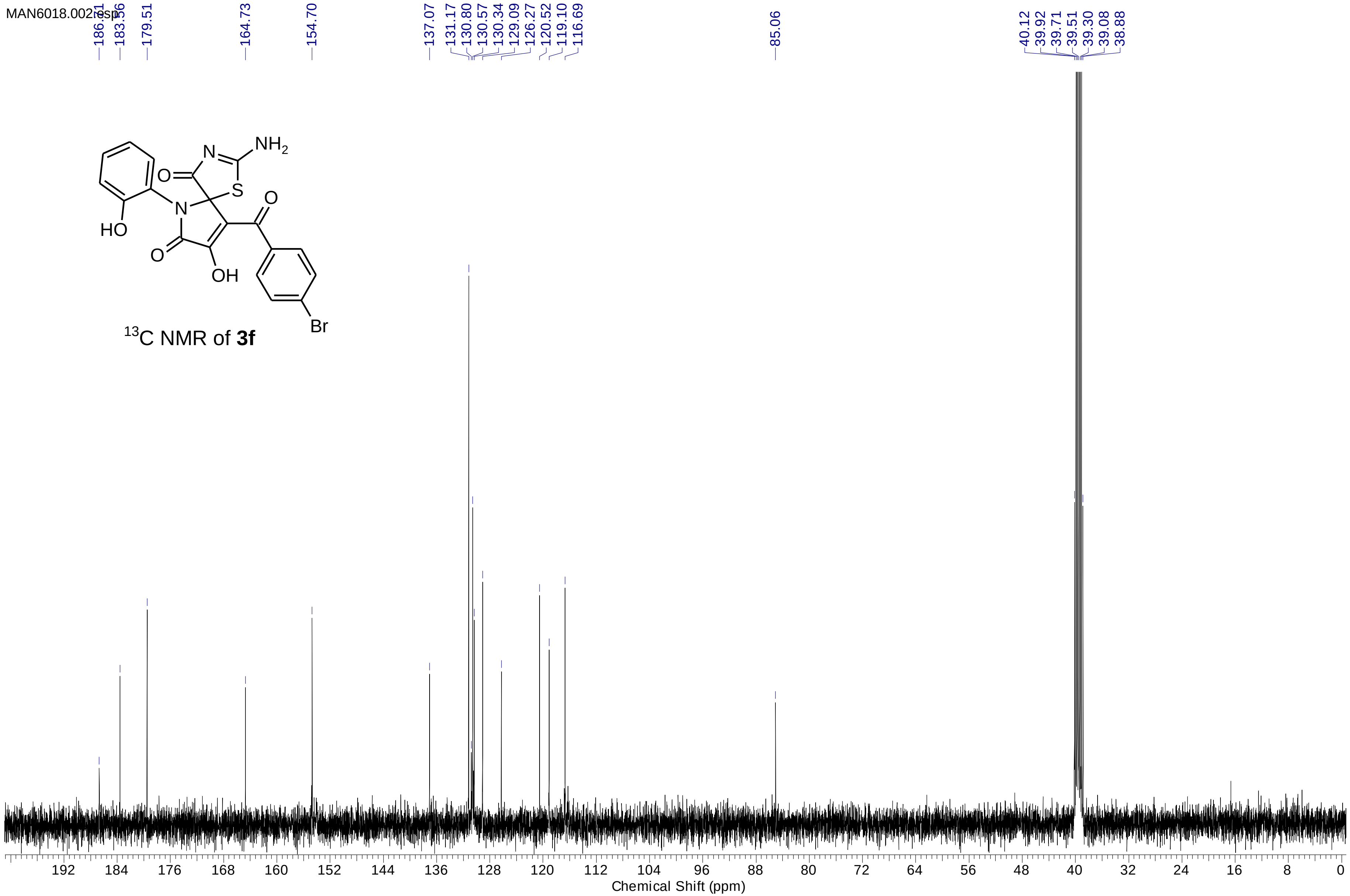
MAN6018.002.esp
186.61
183.56
179.51
164.73
154.70
137.07
131.17
130.80
130.57
130.34
129.09
126.27
120.52
119.10
116.69
85.06
40.12
39.92
39.71
39.51
39.30
39.08
38.88
NH2
N
O
S
O
N
HO
O
OH
Br
^{13}C NMR of **3f**
192 184 176 168 160 152 144 136 128 120 112 104 96 88 80 72 64 56 48 40 32 24 16 8 0
Chemical Shift (ppm)

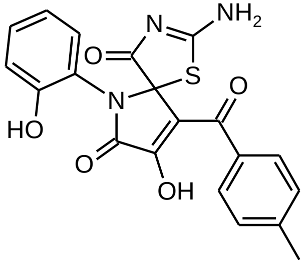

^{1}H NMR of **3g** (solvate with methanol)

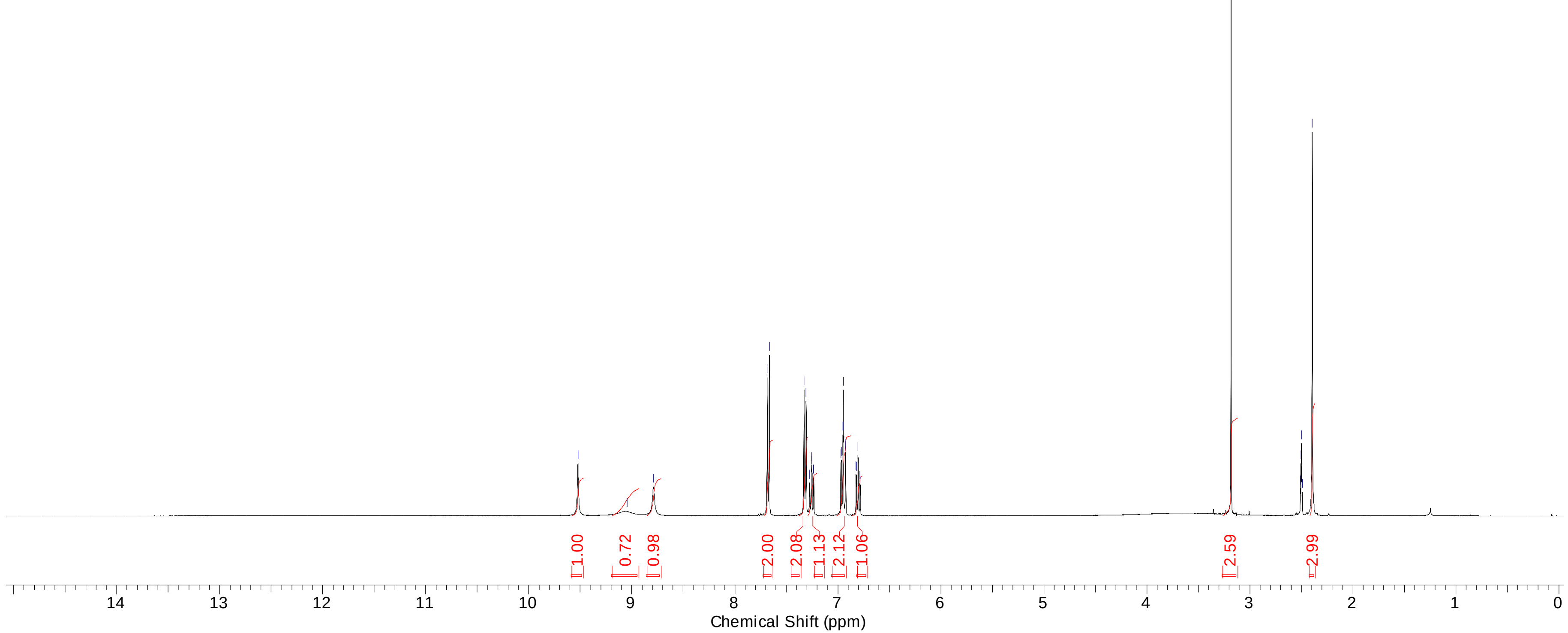

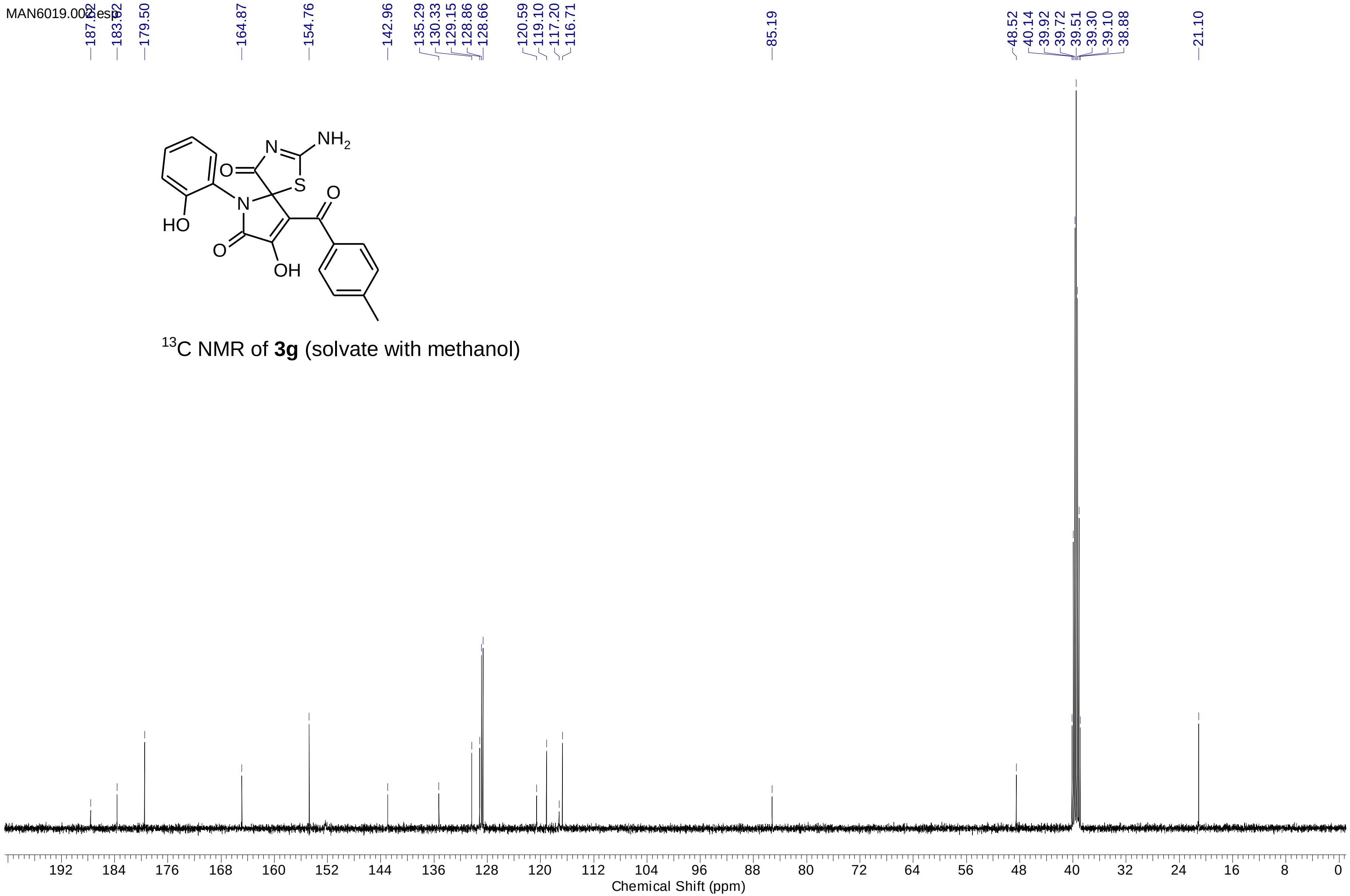

^{13}C NMR of **3g** (solvate with methanol)

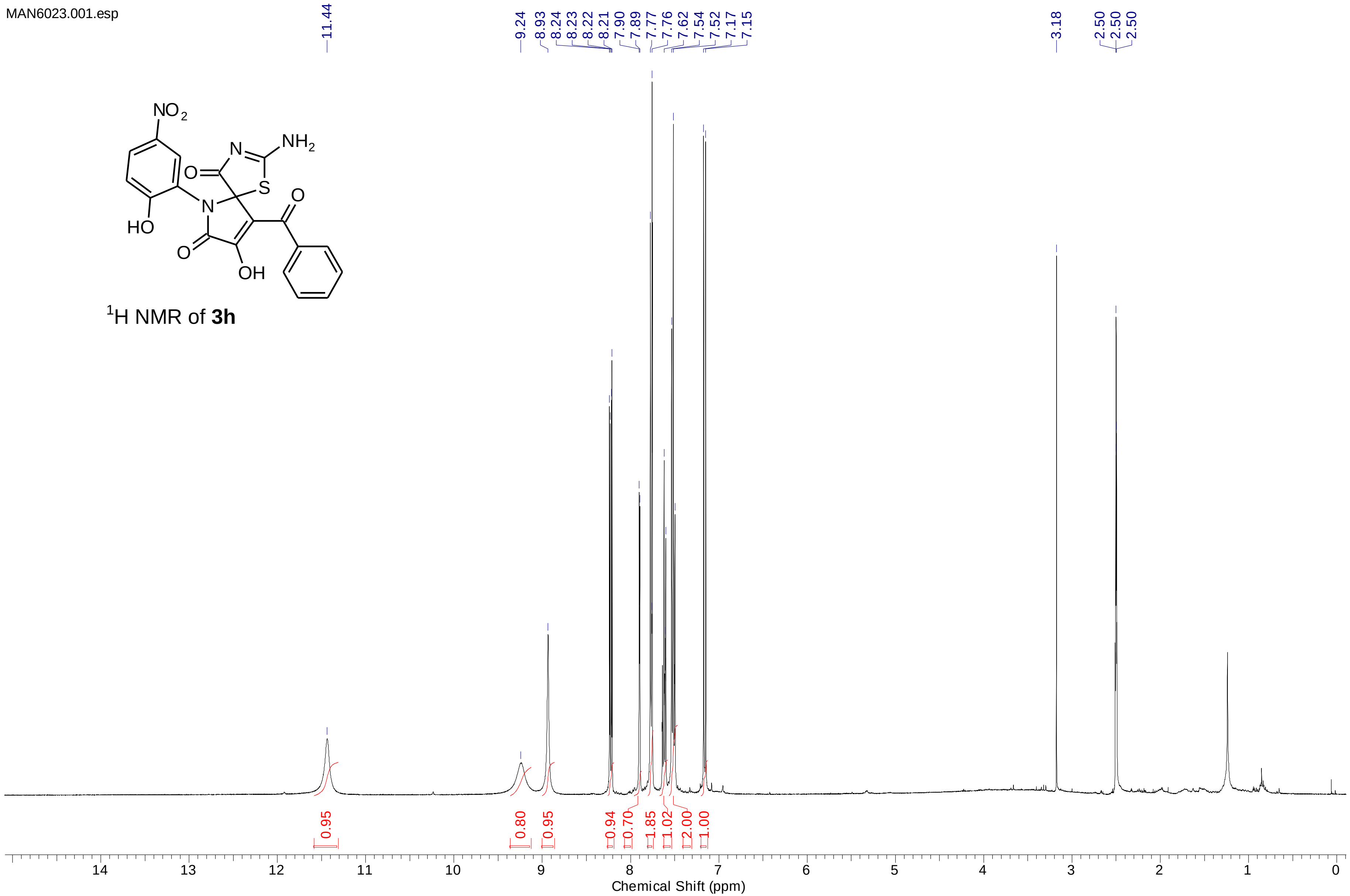
MAN6023.001.esp
11.44
9.24
8.93
8.24
8.23
8.22
8.21
7.90
7.89
7.77
7.76
7.62
7.54
7.52
7.17
7.15
3.18
2.50
2.50
2.50
NO2
NH2
N
O
S
O
N
HO
O
OH
1H NMR of 3h
0.95
0.80
0.95
0.94
0.70
1.85
1.02
2.00
1.00
14
13
12
11
10
9
8
7
6
5
4
3
2
1
0
Chemical Shift (ppm)

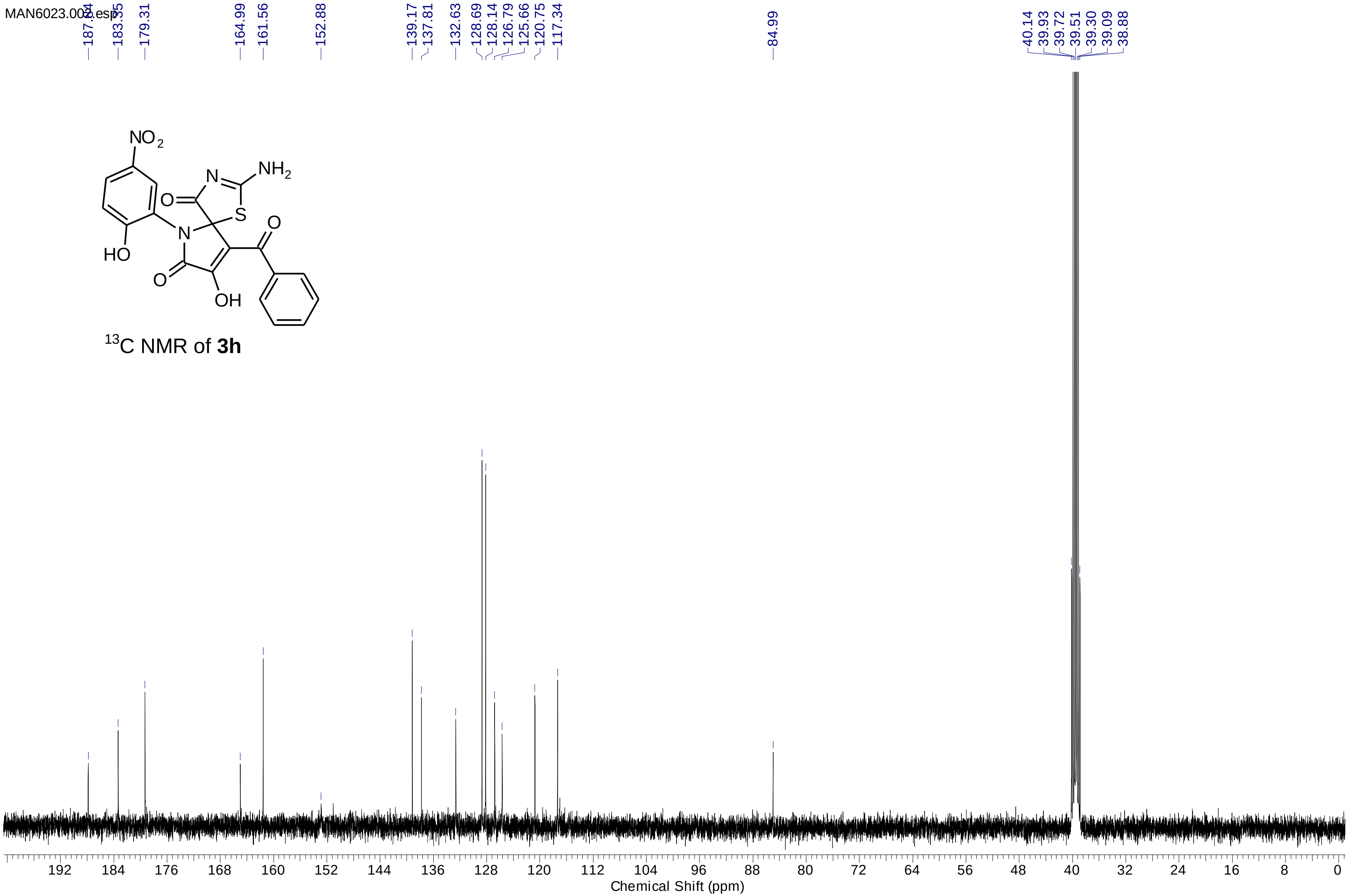
MAN6023.002.esp
187.64
183.35
179.31
164.99
161.56
152.88
139.17
137.81
132.63
128.69
128.14
126.79
125.66
120.75
117.34
84.99
40.14
39.93
39.72
39.51
39.30
39.09
38.88
192 184 176 168 160 152 144 136 128 120 112 104 96 88 80 72 64 56 48 40 32 24 16 8 0
Chemical Shift (ppm)
NO2
NH2
N
O
S
O
N
HO
O
OH

^{13}C NMR of **3h**

MAN6041.001.esp

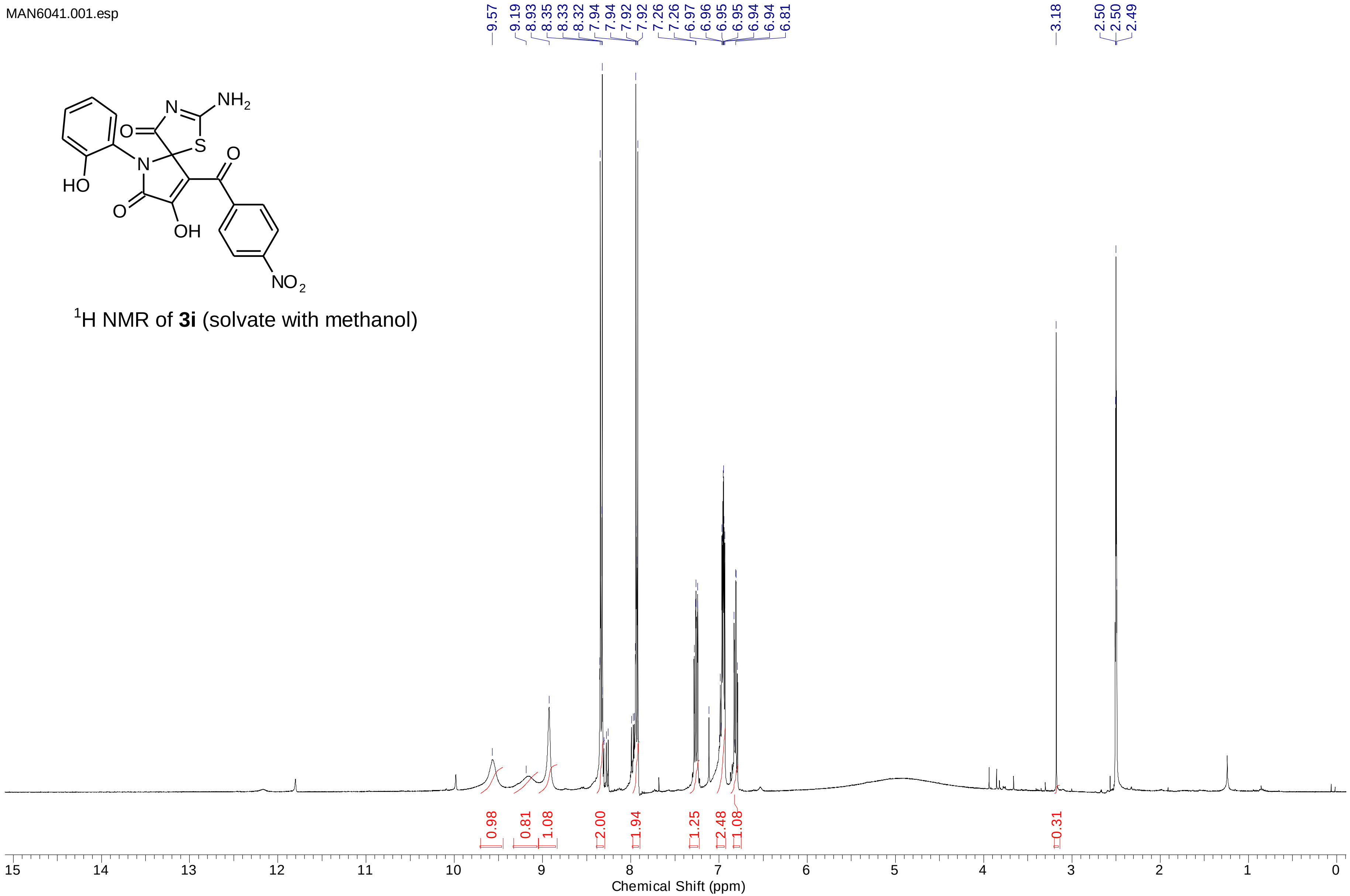

^{1}H NMR of **3i** (solvate with methanol)

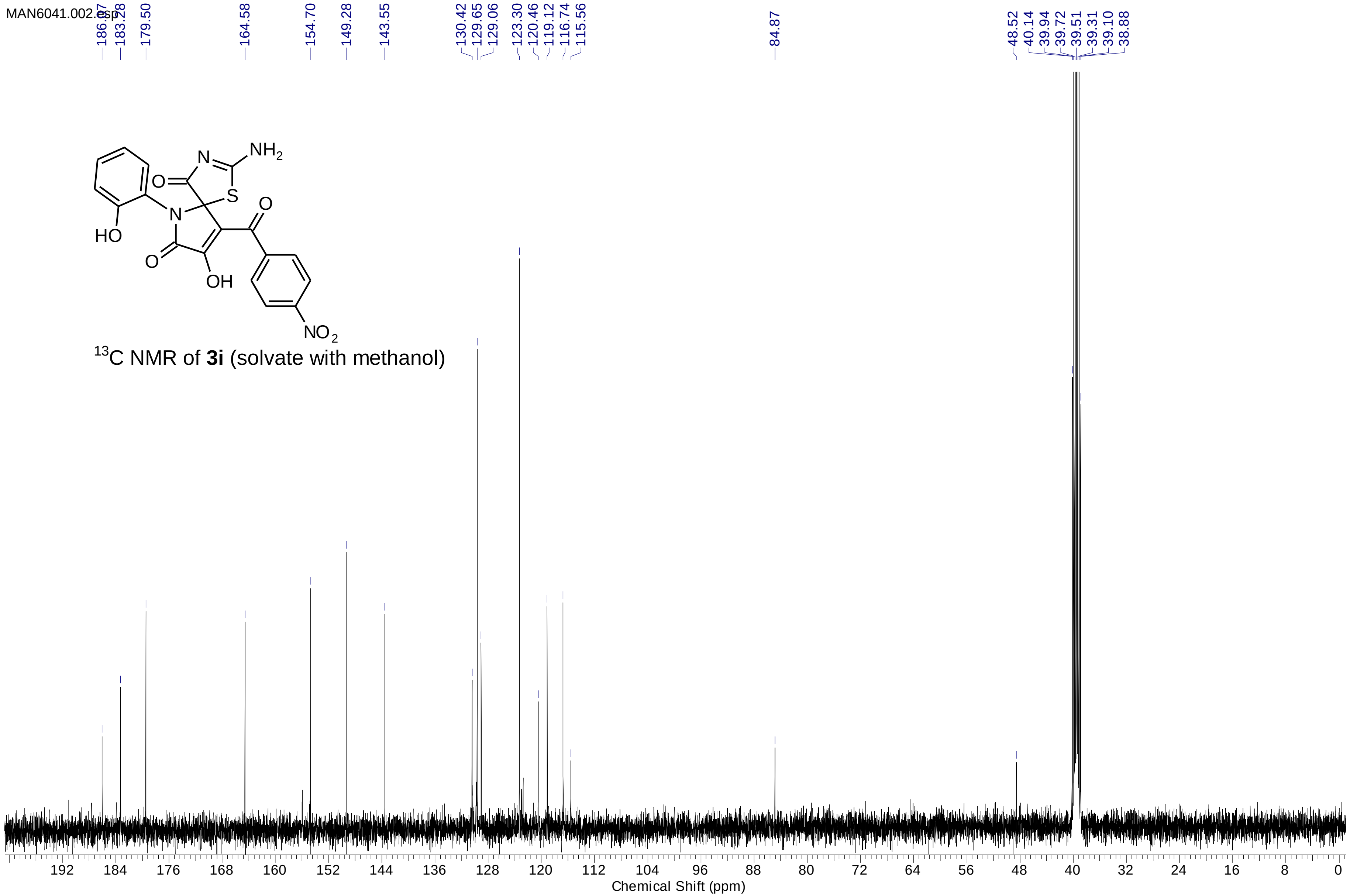

^{13}C NMR of **3i** (solvate with methanol)

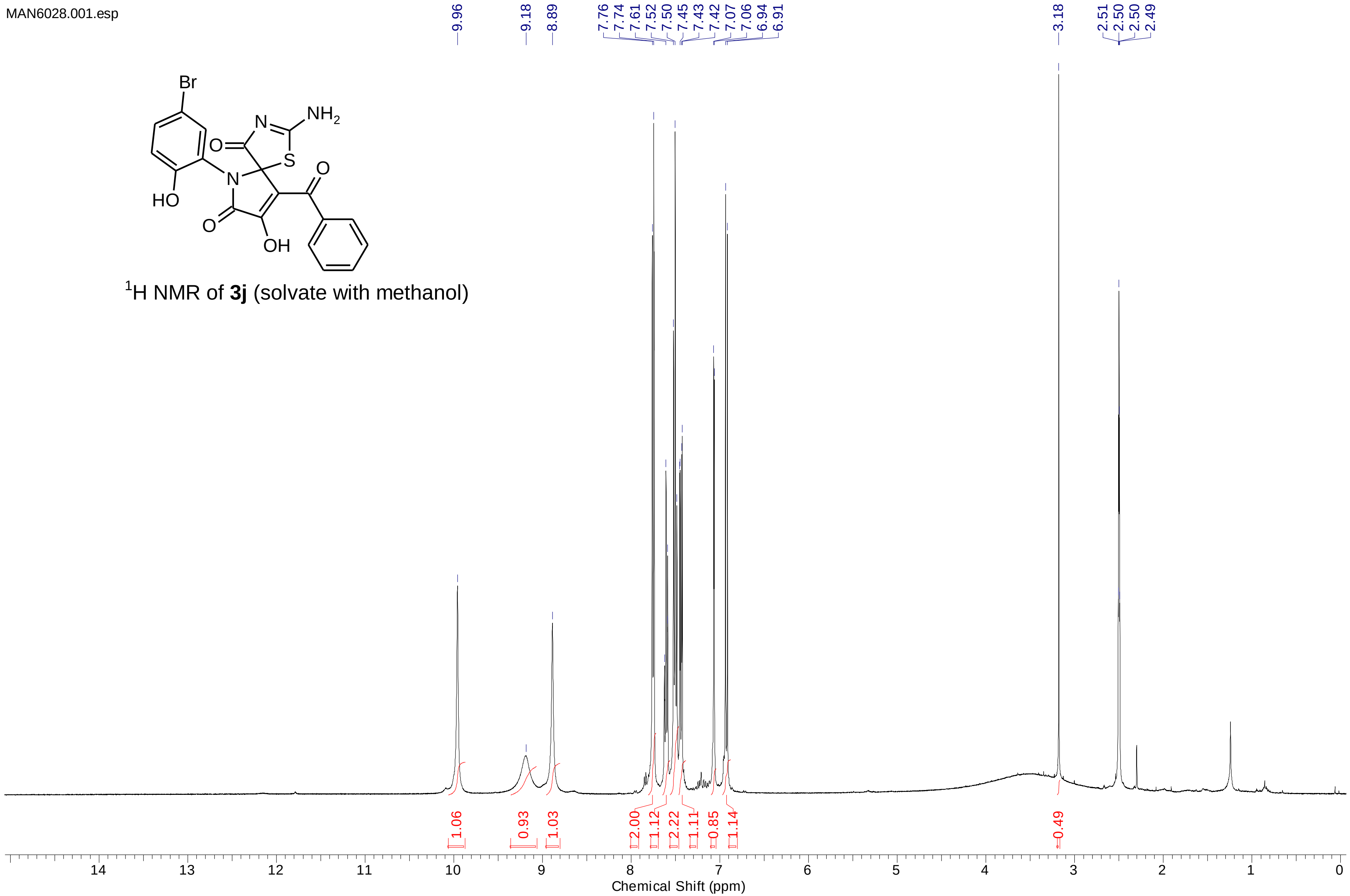

^{1}H NMR of **3j** (solvate with methanol)

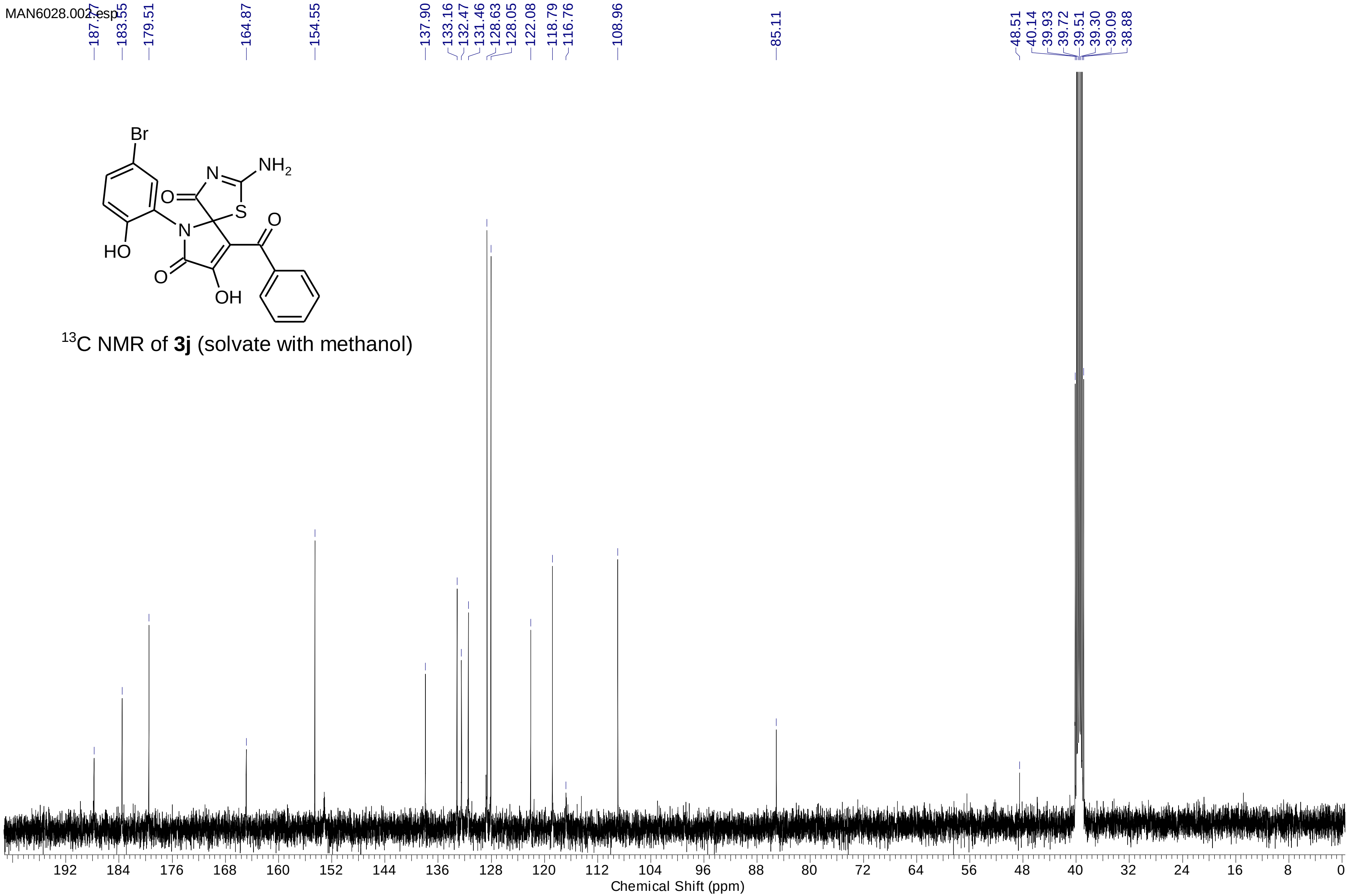

^{13}C NMR of **3j** (solvate with methanol)

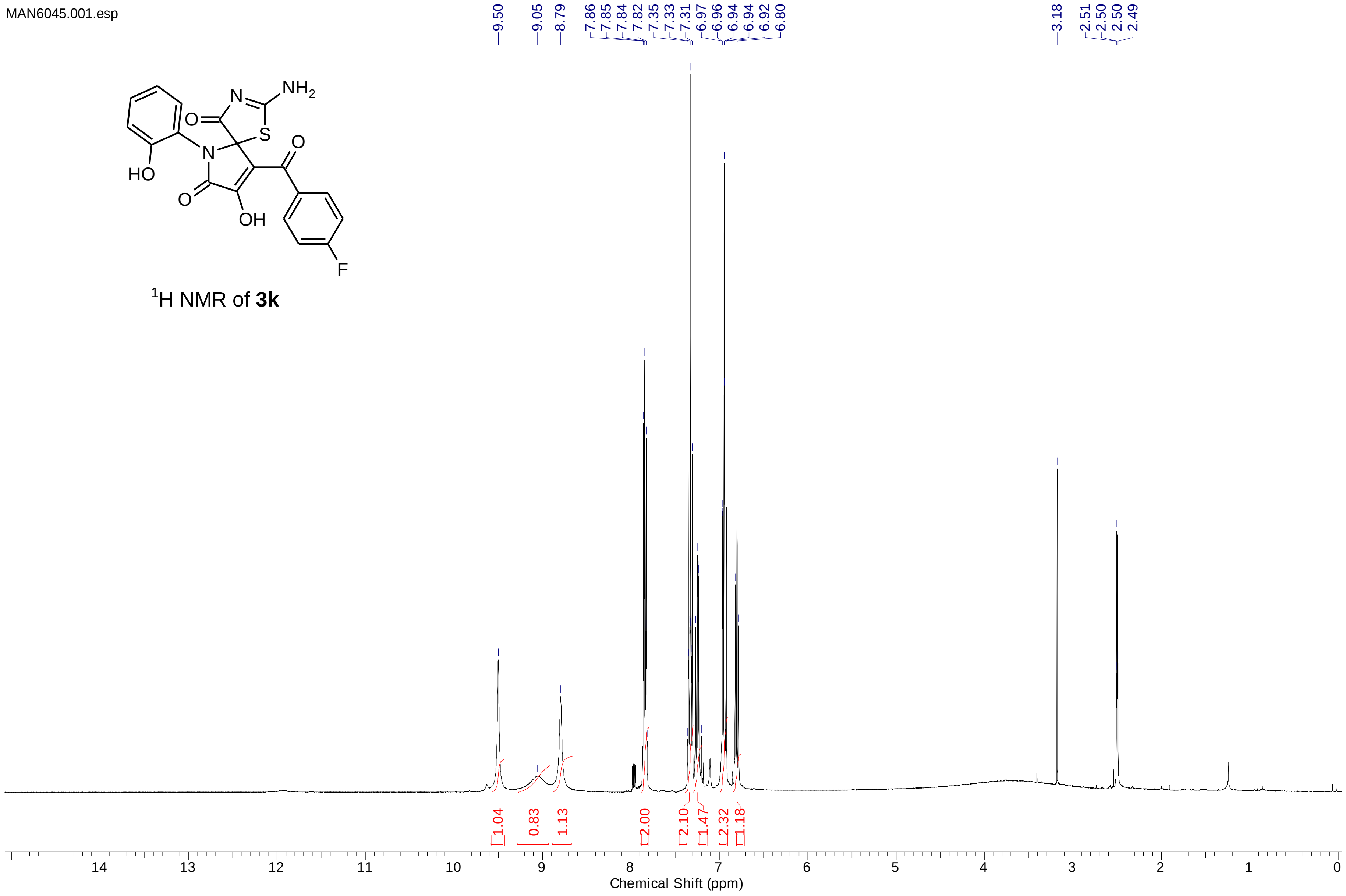
MAN6045.001.esp
9.50
9.05
8.79
7.86
7.85
7.84
7.82
7.35
7.33
7.31
6.97
6.96
6.94
6.94
6.92
6.80
3.18
2.51
2.50
2.50
2.49
1.04
0.83
1.13
2.00
2.10
1.47
2.32
1.18
14
13
12
11
10
9
8
7
6
5
4
3
2
1
0
Chemical Shift (ppm)

^{1}H NMR of **3k**

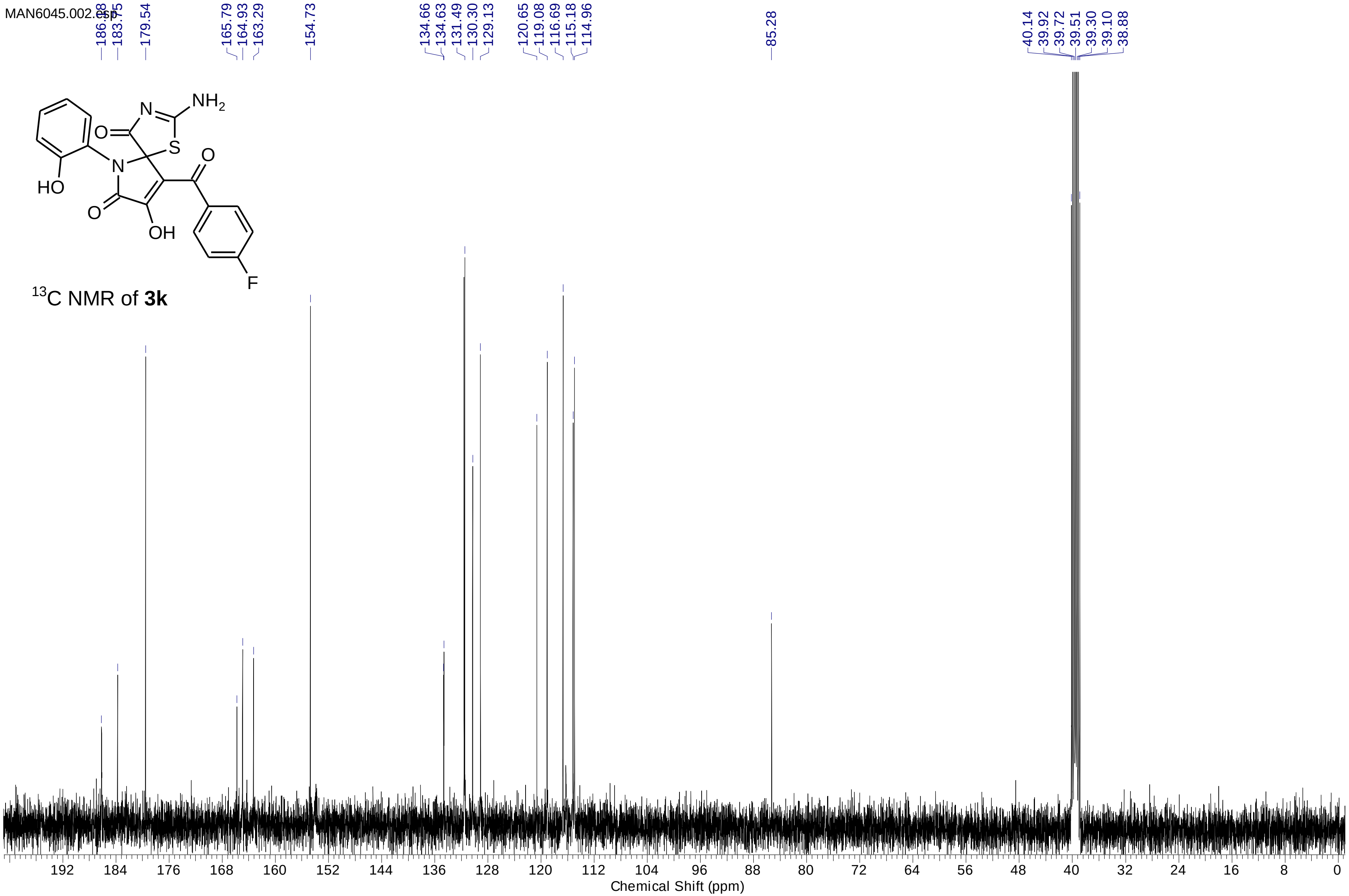
MAN6045.002.esp
186.88
183.75
179.54
165.79
164.93
163.29
154.73
134.66
134.63
131.49
130.30
129.13
120.65
119.08
116.69
115.18
114.96
85.28
40.14
39.92
39.72
39.51
39.30
39.10
38.88
NH2
N
O
S
O
N
HO
O
OH
F
Chemical Shift (ppm)

^{13}C NMR of **3k**

MAN6153.001.esp

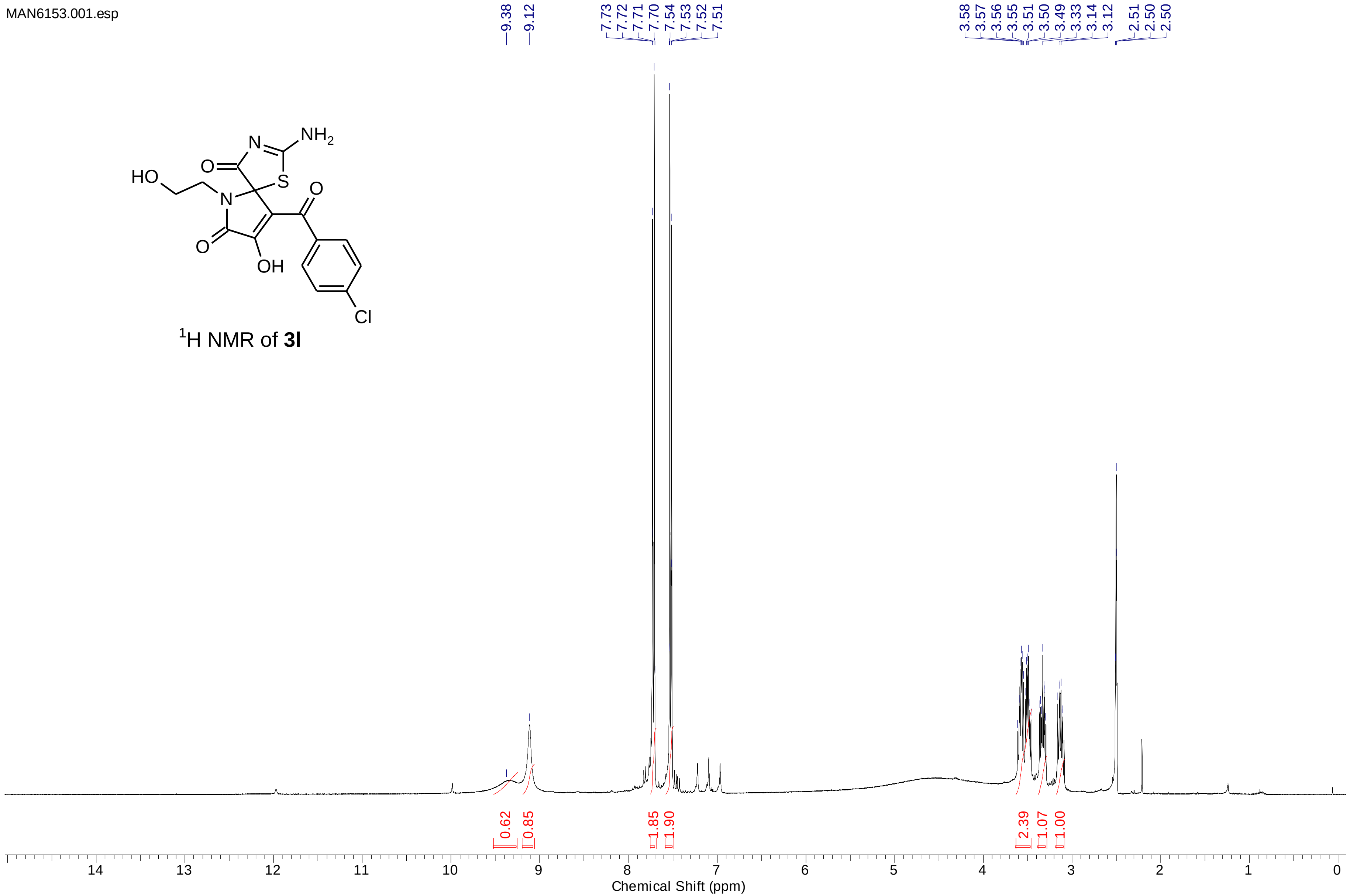

^{1}H NMR of **3l**

MAN6155.001.esp

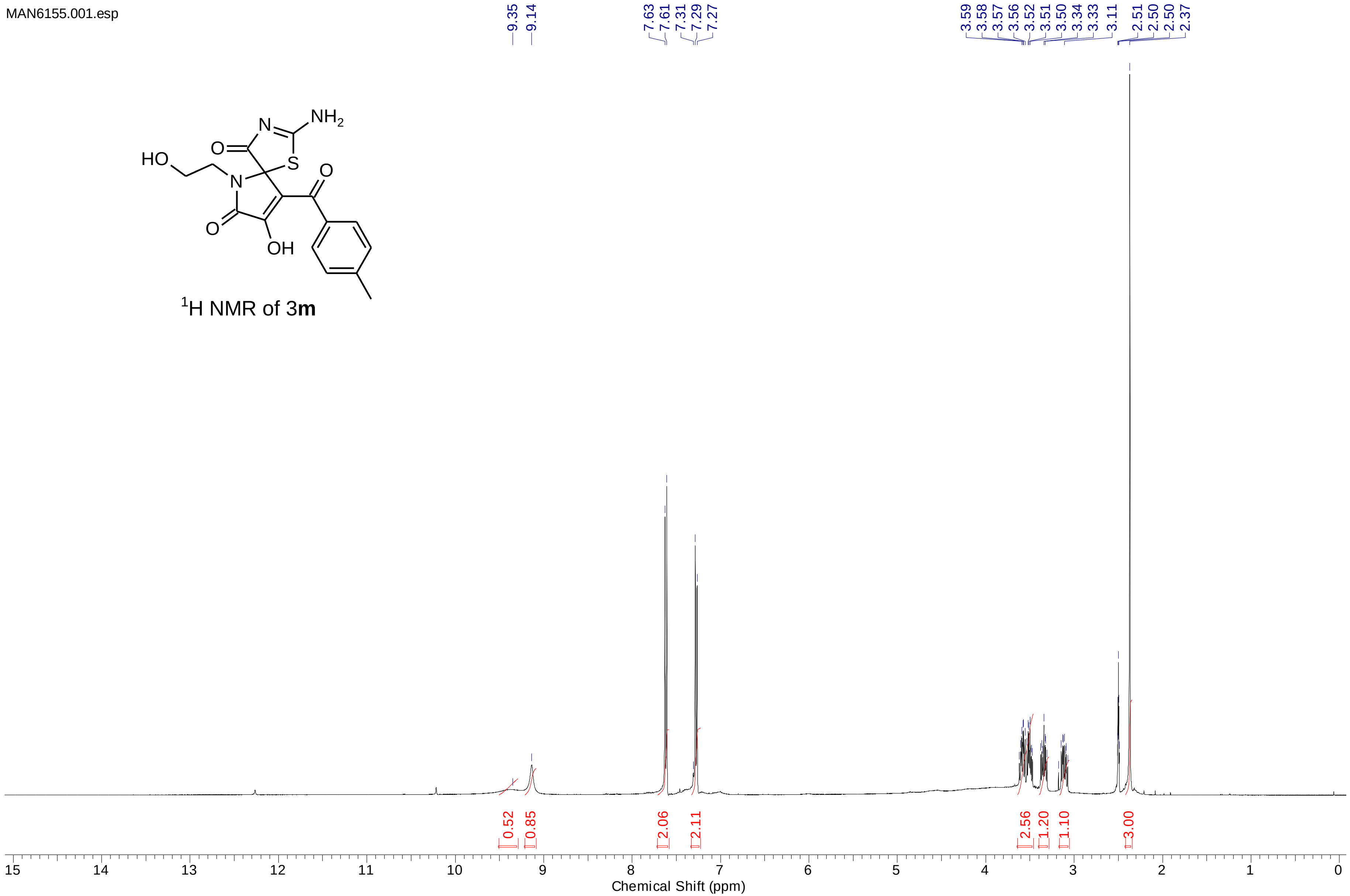

^{1}H NMR of 3**m**

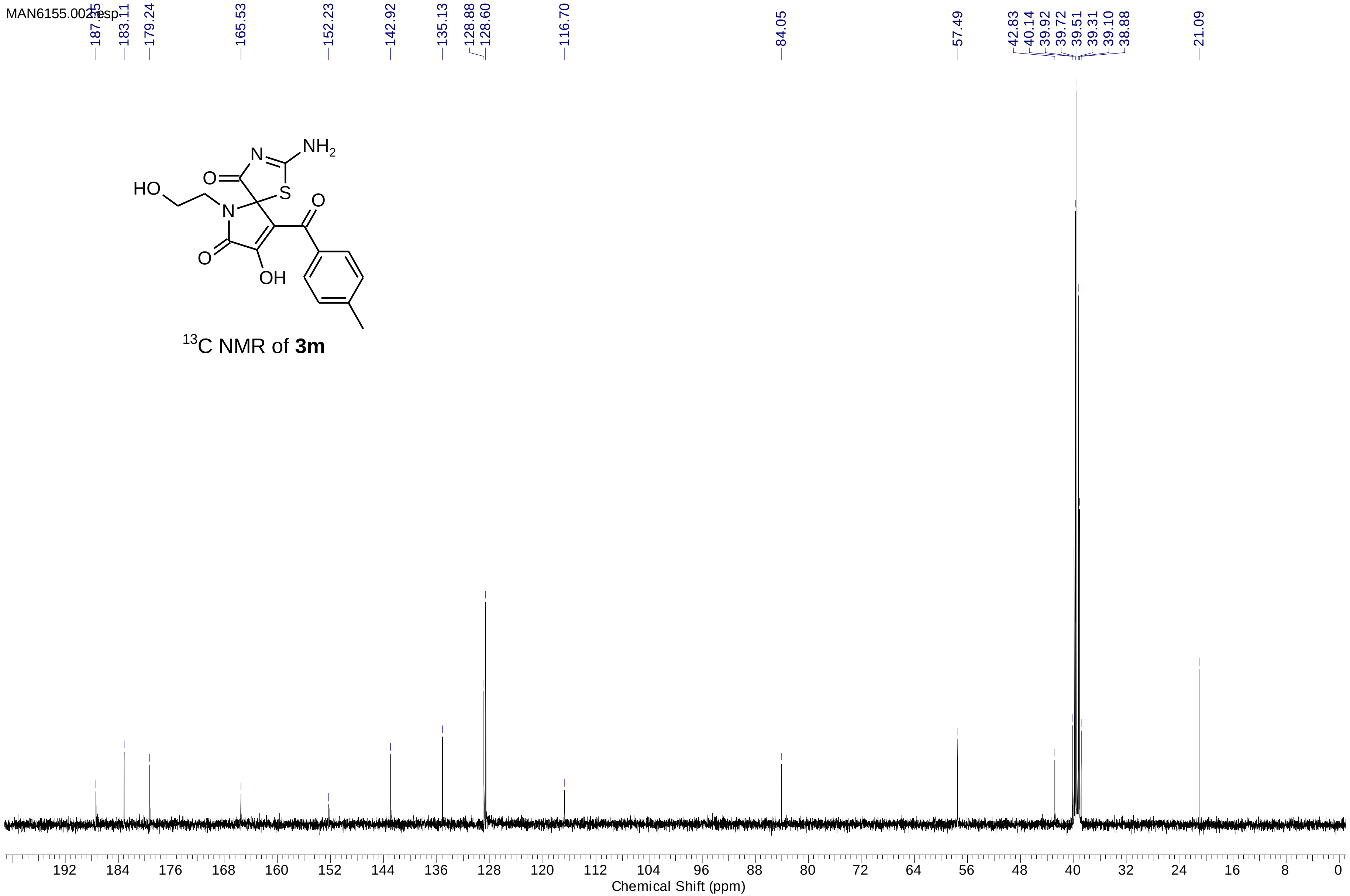
MAN6155.002.esp
187.55
183.11
179.24
165.53
152.23
142.92
135.13
128.88
128.60
116.70
84.05
57.49
42.83
40.14
39.92
39.72
39.51
39.31
39.10
38.88
21.09
NH2
N
O
HO
S
O
N
O
OH
Chemical Shift (ppm)

^{13}C NMR of **3m**

Pseudothiohydantoins 4a–c; General procedure

N-Acetylthiourea (1.5 mmol, 177 mg) was added to a suspension of FPD **1a,b,e** (1.5 mmol) in anhydrous ethyl acetate (10 mL). The mixture was stirred at room temperature for 12 h. The formed solid was filtered off and recrystallized from methanol to afford the desired pseudothiohydantoin **4a–c** (if the product still contains impurities after the recrystallization, it can be purified by stirring it in ethyl acetate (5 mL) for 12–24h, and the subsequent filtration of the solid gives the desired pseudothiohydantoin).

***N*-(9-Benzoyl-8-hydroxy-6-(2-hydroxyphenyl)-4,7-dioxo-1-thia-3,6-diazaspiro[4.4]nona-2,8-dien-2-yl)acetamide (4a)**

Yield: 524 mg (80%); yellow solid; mp 225–228 °C (decomp.).

^{1}H NMR (400 MHz, DMSO-d_6): δ = 12.69 (br.s, 1 H), 9.69 (br.s, 1 H), 7.77 (m, 2 H), 7.62 (m, 1 H), 7.52 (m, 2 H), 7.26 (m, 1 H), 6.96–6.89 (m, 2 H), 6.81 (m, 1 H), 2.11 (s, 3 H).

^{13}C NMR (100 MHz, DMSO-d_6): δ = 188.0, 179.5, 173.5, 165.1, 154.7, 153.4, 137.5, 132.8, 130.6, 128.9, 128.7 (2 C), 128.2 (2 C), 128.1, 120.4, 119.2, 117.0, 116.5, 79.6, 23.7.

IR (mineral oil): 3175, 1733, 1690 cm^{-1}.

MS (ESI+): m/z calcd for $C_{21}H_{15}N_3O_6S+H^+$: 438.08 [M+H$^+$]; found: 438.18.

Anal. Calcd (%) for $C_{21}H_{15}N_3O_6S$: C 57.66; H 3.46; N 9.61. Found: C 57.43; H 3.55; N 9.61.

***N*-(9-(4-Chlorobenzoyl)-8-hydroxy-6-(2-hydroxyphenyl)-4,7-dioxo-1-thia-3,6-diazaspiro[4.4]nona-2,8-dien-2-yl)acetamide (4b)**

Yield: 530 mg (75%); yellow solid; mp 184–186 °C (decomp.).

^{1}H NMR (400 MHz, DMSO-d_6): δ = 12.73 (br.s, 1 H), 9.66 (br.s, 1 H), 7.77 (m, 2 H), 7.59 (m, 2 H), 7.26 (m, 1 H), 6.96–6.89 (m, 2 H), 6.80 (m, 1 H), 2.11 (s, 3 H).

^{13}C NMR (100 MHz, DMSO-d_6): δ = 186.8, 179.5, 170.0, 164.9, 154.7, 153.9, 137.6, 136.2, 130.6 (3 C), 129.1, 128.9, 128.3 (2 C), 130.4, 119.2, 117.0, 116.2, 79.5, 23.6.

IR (mineral oil): 3124, 1717, 1667, 1645 cm^{-1}.

MS (ESI+): m/z calcd for $C_{21}H_{14}ClN_3O_6S+H^+$: 472.04 [M+H$^+$]; found: 472.08.

Anal. Calcd (%) for $C_{21}H_{14}ClN_3O_6S$: C 53.45; H 2.99; N 8.91. Found: C 53.47; H 3.14; N 9.00.

***N*-(9-Benzoyl-6-(5-chloro-2-hydroxyphenyl)-8-hydroxy-4,7-dioxo-1-thia-3,6-diazaspiro[4.4]nona-2,8-dien-2-yl)acetamide (4c)**

Yield: 558 mg (79%); yellow solid; mp 221–223 °C (decomp.).

^{1}H NMR (400 MHz, DMSO-d_6): δ = 12.83 (br.s, 1 H), 10.09 (br.s, 1 H), 7.77 (m, 2 H), 7.62 (m, 1 H), 7.52 (m, 2 H), 7.34 (m, 1 H), 6.98 (m, 1 H), 6.89 (m, 1 H), 2.13 (s, 3 H).
^{13}C NMR (100 MHz, DMSO-d_6): δ = 188.0, 179.7, 171.9, 165.0, 154.1, 153.1, 137.4, 132.8, 131.4, 130.6, 128.7 (2 C), 128.4, 128.2 (2 C), 121.9, 121.4, 118.6, 116.7, 79.5, 23.6.
IR (mineral oil): 3110, 1748, 1709, 1673 cm^{-1}.
MS (ESI+): m/z calcd for $C_{21}H_{14}ClN_3O_6S+H^+$: 472.04 [$M+H^+$]; found: 472.05.
Anal. Calcd (%) for $C_{21}H_{14}ClN_3O_6S$: C 53.45; H 2.99; N 8.91. Found: C 53.41; H 2.91; N 9.06.

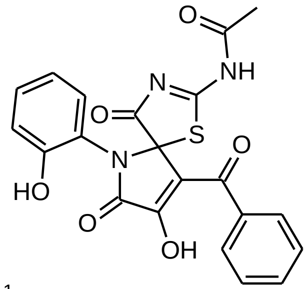
O
NH
N
O
S
O
N
HO
O
OH

^{1}H NMR of **4a**

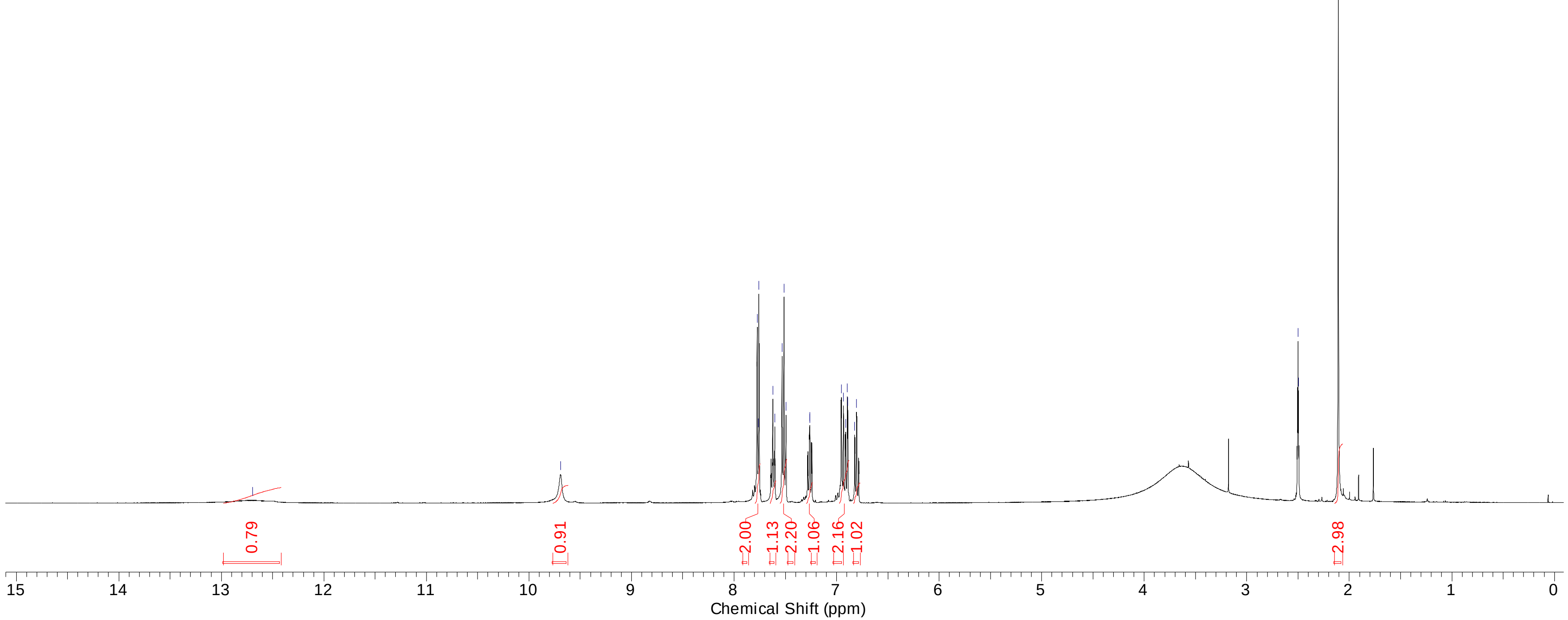
MAN5851.001.esp
12.69
9.69
7.77 7.76 7.62 7.60 7.53 7.51 7.49 7.26 6.95 6.94 6.89 6.89 6.81
2.50 2.50 2.11
0.79
0.91
2.00 1.13 2.20 1.06 2.16 1.02
2.98
15 14 13 12 11 10 9 8 7 6 5 4 3 2 1 0
Chemical Shift (ppm)

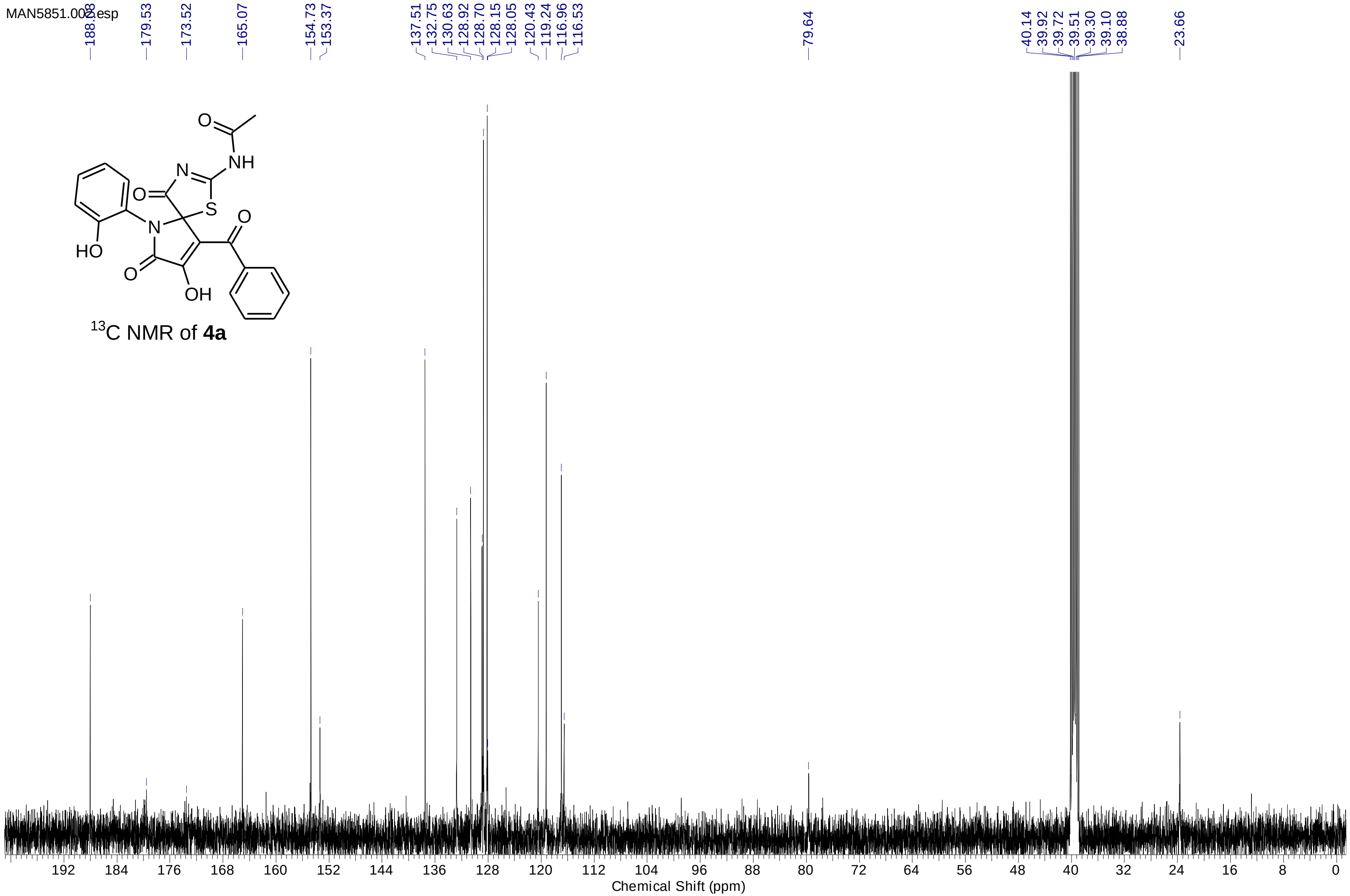
MAN5851.002.esp
188.03
179.53
173.52
165.07
154.73
153.37
137.51
132.75
130.63
128.92
128.70
128.15
128.05
120.43
119.24
116.96
116.53
79.64
40.14
39.92
39.72
39.51
39.30
39.10
38.88
23.66
O
NH
N
O
S
O
N
HO
O
OH
192 184 176 168 160 152 144 136 128 120 112 104 96 88 80 72 64 56 48 40 32 24 16 8 0
Chemical Shift (ppm)

^{13}C NMR of **4a**

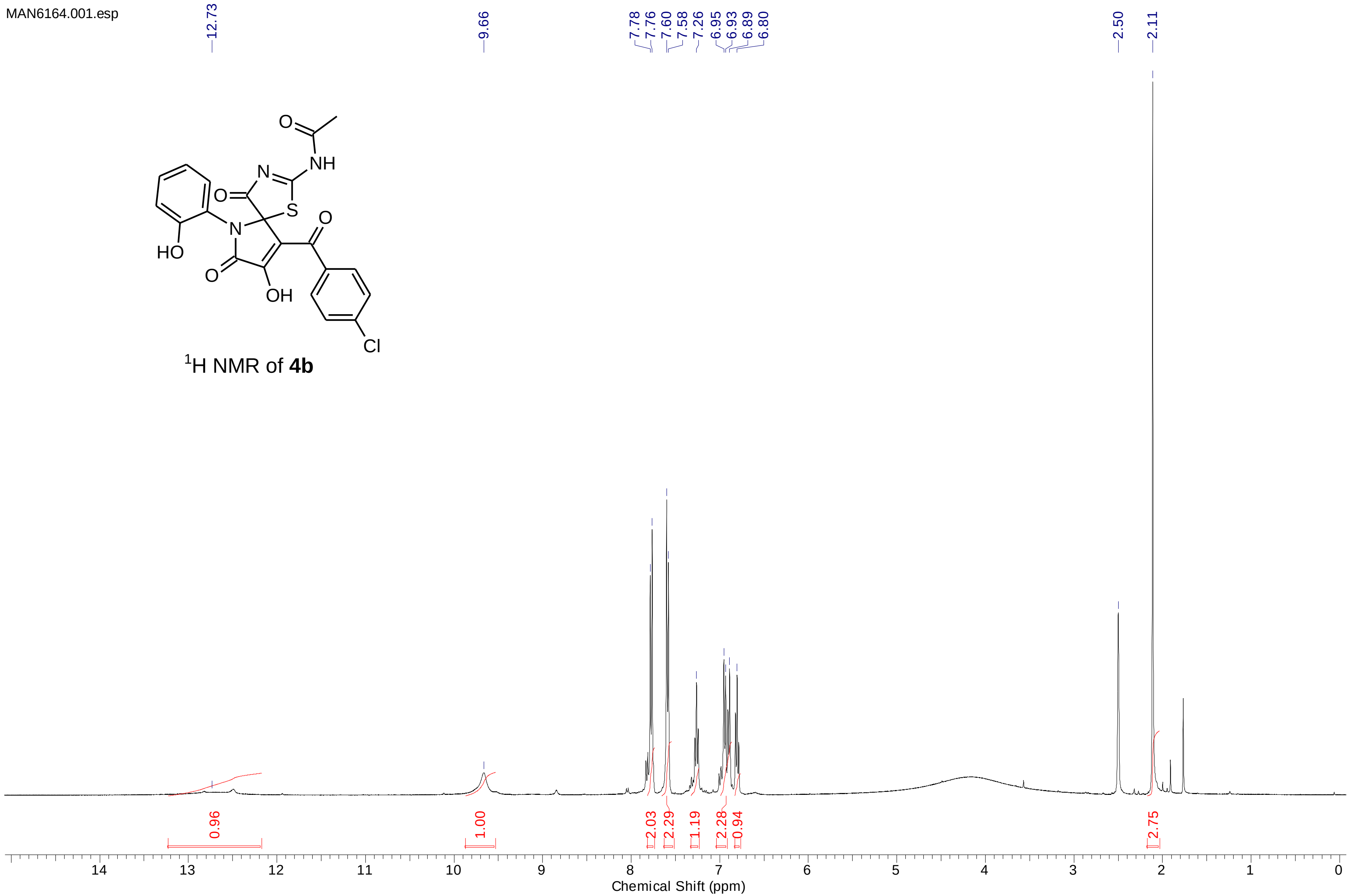
MAN6164.001.esp
12.73
9.66
7.78
7.76
7.60
7.58
7.26
6.95
6.93
6.89
6.80
2.50
2.11
O
NH
N
O
S
O
N
HO
O
OH
Cl
0.96
1.00
2.03
2.29
1.19
2.28
0.94
2.75
14
13
12
11
10
9
8
7
6
5
4
3
2
1
0
Chemical Shift (ppm)

^{1}H NMR of **4b**

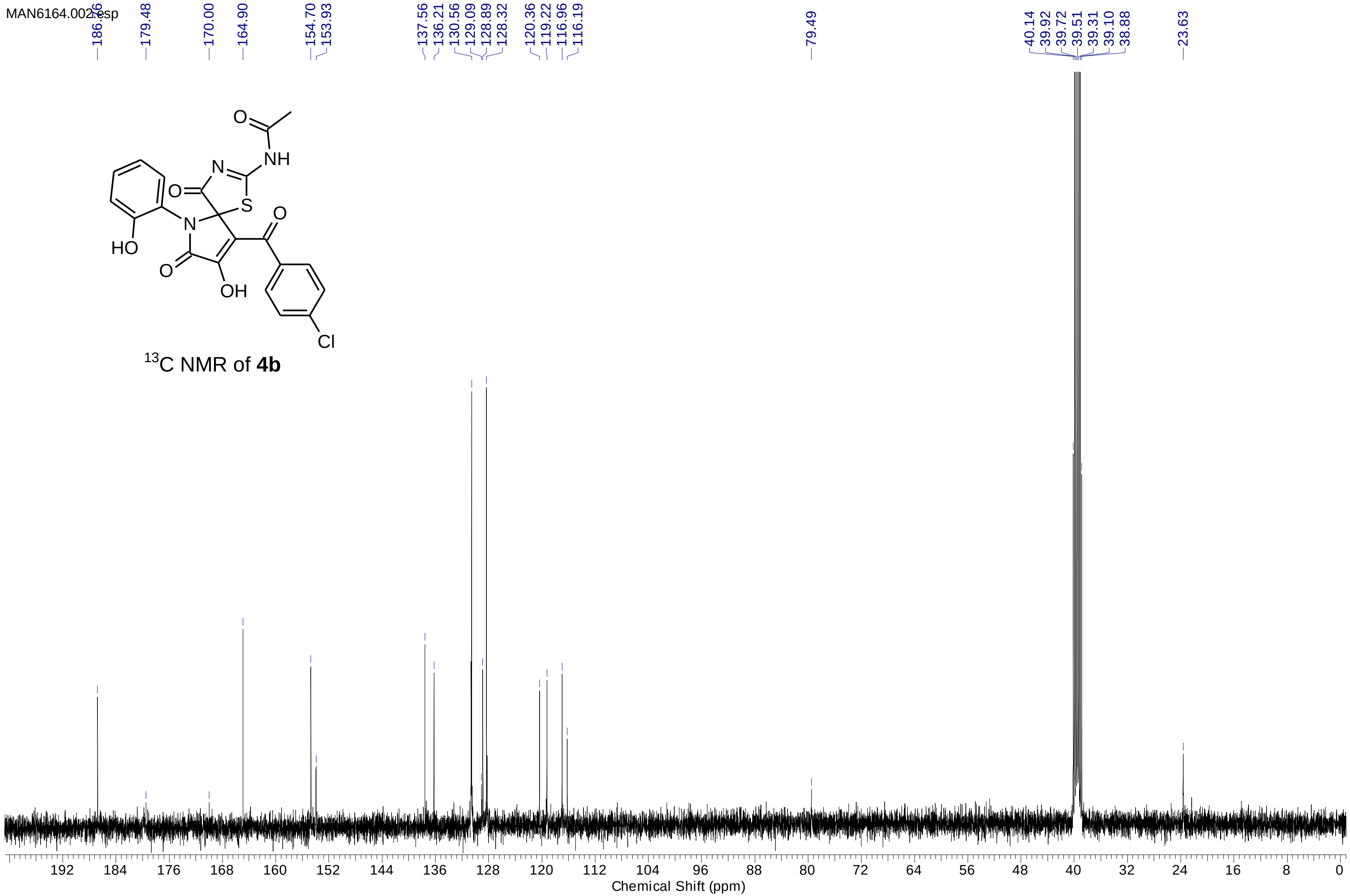
MAN6164.002.esp
186.76
179.48
170.00
164.90
154.70
153.93
137.56
136.21
130.56
129.09
128.89
128.32
120.36
119.22
116.96
116.19
79.49
40.14
39.92
39.72
39.51
39.31
39.10
38.88
23.63
O
NH
N
O
S
O
N
HO
O
OH
Cl
192
184
176
168
160
152
144
136
128
120
112
104
96
88
80
72
64
56
48
40
32
24
16
8
0
Chemical Shift (ppm)

^{13}C NMR of **4b**

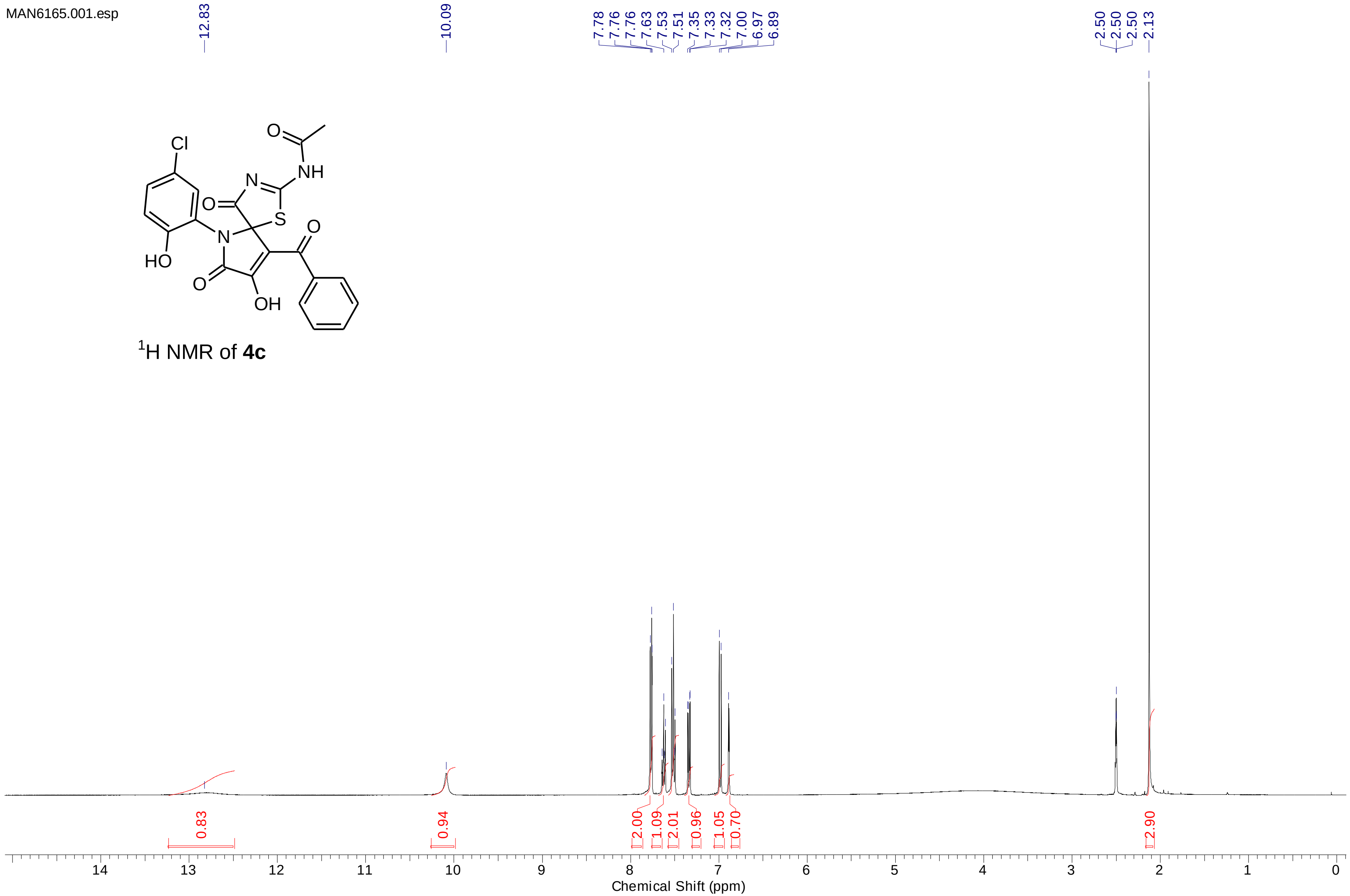
MAN6165.001.esp
12.83
10.09
7.78 7.76 7.76 7.63 7.53 7.51 7.35 7.33 7.32 7.00 6.97 6.89
2.50 2.50 2.50 2.13
Cl
O
N
NH
S
HO
OH
0.83
0.94
2.00 1.09 2.01 0.96 1.05 0.70
2.90
14 13 12 11 10 9 8 7 6 5 4 3 2 1 0
Chemical Shift (ppm)

^{1}H NMR of **4c**

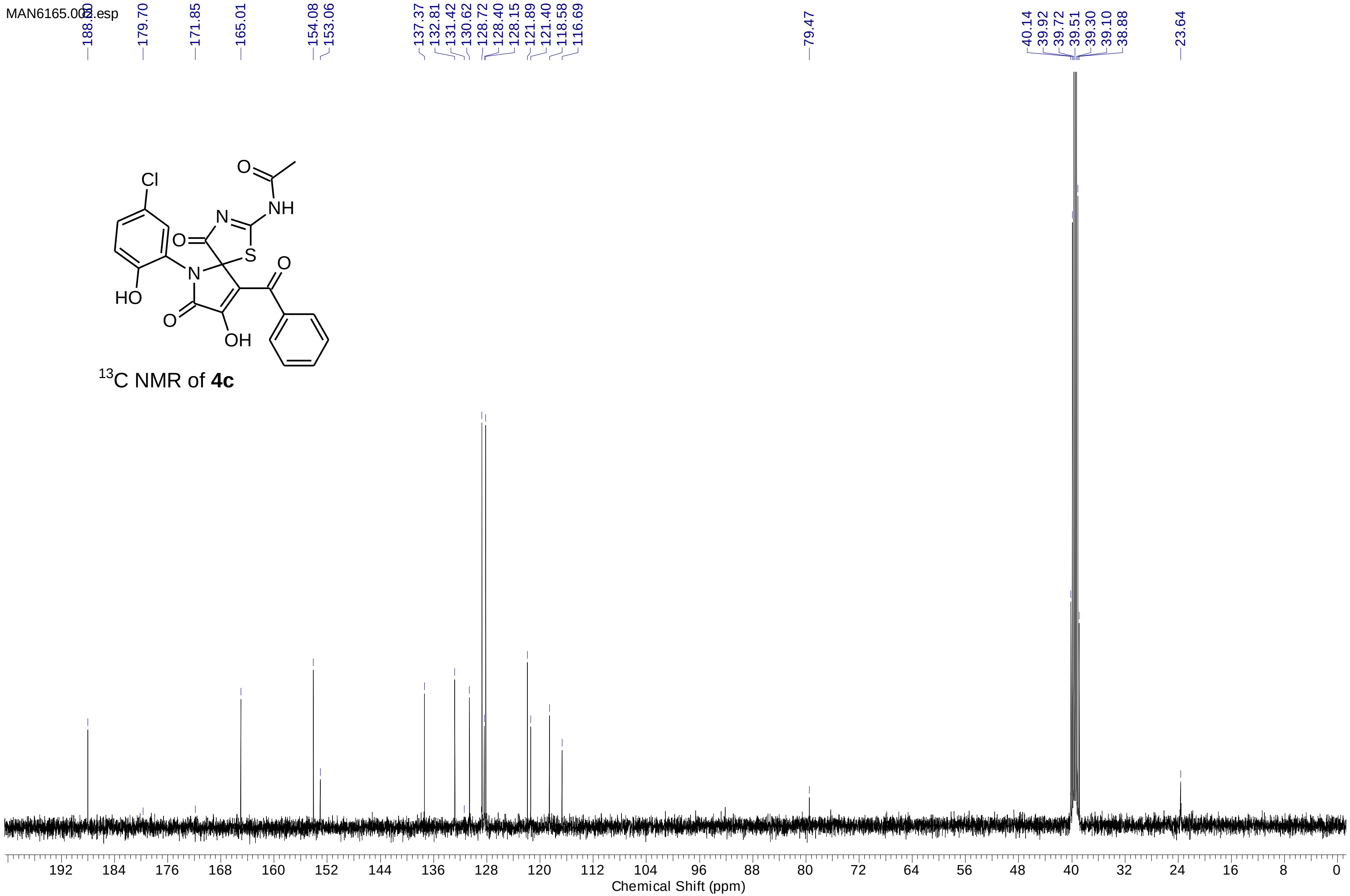
MAN6165.002.esp
188.00
179.70
171.85
165.01
154.08
153.06
137.37
132.81
131.42
130.62
128.72
128.40
128.15
121.89
121.40
118.58
116.69
79.47
40.14
39.92
39.72
39.51
39.30
39.10
38.88
23.64
Cl
O
NH
N
O
S
O
N
HO
O
OH
Chemical Shift (ppm)

^{13}C NMR of **4c**

Thiohydantoins 5a–c; General procedure

1,3-Dibutylthiourea (1.5 mmol, 282 mg) was added to a boiling solution of FPD **1a,b,l** (1.5 mmol) in anhydrous 1,4-dioxane (10 mL). The mixture was heated at reflux for 2–4 h, cooled to room temperature. The solvent was evaporated, and the resulting residue was triturated with toluene (15 mL). The formed solid was filtered off and recrystallized from toluene to afford the desired thiohydantoin **5a–c** (if the product still contains impurities after the recrystallization, it can be purified by an additional recrystallization from ethyl acetate (yields solvates), acetonitrile, methanol or toluene).

9-Benzoyl-1,3-dibutyl-8-hydroxy-6-(2-hydroxyphenyl)-2-thioxo-1,3,6-triazaspiro[4.4]non-8-ene-4,7-dione (5a)

Yield: 570 mg (75%); yellow solid; mp 135–137 °C (decomp.).

^{1}H NMR (400 MHz, DMSO-d_6): δ = 9.99 (br.s, 1 H), 7.81 (m, 2 H), 7.61 (m, 1 H), 7.51 (m, 2 H), 7.23 (m, 1 H), 6.95 (m, 1 H), 6.86 (m, 1 H), 6.78 (m, 1 H), 3.79–3.65 (m, 3 H), 3.53–3.45 (m, 1 H), 1.72–1.60 (m, 1 H), 1.48–1.40 (m, 2 H), 1.23–1.04 (m, 5 H), 0.83 (t, *J* = 7.3 Hz, 3 H), 0.68 (t, *J* = 7.3 Hz, 3 H).

^{13}C NMR (100 MHz, DMSO-d_6): δ = 187.3, 182.3, 169.5, 164.1, 156.4, 154.2, 137.1, 132.6, 130.4, 128.8 (2 C), 128.4, 128.0 (2 C), 119.5, 119.1, 116.8, 111.7, 81.2, 43.2, 41.2, 28.9, 28.6, 19.5, 19.1, 13.5, 13.3.

IR (mineral oil): 3172, 1747, 1723, 1678 cm^{-1}.

MS (ESI+): m/z calcd for $C_{27}H_{29}N_3O_5S$+H^+: 508.19 [M+H^+]; found: 508.16.

Anal. Calcd (%) for $C_{27}H_{29}N_3O_5S$: C 63.89; H 5.76; N 8.28. Found: C 63.97; H 5.66; N 8.18.

9-Benzoyl-1,3-dibutyl-6-(5-chloro-2-hydroxyphenyl)-8-hydroxy-2-thioxo-1,3,6-triazaspiro[4.4]non-8-ene-4,7-dione (5b)

Yield: 617 mg (76%); yellow solid; mp 235–237 °C (decomp.).

^{1}H NMR (400 MHz, DMSO-d_6): δ = 10.40 (br.s, 1 H), 7.81 (m, 2 H), 7.61 (m, 1 H), 7.50 (m, 2 H), 7.31 (m, 1 H), 6.98 (m, 1 H), 6.86 (m, 1 H), 3.83–3.63 (m, 3 H), 3.53–3.46 (m, 1 H), 1.68–1.58 (m, 1 H), 1.49–1.36 (m, 2 H), 1.23–1.04 (m, 5 H), 0.84 (t, *J* = 7.3 Hz, 3 H), 0.67 (t, *J* = 7.3 Hz, 3 H).

^{13}C NMR (100 MHz, DMSO-d_6): δ = 187.3, 182.2, 169.4, 164.1, 156.2, 153.6, 137.0, 132.7, 130.3, 128.8 (2 C), 128.1, 128.0 (2 C), 122.0, 120.6, 118.3, 111.8, 81.2, 43.3, 41.2, 29.2, 28.6, 19.5, 19.1, 13.5, 13.3.

IR (mineral oil): 3324, 1727, 1676 cm^{-1}.

MS (ESI+): m/z calcd for $C_{27}H_{28}ClN_3O_5S$+H^+: 542.15 [M+H^+]; found: 542.25.

Anal. Calcd (%) for $C_{27}H_{28}ClN_3O_5S$: C 59.83; H 5.21; N 7.75. Found: C 59.91; H 5.20; N 7.56.

1,3-Dibutyl-9-(4-chlorobenzoyl)-8-hydroxy-6-(2-hydroxyethyl)-2-thioxo-1,3,6-triazaspiro[4.4]non-8-ene-4,7-dione (5c)

Yield: 562 mg (76%); yellow solid; mp 81–83 °C (decomp.).

^{1}H NMR (400 MHz, DMSO-d_6): δ = 7.78 (m, 2 H), 7.53 (m, 2 H), 3.90–3.78 (m, 2 H), 3.68–3.60 (m, 1 H), 3.52–3.45 (m, 1 H), 3.38–3.31 (m, 1 H), 3.28–3.20 (m, 1 H), 3.13–3.07 (m, 2 H), 1.72–1.65 (m, 2 H), 1.62–1.53 (m, 1 H), 1.43–1.34 (m, 2 H), 1.24–1.12 (m, 3 H), 0.94 (t, *J* = 7.3 Hz, 3 H), 0.70 (t, *J* = 7.3 Hz, 3 H).

^{13}C NMR (100 MHz, DMSO-d_6): δ = 185.6, 182.6, 169.6, 165.5, 157.6, 137.2, 135.9, 130.6 (2 C), 128.1 (2 C), 110.7, 80.0, 57.5, 42.7, 42.5, 41.4, 29.1 (2 C), 19.4, 19.3, 13.5, 13.4.

IR (mineral oil): 3178, 1722, 1669 cm^{-1}.

MS (ESI+): m/z calcd for $C_{23}H_{28}ClN_3O_5S+H^+$: 494.15 [M+H$^+$]; found: 494.14.

Anal. Calcd (%) for $C_{23}H_{28}ClN_3O_5S$: C 55.92; H 5.71; N 8.51. Found: C 55.73; H 5.76; N 8.39.

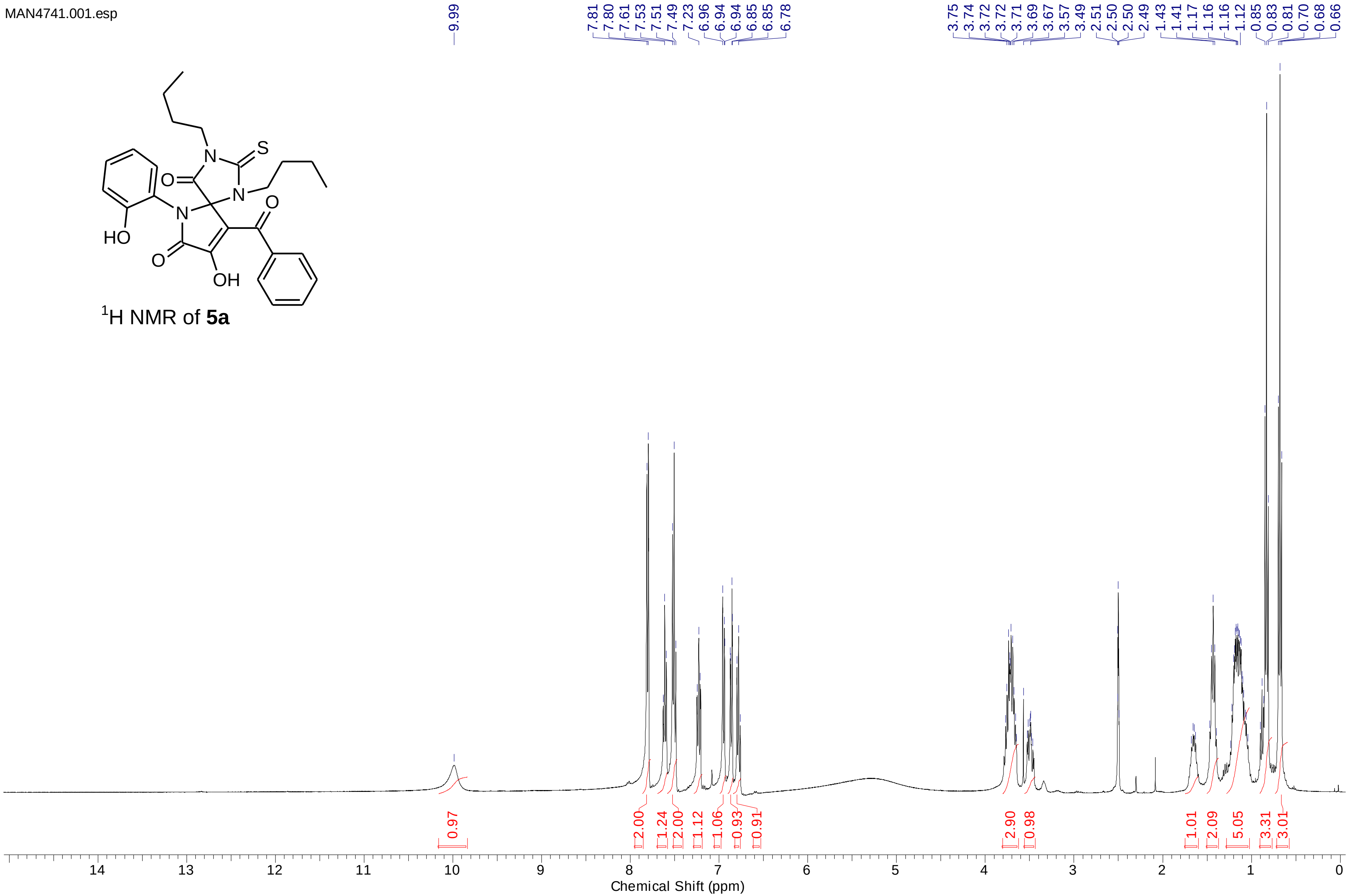

MAN4741.001.esp
9.99
7.81 7.80 7.61 7.53 7.51 7.49 7.23 6.96 6.94 6.94 6.85 6.85 6.78
3.75 3.74 3.72 3.72 3.71 3.69 3.67 3.57 3.49 2.51 2.50 2.50 2.49 1.43 1.41 1.17 1.16 1.16 1.12 0.85 0.83 0.81 0.70 0.68 0.66
0.97
2.00 1.24 2.00 1.12 1.06 0.93 0.91
2.90 0.98
1.01 2.09 5.05 3.31 3.01
14 13 12 11 10 9 8 7 6 5 4 3 2 1 0
Chemical Shift (ppm)

^{1}H NMR of **5a**

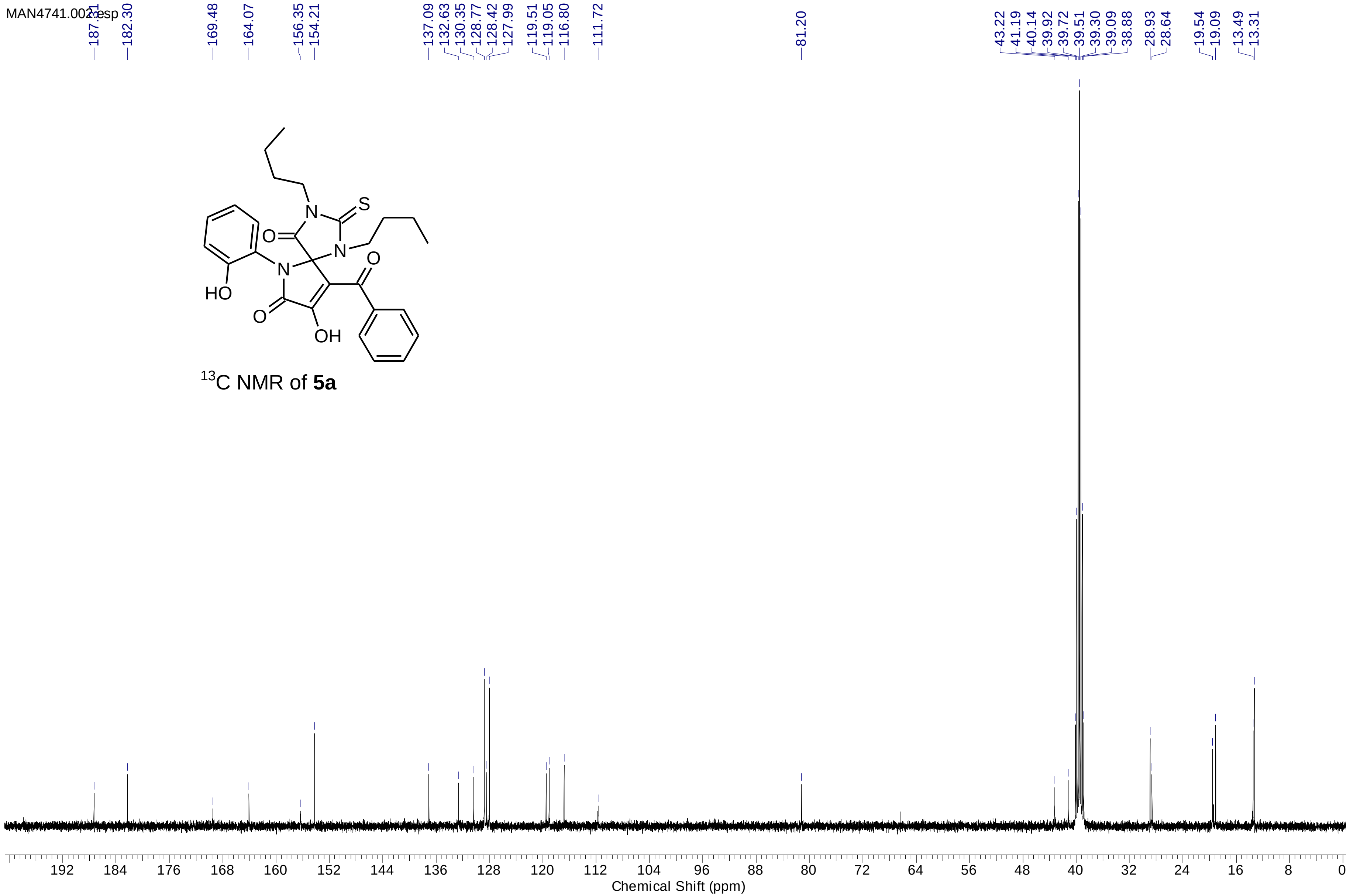
MAN4741.002.esp
187.[illegible]
182.30
169.48
164.07
156.35
154.21
137.09
132.63
130.35
128.77
128.42
127.99
119.51
119.05
116.80
111.72
81.20
43.22
41.19
40.14
39.92
39.72
39.51
39.30
39.09
38.88
28.93
28.64
19.54
19.09
13.49
13.31
192 184 176 168 160 152 144 136 128 120 112 104 96 88 80 72 64 56 48 40 32 24 16 8 0
Chemical Shift (ppm)

^{13}C NMR of **5a**

MAN6167.001.esp

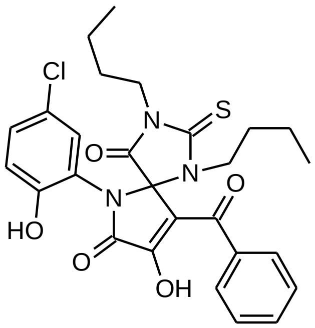

^{1}H NMR of **5b**

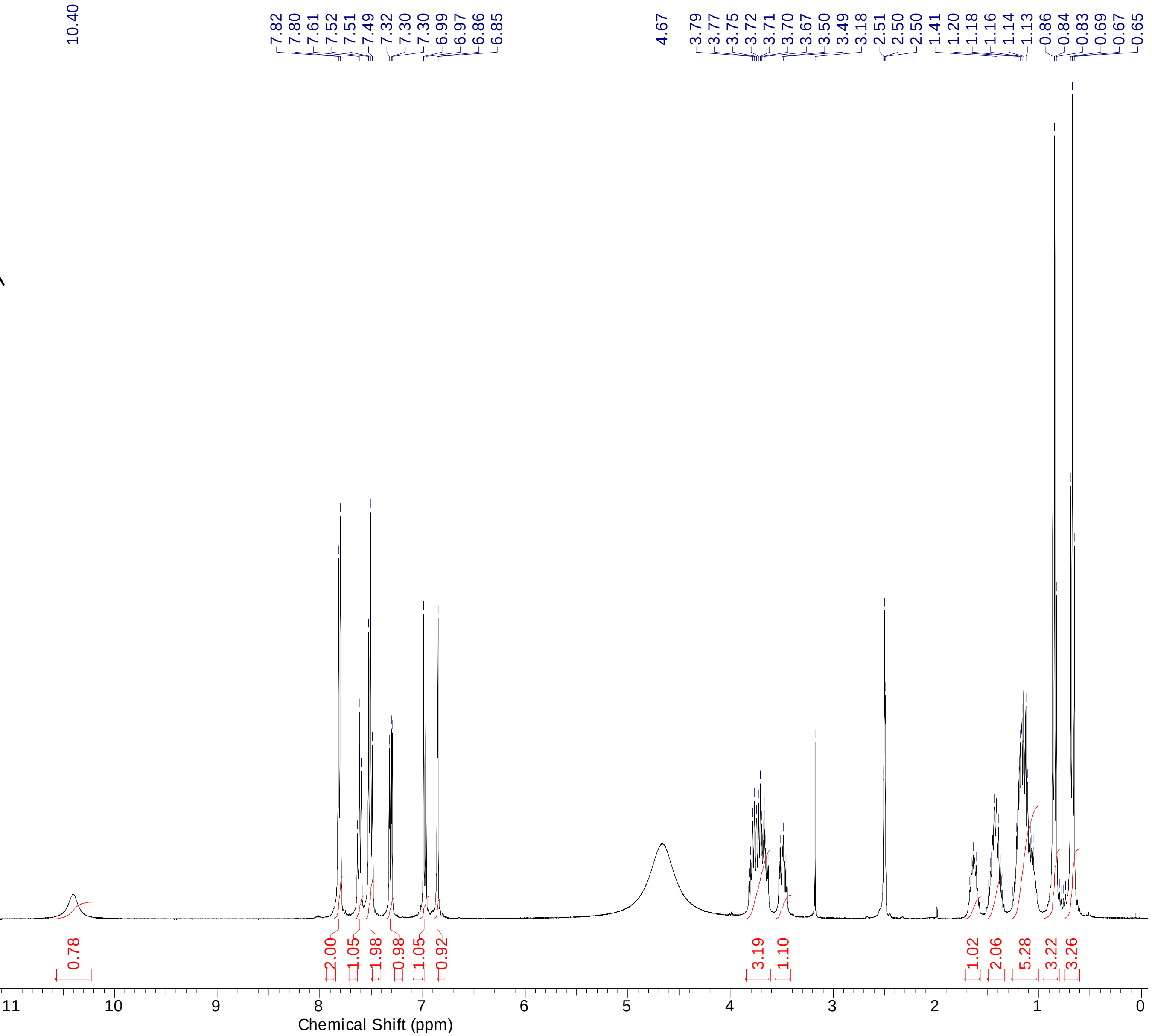

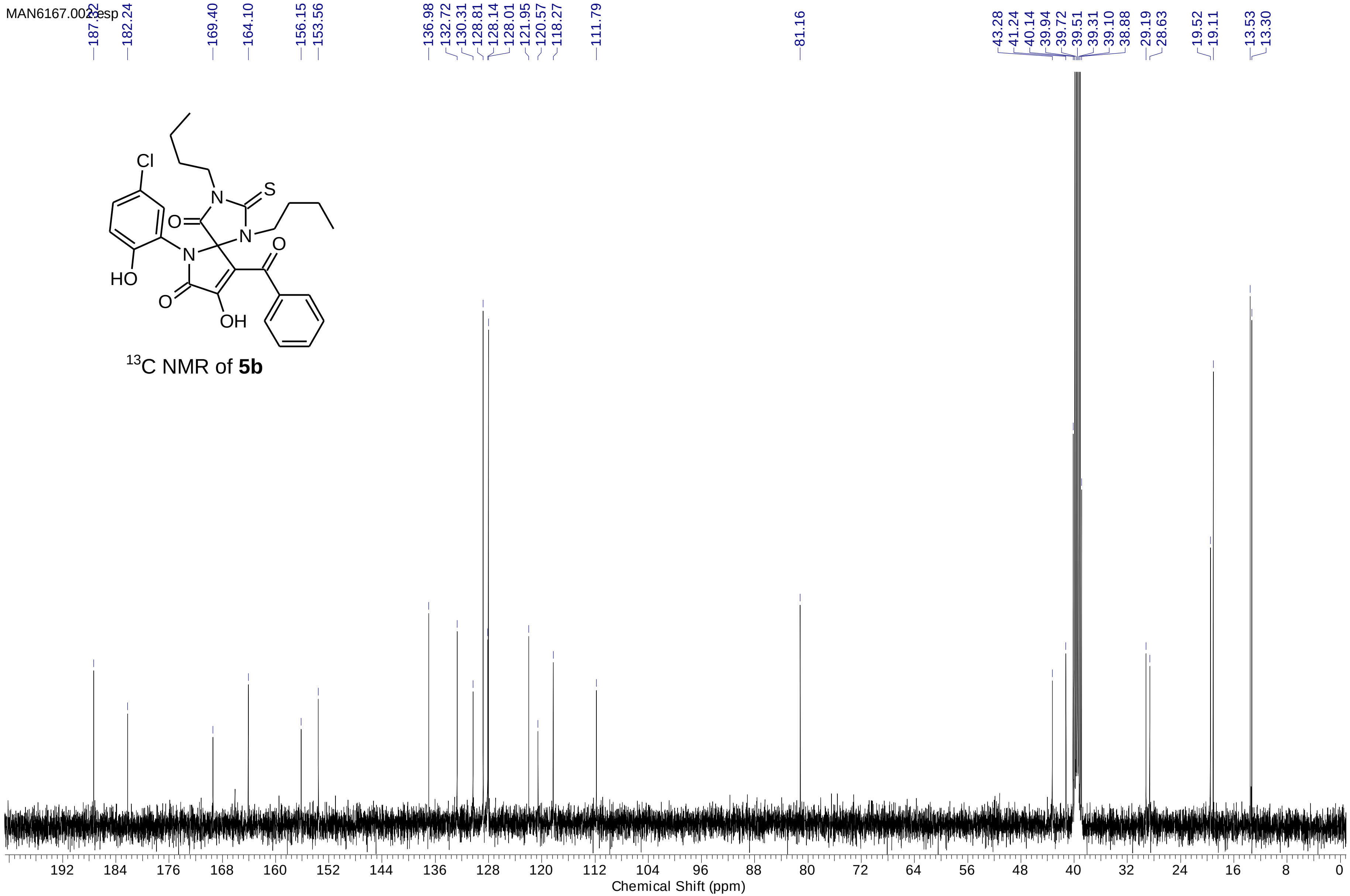

MAN6167.002.esp
187.22
182.24
169.40
164.10
156.15
153.56
136.98
132.72
130.31
128.81
128.14
128.01
121.95
120.57
118.27
111.79
81.16
43.28
41.24
40.14
39.94
39.72
39.51
39.31
39.10
38.88
29.19
28.63
19.52
19.11
13.53
13.30
Cl
N
S
O
N
N
O
HO
O
OH
^{13}C NMR of **5b**
192
184
176
168
160
152
144
136
128
120
112
104
96
88
80
72
64
56
48
40
32
24
16
8
0
Chemical Shift (ppm)

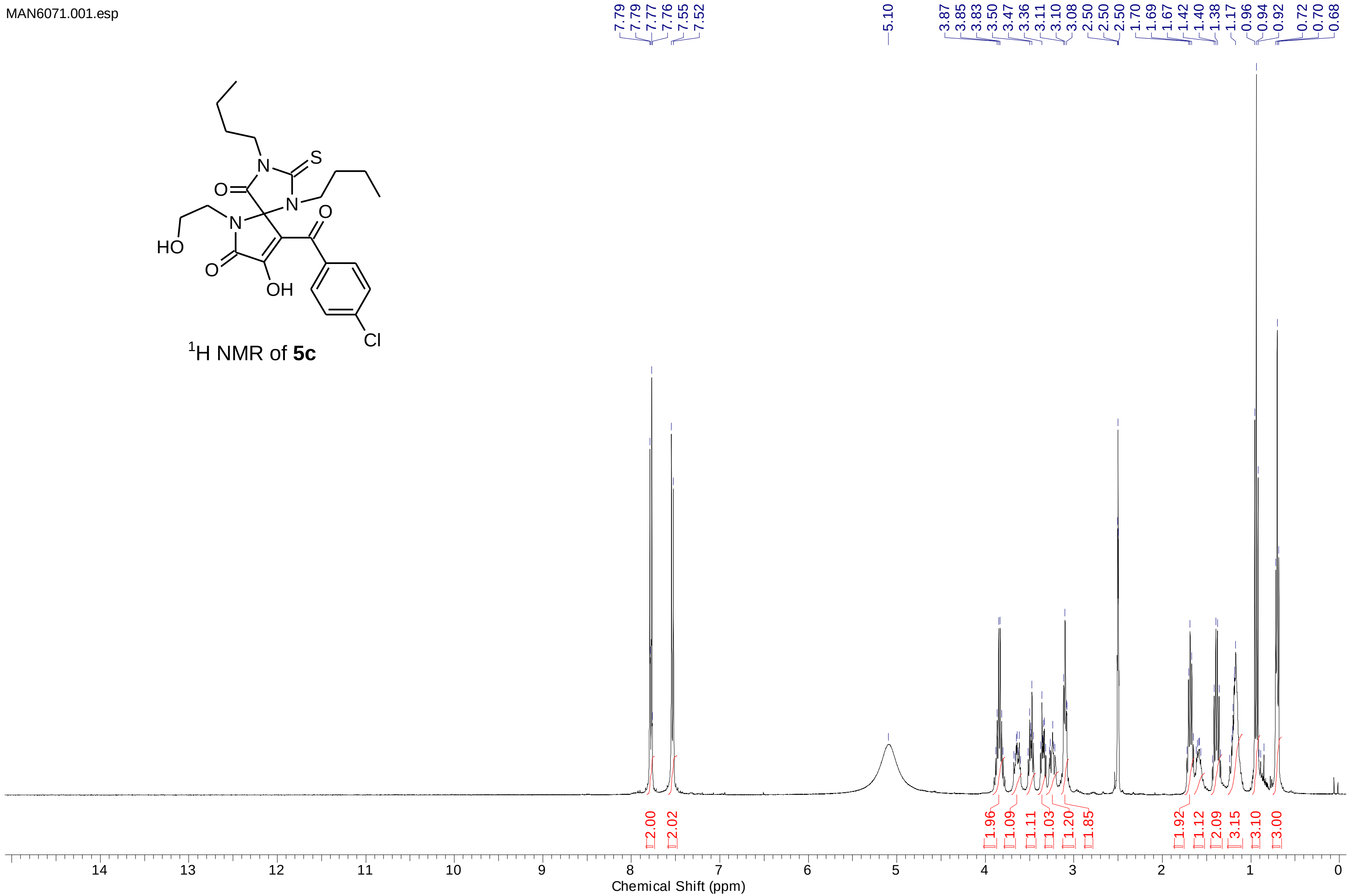
MAN6071.001.esp
7.79 7.79 7.77 7.76 7.55 7.52
5.10
3.87 3.85 3.83 3.50 3.47 3.36 3.11 3.10 3.08 2.50 2.50 2.50 1.70 1.69 1.67 1.42 1.40 1.38 1.17 0.96 0.94 0.92 0.72 0.70 0.68
N
S
O
N
HO
N
O
O
OH
Cl
2.00 2.02
1.96 1.09 1.11 1.03 1.20 1.85
1.92 1.12 2.09 3.15 3.10 3.00
14 13 12 11 10 9 8 7 6 5 4 3 2 1 0
Chemical Shift (ppm)

^{1}H NMR of **5c**

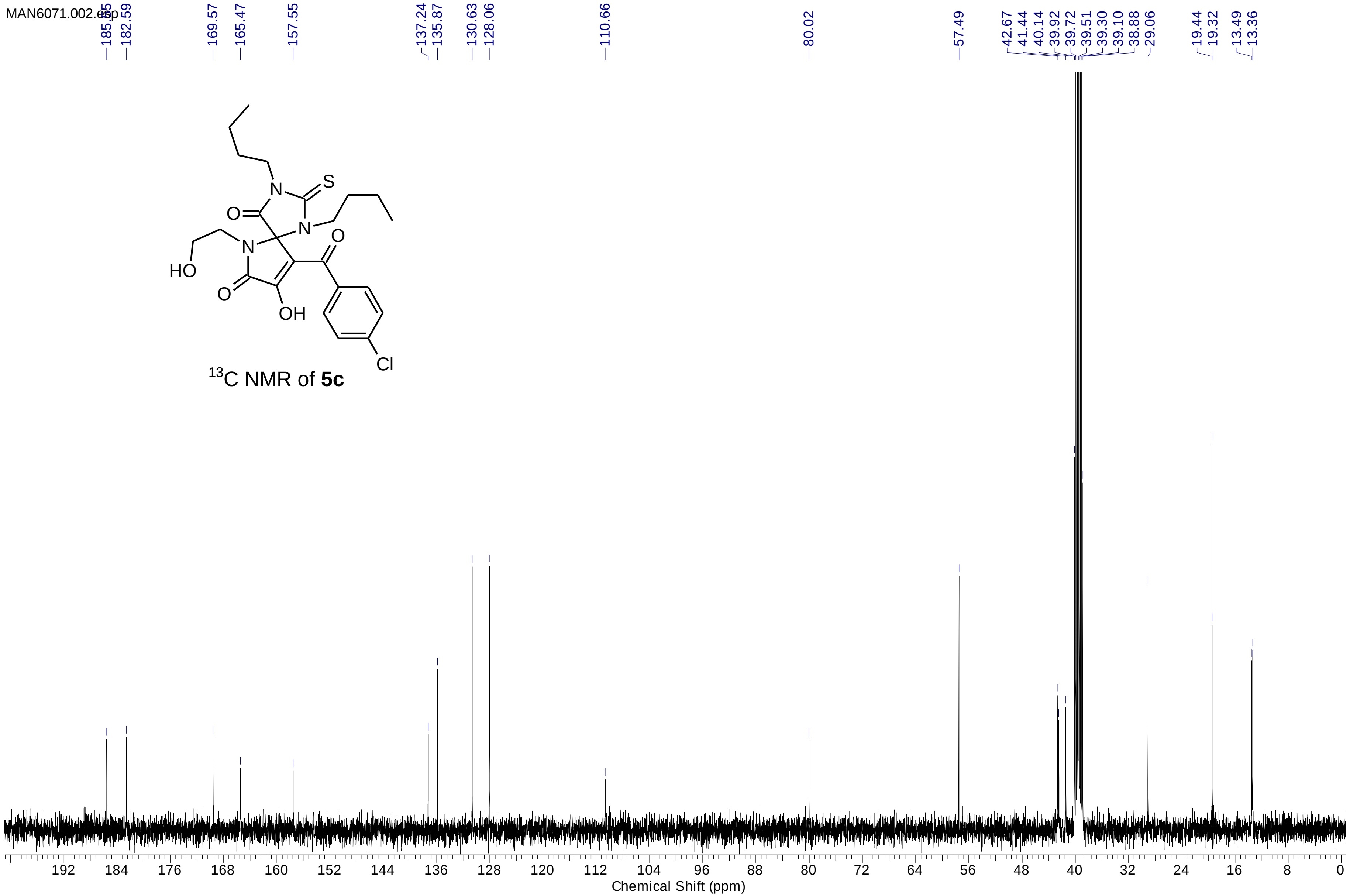
MAN6071.002.esp
185.85
182.59
169.57
165.47
157.55
137.24
135.87
130.63
128.06
110.66
80.02
57.49
42.67
41.44
40.14
39.92
39.72
39.51
39.30
39.10
38.88
29.06
19.44
19.32
13.49
13.36
O
N
S
N
O
N
HO
O
OH
Cl
192 184 176 168 160 152 144 136 128 120 112 104 96 88 80 72 64 56 48 40 32 24 16 8 0
Chemical Shift (ppm)

^{13}C NMR of **5c**

Thiohydantoins 7a–c; General procedure

1,3-Diphenylthiourea (1.5 mmol, 342 mg) was added to a boiling solution of FPD **1a,l,m** (1.5 mmol) in anhydrous 1,4-dioxane (10 mL). The mixture was heated at reflux for 20 h, cooled to room temperature. The solvent was evaporated, and the resulting residue was triturated with toluene (15 mL). The formed solid was filtered off and recrystallized from toluene to afford the desired thiohydantoin **7a–c** (if the product still contains impurities after the recrystallization, it can be purified by an additional recrystallization from ethyl acetate (yields solvates), acetonitrile, methanol or toluene).

9-Benzoyl-8-hydroxy-6-(2-hydroxyphenyl)-1,3-diphenyl-2-thioxo-1,3,6-triazaspiro[4.4]non-8-ene-4,7-dione (7a)

Yield: 583 mg (71%); yellow solid; mp 139-141 °C (decomp.).

^{1}H NMR (400 MHz, DMSO-d_6): δ = 10.16 (br.s, 1 H), 7.65–6.87 (m, 19 H).

^{13}C NMR (100 MHz, DMSO-d_6): δ = 188.0, 183.2, 168.4, 163.9, 156.8, 154.0, 137.4, 136.1, 133.7, 132.5, 130.3, 129.2 (3 C), 128.9 (2 C), 128.5, 128.4 (2 C), 128.3 (2 C), 128.0 (2 C), 127.7 (2 C), 127.2, 120.6, 119.2, 117.4, 112.0, 83.3.

IR (mineral oil): 3322, 1741, 1673 cm^{-1}.

MS (ESI+): m/z calcd for $C_{31}H_{21}N_3O_5S+H^+$: 548.13 [M+H$^+$]; found: 548.11.

Anal. Calcd (%) for $C_{31}H_{21}N_3O_5S$: C 68.00; H 3.87; N 7.67. Found: C 68.34; H 3.77; N 7.69.

9-(4-Chlorobenzoyl)-8-hydroxy-6-(2-hydroxyethyl)-1,3-diphenyl-2-thioxo-1,3,6-triazaspiro[4.4]non-8-ene-4,7-dione (7b)

Yield: 505 mg (63%); yellow solid; mp 239–241 °C (decomp.).

^{1}H NMR (400 MHz, DMSO-d_6): δ = 7.66–7.34 (m, 12 H), 7.16 (m, 2 H), 3.80 (m, 1 H), 3.71 (m, 1 H), 3.58 (m, 1 H), 3.49 (m, 1 H).

^{13}C NMR (100 MHz, DMSO-d_6): δ = 186.1, 183.3, 168.6, 165.7, 158.4, 136.9, 136.2, 135.3, 133.8, 130.2 (2 C), 129.3 (2 C), 129.1, 129.0 (2 C), 128.9, 128.6 (2 C), 128.0 (2 C), 127.8 (2 C), 110.7, 82.3, 58.0, 43.4.

IR (mineral oil): 3300, 1742, 1722, 1672 cm^{-1}.

MS (ESI+): m/z calcd for $C_{27}H_{20}ClN_3O_5S+H^+$: 534.09 [M+H$^+$]; found: 534.19.

Anal. Calcd (%) for $C_{27}H_{20}ClN_3O_5S$: C 60.73; H 3.78; N 7.87. Found: C 60.52; H 3.78; N 7.93.

8-Hydroxy-6-(2-hydroxyethyl)-9-(4-methylbenzoyl)-1,3-diphenyl-2-thioxo-1,3,6-triazaspiro[4.4]non-8-ene-4,7-dione (7c)

Yield: 555 mg (72%); yellow solid; mp 245–251 °C (decomp.).

^{1}H NMR (400 MHz, DMSO-d_6): δ = 7.63–7.52 (m, 5 H), 7.40 (m, 3 H), 7.32 (m, 2 H), 7.24–7.15 (m, 4 H), 3.80 (m, 1 H), 3.73 (m, 1 H), 3.60 (m, 1 H), 3.52 (m, 1 H), 2.35 (s, 3 H).
^{13}C NMR (100 MHz, DMSO-d_6): δ = 187.6, 183.4, 168.4, 165.4, 155.9, 143.0, 135.3, 134.5, 133.8, 129.3 (2 C), 129.2, 129.0 (2 C), 128.9, 128.6 (4 C), 128.5 (2 C), 127.8 (2 C), 112.3, 82.2, 58.0, 43.5, 21.1.
IR (mineral oil): 3177, 1757, 1704, 1681 cm^{-1}.
MS (ESI+): m/z calcd for $C_{28}H_{23}N_3O_5S+H^+$: 514.14 [M+H$^+$]; found: 514.14.
Anal. Calcd (%) for $C_{28}H_{23}N_3O_5S$: C 65.48; H 4.51; N 8.18. Found: C 65.79; H 4.56; N 7.98.

MAN4821.001.esp

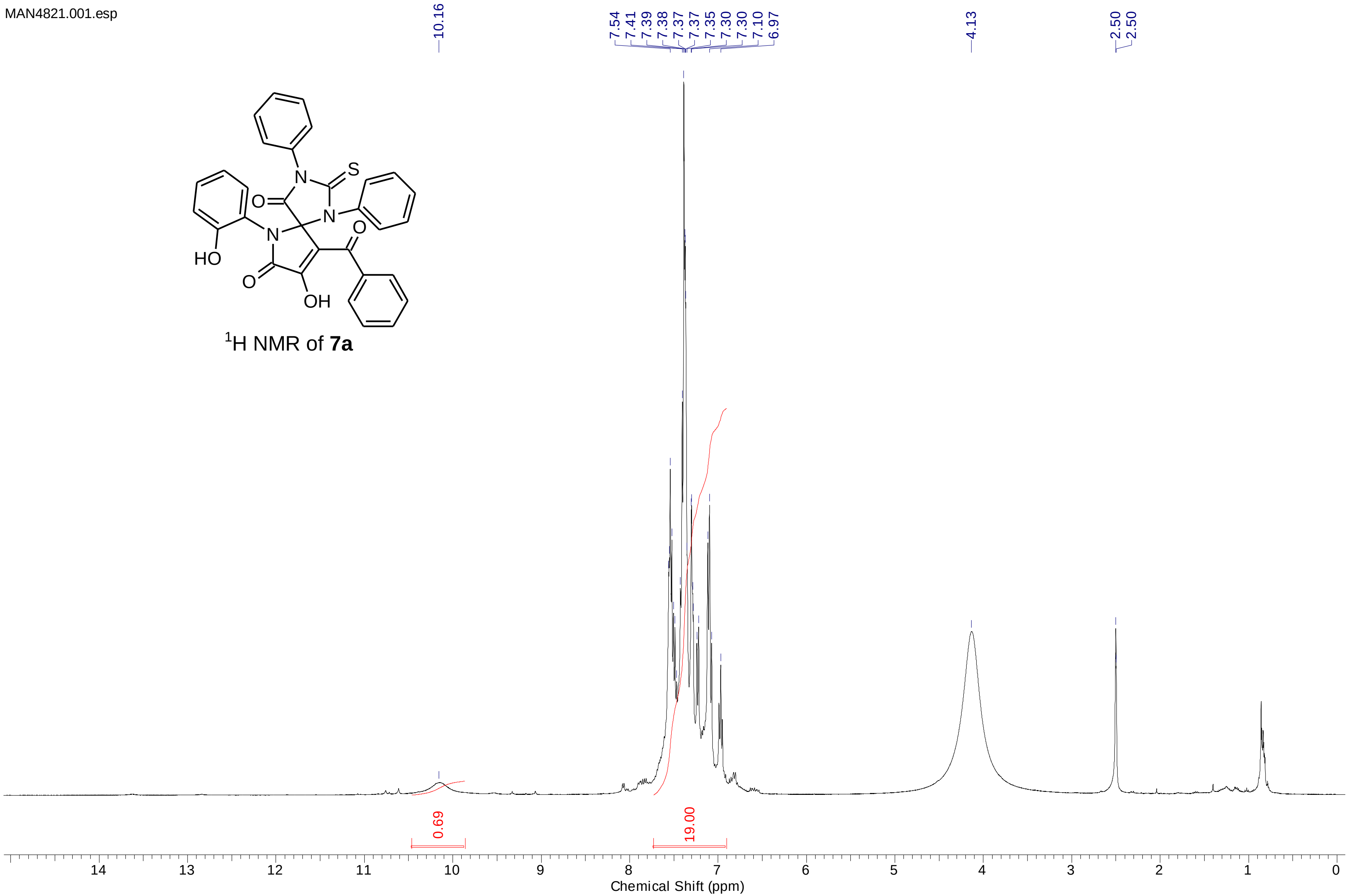

^{1}H NMR of **7a**

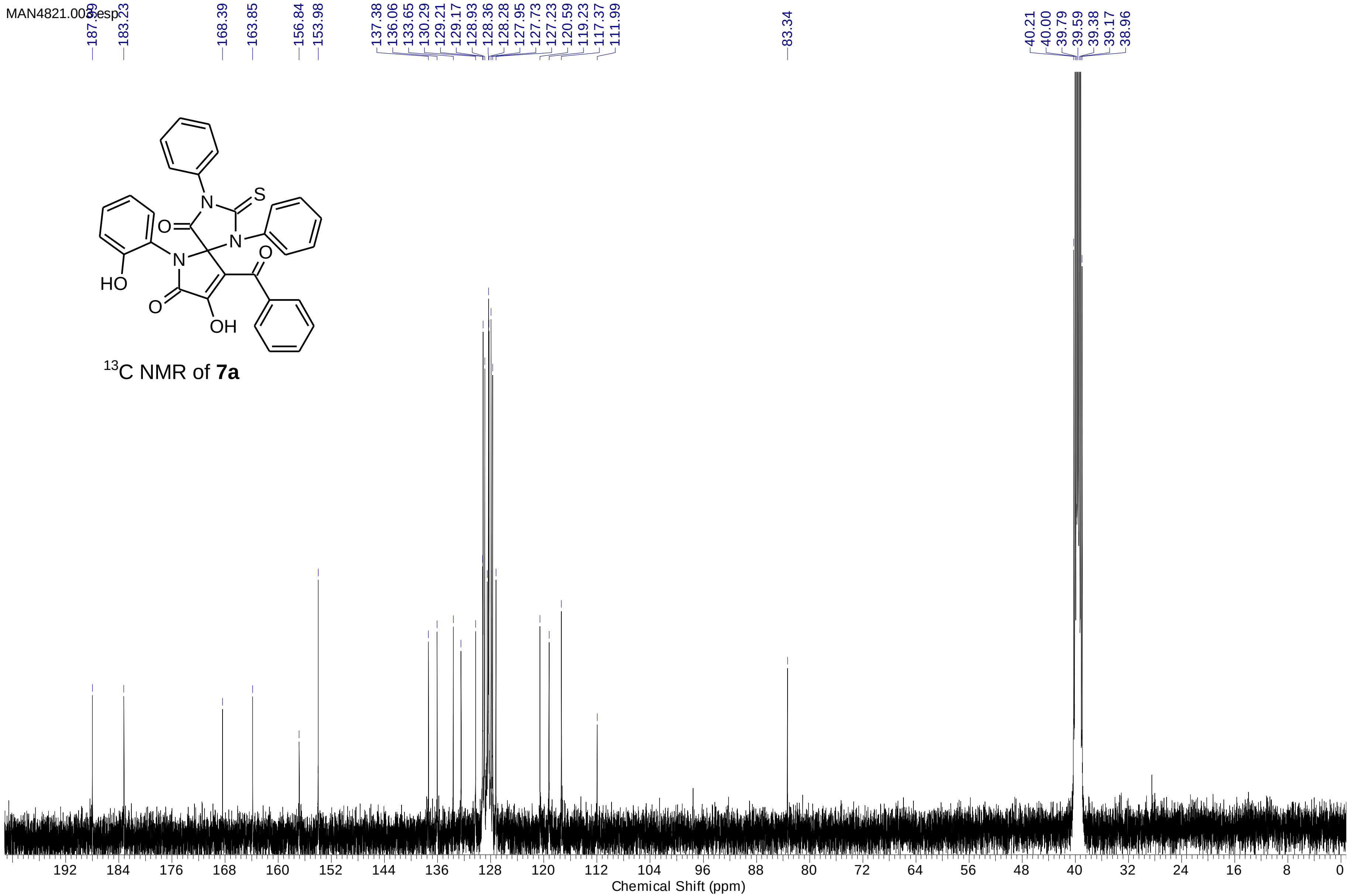
MAN4821.003.esp
187.99
183.23
168.39
163.85
156.84
153.98
137.38
136.06
133.65
130.29
129.21
129.17
128.93
128.36
128.28
127.95
127.73
127.23
120.59
119.23
117.37
111.99
83.34
40.21
40.00
39.79
39.59
39.38
39.17
38.96
O
N
S
N
N
HO
O
O
OH
192
184
176
168
160
152
144
136
128
120
112
104
96
88
80
72
64
56
48
40
32
24
16
8
0
Chemical Shift (ppm)

^{13}C NMR of **7a**

MAN6043.001.esp

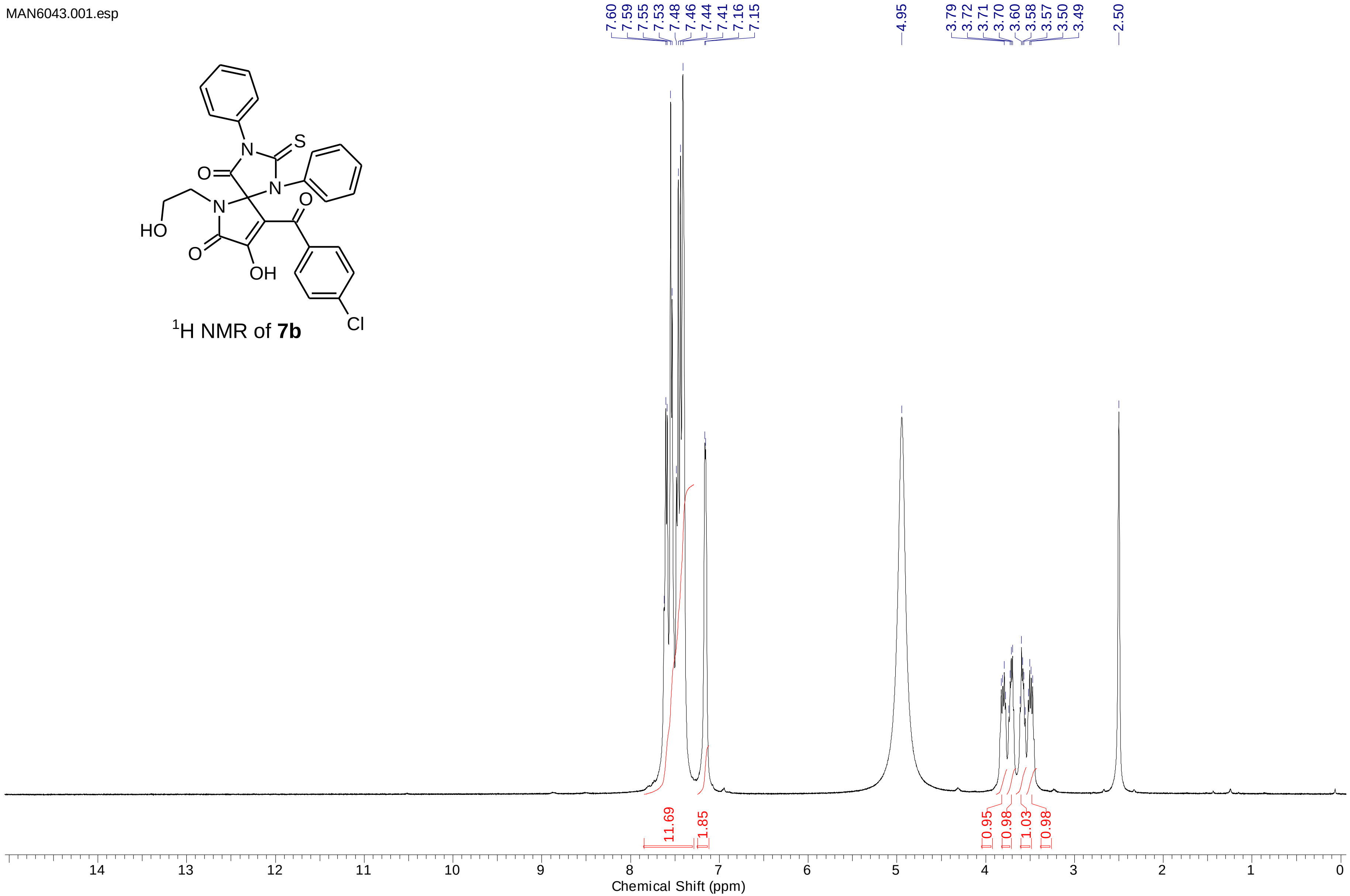

^{1}H NMR of **7b**

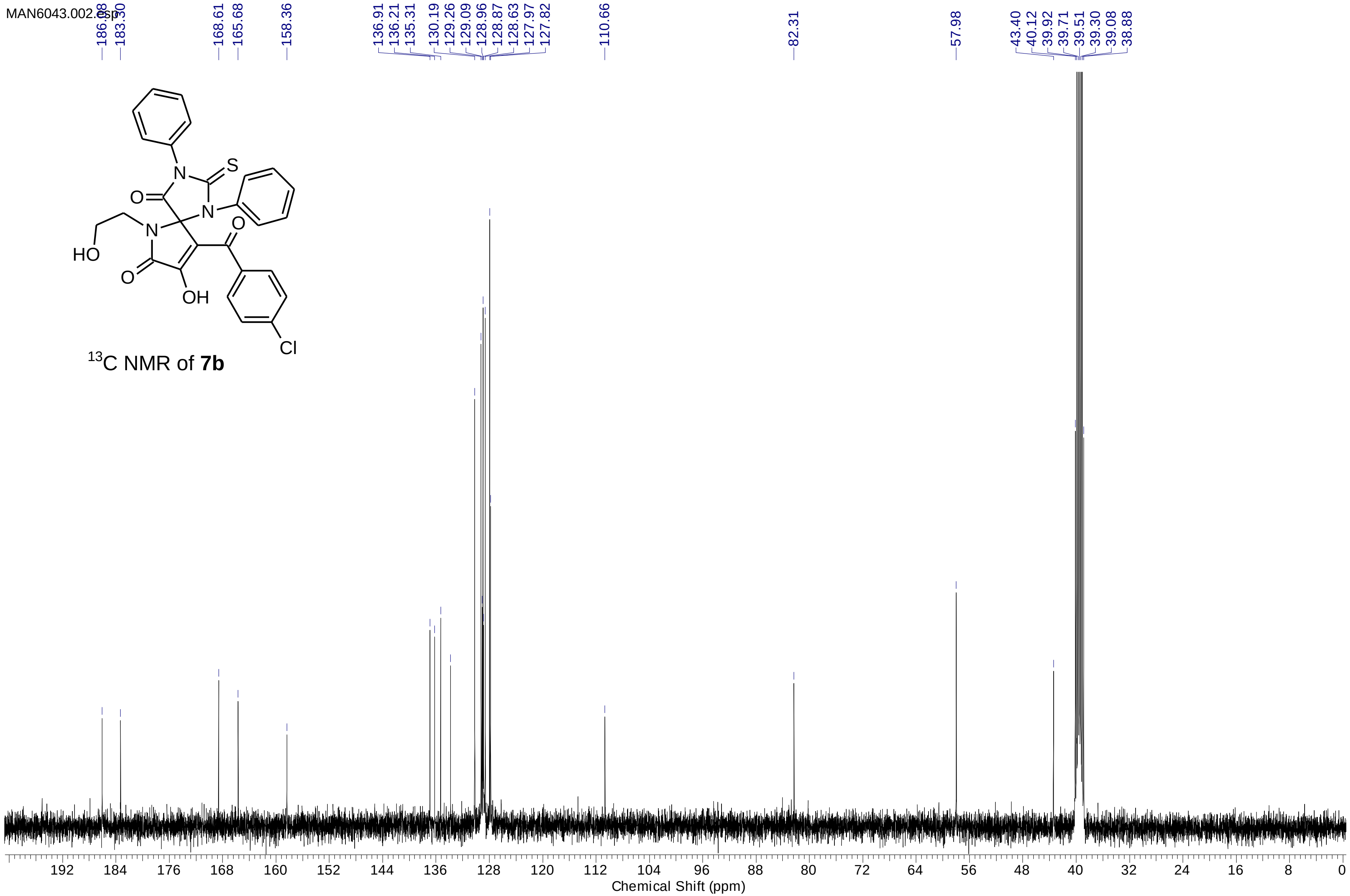
MAN6043.002.esp
186.88
183.30
168.61
165.68
158.36
136.91
136.21
135.31
130.19
129.26
129.09
128.96
128.87
128.63
127.97
127.82
110.66
82.31
57.98
43.40
40.12
39.92
39.71
39.51
39.30
39.08
38.88
S
N
O
N
O
N
HO
O
OH
Cl
192
184
176
168
160
152
144
136
128
120
112
104
96
88
80
72
64
56
48
40
32
24
16
8
0
Chemical Shift (ppm)

^{13}C NMR of **7b**

MAN6042.001.esp

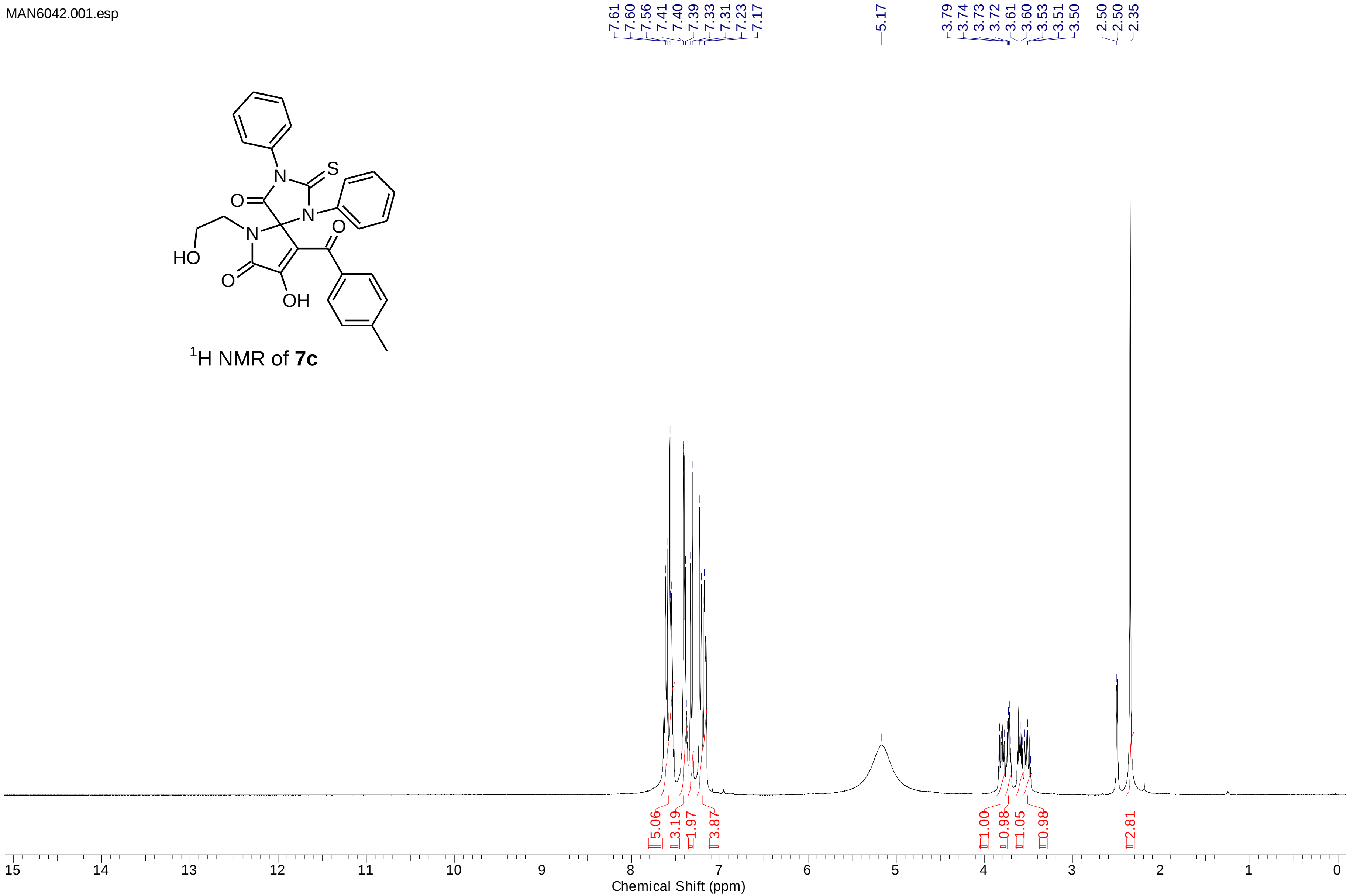

^{1}H NMR of **7c**

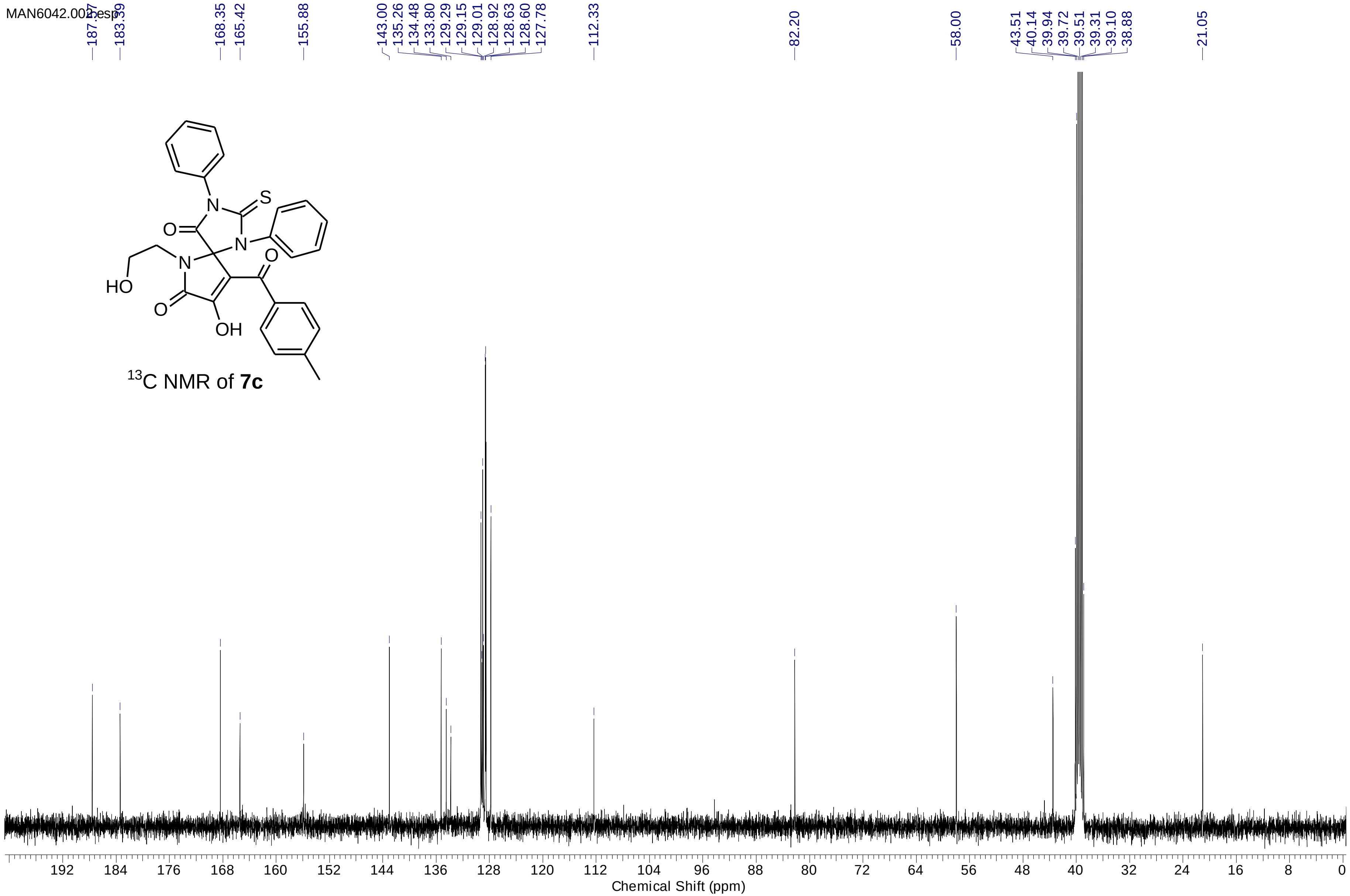

MAN6042.002.esp
187.27
183.39
168.35
165.42
155.88
143.00
135.26
134.48
133.80
129.29
129.15
129.01
128.92
128.63
128.60
127.78
112.33
82.20
58.00
43.51
40.14
39.94
39.72
39.51
39.31
39.10
38.88
21.05
192 184 176 168 160 152 144 136 128 120 112 104 96 88 80 72 64 56 48 40 32 24 16 8 0
Chemical Shift (ppm)

^{13}C NMR of **7c**

Pseudothiohydantoins 8a–c; General procedure

1,3-Diphenylthiourea (1.5 mmol, 342 mg) was added to a suspension of FPD **1a,l,m** (1.5 mmol) in anhydrous ethyl acetate (10 mL). The mixture was stirred at room temperature for 12 h. The formed solid was filtered off and recrystallized from methanol or toluene to afford the desired pseudothiohydantoin **8a–c** (if the product still contains impurities after the recrystallization, it can be purified by stirring it in ethyl acetate (5 mL) for 12–24h, and the subsequent filtration of the solid gives the desired pseudothiohydantoin).

9-Benzoyl-8-hydroxy-6-(2-hydroxyphenyl)-3-phenyl-2-(phenylimino)-1-thia-3,6-diazaspiro[4.4]non-8-ene-4,7-dione (8a)

Yield: 641 mg (78%); yellow solid; mp 155–157 °C (decomp.).

^{1}H NMR (400 MHz, DMSO-d_6): δ = 9.73 (br.s, 1 H), 7.84 (m, 2 H), 7.66–7.48 (m, 6 H), 7.43–7.31 (m, 3 H), 7.24 (m, 2 H), 7.13–6.98 (m, 4 H), 6.60 (m, 2 H).

^{13}C NMR (100 MHz, DMSO-d_6): δ = 188.1, 170.1, 164.7, 155.0, 154.2, 152.0, 147.2, 137.5, 135.2, 132.9, 131.1, 129.3 (2 C), 129.2 (2 C), 128.9 (2 C), 128.3, 128.1 (2 C), 127.9 (2 C), 124.5, 123.6, 120.3 (2 C), 120.0, 119.4, 117.1, 116.3, 77.4.

IR (mineral oil): 3195, 1713, 1666, 1641 cm^{-1}.

MS (ESI+): m/z calcd for $C_{31}H_{21}N_3O_5S+H^+$: 548.13 [M+H$^+$]; found: 548.13.

Anal. Calcd (%) for $10C_{31}H_{21}N_3O_5S \cdot C_7H_8$: C 68.38; H 3.95; N 7.55. Found: C 68.21; H 3.81; N 7.66.

9-(4-Chlorobenzoyl)-8-hydroxy-6-(2-hydroxyethyl)-3-phenyl-2-(phenylimino)-1-thia-3,6-diazaspiro[4.4]non-8-ene-4,7-dione (8b)

Yield: 657 mg (82%); yellow solid; mp 184–186 °C (decomp.).

^{1}H NMR (400 MHz, DMSO-d_6): δ = 7.81 (m, 2 H), 7.61–7.48 (m, 7 H), 7.31 (m, 2 H), 7.09 (m, 1 H), 6.90 (m, 2 H), 3.71–3.61 (m, 2 H), 3.60–3.54 (m, 2 H).

^{13}C NMR (100 MHz, DMSO-d_6): δ = 186.8, 169.8, 165.6, 154.8, 152.1, 154.5, 137.5, 136.3, 135.5, 130.8 (2 C), 129.2 (2 C), 129.0 (2 C), 128.7, 128.2 (2 C), 128.1 (2 C), 124.5, 120.5 (2 C), 116.2, 76.0, 58.2, 43.2.

IR (mineral oil): 3300, 1720, 1710, 1669, 1650 cm^{-1}.

MS (ESI+): m/z calcd for $C_{27}H_{20}ClN_3O_5S+H^+$: 534.09 [M+H$^+$]; found: 534.18.

Anal. Calcd (%) for $C_{27}H_{20}ClN_3O_5S$: C 60.73; H 3.78; N 7.87. Found: C 60.96; H 3.79; N 7.81.

8-Hydroxy-6-(2-hydroxyethyl)-9-(4-methylbenzoyl)-3-phenyl-2-(phenylimino)-1-thia-3,6-diazaspiro[4.4]non-8-ene-4,7-dione (8c)

Yield: 586 mg (76%); yellow solid; mp 173–175 °C (decomp.).

^{1}H NMR (400 MHz, DMSO-d_6): δ = 7.72 (m, 2 H), 7.59 (m, 4 H), 7.51 (m, 1 H), 7.31 (m, 4 H), 7.09 (m, 1 H), 6.89 (m, 2 H), 3.73–3.54 (m, 4 H), 2.38 (s, 3 H).
^{13}C NMR (100 MHz, DMSO-d_6): δ = 187.8, 169.8, 165.7, 153.2, 152.1, 147.5, 143.3, 135.6, 134.9, 129.2 (4 C), 129.0 (2 C), 128.7 (2 C), 128.6, 128.1 (2 C), 124.5, 120.6 (2 C), 117.0, 76.1, 58.2, 43.3, 21.1.
IR (mineral oil): 3300, 1721, 1712, 1669 cm^{-1}.
MS (ESI+): m/z calcd for $C_{28}H_{23}N_3O_5S+H^+$: 514.14 [$M+H^+$]; found: 514.18.
Anal. Calcd (%) for $C_{28}H_{23}N_3O_5S$: C 65.48; H 4.51; N 8.18. Found: C 65.73; H 4.58; N 8.23.

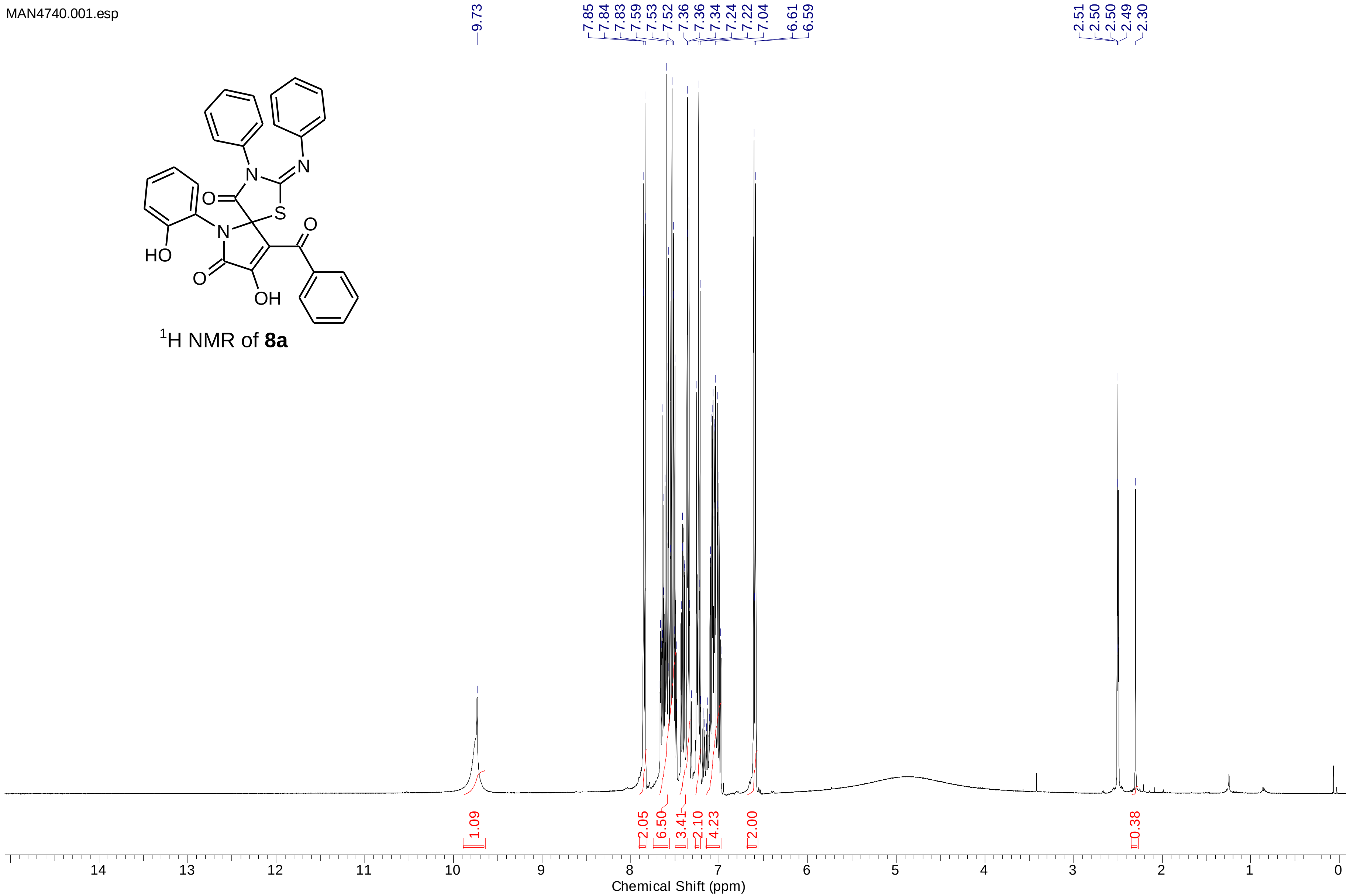

MAN4740.001.esp
9.73
7.85
7.84
7.83
7.59
7.53
7.52
7.36
7.36
7.34
7.24
7.22
7.04
6.61
6.59
2.51
2.50
2.50
2.49
2.30
HO
O
N
N
S
N
O
O
OH
1.09
2.05
6.50
3.41
2.10
4.23
2.00
0.38
Chemical Shift (ppm)

^{1}H NMR of **8a**

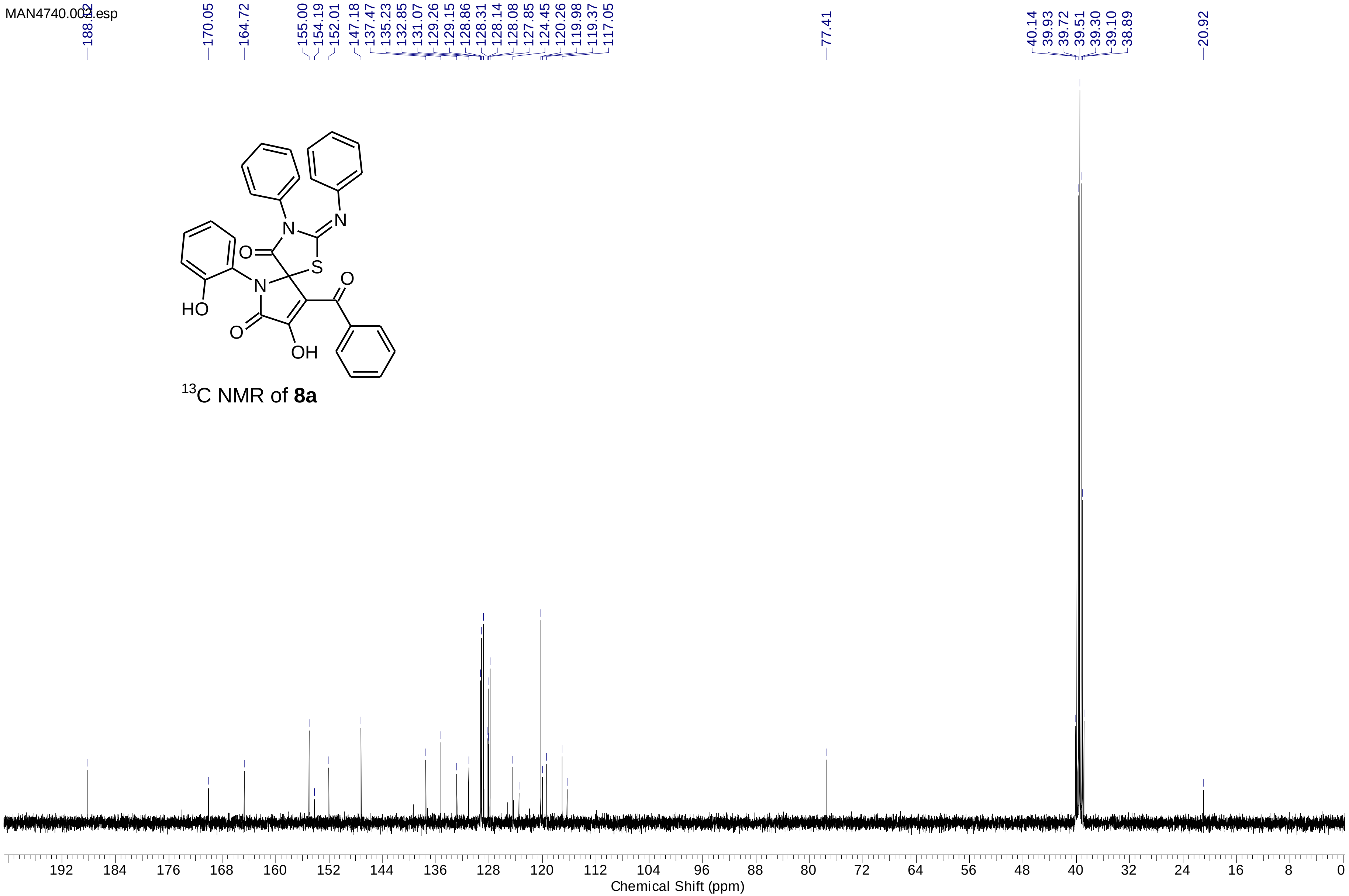
MAN4740.002.esp
188.12
170.05
164.72
155.00
154.19
152.01
147.18
137.47
135.23
132.85
131.07
129.26
129.15
128.86
128.31
128.14
128.08
127.85
124.45
120.26
119.98
119.37
117.05
77.41
40.14
39.93
39.72
39.51
39.30
39.10
38.89
20.92
O
N
N
S
O
N
HO
O
OH
192
184
176
168
160
152
144
136
128
120
112
104
96
88
80
72
64
56
48
40
32
24
16
8
0
Chemical Shift (ppm)

^{13}C NMR of **8a**

MAN6035.001.esp

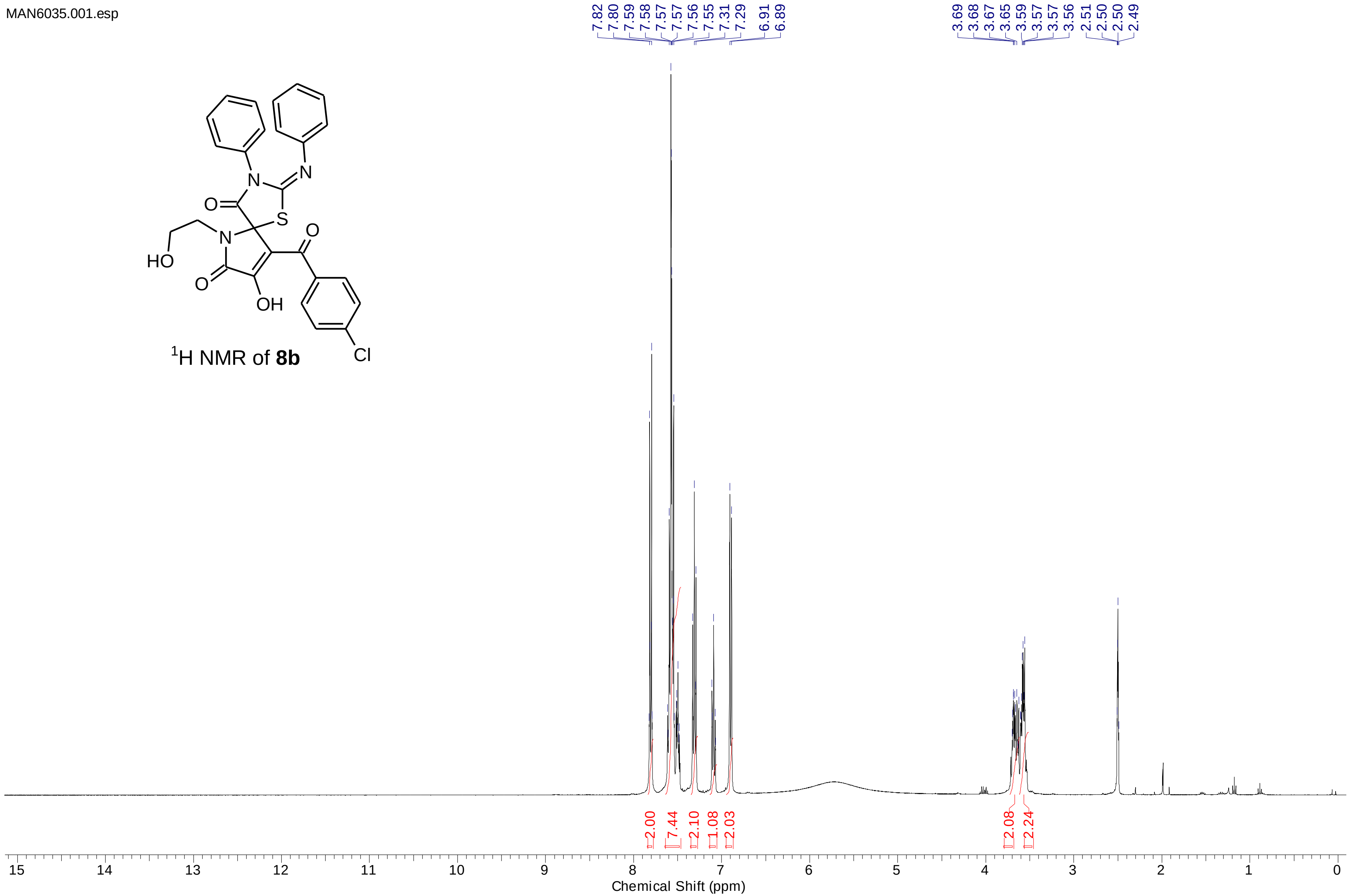

^{1}H NMR of **8b**

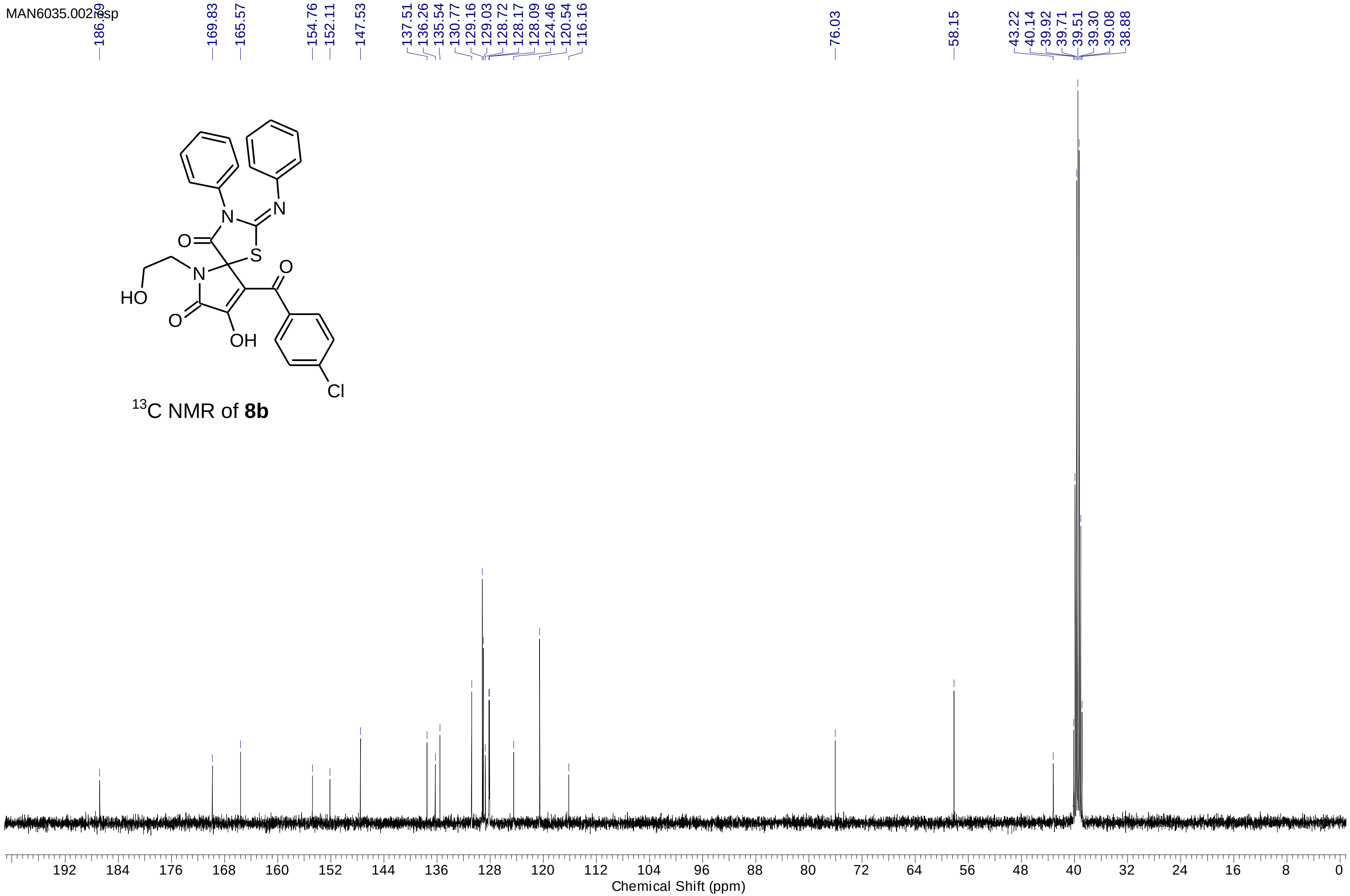
MAN6035.002.esp
186.79
169.83
165.57
154.76
152.11
147.53
137.51
136.26
135.54
130.77
129.16
129.03
128.72
128.17
128.09
124.46
120.54
116.16
76.03
58.15
43.22
40.14
39.92
39.71
39.51
39.30
39.08
38.88
O
N
N
S
O
N
HO
O
OH
O
Cl
192
184
176
168
160
152
144
136
128
120
112
104
96
88
80
72
64
56
48
40
32
24
16
8
0
Chemical Shift (ppm)

^{13}C NMR of **8b**

MAN6036.001.esp

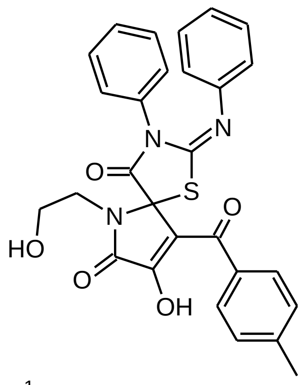

^{1}H NMR of **8c**

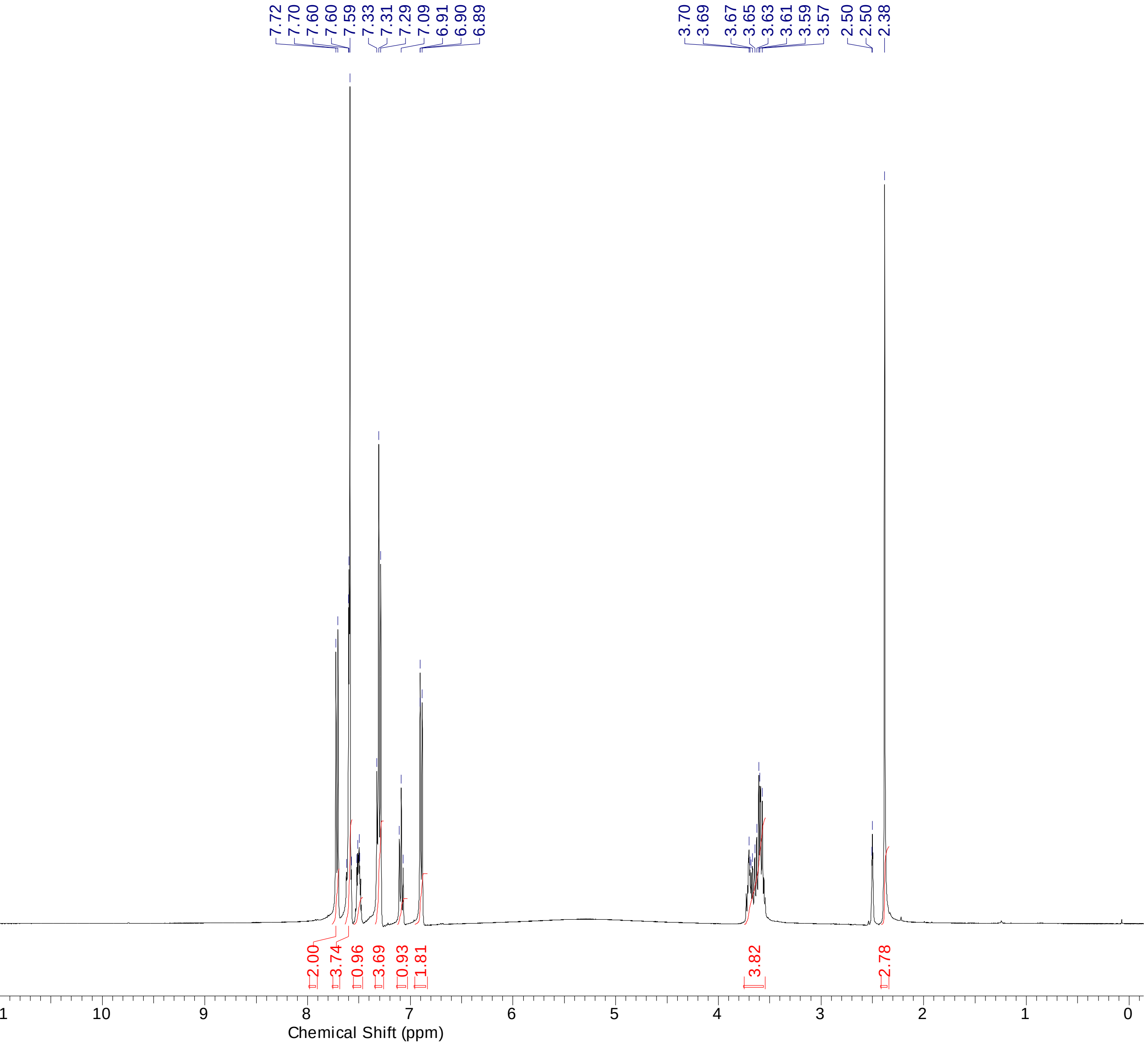

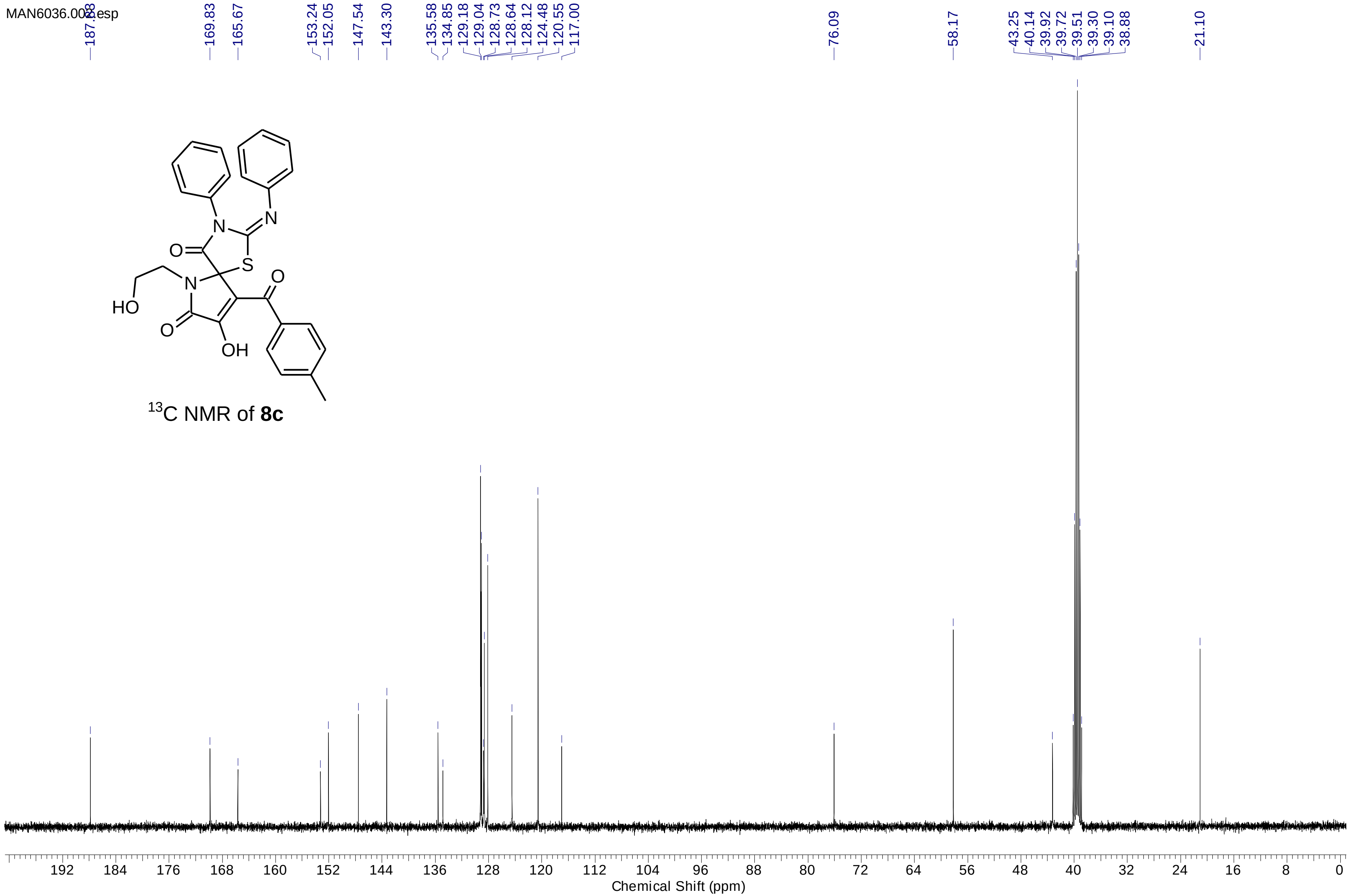

MAN6036.002.esp
187.68
169.83
165.67
153.24
152.05
147.54
143.30
135.58
134.85
129.18
129.04
128.73
128.64
128.12
124.48
120.55
117.00
76.09
58.17
43.25
40.14
39.92
39.72
39.51
39.30
39.10
38.88
21.10
^{13}C NMR of **8c**
Chemical Shift (ppm)

Crystal structure determination

The unit cell parameters and the X-ray diffraction intensities were measured on a Xcalibur Ruby diffractometer. The empirical absorption correction was introduced by multi-scan method using SCALE3 ABSPACK algorithm[2]. Using the Olex2[3], the structures were solved with the SHELXS[4], SHELXT[5] or SUPERFLIP[6] programs and refined by the full-matrix least-squares method in the anisotropic approximation for all non-hydrogen atoms with the SHELXL program[7]. Hydrogen atoms bound to carbon were located from the Fourier synthesis of the electron density and refined using a riding model. The hydrogen atoms of NH, NH_2 and OH groups were refined independently with isotropic displacement parameters.

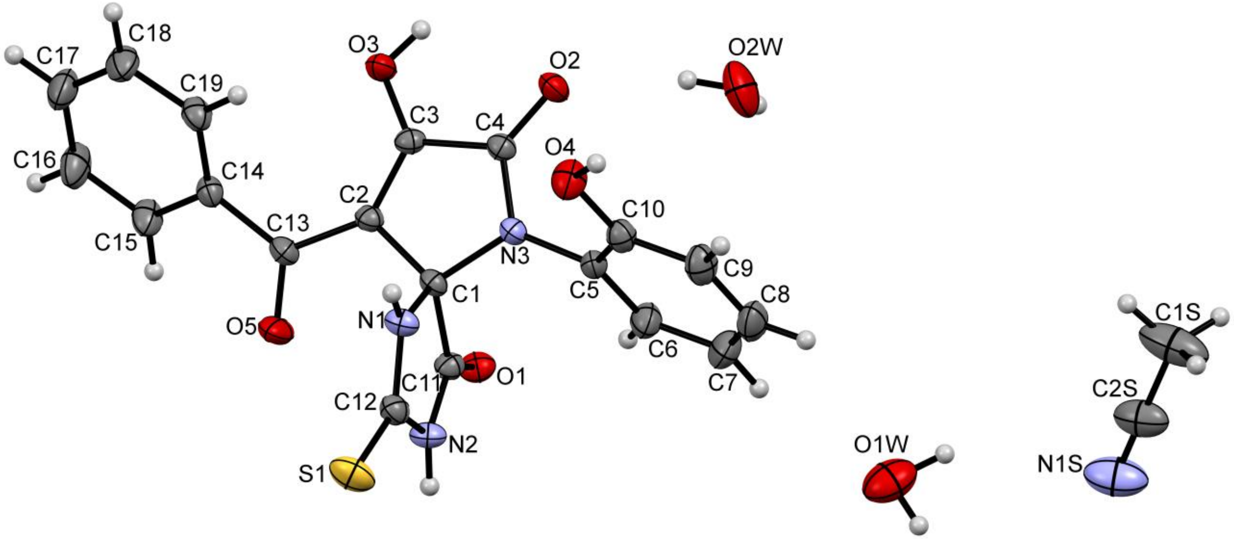

Figure S1. *Molecular structure of compound **2a** showing 30% probability amplitude displacement ellipsoids (CCDC 1952743).*

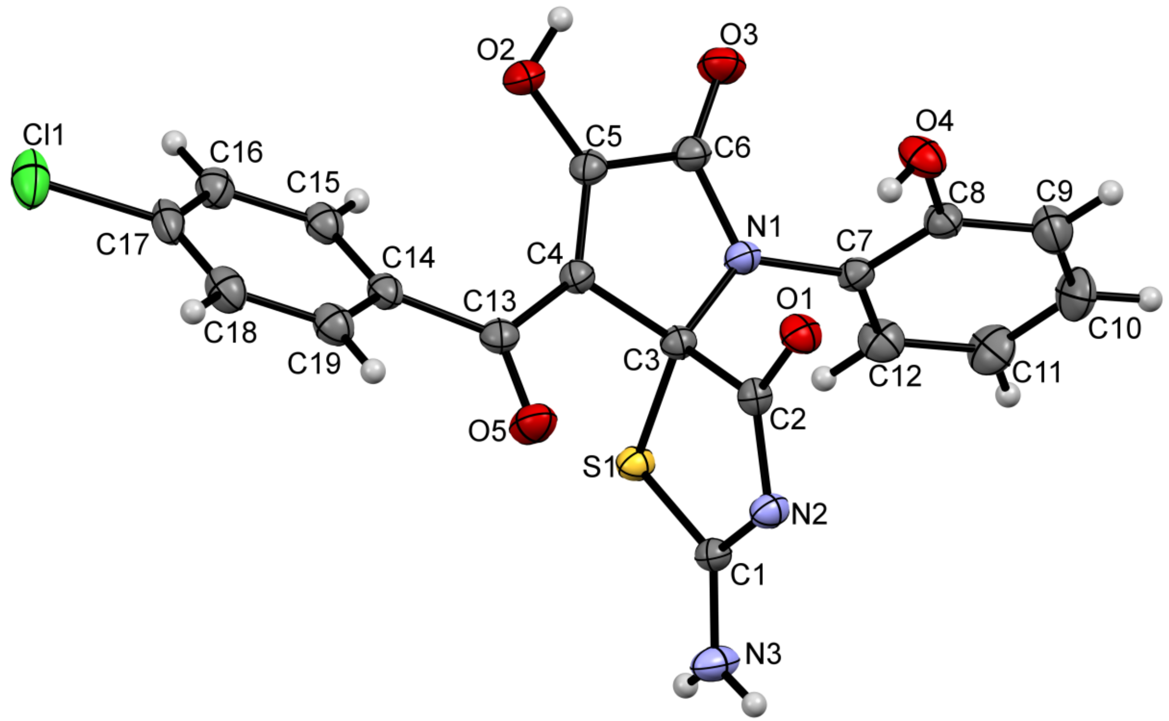

Figure S2. *Molecular structure of compound* ***3e*** *showing 30% probability amplitude displacement ellipsoids (CCDC 1952745).*

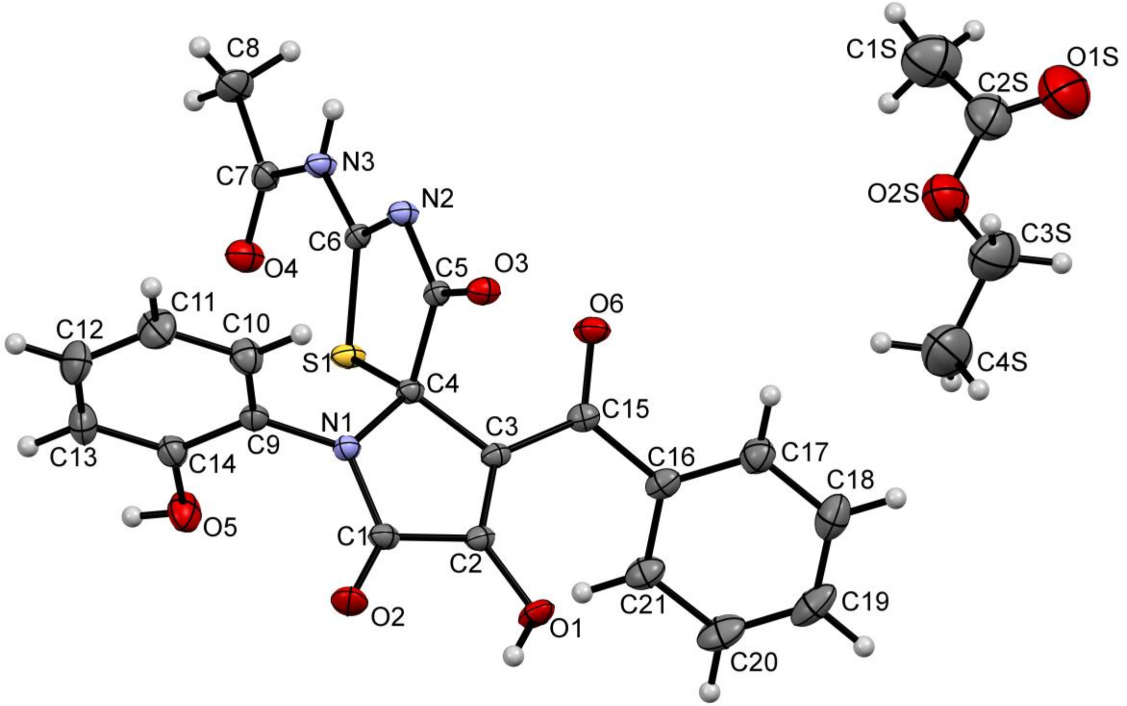

Figure S3. *Molecular structure of compound* ***4a*** *showing 30% probability amplitude displacement ellipsoids (CCDC 1952746).*

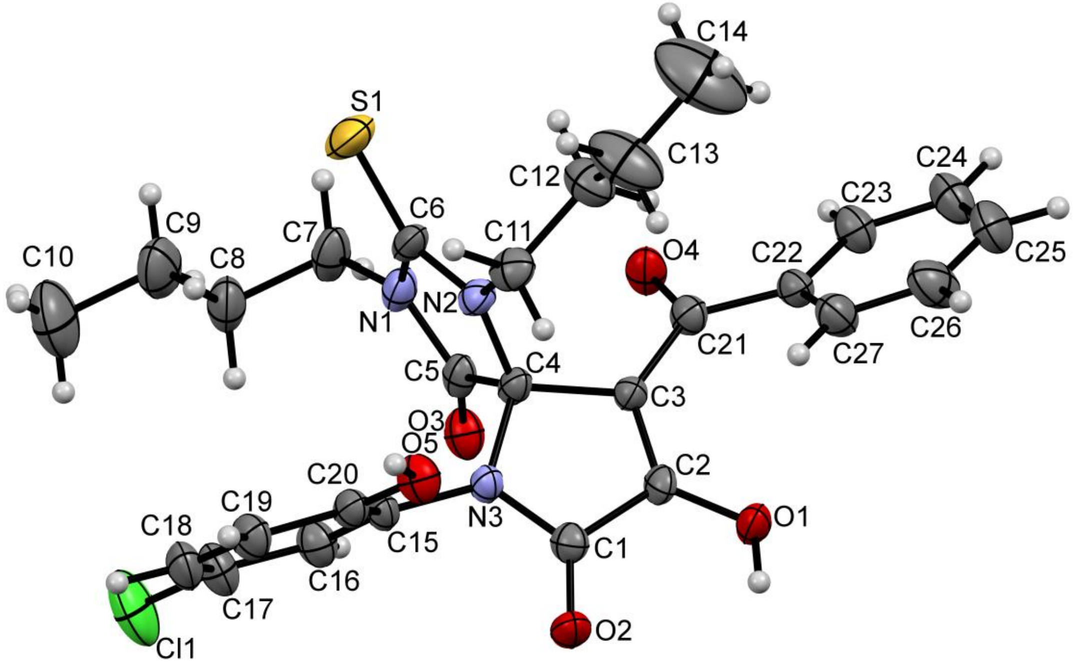

Figure S4. *Molecular structure of compound* ***5b*** *showing 30% probability amplitude displacement ellipsoids (CCDC 1952744).*

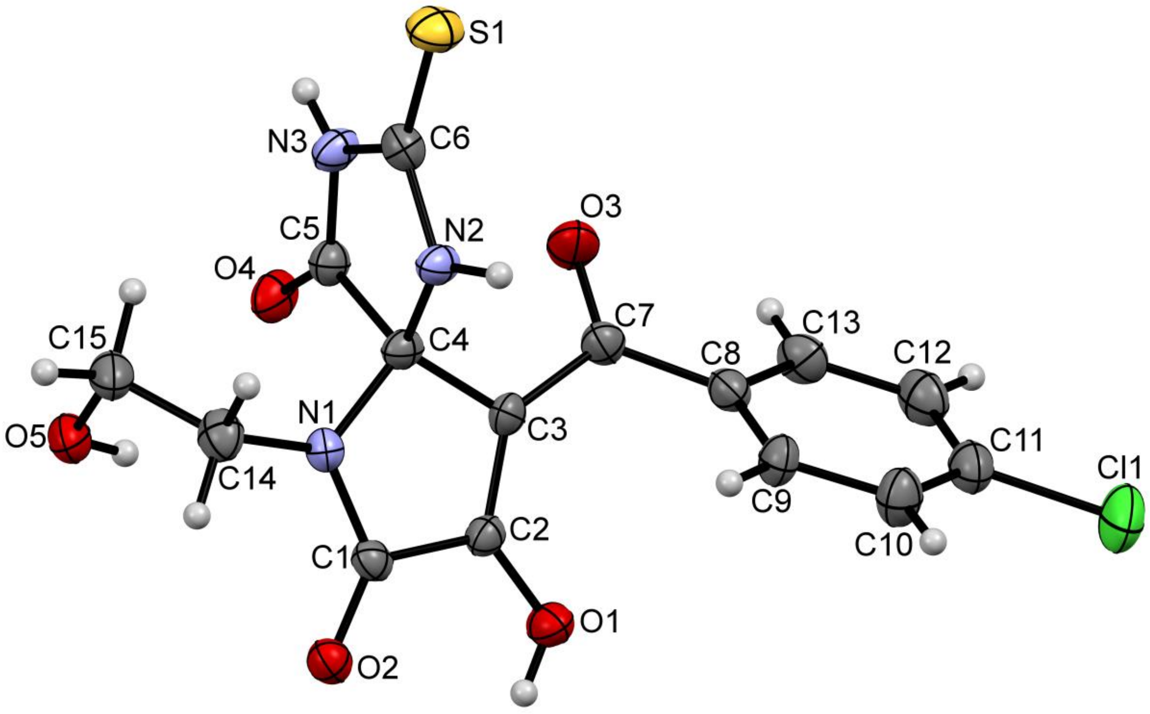

Figure S5. *Molecular structure of compound **2l** showing 30% probability amplitude displacement ellipsoids (CCDC 1952798).*

Table S1. Crystal data and structure refinement for compound **2a**.

Empirical formula	$C_{19}H_{13}N_3O_5S·2H_2O·C_2H_3N$
M_r	472.47
Temperature (K)	295
Radiation type, wavelength	Mo*K*α radiation, λ = 0.71073 Å
Scan mode	ω scans
Crystal system, space group	Monoclinic, $P2_1/c$
Unit cell dimensions a, b, c (Å)	19.181(3), 8.5178(15), 13.822(2)
β (°)	92.608(15)

Volume (Å^3)	2255.8(7)
Z	4
Calculated density (g/cm^3)	1.391
Absorption coefficient μ (mm^{-1})	0.194
Crystal size (mm)	0.43 × 0.19 × 0.02
Theta range for data collection	θ_{max} = 29.4°, θ_{min} = 2.8°
Limiting indices	h = −26→22<br>k = −8→11<br>l = −16→18
No. of measured, independent and observed [I > 2σ(I)] reflections	13361, 5353, 3164
R_{int}	0.057
Completeness to θ = 26.0°	0.999
Absorption correction	Empirical absorption correction using spherical harmonics, implemented in SCALE3 ABSPACK scaling algorithm[2].
Min. and max. transmission	0.611, 1.000
Method of structure solution and program	Charge flipping method; SUPERFLIP program[6]
Method of refinement and program	Full-matrix least-squares method on F^2; SHELXL[7]
Treatment of H atoms	Mixed refinement
Data / parameters / restraints	5353 / 328 / 2
Goodness-of-fit on F^2	1.04
Final R indices [I>2 σ (I)]	R_1 = 0.0648, wR_2 = 0.1538
R indices (all data)	R_1 = 0.1116, wR_2 = 0.1937
Largest diff. peak and hole (eÅ^{-3})	0.34, −0.40
CCDC	1952743

Table 2. Crystal data and structure refinement for compound **3e**.

Empirical formula	$C_{19}H_{12}ClN_3O_5S\cdot CCl_4$
M_r	583.64
Temperature (K)	295
Radiation type, wavelength	Mo*K*α radiation, λ = 0.71073 Å
Scan mode	ω scans
Crystal system, space group	Triclinic, P–1
Unit cell dimensions a, b, c (Å)	6.9143(9), 12.6189(17), 14.0439(19)
α, β, γ (°)	75.919(12), 89.906(11), 83.597(11)
Volume (Å^3)	1180.7(3)
Z	2
Calculated density (g/cm^3)	1.642
Absorption coefficient μ (mm^{-1})	0.74
Crystal size (mm)	0.47 × 0.34 × 0.15
Theta range for data collection	θ_{max} = 29.6°, θ_{min} = 3.0°
Limiting indices	h = −9→9<br>k = −16→13<br>l = −18→16
No. of measured, independent and observed [I > 2σ(I)] reflections	9554, 5492, 3785
R_{int}	0.046
Completeness to θ = 26.0°	0.998
Absorption correction	Empirical absorption correction using spherical harmonics, implemented in SCALE3 ABSPACK scaling algorithm[2].
Min. and max. transmission	0.904, 1.000
Method of structure solution and program	Direct method; SHELXS program[4]
Method of refinement and program	Full-matrix least-squares method on F^2; SHELXL[7]
Treatment of H atoms	Mixed refinement
Data / parameters / restraints	5492 / 276 / 0

Goodness-of-fit on F^2	1.02
Final R indices [I>2 σ (I)]	R_1 = 0.0573, wR_2 = 0.1465
R indices (all data)	R_1 = 0.0821, wR_2 = 0.1674
Largest diff. peak and hole (eÅ^{-3})	0.47, −0.38
CCDC	1952745

Table S3. Crystal data and structure refinement for compound **4a**.

Empirical formula	$C_{21}H_{15}N_3O_6S·C_4H_8O_2$
M_r	525.52
Temperature (K)	295
Radiation type, wavelength	Mo*K*α radiation, λ = 0.71073 Å
Scan mode	ω scans
Crystal system, space group	Monoclinic, C2/c
Unit cell dimensions a, b, c (Å)	19.203(5), 14.590(3), 21.458(6)
β (°)	122.16(4)
Volume (Å^3)	5089(3)
Z	8
Calculated density (g/cm^3)	1.372
Absorption coefficient μ (mm^{-1})	0.18
Crystal size (mm)	0.58 × 0.37 × 0.09
Theta range for data collection	$θ_{max}$ = 29.3°, $θ_{min}$ = 3.0°
Limiting indices	h = −17→25<br>k = −16→19<br>l = −26→23
No. of measured, independent and observed [I > 2σ(I)] reflections	12267, 5914, 4300
R_{int}	0.039
Completeness to θ = 26.0°	0.998
Absorption correction	Empirical absorption correction using spherical harmonics, implemented in SCALE3 ABSPACK scaling algorithm[2].

Min. and max. transmission	0.771, 1.000
Method of structure solution and program	Charge flipping method; SUPERFLIP program[6]
Method of refinement and program	Full-matrix least-squares method on F^2; SHELXL[7]
Treatment of H atoms	Mixed refinement
Data / parameters / restraints	5914 / 349 / 0
Goodness-of-fit on F^2	1.03
Final R indices [I>2 σ (I)]	R_1 = 0.0543, wR_2 = 0.1247
R indices (all data)	R_1 = 0.0806, wR_2 = 0.1428
Largest diff. peak and hole (eÅ^{-3})	0.31, −0.31
CCDC	1952746

Table S4. Crystal data and structure refinement for compound **5b**.

Empirical formula	$C_{27}H_{28}ClN_3O_5S\cdot C_{16}H_{10}ClNO_3$
M_r	841.73
Temperature (K)	295
Radiation type, wavelength	Mo*K*α radiation, λ = 0.71073 Å
Scan mode	ω scans
Crystal system, space group	Triclinic, P–1
Unit cell dimensions a, b, c (Å)	9.0037(19), 14.832(3), 16.627(3)
α, β, γ (°)	71.876(17), 82.715(17), 76.222(18)
Volume (Å^3)	2046.3(7)
Z	2
Calculated density (g/cm^3)	1.366
Absorption coefficient μ (mm^{-1})	0.27
Crystal size (mm)	0.35 × 0.14 × 0.02
Theta range for data collection	θ_{max} = 29.5°, θ_{min} = 2.9°
Limiting indices	h = −12→9<br>k = −19→20<br>l = −22→18

No. of measured, independent and observed [I > 2σ(I)] reflections	18529, 9626, 3255
R_{int}	0.081
Completeness to θ = 26.0°	0.999
Absorption correction	Empirical absorption correction using spherical harmonics, implemented in SCALE3 ABSPACK scaling algorithm[2].
Min. and max. transmission	0.468, 1.000
Method of structure solution and program	Direct method; SHELXT program[5]
Method of refinement and program	Full-matrix least-squares method on F^2; SHELXL[7]
Treatment of H atoms	Mixed refinement
Data / parameters / restraints	9626 / 537 / 0
Goodness-of-fit on F^2	0.96
Final R indices [I>2 σ (I)]	R_1 = 0.0679, wR_2 = 0.1310
R indices (all data)	R_1 = 0.2242, wR_2 = 0.1942
Largest diff. peak and hole (eÅ^{-3})	0.24, −0.30
CCDC	1952744

Table S5. Crystal data and structure refinement for compound **2l**.

Empirical formula	$C_{15}H_{12}ClN_3O_5S·0.5C_4H_8O_2$
M_r	425.84
Temperature (K)	295
Radiation type, wavelength	Mo*K*α radiation, λ = 0.71073 Å
Scan mode	ω scans
Crystal system, space group	Triclinic, P–1
Unit cell dimensions a, b, c (Å)	6.0732(14), 11.738(3), 13.807(2)
α, β, γ (°)	107.033(18), 100.280(18), 91.836(18)
Volume (Å^3)	922.3(3)
Z	2
Calculated density (g/cm^3)	1.533

Absorption coefficient μ (mm^{-1})	0.36
Crystal size (mm)	0.38 × 0.16 × 0.02
Theta range for data collection	θ_{max} = 29.6°, θ_{min} = 3.1°
Limiting indices	h = −6→8<br>k = −16→15<br>l = −16→18
No. of measured, independent and observed [I > 2σ(I)] reflections	7041, 4272, 2287
R_{int}	0.070
Completeness to θ = 26.0°	0.999
Absorption correction	Empirical absorption correction using spherical harmonics, implemented in SCALE3 ABSPACK scaling algorithm[2].
Min. and max. transmission	0.775, 1.000
Method of structure solution and program	Direct method; SHELXS program[4]
Method of refinement and program	Full-matrix least-squares method on F^2; SHELXL[7]
Treatment of H atoms	Mixed refinement
Data / parameters / restraints	4272 / 285 / 41
Goodness-of-fit on F^2	1.03
Final R indices [I>2 σ (I)]	R_1 = 0.0720, wR_2 = 0.1785
R indices (all data)	R_1 = 0.1257, wR_2 = 0.2256
Largest diff. peak and hole ($eÅ^{-3}$)	0.50, −0.42
CCDC	1952798

CCDC 1952743 [**2a**], 1952745 [**3e**], 1952746 [**4a**], 1952744 [**5b**], and 1952798 [**2l**] contain the supplementary crystallographic data for this paper. The data can be obtained free of charge from The Cambridge Crystallographic Data Centre *via* http://www.ccdc.cam.ac.uk.

Biological evaluation of the reported products

To the moment of the manuscript submission, a library of new compounds reported in this paper was submitted to CO-ADD (https://www.co-add.org/) (project P0795) for antimicrobial evaluation, and the data on the antimicrobial properties of these compounds will be available at the free database at https://db.co-add.org/screening-data/.

Some preliminary antimicrobial data for the reported compounds is available in our previous work [8].

Comparison of structures of the compounds 2 and 3 reported in our previous works and in the present paper

In our first paper on reaction of FPDs **1** with thiourea [8, or ref. 30 from the main paper], we assumed the products structure as ((imidazolidinylidene)ethyl)-1,4-benzoxazin-2-ones **A** (Scheme 1). The structure was proposed on the basis of known at that moment chemical properties of 1*H*-pyrrole-2,3-diones and isatins (their closest analogs) prone to a pyrrole cycle cleavage under the action of nucleophiles [9] and data of quantum-chemical calculations (no single crystal X-ray diffraction data on the products of reaction of FPDs **1** and thiourea was available at that moment). The spectral data (NMR, IR) did not conflict with the proposed structure **A**. Since the products of this reaction had shown antimicrobial activity [8], the research on it continued.

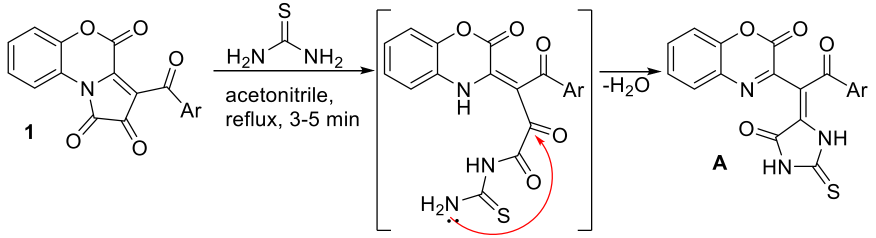

Scheme S1. Reaction of FPDs **1** with thiourea according to the first paper on it [8].

After the discovery of one of the most amazing and important chemical properties of some 1*H*-pyrrole-2,3-diones, their ability to afford spiro-bis-heterocyclic compounds under the action of 1,3-binucleophiles [for the first reaction of this type, see 10], the structure **A** of the products of reaction of FPDs **1** and thiourea was revised as thiohydantoins **B** (Scheme S2) [11, or ref. 31 from the main paper]. The structure was proposed on the basis of the NMR spectra similarity of products **B** and spiro-products **C** [10b] (no single crystal X-ray diffraction data on the products of reaction of FPDs **1** and thiourea was available at that moment). The spectral data (NMR, IR) did not conflict with the proposed structure **B**. The choice of thiohydantoin structure **B** was made without the considering a possible regioisomeric pseudothiohydantoin.

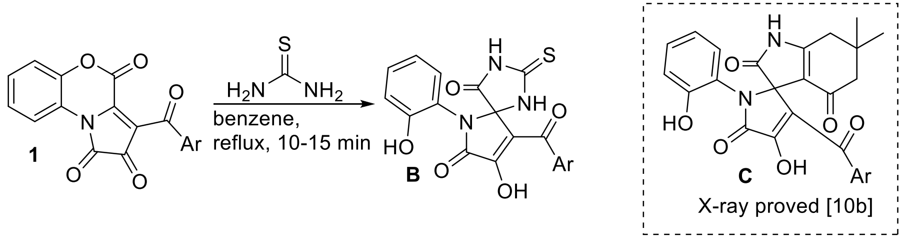

Scheme S2. Reaction of FPDs **1** with thiourea according to the second paper on it [11].

Finally, in the present paper, we provide all information on the reaction of FPDs **1** and thiourea with the employment of single crystal X-ray diffraction data for all regioisomers **2** and **3** (Scheme S3).

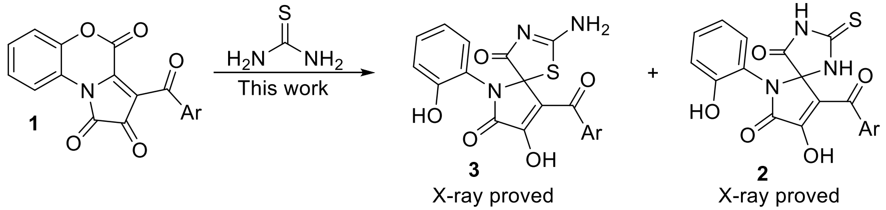

Scheme S3. Reaction of FPDs **1** with thiourea according to the present paper.

Comparison of spectral data of structures **A**, **B** and **2**, **3** is given in Table S6.

Table S6: Comparison of spectral data of structures **A**, **B** and **2**, **3**.

	**A** (compound XII) [8]	**B** (compound V) [11]	**2a** [the present paper]	**3a** [the present paper]
	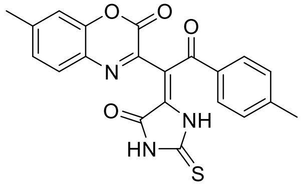	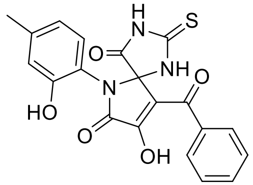	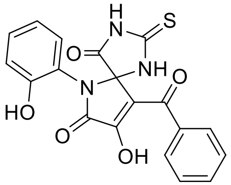	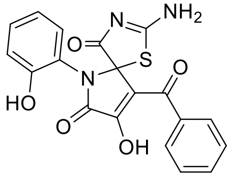
^{1}H NMR (δ, ppm, DMSO-d_6)[a]	8.33–8.93, 9.65–11.08	8.85, 9.13, 9.43, 12.50	9.80, 10.10, 11.88	8.80, 9.07, 9.54
^{13}C NMR (δ, ppm, DMSO-d_6)[a]	80.6, 154.8, 155.6 ($\underline{C}_{Ar}$–Me), 157.4 ($\underline{C}_{Ar}$–Me), 165.7, 172.9, 189.4	85.20, 153.05 ($\underline{C}_{Ar}$–Me), 154.45, 164.96, 183.73, 188.00	80.6, 154.8, 164.2, 171.7, 183.4, 187.7	85.2, 154.7, 164.9, 179.6, 183.8, 187.8
NMR conditions	^{1}H NMR: RYa-2310 (60 MHz), Bruker WP-80-54 (80 MHz) spectrometers. ^{13}C NMR: Bruker HX-90 spectrometer (22.63 MHz).	^{1}H NMR: Bruker DRX 400 (400 MHz) spectrometer. ^{13}C NMR: Bruker DRX 400 (100 MHz) spectrometer.	^{1}H NMR: Bruker Avance-III HD 400 (400 MHz) spectrometer. ^{13}C NMR: Bruker Avance-III HD 400 (100 MHz) spectrometer.	

[a]Characteristic signals (signals except aromatic and aliphatic groups' signals) are given only.

From Table 1, it is obvious, that spectral data of regioisomeric compounds **2** and **3** is very similar, and it is impossible to distinguish them from each other using only the NMR spectra without any reference regioisomer.

So, now we can confirm that the real structure of compound XII from [8] is thiohydantoin **D** (Figure S6). Slight differences in characteristic NMR signals (a missing signal at about 183 ppm at ^{13}C NMR of compound XII [8]) can be explained by the poorer quality of NMR spectra obtained with an older NMR instrument [8].

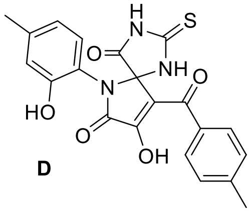

Figure S6. Structure of compound XII from [8] according the present paper's data.

And we can confirm that the real structure of compound V from [11] is pseudothiohydantoin **E** (Figure S7).

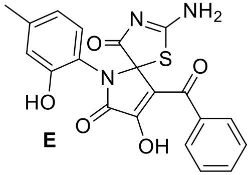

Figure S7. Structure of compound V from [11] according the present paper's data.

To explain differences in characteristic NMR signals (a missing signal at about 179 ppm at ^{13}C NMR of compound V [11]), we found and revised that NMR spectrum (Figures S3 and S4). It is obvious, that accidentally the signal at 179.6 ppm (Figure S4) was missed in the description of NMR of compound V in [11].

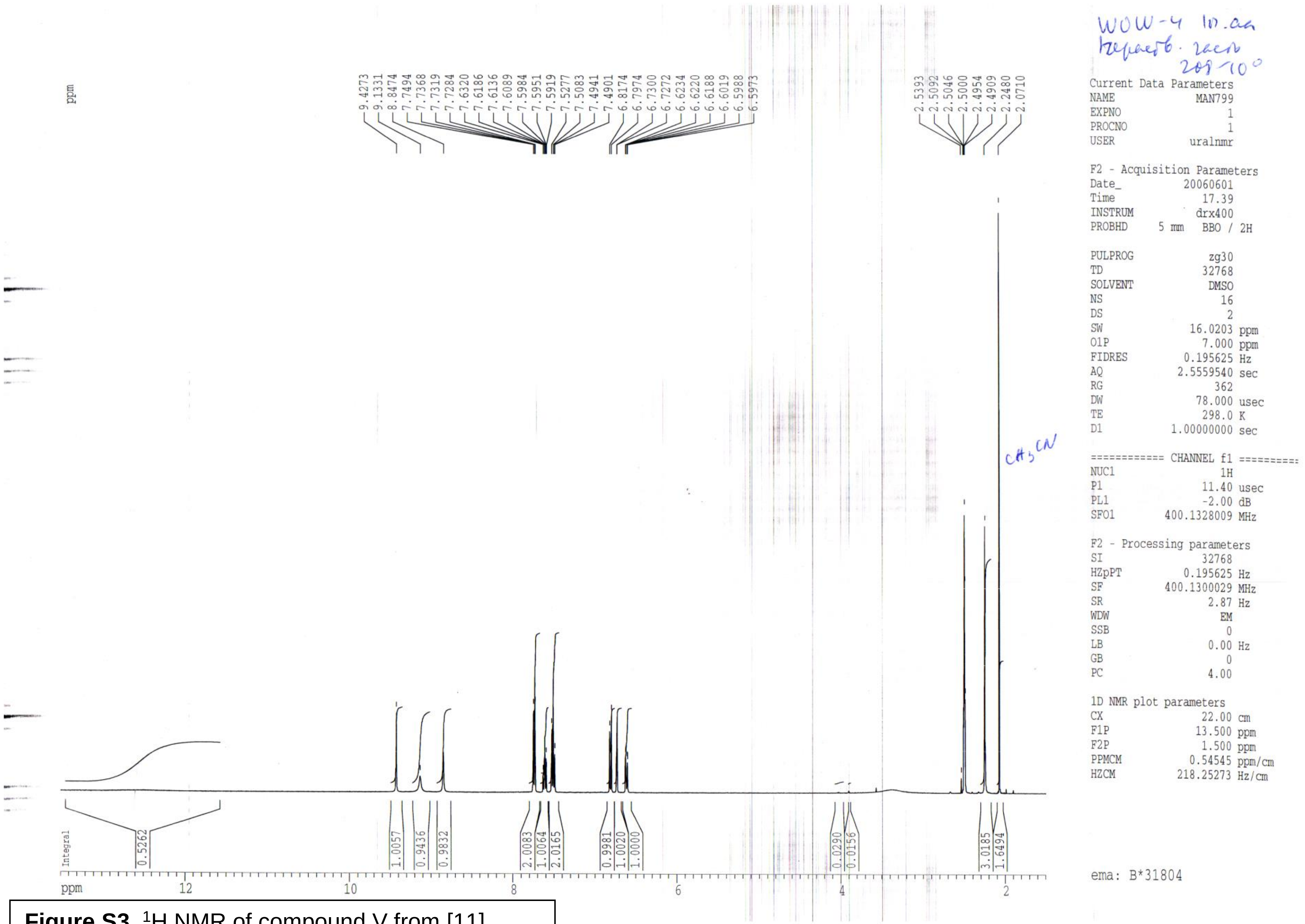

Figure S3. ^{1}H NMR of compound V from [11].

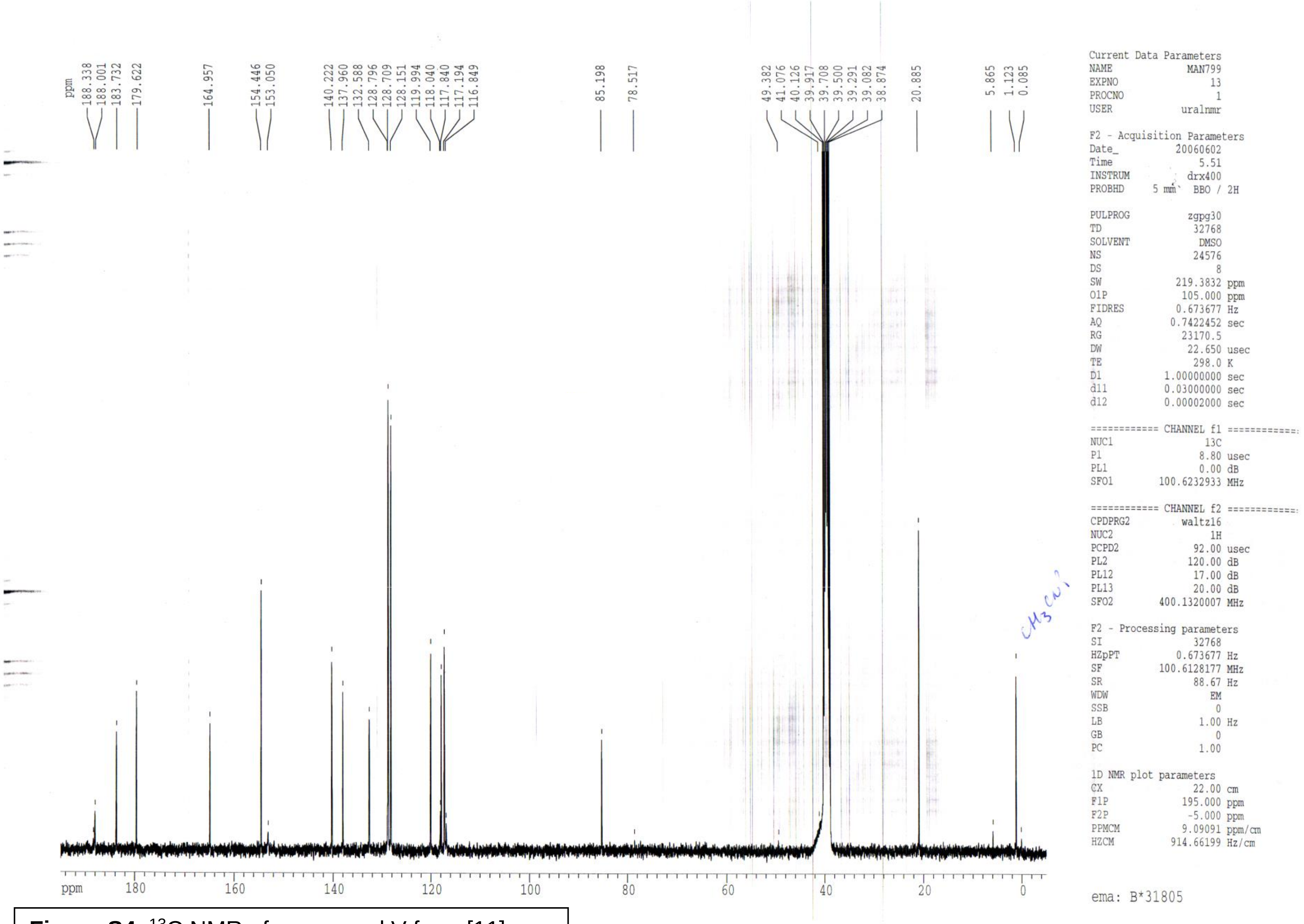

Figure S4. ^{13}C NMR of compound V from [11].

References:

1. (a) Bozdyreva K. S., Smirnova I. V., Maslivets A. N. *Russ. J. Org. Chem.,* **2005**, *41*, 1081; (b) Mashevskaya I. V., Mokrushin I. G., Bozdyreva K. S., Maslivets A. N. *Russ. J. Org. Chem.,* **2011**, *47*, 253; (c) Maslivets A. N., Mashevskaya I. V., Smirnova L. I., Krasnykh O. P., Shurov S. N., Andreichikov Yu. S. *Zh. Org. Khim.,* **1992**, *28*, 2545; (d) Stepanova E. E., Babenysheva A. V., Maslivets A. N. *Russ. J. Org. Chem.,* **2011**, *47*, 937; (e) Tretyakov N. A., Shavrina T. V., Maslivets A. N. *Russ. J. Org. Chem*., **2019**, *55*, 719.
2. CrysAlisPro, Agilent Technologies, Version 1.171.37.33 (release 27-03-2014 CrysAlis171 .NET).
3. Dolomanov O. V., Bourhis L. J., Gildea R. J., Howard J. A. K., Puschmann H. *J. Appl. Cryst.*, **2009**, *42*, 339.
4. Sheldrick G. M. *ActaCryst*. **2008**, *A64*, 112.
5. Sheldrick G. M. *ActaCryst*. **2015**, *A71*, 3.
6. Palatinus L., Chapuis G. *J. Appl. Cryst.* **2007***, 40*, 786.
7. Sheldrick G. M. *ActaCryst*. **2015**, *C71*, 3.
8. Mashevskaya I. V., Kol'tsova S. V., Voronina E. V., Odegova T. F., Maslivets A. N. *Pharm. Chem. J.*, **2001**, *35*, 18.
9. Yamagishi M., Ozaki K., Yamada Yo., Da-te T., Okamura K., Suzuki M. *Chem. Pharm. Bull.*, **1991**, *39*, 1694.
10. (a) Mashevskaya I. V., Tolmacheva I. A., Tiunova O. Yu., Aliev Z. G., Maslivets A. N. *Chem. of Heterocycl. Comp.*, **2002**, *38*, 500; (b) Mashevskaya I. V., Duvalov A. V., Tolmacheva I. A., Aliev Z. G., Maslivets A. N. *Russ. J. Org. Chem.,* **2004**, *40*, 1359.
11. Babenysheva A. V., Maslivets V. A., Maslivets A. N. *Russ. J. Org. Chem*., **2007**, *43*, 1577.
